# Anion‐Controlled Speciation and Stability of Silylzinc Reagents

**DOI:** 10.1002/open.70301

**Published:** 2026-09-15

**Authors:** Zhili Duan, Konrad Koszinowski

**Affiliations:** ^1^ Institut für Organische und Biomolekulare Chemie Universität Göttingen Göttingen Germany

**Keywords:** electrospray ionization (ESI)‐mass spectrometry, pivalate, protodemetalation, Schlenk equilibria, silylzinc reagents

## Abstract

Silylzinc reagents are valuable silicon‐based nucleophiles, yet their molecular‐level speciation and stability remain poorly understood. Here, we use a combination of electrospray ionization (ESI)‐mass spectrometry, NMR spectroscopy, and X‐ray crystallography to probe the speciation and coordination of (Ph_*z*_Me_3−*z*_Si)ZnX·LiX·LiCl (*z* = 1–3; X = OOC^*t*^Bu, Cl, Br, I) both in solution and the solid state. In the presence of *N*,*N*,*Nꞌ*,*Nꞌ*‐tetramethylethylenediamine (TMEDA), the (Ph_3_Si)ZnX·LiX·LiCl reagents with X = Cl and I, respectively, afford complexes (Ph_3_Si)ZnX(TMEDA), whose approximately tetrahedral coordination geometry resembles that of related compounds described previously. In tetrahydrofuran solutions, ESI‐mass spectrometry reveals the formation of mononuclear and polynuclear zincate species interconnected by Schlenk‐type equilibria, closely resembling organozinc systems. Upon exposure to ambient atmosphere, time‐resolved experiments uncover ligand‐dependent degradation pathways likely involving protodemetalation and oxidation via O_2_ insertion into the Zn─Si bond, with silanol formation directly observed by NMR spectroscopy. The stability of the silylzinc reagents depends sensitively on the identity of the anion X^−^. In particular, pivalate (X = OOC^*t*^Bu) coordination markedly suppresses decomposition, which we attribute to enhanced Li^+^ binding and aggregation effects, steric shielding, as well as proton scavenging. These findings provide a molecular‐level understanding of silylzinc speciation and establish anion coordination as a key factor influencing their stability.

## Graphical abstract

A combination of ESI‐mass spectrometry, ^1^H‐NMR spectroscopy, and X‐ray crystallography gives detailed insight into the speciation of silylzinc reagents. Time‐dependent experiments show an enhanced stability of silylzinc pivalates upon exposure to ambient atmosphere, which renders these compounds particularly interesting for synthetic applications.

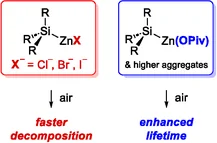

## 1 Introduction

Organozinc halides RZnX (X = Cl, Br, and I) are versatile reagents that play a central role in organic synthesis, including nucleophilic additions to carbonyl compounds and transition‐metal‐catalyzed cross‐coupling reactions (Negishi reactions) [1, 2, 3]. Their broad applicability arises from a well‐balanced reactivity, combining high nucleophilicity with excellent functional group tolerance. A related class of reagents, organozinc pivalates RZn(OPiv)‍·Mg(OPiv)X·*n*LiCl (OPiv = OOC^*t*^Bu), developed by Knochel and coworkers, exhibit enhanced stability toward moisture and air, making them particularly attractive for practical applications [4, 5, 6].

Given their widespread use, the molecular constitution of organozinc reagents has been studied extensively to afford detailed insight into their aggregation behavior and solution speciation [7, 13]. In this context, electrospray ionization (ESI)‐mass spectrometry and nuclear magnetic resonance (NMR) spectroscopy have proven especially powerful. In particular, ESI‐mass spectrometry has revealed the formation of anionic zincate species of the type RZnX_2_^−^ in the presence of LiX, arising either from deliberate addition or as a byproduct of transmetalation processes [14, 15, 16]. These studies have provided important insight into aggregation phenomena and Schlenk‐type equilibria in organozinc systems.

In contrast, the speciation and stability of the corresponding silylzinc reagents [17] have not been analyzed in comparable detail. These reagents are widely used as silyl nucleophile sources in transformations analogous to those of their organozinc counterparts [18] and thereby give access to organosilicon compounds, which find important applications, inter alia, in pharmaceutical chemistry [19] and material science [20]. Besides silylzinc halides, the corresponding pivalates have been developed, which, like their organozinc congeners, appear to be more resistant to hydrolysis and degradation [21]. However, a systematic understanding of the structure and decomposition pathways of the silylzinc reagents has not yet been established.

**SCHEME 1:**
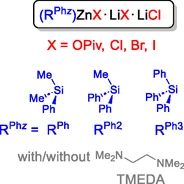
Silylzinc reagents investigated in this work.

Here, we combine ESI‐mass spectrometry, NMR spectroscopy, and X‐ray crystallography to establish a molecular‐level understanding of the speciation and stability of silylzinc reagents in tetrahydrofuran (THF). The investigated systems comprise (Ph_*z*_Me_3−*z*_Si)ZnX·LiX·LiCl, *z* = 1–3; X = OPiv, Cl, Br, and I (Scheme 1). Particular emphasis is placed on elucidating how anion identity influences aggregation, ion pairing, and decomposition pathways.

## 2 Results and Discussion

### 2.1 Mono‐ and Polynuclear Silylzincate Anions

ESI‐mass spectrometric analysis of freshly prepared THF solutions of (R^Ph*z*^)ZnX·LiX·LiCl revealed abundant ionic species, suggesting that zincate‐type aggregates contribute to the solution speciation (Figures 1a,b and S1–S36). Among the predominant anionic species, we consistently observed mononuclear zincates of the type (R^Ph*z*^)ZnX_2_^‒^ (Figures 1a and S1, S3, S7, S9, S11–S13, S17, S23, S25, S27, S29, S31, and S33), which could be easily identified by their characteristic isotope patterns (see Figures S12–S15 as representative examples). Besides (R^Ph*z*^)ZnX_2_^‒^ complexes, we also detected (R^Ph*z*^)_2_ZnX^‒^ ions in several cases (Figures 1a and S25, S27, S29, and S31). This finding points to the operation of Schlenk‐type equilibria (Scheme 2). We attribute the absence of the supposedly co‐generated ZnX_3_^–^ species in most of the recorded mass spectra to the higher tendency of these species to form contact‐ion pairs with the Li^+^ counter‐ions and/or their lower ESI activities (the ESI activity of a given analyte ion is known to correlate with its size and hydrophobicity) [22, 23, 24].

**FIGURE 1:**
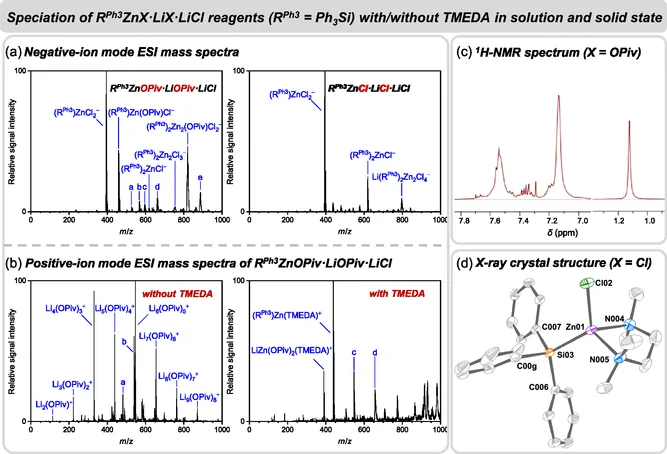
Speciation of R^Ph3^ZnX·LiX·LiCl reagents. (a) Negative ion‐mode ESI‐mass spectra of solutions of R^Ph3^Zn(OPiv)·Li(OPiv)·LiCl (left) and R^Ph3^ZnCl·2LiCl (right) in THF (*c* = 0.038 m). Peak assignments: a = (R^Ph3^)Zn(OPiv)_2_^−^, b = Li(R^Ph3^)Zn(OPiv)_2_Cl^−^, c = (R^Ph3^)Zn_2_(OPiv)Cl_3_^−^, d = (R^Ph3^)Zn_2_(OPiv)_2_Cl_2_^−^, e = (R^Ph3^)_2_Zn_2_(OPiv)_2_Cl^−^. (b) Positive ion‐mode ESI‐mass spectra of solutions of R^Ph3^Zn(OPiv)·Li(OPiv)·LiCl (left) and R^Ph3^Zn(OPiv)·Li(OPiv)·LiCl·TMEDA (right) in THF (*c* = 0.038 m). Peak assignments: a = Li_6_(OPiv)_4_Cl^+^, b = Li_2_(R^Ph3^)Zn(OPiv)_2_^+^, c = Li(R^Ph3^)Zn(OPiv)(TMEDA)^+^, d = Li_2_(R^Ph3^)Zn(OPiv)_2_(TMEDA)^+^. (c) Sections from the ^1^H‐NMR spectrum of a solution of R^Ph3^Zn(OPiv)·Li(OPiv)·LiCl in THF‐*d8* (*c* = 0.16 m). (d) X‐ray crystal structure of R^Ph3^ZnCl(TMEDA). Selected bond lengths (Å) and angles (◦): Zn01–Cl02 2.2688(8), Zn01–Si03 2.3839(8), Zn01–N004 2.145(2), Zn01–N005 2.128(2), Si03–C006 1.896(3), Si03–C007 1.893(3), Si03–C00g 1.895(3), Cl02–Zn01–Si03 120.22(3), N004–Zn01–Cl02 103.15(7), N004–Zn01–Si03 118.94(7), N005–Zn01–Cl02 105.32(7), N005–Zn01–Si03 117.77(7), N005–Zn01–N004 85.36(10), C006–Si03–Zn01 115.93(9), C007–Si03–Zn01 110.99(9), C007–Si03–C006 107.85(12), C007–Si03–C00G 105.60(12), C00g–Si03–Zn01 111.29(11), C00G–Si03–C006 104.48(13).

**SCHEME 2:**
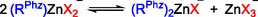
Schlenk‐type equilibrium of silylzincate complexes.

To varying degrees, we also observed polynuclear zincate species, including (R^Ph*z*^)_2_Zn_2_X_3_^−^ and Li(R^Ph*z*^)_2_Zn_2_X_4_^−^ (Figures 1a and S1, S3, S11, S14, S15, S17, S27, and S29). These polynuclear ions were most pronounced for complexes containing pivalate moieties and least conspicuous for those with X^−^ = Br^−^ or I^−^. As the solvent evaporation during the ESI process results in a strong increase of analyte concentrations in the formed microdroplets and the readjustment of association equilibria, ESI‐mass spectrometry typically is not capable of faithfully determining the absolute aggregation states of organometallic complexes in the sampled solutions. Nevertheless, differences in the behavior of related systems (probed under identical conditions) do reflect trends in their tendency toward aggregation [9, 25, 26]. Thus, the observation of the different signal intensities of the polynuclear zincates is consistent with a lower tendency of Br^−^ and I^−^ to adopt bridging binding modes than that of the harder Lewis bases Cl^−^ and, in particular, OPiv^−^. The different nature of the individual anions X^−^ also manifested itself in the extent to which they were depleted or enriched in the detected complexes. If all anions behaved in the same manner, the composition of the latter should correspond to the overall stoichiometry of the (R^Ph*z*^)ZnX·LiX·LiCl sample solutions and show a 2:1 ratio between X^−^ and Cl^−^. In contrast, the observed ratio was markedly lower for X^−^ = OPiv^−^ (e.g., Figure 1a) and higher for X^−^ = Br^−^ and I^−^ (e.g., Figures S31 and S33). This behavior is consistent with the lower affinity of heavier, softer halides for Li^+^, which reduces ion pairing. As a result, a larger fraction of the bromide‐ and iodide‐containing complexes remains detectable by ESI‐mass spectrometry, whereas neutral ion‐paired species elude detection [16]. As a control experiment, we also probed a solution of (R^Ph3^)ZnX·LiX·LiCl, X = OAc, which afforded ESI‐mass spectra closely resembling those obtained for the corresponding pivalate‐containing system (Figures S35 and S36). A change of the silyl group, R^Ph*z,*^ did not result in any obvious effect. The addition of 1 equiv. of TMEDA did not significantly alter the distribution of zincate species either. In no case did we detect TMEDA‐containing complexes.

Complementary information was obtained from positive‐ion mode mass spectra (Figures 1b and S2, S4, S5, S6, S8, S10, S16, S18–S22, S24, S26, S28, S30, S32, and S34). For X = OPiv, extensive Li_*n* + 1_X_*n*_^+^ aggregates (1 ≤ *n* ≤ 8, Figures 1a, S2, S16, and S28) were observed. Such species are known to form during the ESI process due to solvent evaporation, leading to increased effective analyte concentrations [27]. The exclusive formation of these aggregates for pivalate‐containing systems, but not for halides, once more points to a higher affinity of OPiv^−^ for Li^+^ [9]. In contrast, halide‐containing systems exhibited less pronounced aggregation, consistent with a reduced Li^+^ affinity. Accordingly, the pivalate‐free sample solutions afforded less characteristic positive‐ion mode ESI‐mass spectra and rendered the identification of the detected ions more difficult. The species observed most consistently were the lithium–silanol adducts Li(HOR^Ph*z*^)*n* ^+^, *n* = 1 and 2 (Figures S18–S22, S24, S26, S30, S32, and S34), whose inferred identity was supported by gas‐phase fragmentation experiments (Figures S19 and S20) as well as by the analysis of the isotope patterns of representative examples (Figures S21 and S22) and whose likely origin will be discussed below. The observation of these ions emphasizes the high affinity of lithium toward oxygen‐based ligands. In the absence of TMEDA, the positive‐ion mode spectra showed almost no zinc‐containing ions of significant intensity. Upon addition of TMEDA (X = OPiv), strong signals corresponding to (R^Ph3^)Zn(TMEDA)^+^ were observed (Figure S28), indicating the stabilization of silylzinc cations by TMEDA coordination. Additional adducts with one or two LiOPiv units, as well as LiZn(OPiv)_2_(TMEDA)^+^ were also detected.

### 2.2 Neutral Silylzinc Species

^1^H‐NMR spectroscopy complements ESI‐mass spectrometry by probing both ionic and neutral components of the investigated solutions, thereby providing information on the overall speciation. Due to the lower sensitivity of the former, we increased the sample concentration relative to that of the ESI experiments (*c* = 0.16 vs. 0.038 m). The spectra of (R^Ph*z*^)ZnX·LiX·LiCl solutions showed multiple signals in the aromatic region (Figure 1c as a representative example), thus indicating the presence of several species in dynamic equilibrium, in agreement with the complex speciation revealed by ESI‐mass spectrometry. Due to the relatively low time resolution of NMR spectroscopy, the recorded signals likely represent averaged responses of rapidly interconverting species, thus precluding detailed structural assignments in solution.

Additional structural insight was provided by X‐ray diffraction analysis of single crystals of (Ph_3_Si)ZnX(TMEDA) (X = Cl and I), which were obtained from the corresponding solutions by removal of THF under reduced pressure, washing the residual solid material with pentane and redissolving it in Et_2_O, followed by the addition of pentane and cooling to 250 K (see Supporting Information for further details). The complexes featured a direct Zn─Si bond and a distorted tetrahedral coordination geometry of the zinc center (Figures 1d, S37, and S38) [28]. These results provide a structural reference for interpreting the solution‐phase speciation of silylzinc reagents and highlight the Lewis acidity of the zinc center as well as its propensity for ligand coordination, which underlies the observed TMEDA stabilization.

### 2.3 Degradation Reactions

The stability of silylzinc reagents is governed by the susceptibility of the Zn─Si bond toward cleavage, which can possibly occur via protodemetalation or oxidation pathways. To probe and characterize these processes, solutions of (R^Ph*z*^)ZnX·LiX·LiCl were exposed to the ambient atmosphere and analyzed over time by ESI‐mass spectrometry (Figures S39−S64) and ^1^H‐NMR spectroscopy (Figures S65−S103).

**FIGURE 2:**
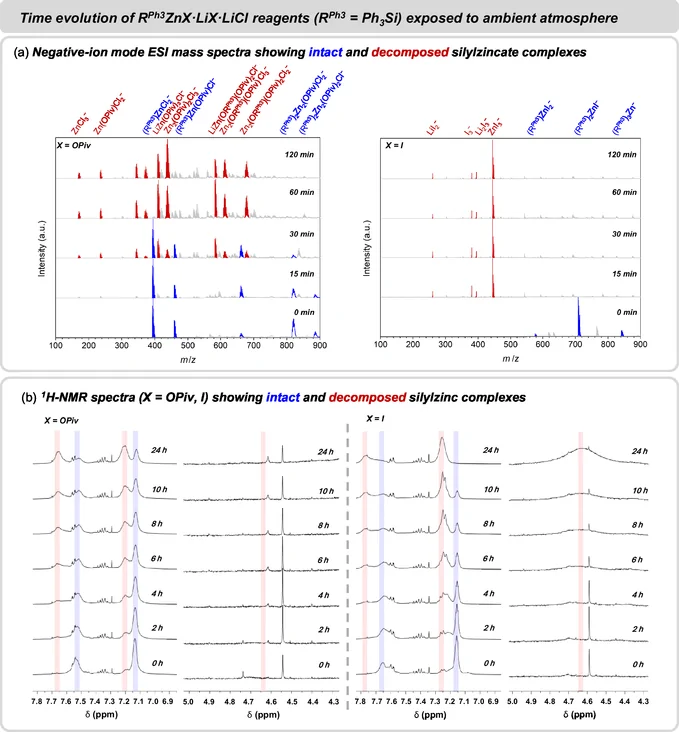
Time evolution of R^Ph3^ZnX·LiX·LiCl reagents (X = OPiv, I) exposed to ambient atmosphere. (a) Negative ion‐mode ESI‐mass spectra for X = OPiv (left) and I (right). (b) ^1^H‐NMR spectra for X = OPiv (left) and I (right) showing the aromatic region (6.9–7.8 ppm) and the SiOH region (4.3–5.0 ppm). Notably, no broad silanol signal is observed for the pivalate‐containing system in this region.

Negative‐ion mode ESI‐mass spectrometric analysis of (R^Ph*z*^)ZnX·LiX·LiCl solutions revealed a gradual transformation of intact silylzincate species into simpler inorganic zinc‐containing ions Li_*m*_Zn_*n*_X_*m* + 2*n* + 1_‒, indicating the occurrence of decomposition processes, presumably protodemetalation reactions induced by traces of moisture. For a closer analysis, we focus on the ESI‐mass spectra recorded for (R^Ph3^)ZnX·LiX·LiCl solutions of higher concentrations, i.e., *c* = 0.16 m (Figures S55−S64), which displayed more robust trends than those measured for lower concentrations, i.e., *c* = 0.038 m. These high‐concentration ESI‐mass spectra revealed a strong dependence of the decomposition rate on the nature of the anion X^−^. While halide‐containing systems (X = Cl, Br, and I) showed complete loss of intact silylzincate species within 15 min, pivalate‐containing analogs retained significant signal intensity even after 30 min, indicating a markedly enhanced stability (Figures 2a and S55, S57, S59, and S61). To test whether the enhanced lifetime of the intact silylzincates is specific to the pivalate system or forms a general feature of carboxylate‐containing reagents, we performed control experiments with (R^Ph3^)ZnX·LiX·LiCl, where X = OAc (Figure S63). We observed the appearance of decomposition products after 20 min, which is earlier than for the analogous pivalate‐containing system but later than for the halide‐containing ones. This observation indicates that although carboxylates in general slow down the decomposition of silylzincates, pivalate exerts an additional stability‐enhancing effect.

In addition to protodemetalation, oxidation processes were observed. The formation of siloxide‐containing species, such as Zn(OR^Ph*z*^)X_2_^−^ and related complexes detected by negative‐ion mode ESI‐mass spectrometry (Figures 2a and S55, S57, S59, and S61), suggests the insertion of molecular oxygen into the Zn─Si bond followed by O─O bond cleavage. Analogous processes are known for alkylzinc reagents [29, 30]. Our assignment is supported by the detection of Li(HOR^Ph3^)^+^ adducts in the positive‐ion mode mass spectra (Figures S58, S60, and S62) and by ^1^H‐NMR‐spectroscopic evidence (see below) [31, 32]. The time scale of oxidation mirrors that of protodemetalation, with halide‐containing systems undergoing rapid oxidation, whereas pivalate‐containing systems exhibit significantly delayed formation of oxidized species.

Further support for the occurrence of oxidation processes was obtained from ^1^H‐NMR spectroscopy. Upon sample exposure to air, broad signals around *δ* ≈ 4.5 ppm appeared (Figures S68, S71, S74, S80, S83, S86, S92, S95, and S98), which are characteristic of silanol OH groups [31, 33, 34, 35, 36]. Control experiments employing authentic HOR^Ph3^ confirmed this assignment (Figures S104‍−‍S107). We attribute the formation of silanols to the oxidation of the Zn─Si bond, followed by protonation of the resulting siloxide species by trace amounts of water from air. The silanol OH signals were absent for pivalate‐based reagents under identical conditions (Figures S65, S77, and S89), indicating that pivalate coordination effectively suppresses silanol formation. Likewise, these signals were also missing for the acetate‐containing system (Figure S101). Presumably, the more protophilic carboxylates not only enhance the stability of the silylzinc reagents against oxidation, but also partly deprotonate the formed silanols and, thus, render their detection by ^1^H‐NMR spectroscopy more difficult. Note that the time scale of the appearance of the silanol signals (few hours) was comparatively long due to the slower intake of O_2_/H_2_O into the NMR tubes than into the flasks containing the sample solutions probed by ESI‐mass spectrometry.

A comparison of reagents with different silyl groups indicates a reduced tendency toward silanol formation for sterically demanding R^Ph*z*^ residues, suggesting that steric protection of the Zn─Si bond slows down both protodemetalation and oxidation pathways (Figures S68, S80, and S92). Steric protection also explains why pivalate appears to be more effective than acetate in enhancing the stability of the silylzinc reagents toward degradation.

^1^H‐NMR spectroscopy also revealed significant quantities of silanes, including Me_2_(Ph)SiH, which was easily identified by its characteristic septet at *δ* = 4.4 ppm [37] (Figures S65−S76). These species are of possible interest because they correspond to the products of protodemetalation. However, these signals were already present at very short reaction times, suggesting that they were, at least in part, derived from the R^Ph*z*^Li reagents employed for the synthesis of the silylzinc compounds. For this reason, NMR spectroscopy could not be used for monitoring protodemetalation reactions during the exposure of the (R^Phz^)ZnX·LiX·LiCl reagents to the ambient atmosphere. Taken together, the present results establish that the degradation behavior of silylzinc reagents is controlled by a combination of anion coordination, ion pairing interactions (modulated by Li^+^), and steric effects. In particular, pivalate ligands significantly enhance stability, likely through the observed stronger Li^+^ interactions and increased aggregation. The increased aggregation presumably stabilizes the silylzinc moieties energetically. It also reduces the accessibility of the Zn─Si bond, thereby suppressing both protodemetalation and oxidation processes. A similar effect has previously been reported for organocuprates [38]. In addition, the higher protophilicity of pivalate and other carboxylates, compared to that of halides, supposedly results in the scavenging of protons, thus also helping in preventing protodemetalation reactions.

### 2.4 Comparison With Related Systems

Previous studies have established that organozinc reagents commonly form zincate species in solution and undergo aggregation processes governed by Schlenk‐type equilibria [7, 8, 9]. The present results indicate that silylzinc reagents exhibit closely related speciation behavior, including the formation of mono‐ and polynuclear zincate complexes. Similarly, the structures of the neutral compounds Ph_3_SiZnCl(TMEDA) and Ph_3_SiZnI(TMEDA) as well as those of their previously reported Me_3_SiZnI(TMEDA) and Me_2_(Ph)SiZnI(TMEDA) [28] congeners resemble those of related organozinc complexes [39].

In terms of reactivity, the higher stability of pivalate‐containing silylzinc reagents toward degradation seems to mirror the behavior of their organozinc homologs. Despite these apparent similarities, these classes of reagents differ in the molecular mechanisms by which they bring about the stabilization of the silyl‐ and organylzinc moieties, respectively. As Hernán‐Gómez et al. have shown [6], the superior stability of the organozinc pivalates toward moisture results from the presence of Mg(OPiv)_2_ motifs, which remain separate from the organozinc component and protect the latter by scavenging H_2_O and other proton donors. In contrast, the present silylzinc pivalates do not contain any magnesium. While the pivalate anions of the silylzinc reagents can also deactivate H_2_O molecules by deprotonation, they at the same time bridge zinc and lithium centers and, thus, assist in the formation of heteronuclear aggregates. As explained above, the incorporation of the silylzinc moiety into these aggregates results in energetic stabilization and hinders the approach of O_2_ and H_2_O molecules.

## 3 Conclusion

The present findings demonstrate that anion identity plays a decisive role in controlling both the speciation and stability of silylzinc reagents. In particular, the pivalate counter‐ion significantly enhances resistance toward oxidation and protodemetalation. We find pivalate to exert its beneficial effect in a threefold manner: (i) By forming strong interactions with Li^+^, pivalate favors the formation of heteronuclear aggregates more than halides. These aggregates energetically stabilize the silylzinc moiety and also block the access of O_2_ and H_2_O to the latter. (ii) The high steric demands of the pivalate counter‐ion further help in shielding the silylzinc bond against attacks by O_2_ and H_2_O. (iii) Being more protophilic than the halides, pivalate more effectively suppresses protodemetalation reactions by proton scavenging. These stabilization mechanisms are distinct from those previously elucidated for related organozinc pivalate reagents. The insight thus gained into the decomposition pathways of silylzinc compounds and the critical role of anion control will be instrumental in the design and development of robust organosilicon nucleophiles.

## Funding

This study was supported by the China Scholarship Council, Deutsche Forschungsgemeinschaft (INST 186/1326‐1 FUGG), Niedersächsisches Ministerium für Wissenschaft und Kultur, and the Open Access Publication Funds of Universität Göttingen.

## Conflicts of Interest

The authors declare no conflicts of interest.

## Supporting information

Supplementary Material

## Data Availability

Data is provided in the Supporting Information.
