# Supplementary material for: Anion‐Controlled Speciation and Stability of Silylzinc Reagents
Source: ChemistryOpen. 2026 Sep 15;15(9):e70301. doi: 10.1002/open.70301 (PMC13576932; doi:10.1002/open.70301)
Supplement: Supplementary file 1 [file OPEN-15-e70301-s001.pdf]

Anion-Controlled Speciation and Stability of Silylzinc Reagents

Zhili Duan† and Konrad Koszinowski†*

†Institut für Organische und Biomolekulare Chemie, Universität Göttingen, Tammannstraße 2,

37077 Göttingen, Germany
*Email: konrad.koszinowski@chemie.uni-goettingen.de

Supporting Information

Table of Contents

1. Experimental details ······ S1
  1.1 Materials and general methods ······ S1
  1.2 Preparation of trisorganylsilyllithium reagents ······ S1
  1.3 General preparation of trisorganylsilylzinc sample solutions ······ S1
  1.4 ESI-mass spectrometry ······ S2
  1.5 NMR spectroscopy ······ S3
  1.6 Crystallization procedure ······ S3
2. ESI-mass spectrometric characterization of freshly prepared silylzinc reagents ······ S4
  2.1 (R^{Ph})ZnX (X = OPiv, Cl, Br, I) systems ······ S4
  2.2 (R^{Ph2})ZnX (X = OPiv, Cl, Br, I) systems ······ S8
  2.3 (R^{Ph3})ZnX (X = OPiv, Cl, Br, I, OAc) systems ······ S14
3. X-ray crystallographic characterization ······ S18
4. Time-dependent ESI-mass spectrometric experiments under ambient atmosphere ······ S19
  4.1 (R^{Ph})ZnX·LiX·LiCl (X = OPiv, Cl, Br, I) systems ······ S19
  4.2 (R^{Ph2})ZnX·LiX·LiCl (X = OPiv, Cl, Br, I) systems ······ S23
  4.3 (R^{Ph3})ZnX·LiX·LiCl (X = OPiv, Cl, Br, I, OAc) systems ······ S27
5. Time-dependent ^{1}H-NMR experiments under ambient atmosphere ······ S32
  5.1 (R^{Ph})ZnX·LiX·LiCl (X = OPiv, Cl, Br, I) systems ······ S32
  5.2 (R^{Ph2})ZnX·LiX·LiCl (X = OPiv, Cl, Br, I) systems ······ S38
  5.3 (R^{Ph3})ZnX·LiX·LiCl (X = OPiv, Cl, Br, I, OAc) systems ······ S44
6. NMR studies supporting the assignment of silanol chemical shifts ······ S52
7. References ······ S55

1. Experimental details

Throughout this Supporting Information, R^{Ph}, R^{Ph2}, and R^{Ph3} denote Me_2PhSi, $MePh_2Si$, and Ph_3Si, respectively.

1.1 Materials and general methods

In all cases, standard Schlenk techniques were applied. Tetrahydrofuran (THF) was dried over sodium/benzophenone and freshly distilled before use. Diethyl ether (Et_2O) and pentane were purified using an MBraun SPS-800 solvent purification system and stored over fresh sodium chips and activated molecular sieves under an inert atmosphere. THF-d_8 was degassed by three freeze–pump–thaw cycles and stored under an inert atmosphere prior to use. $ZnCl_2$ was dried by prolonged heating under vacuum. $Zn(OPiv)_2$ was synthesized according to previously described methods.[21] All other chemicals were obtained from commercial sources and used as received (Table S1).

Table S1. Sources of the chemicals used in this study.

Compound	Purity/Concentration	Manufacturer
Pivalic acid	99%	Merck
Diethylzinc	1.0 M in heptane	Sigma-Aldrich
Lithium	≥ 99.0%	Merck
Dimethylphenylsilyl chloride	97%	abcr GmbH
Diphenylmethylsilyl chloride	97%	abcr GmbH
Triphenylsilyl chloride	96%	Sigma-Aldrich
2,2-Diphenylacetic acid	99.93%	BLD PHARMATECH GmbH
$ZnCl_2$	≥ 98.0%	Alfa Aesar
$ZnBr_2$	99.99%	abcr
ZnI_2	99.99%	Acros Organics
MeOH	≥ 99.9%	Fisher Scientific
THF	≥ 99.8%	Sigma-Aldrich
THF-d_8	99.5% D	Eurisotop
TMEDA (tetramethylethylenediamine)	99%	Merck
Et_2O	99%	Merck

1.2 Preparation of trisorganylsilyllithium reagents

The following procedures were adopted from the literature.[21]

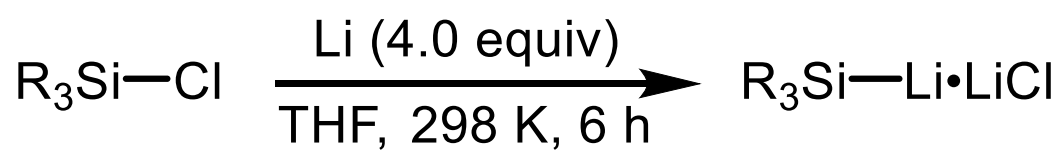

Scheme S1. Preparation of trisorganylsilylithium reagents

A 10-mL two-necked round-bottom flask equipped with a magnetic stirring bar was charged with lithium clippings (84 mg, 12 mmol) and THF (3.0 mL). Then the trisorganylsilyl chloride (3.0 mmol, 1.0 equiv) was added dropwise at 273 K and the mixture was stirred at the same temperature for 6 h. The resulting trisorganylsilyllithium reagent was titrated against 2,2-diphenylacetic acid according to Kofron's method.[40]

1.3 General preparation of trisorganylsilylzinc sample solutions (Scheme S2)

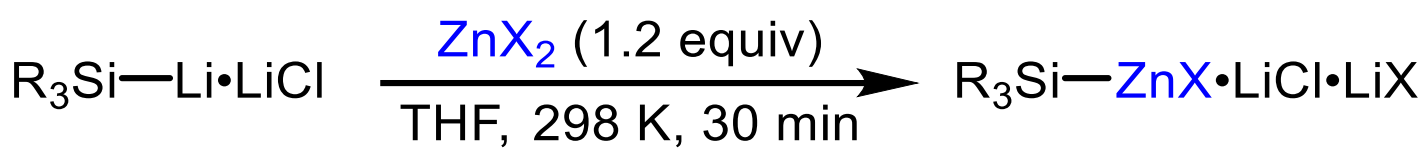

Scheme S2. Preparation of trisorganylsilylzinc reagents

ZnX_2 (X = OPiv, Cl, Br, I, OAc, 0.20 mmol) was quickly added into an oven-dried 10-mL Schlenk tube. After evacuation, the tube was refilled with argon, and this evacuation-refill cycle was repeated three times before the addition of THF to afford an approx. 1.0-M solution. Next, the trisorganylsilyllithium reagent (1.0 equiv) was added under stirring. The stirring was continued for 30 min before the resulting solution of the trisorganylsilylzinc reagent was diluted to a concentration of 0.038 M prior to ESI-mass spectrometric analysis.

1.4 ESI-mass spectrometry

The following conditions and settings were used for the amaZon speed instrument:

ESI source:

ESI voltage:	±4500 V
Nebulizer gas:	N_2 (0.7 bar)
Drying gas:	N_2 (5 L min^{-1}, 333 K)

Ion transfer and ion trapping:

Capillary exit:	±190 V
Funnel 1:	In −100, Out −35, Lens −25
Funnel 2:	In −12, Out −10, Lens −3.3
Octupole:	1PC −2.3, 2PC −1.1
Focus:	L1 −0.5, L2 20, L3 6
Multipole DC: 4.0	
Focus:	L2 5, L3 75
Trap drive:	41.6

Gas-phase fragmentation:

Isolation width:	3u
RF level:	45%
Low-mass cut-off:	50 *m*/*z*

Sample solutions were administered into the ESI source of the mass spectrometer via gas-tight syringes or directly from a cooled reaction flask via the pressurized-sample infusion method developed by McIndoe and coworkers.[41] In the former case, the temperature of the sample solutions could not be rigorously controlled. The spectrometer was externally calibrated with a mixture of betaine, ammonium trifluoroacetate, 2,4,6-tris(heptafluoropropyl)-1,3,5-triazine, and phosphazenes in H_2O/MeCN. Exact *m*/*z* ratios and theoretical isotope patterns were calculated with the Compass software package (Bruker Daltonik).

For the time-dependent ESI-mass spectrometric experiments under ambient atmosphere, the reaction mixtures were exposed to laboratory air directly after completion of the reaction. For experiments involving short exposure times, aliquots were withdrawn after defined time intervals, diluted with THF to a concentration of 0.038 M, transferred into gas-tight syringes, and directly subjected to ESI-mass spectrometric analysis. In selected long-term exposure experiments, aliquots (0.16 M) were directly

transferred from the reaction mixtures into gas-tight syringes and analyzed without prior dilution. For each reported mass spectrum, the concentration of the analyzed sample solution is given.

1.5 NMR spectroscopy

All NMR samples were prepared under inert atmosphere using standard Schlenk techniques. Solutions of the corresponding silylzinc reagents were prepared in THF-d_8 with a concentration of approximately 0.16 M. After preparation, 0.5 mL aliquots were transferred under inert atmosphere into standard NMR tubes. The initial spectrum (defined as 0 h) was recorded immediately after sample preparation. Subsequently, the NMR tube was opened to ambient atmosphere. Except during NMR measurements, the sample was kept in a fume hood throughout the experiment. ^{1}H-NMR spectra were recorded at 2, 4, 6, 8, 10, and 24 h after exposure. All measurements were performed at 298 K on a 400-MHz NMR spectrometer. No external moisture was intentionally added. Integrations are reported relative to the hexamethylbenzene internal standard and are considered approximate due to overlapping resonances from multiple species present in solution (e.g., OPiv-containing species).

1.6 Crystallization procedure and X-ray diffractometry

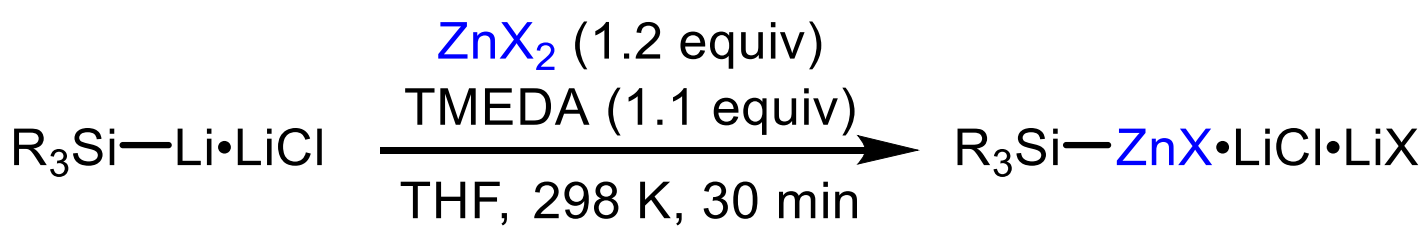

ZnX_2 (X = Cl, I, 0.2 mmol) was quickly added into an oven-dried 10-mL Schlenk tube. After evacuation, the tube was refilled with argon, and this evacuation-refill cycle was repeated three times before the addition of THF to afford an approx. 1.0-M solution. Next, the trisorganylsilyllithium reagent (1.0 equiv) was added under stirring. After stirring at room temperature for 30 min, TMEDA (1.1 equiv) was added to the reaction mixture.

The resulting reaction mixture was transferred into an N_2-filled glovebox and concentrated to dryness under reduced pressure. The resulting solid was dissolved in Et_2O and filtered. Removal of the solvent under reduced pressure afforded a colorless solid, which was washed with pentane three times. The purified solid was then redissolved in anhydrous Et_2O, and pentane was slowly added until the onset of precipitation. The flask was cooled to 250 K and left undisturbed for 24 h, during which time crystals of (R^{Ph3})ZnCl(TMEDA) and (R^{Ph3})ZnI(TMEDA), respectively, formed. Single-crystal X-ray diffraction data were collected on a Bruker D8 diffractometer equipped with a PHOTON detector and a microfocus X-ray source (Mo Kα radiation, λ = 0.71073 Å).

2. ESI-mass spectrometric characterization of freshly prepared silylzinc reagents

2.1 (R^{Ph})ZnX (X = OPiv, Cl, Br, I) systems

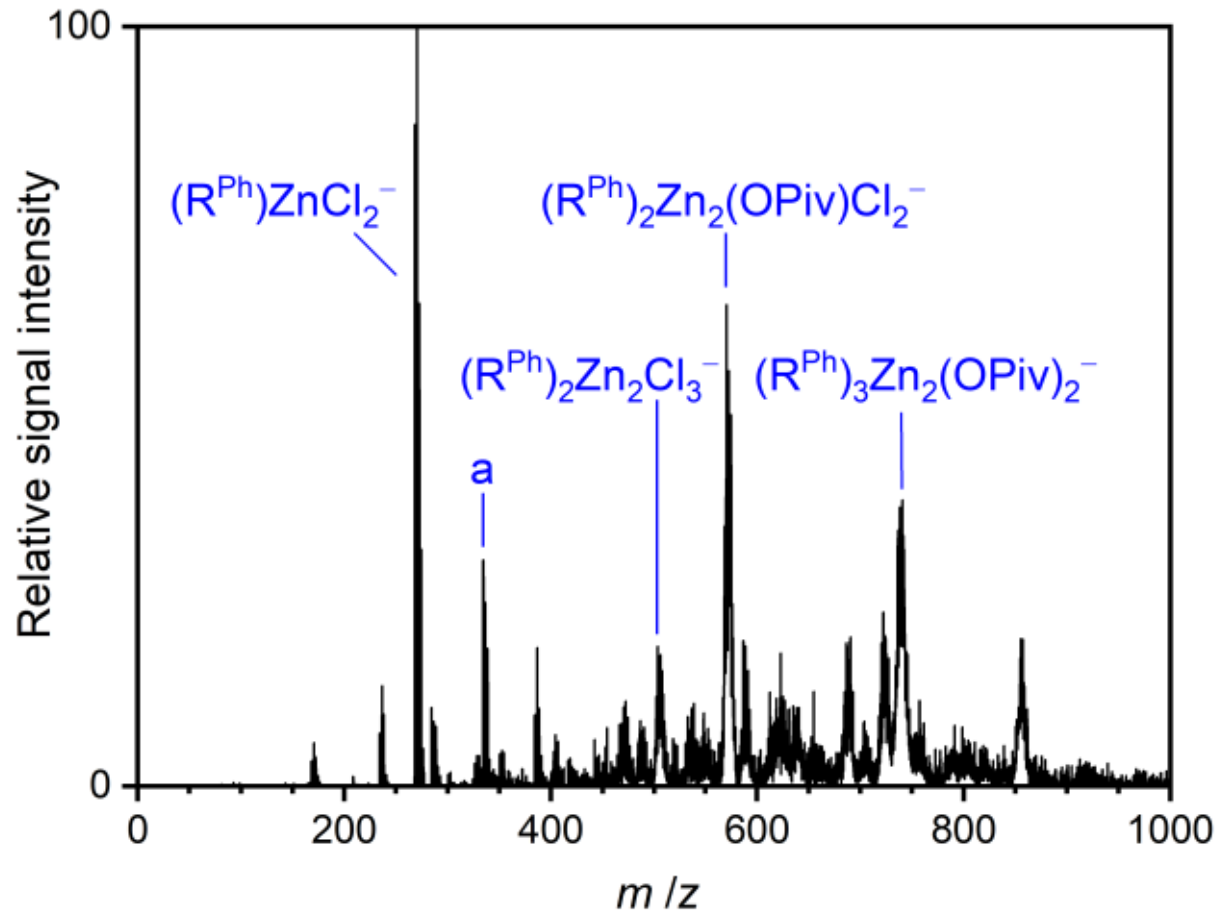

Figure S1. Negative-ion mode ESI-mass spectrum of a solution of (R^{Ph})Zn(OPiv)·Li(OPiv)·LiCl in THF (0.038 M), a = (R^{Ph})Zn(OPiv)Cl^-.

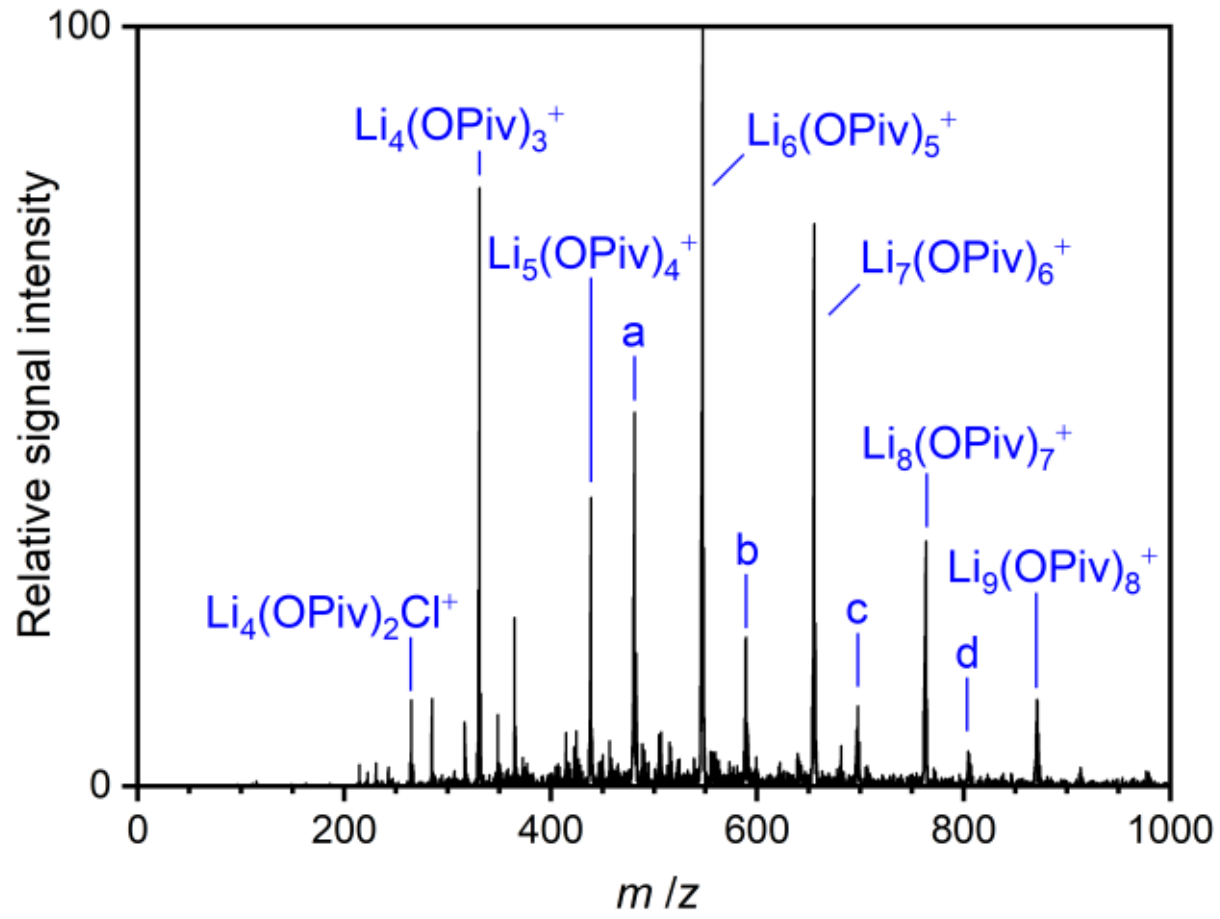

Figure S2. Positive-ion mode ESI-mass spectrum of a solution of (R^{Ph})Zn(OPiv)·Li(OPiv)·LiCl in THF (0.038 M), a = $Li_6(OPiv)_4Cl^+$, b = $Li_7(OPiv)_5Cl^+$, c = $Li_8(OPiv)_6Cl^+$, d = $Li_9(OPiv)_7Cl^+$.

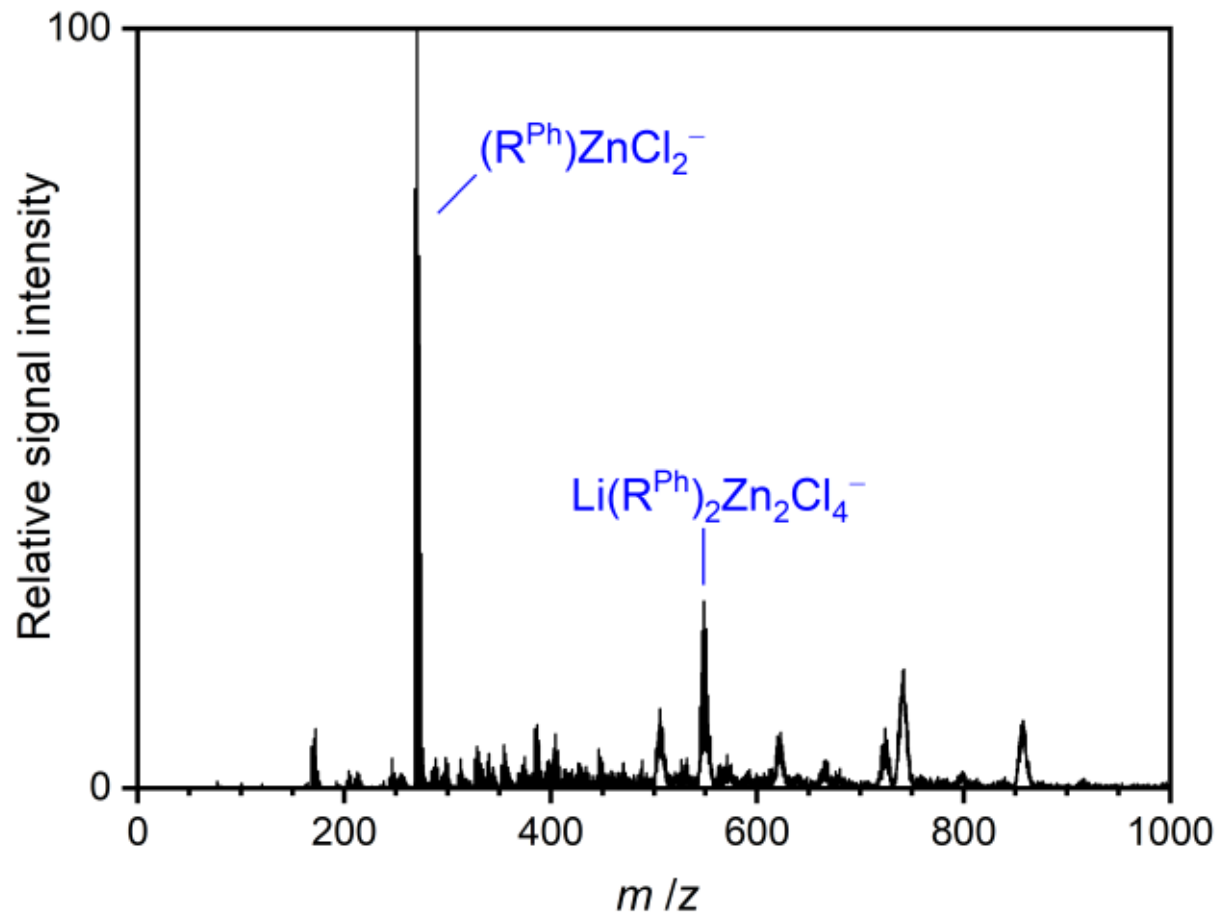

Figure S3. Negative-ion mode ESI-mass spectrum of a solution of (R^{Ph})ZnCl·2 LiCl in THF (0.038 M).

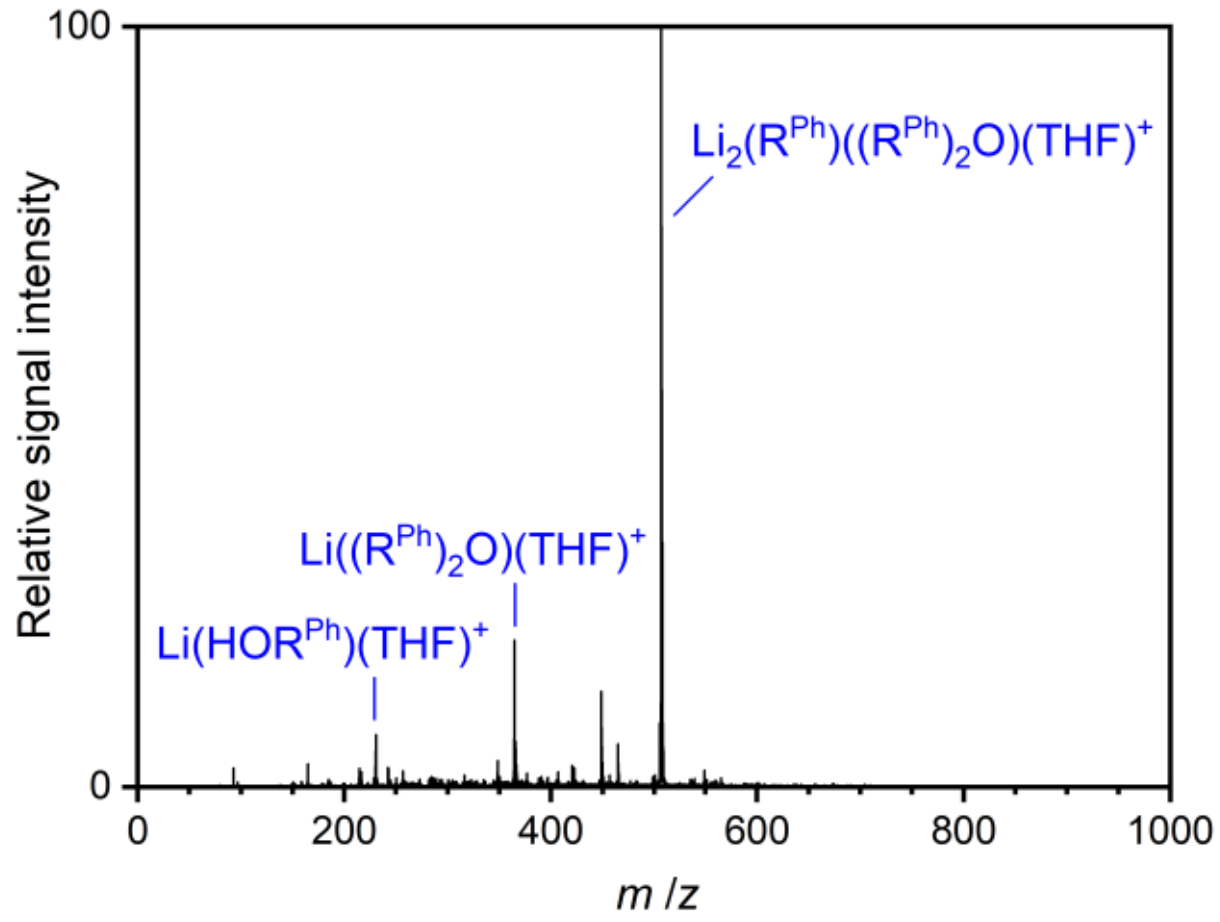

Figure S4. Positive-ion mode ESI-mass spectrum of a solution of $(R^{Ph})ZnCl{\cdot}2\,LiCl$ in THF (0.038 M).

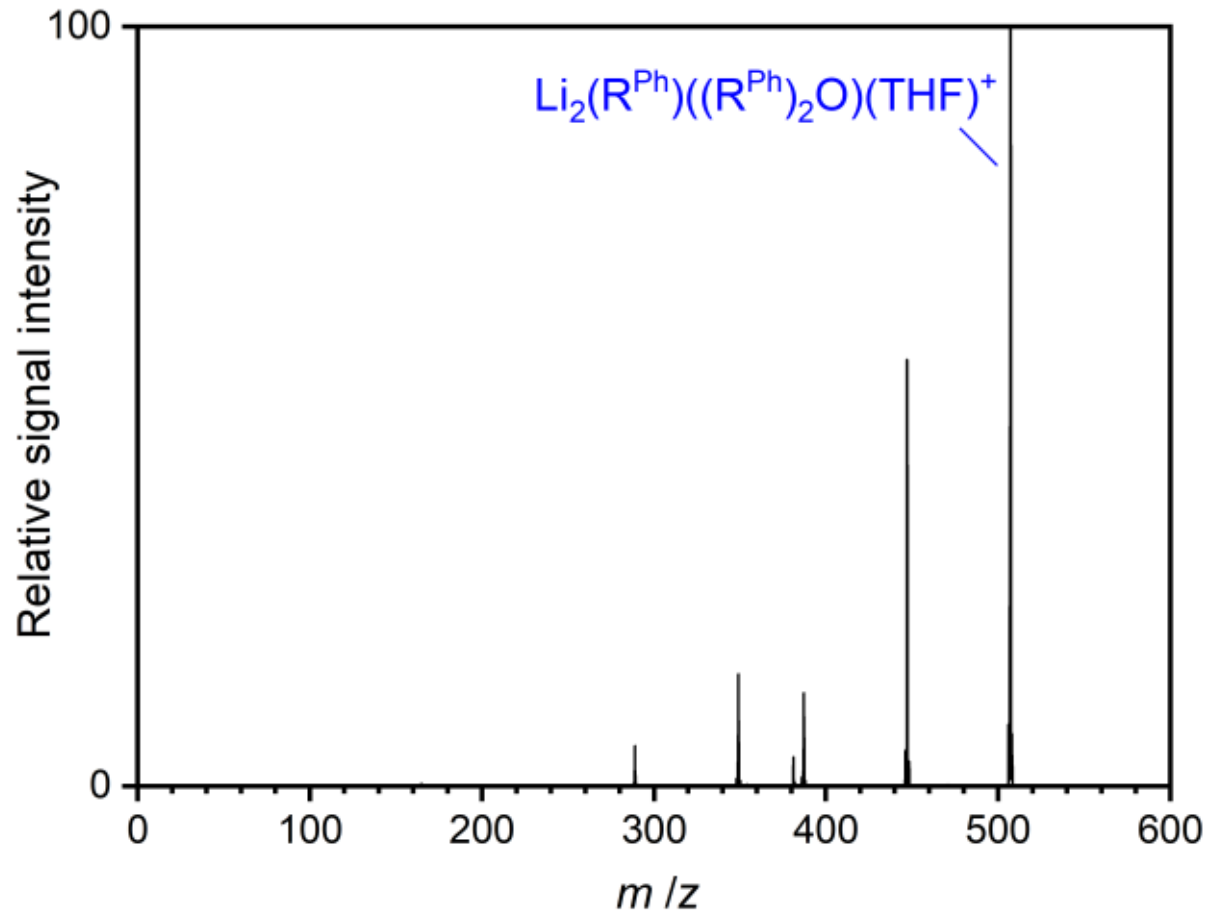

Figure S5. Mass spectrum of mass-selected $Li_2(R^{Ph})((R^{Ph})_2O)(THF)^+$ (*m/z* = 507) and its fragment ions formed upon collision-induced dissociation.

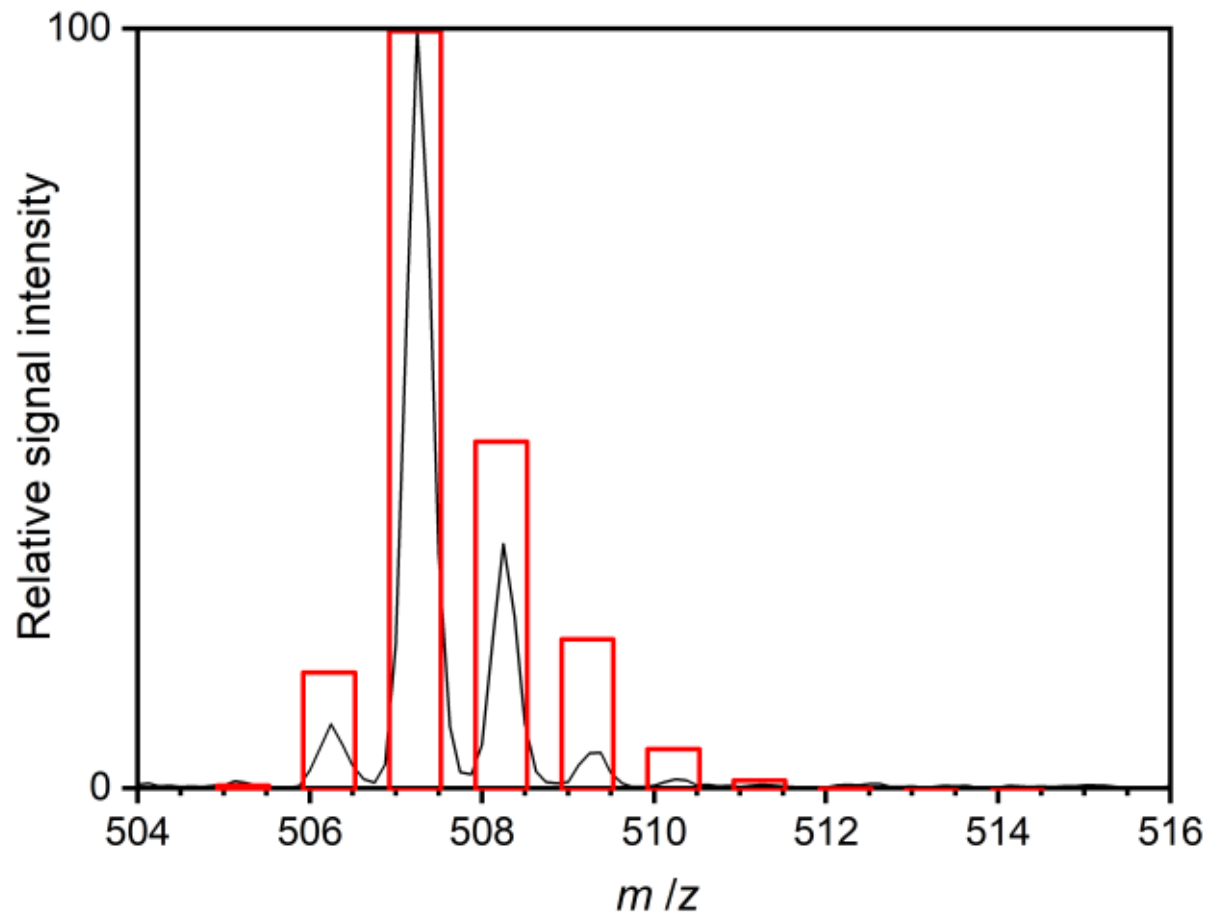

Figure S6. Measured (black) and simulated (red) isotope pattern of $Li(R^{Ph})((R^{Ph})_2O)(THF)^+$.

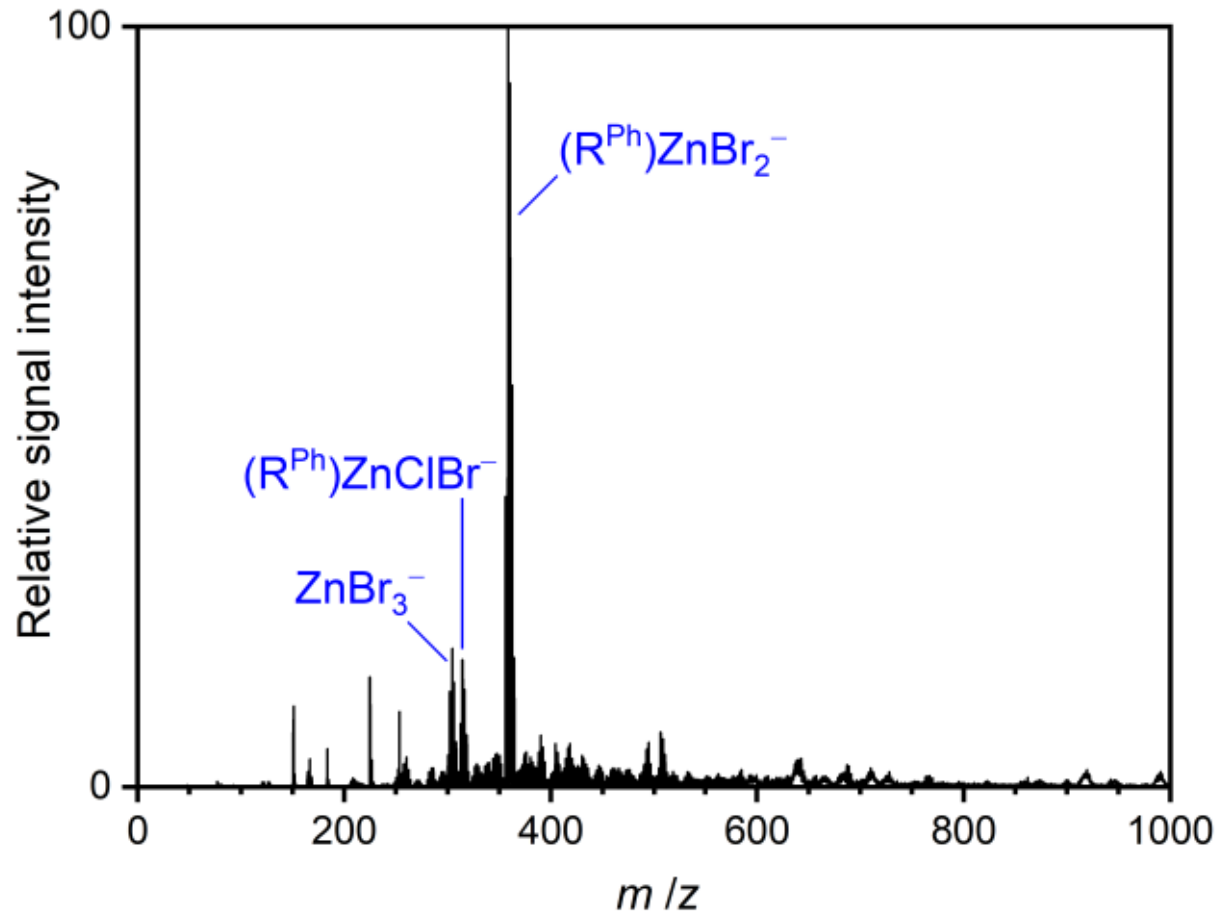

Figure S7. Negative-ion mode ESI-mass spectrum of a solution of (R^{Ph})ZnBr·LiCl·LiBrin THF (0.038 M).

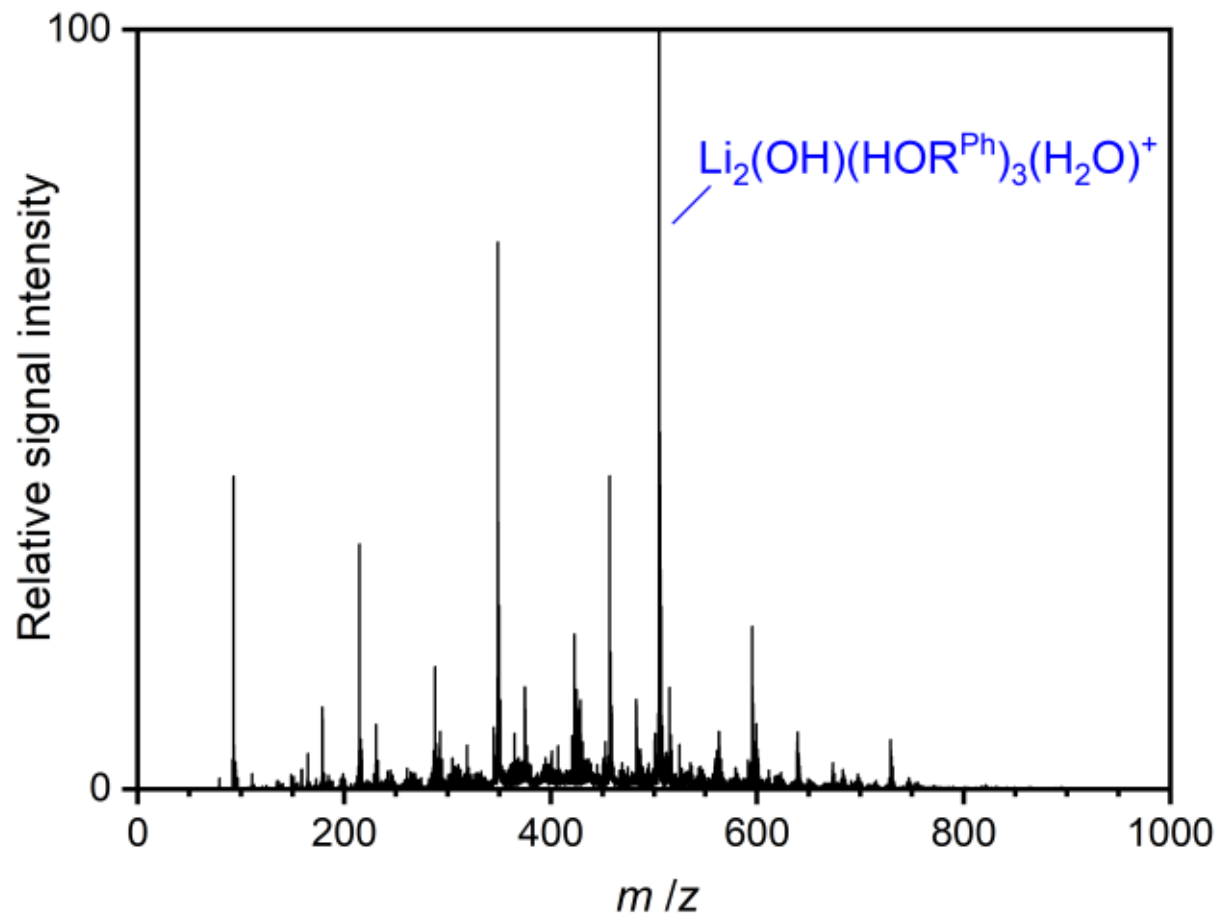

Figure S8. Positive-ion mode ESI-mass spectrum of a solution of (R^{Ph})ZnBr·LiCl·LiBr in THF (0.038 M).

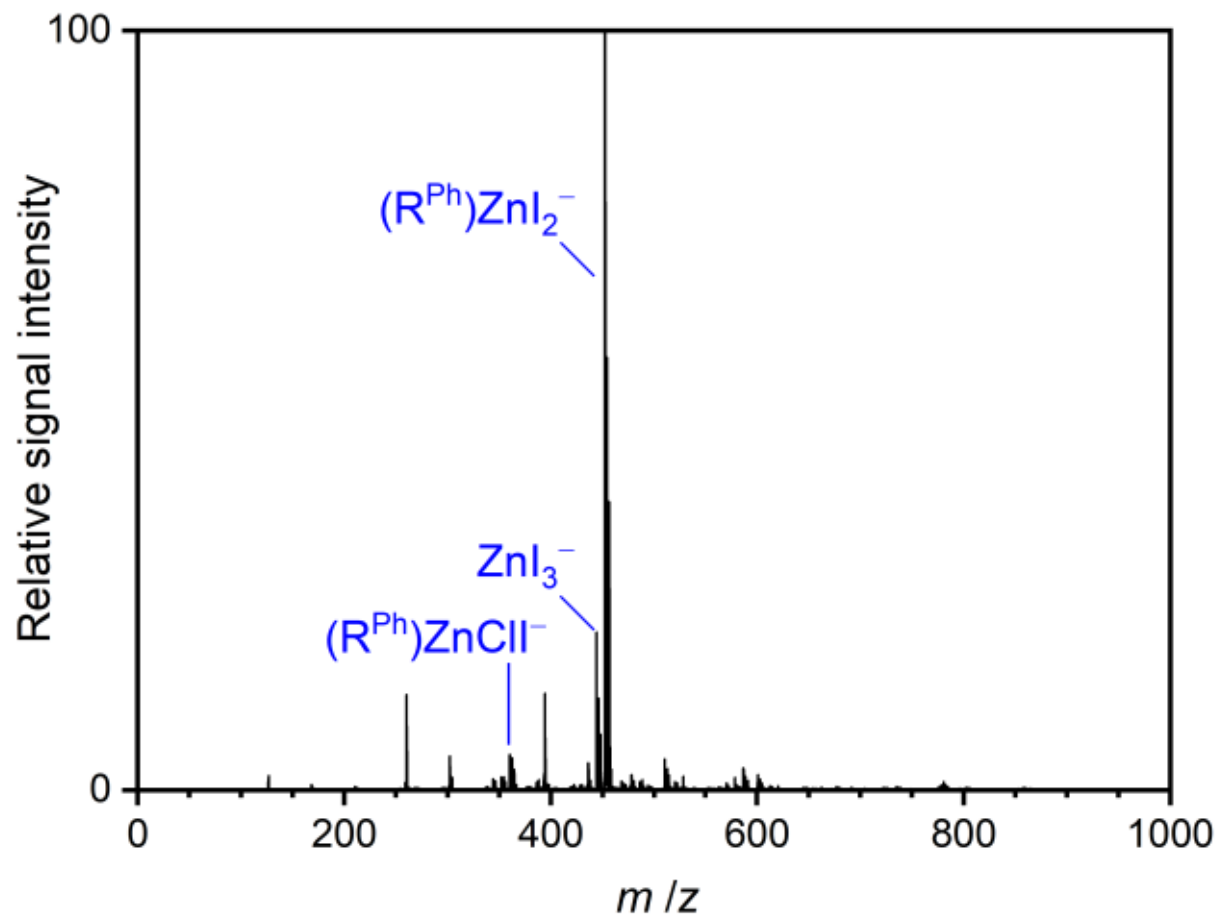

Figure S9. Negative-ion mode ESI-mass spectrum of a solution of (R^{Ph})ZnI·LiCl·LiI in THF (0.038 M).

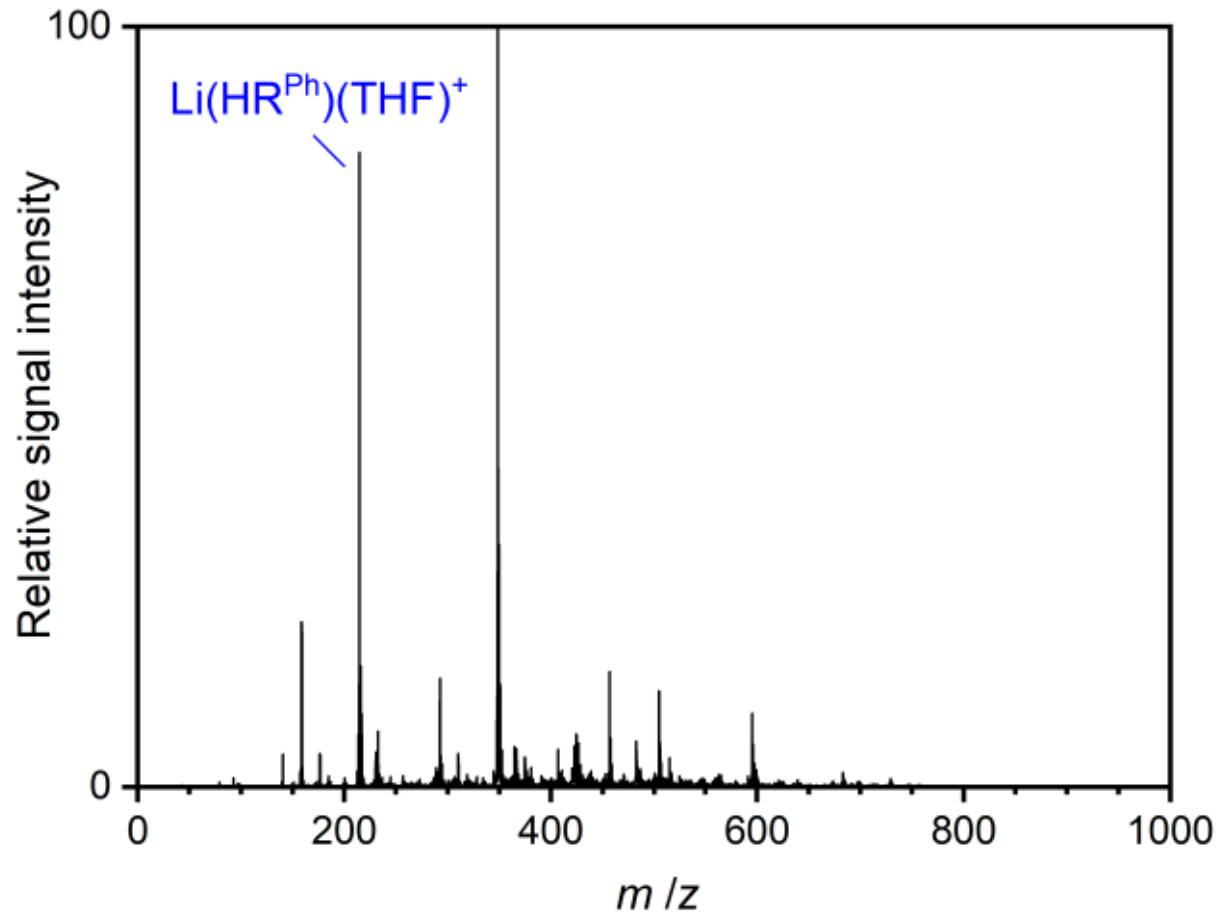

Figure S10. Positive-ion mode ESI-mass spectrum of a solution of (R^{Ph})ZnI·LiCl·LiI in THF (0.038 M).

2.2 (R^{Ph2})ZnX (X = OPiv, Cl, Br, I) systems

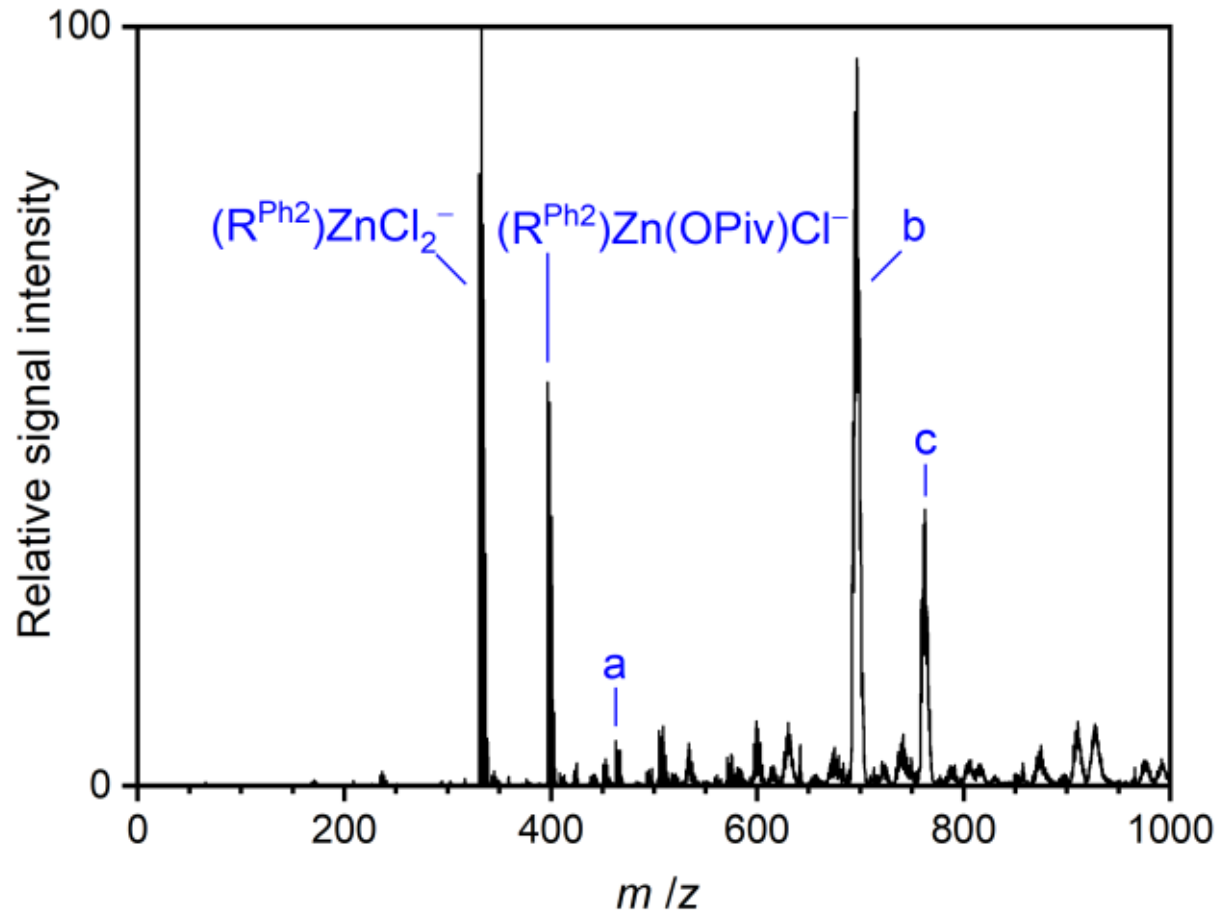

Figure S11. Negative-ion mode ESI-mass spectrum of a solution of (R^{Ph2})Zn(OPiv)·Li(OPiv)·LiCl in THF (0.038 M), a = (R^{Ph2})$Zn(OPiv)_2^-$, b = $(R^{Ph2})_2Zn_2(OPiv)Cl_2^-$, c = $(R^{Ph2})_2Zn_2(OPiv)_2Cl^-$.

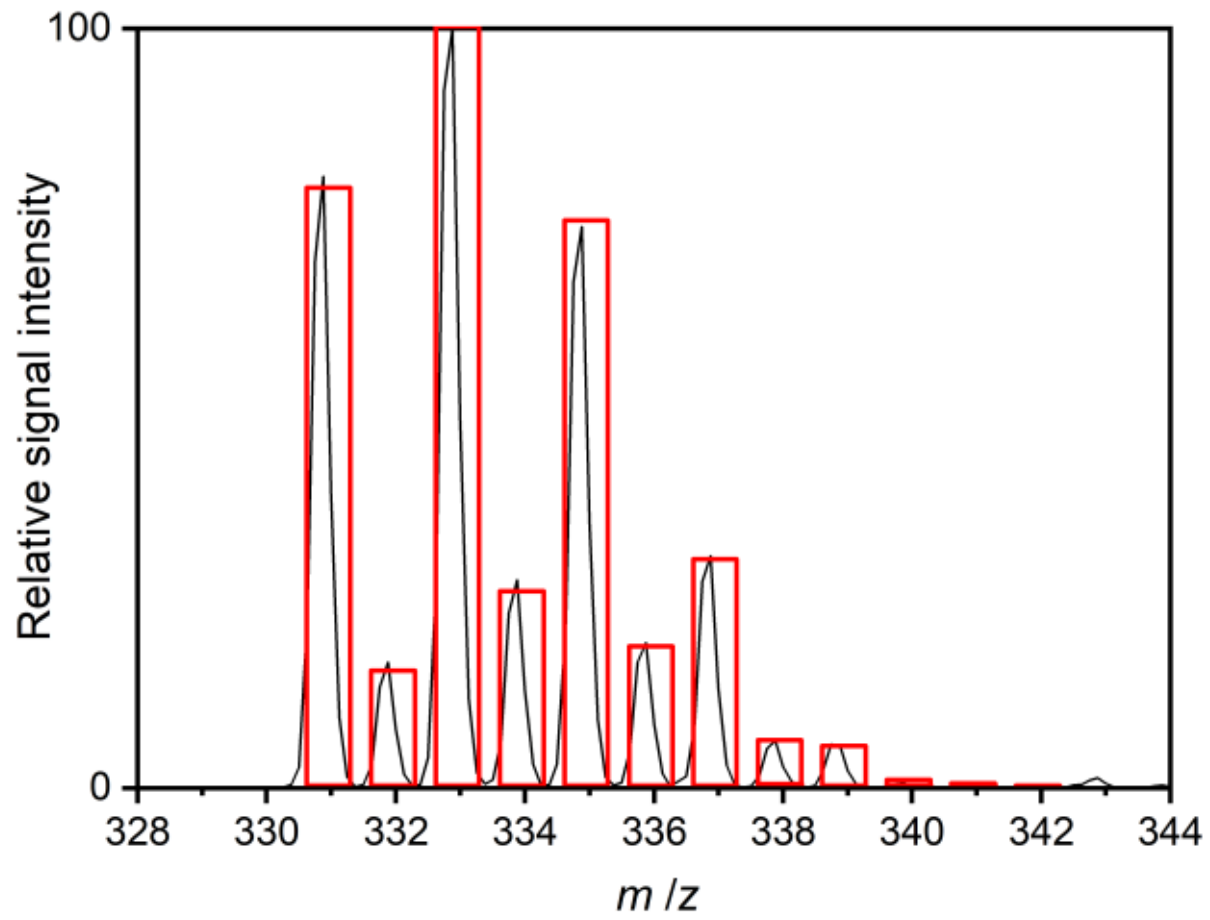

Figure S12. Measured (black) and simulated (red) isotope pattern of $R^{Ph2}ZnCl_2^-$.

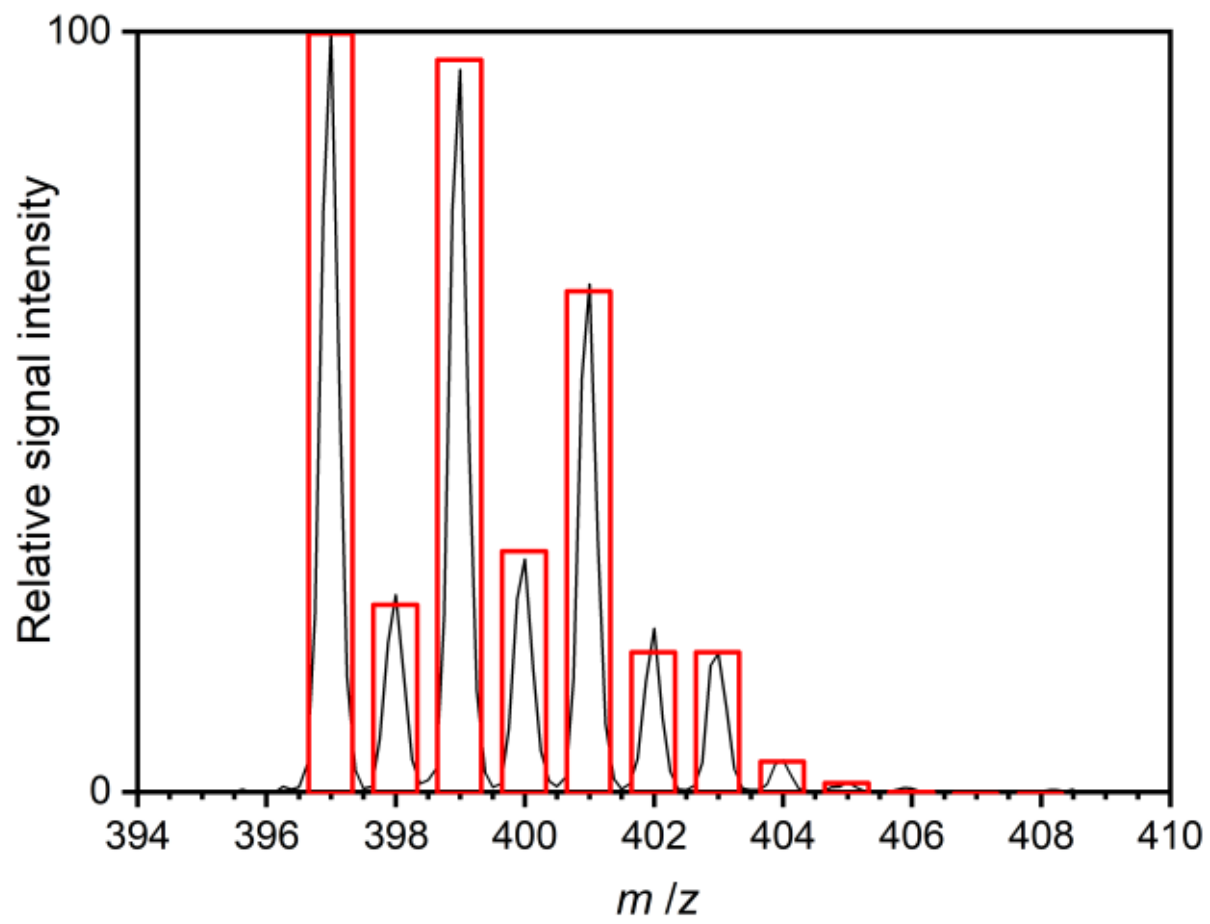

Figure S13. Measured (black) and simulated (red) isotope pattern of $R^{Ph2}Zn(OPiv)Cl^-$.

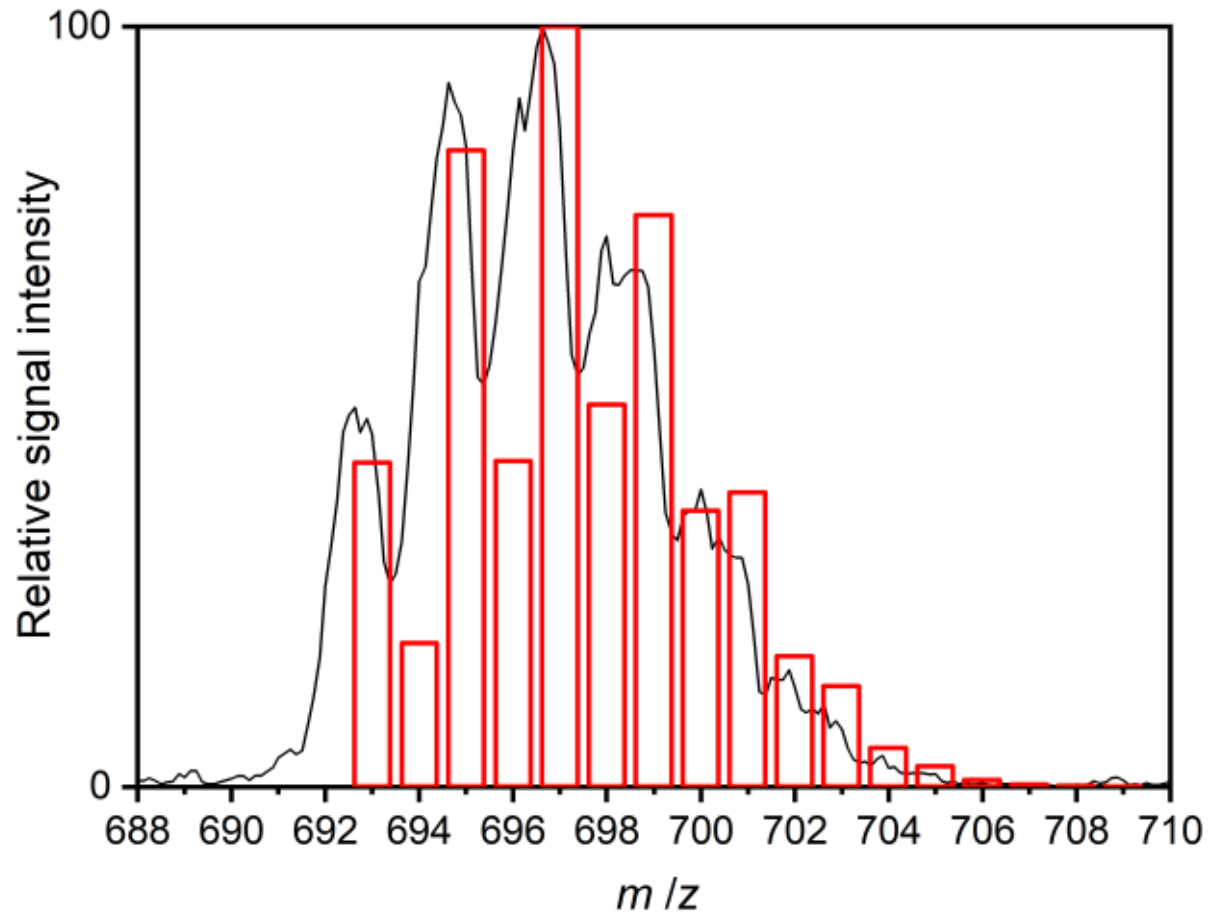

Figure S14. Measured (black) and simulated (red) isotope pattern of $(R^{Ph2})_2Zn_2(OPiv)Cl_2^-$.

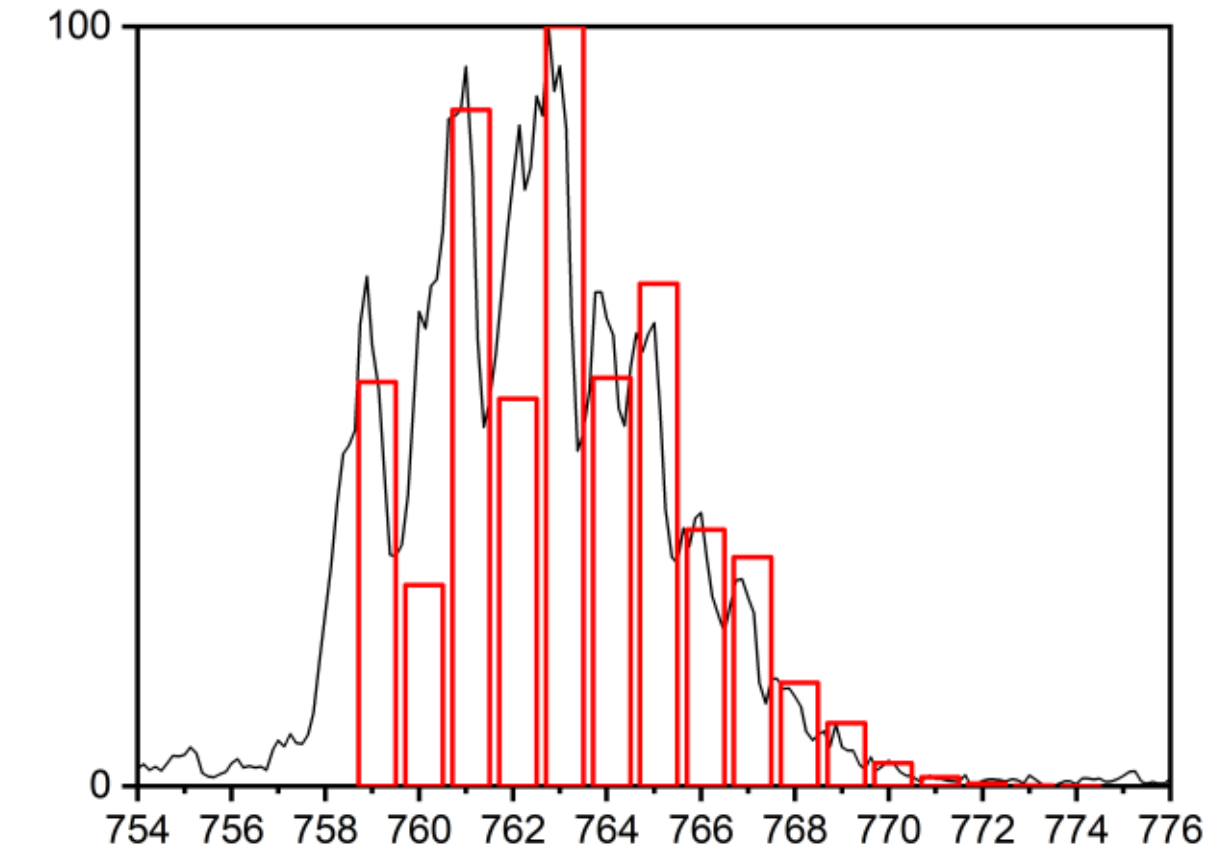

Figure S15. Measured (black) and simulated (red) isotope pattern of $(R^{Ph2})_2Zn_2(OPiv)_2Cl^-$.

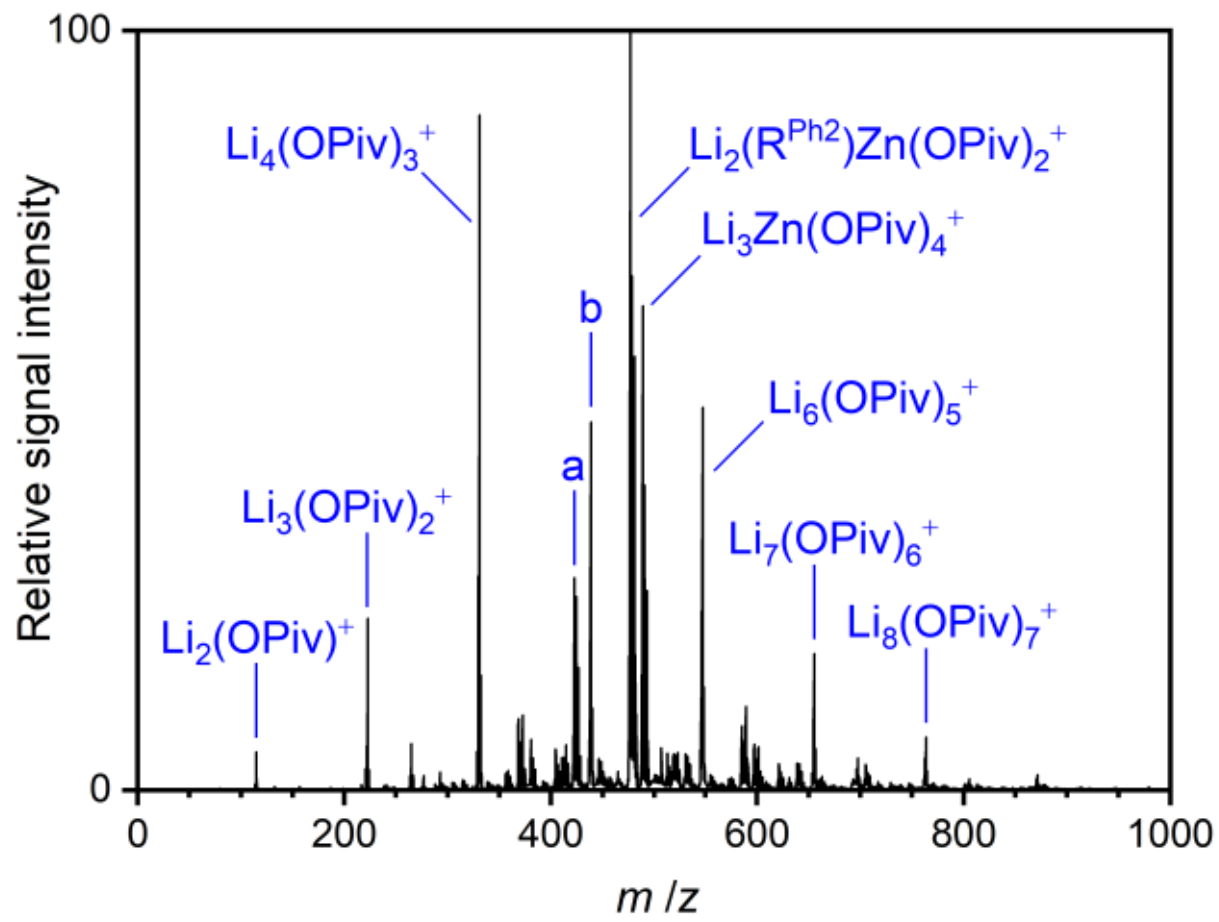

Figure S16. Positive-ion mode ESI-mass spectrum of a solution of $(R^{Ph2})Zn(OPiv)\cdot Li(OPiv)\cdot LiCl$ in THF (0.038 M), a = $Li_3Zn(OPiv)_3Cl^+$, b = $Li_5(OPiv)_4^+$.

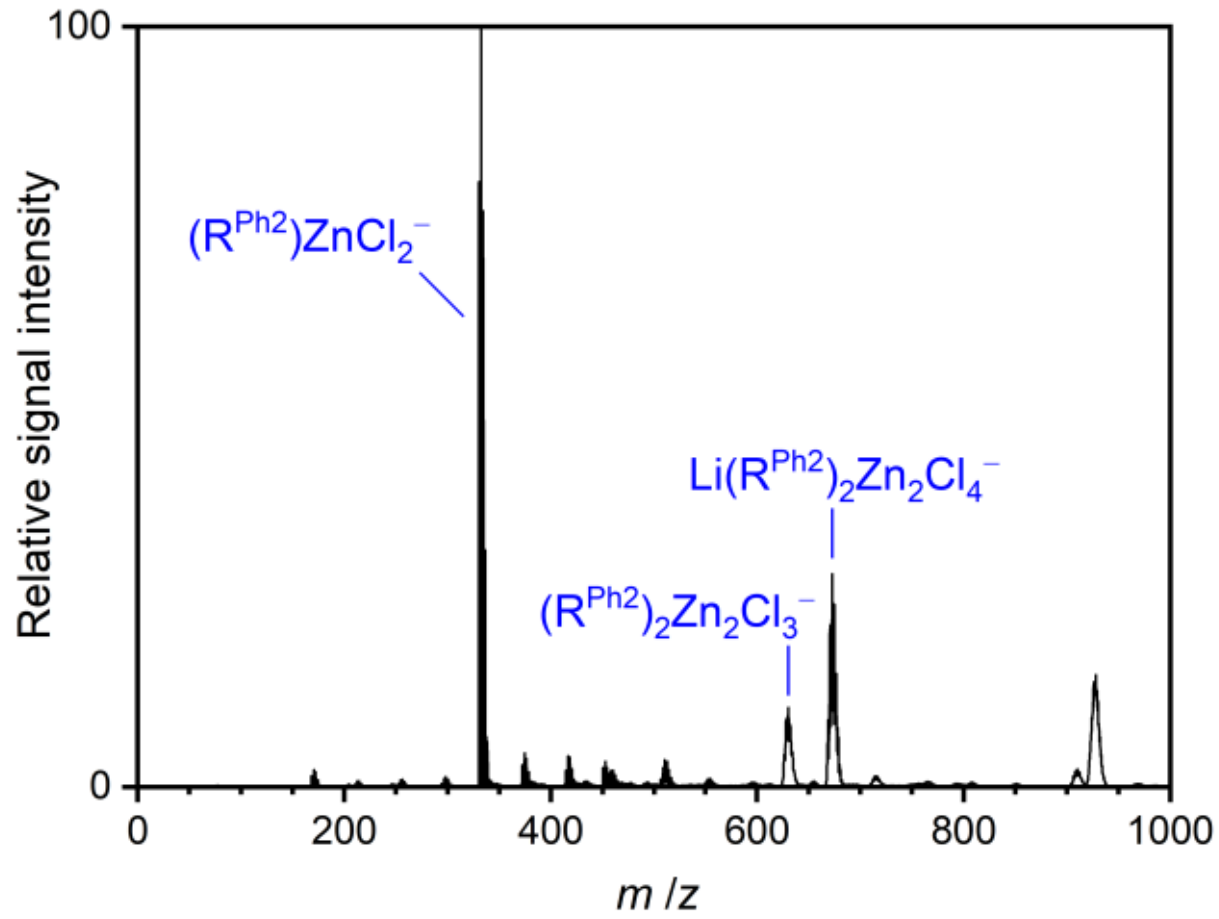

Figure S17. Negative-ion mode ESI-mass spectrum of a solution of $(R^{Ph2})ZnCl \cdot 2\,LiCl$ in THF (0.038 M).

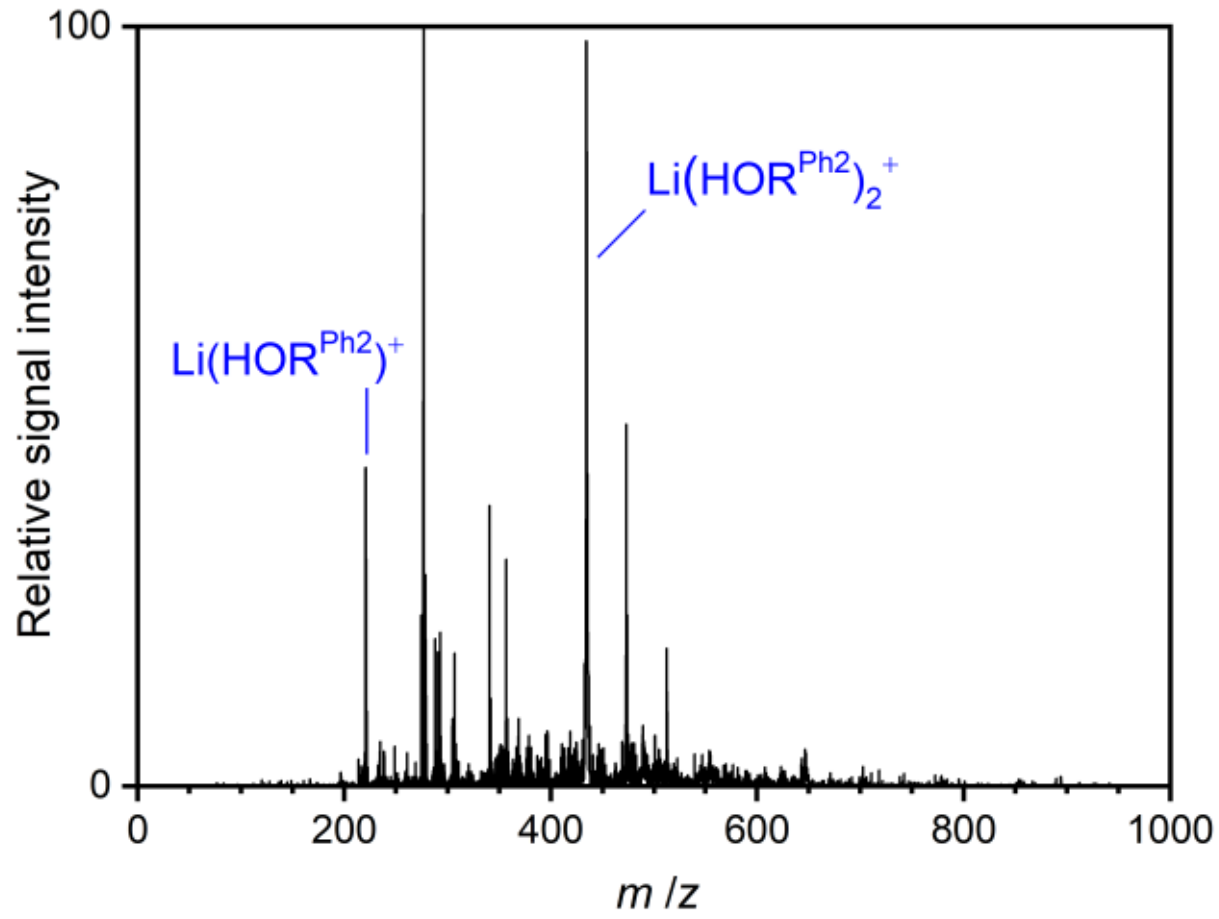

Figure S18. Positive-ion mode ESI-mass spectrum of a solution of $(R^{Ph2})ZnCl \cdot 2\,LiCl$ in THF (0.038 M).

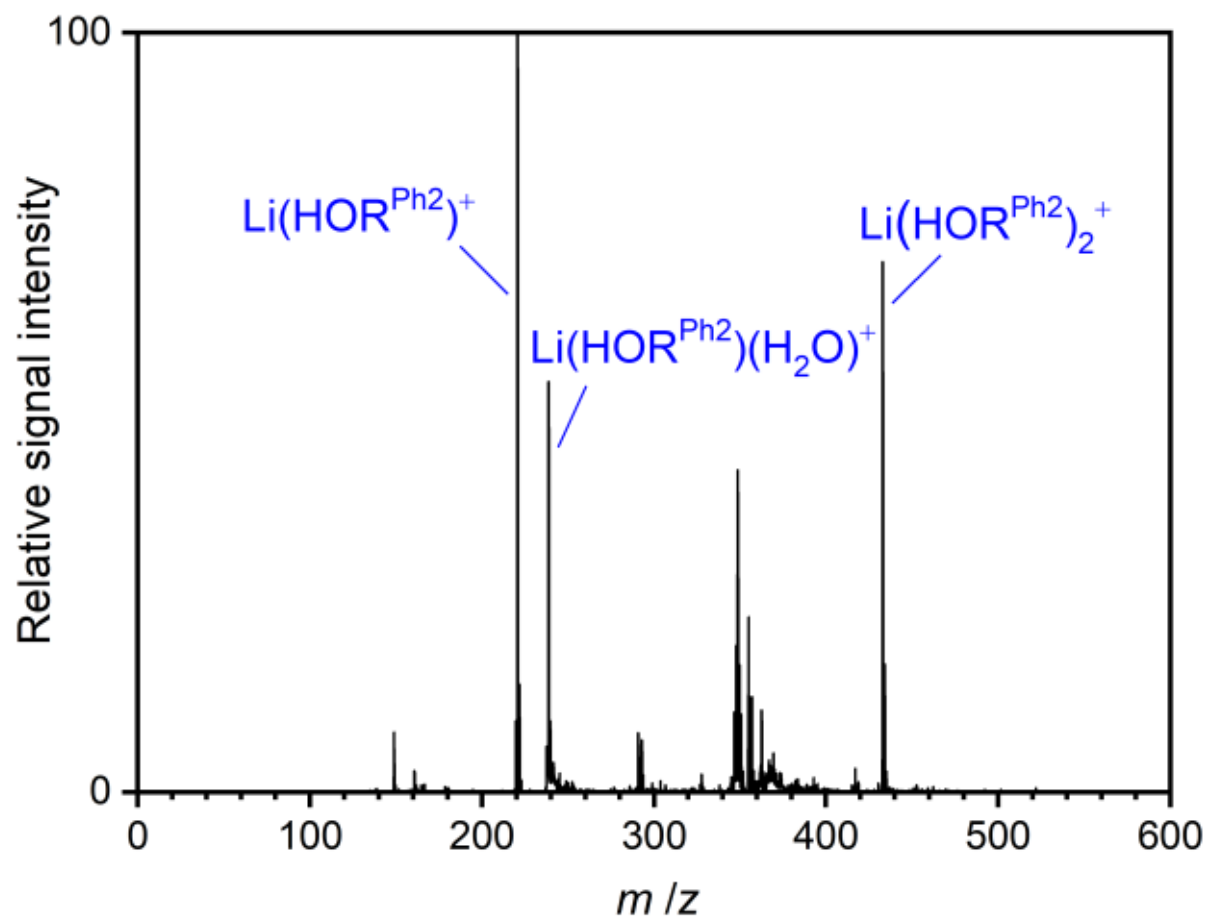

Figure S19. Mass spectrum of mass-selected $Li(HOR^{Ph2})_2^+$ (*m/z* = 435) and its fragment ions formed upon collision-induced dissociation.

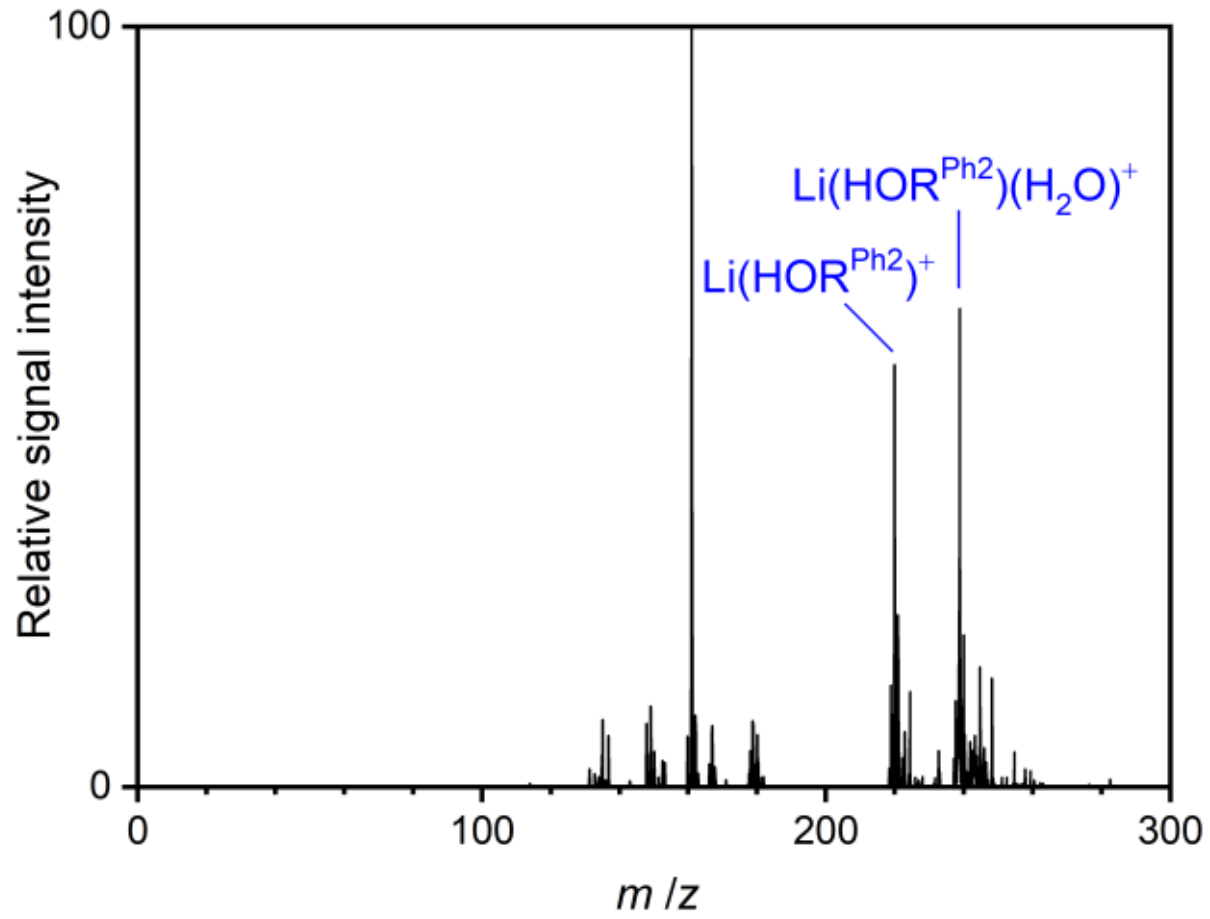

Figure S20. Mass spectrum of mass-selected Li(HOR^{Ph2})$^+$ *(m/z* = 221) and its fragment ions formed upon collision-induced dissociation.

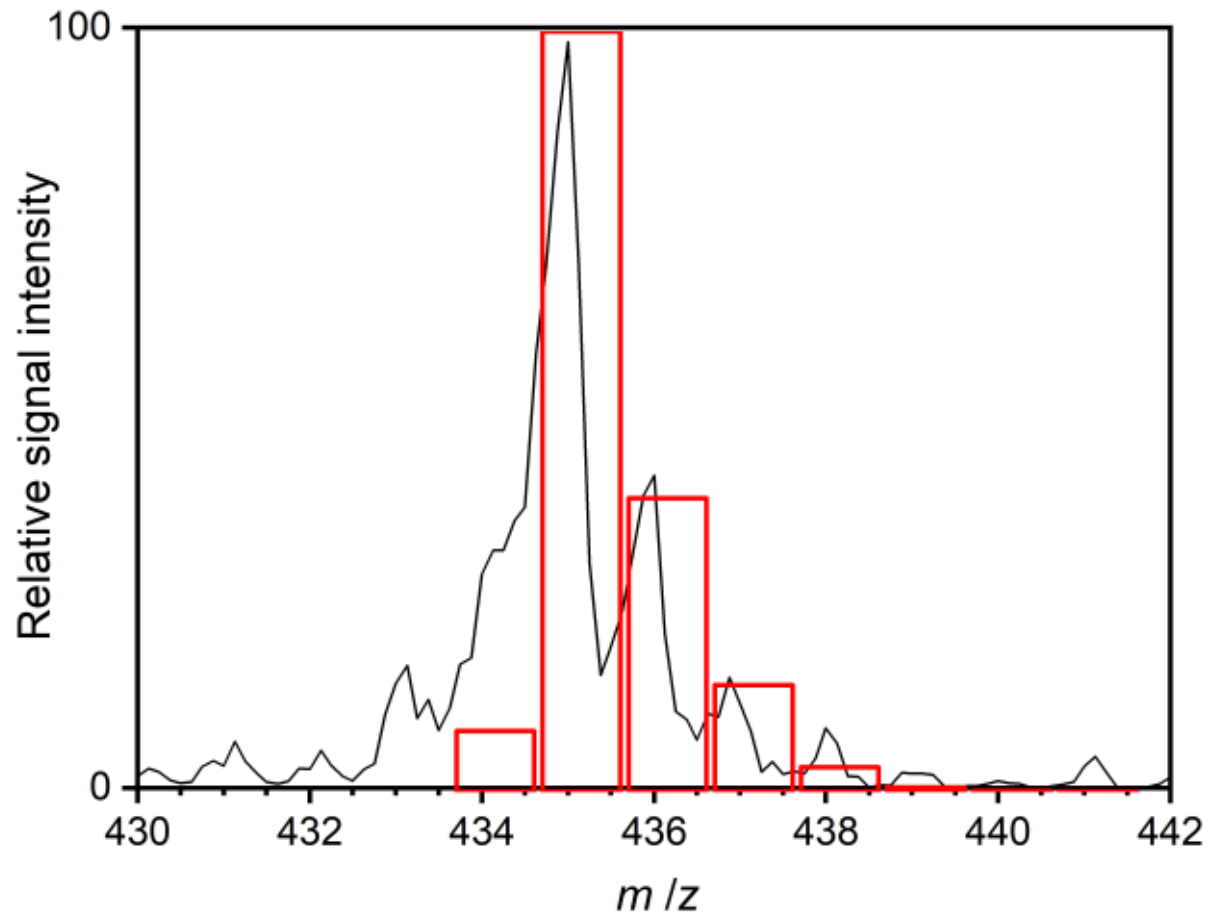

Figure S21. Measured (black) and simulated (red) isotope pattern of Li(HOR^{Ph2})$_2^+$.

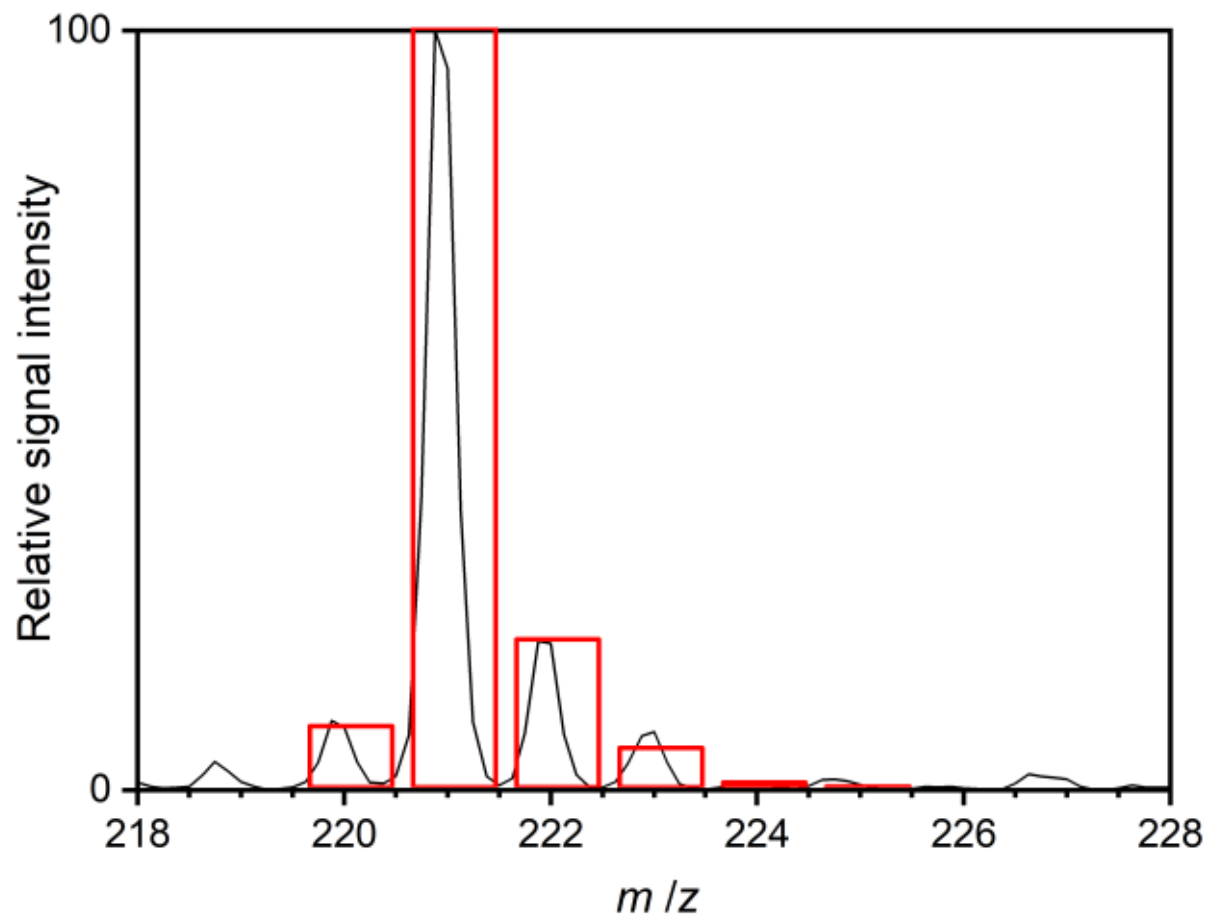

Figure S22. Measured (black) and simulated (red) isotope pattern of Li(HOR^{Ph2})$^+$.

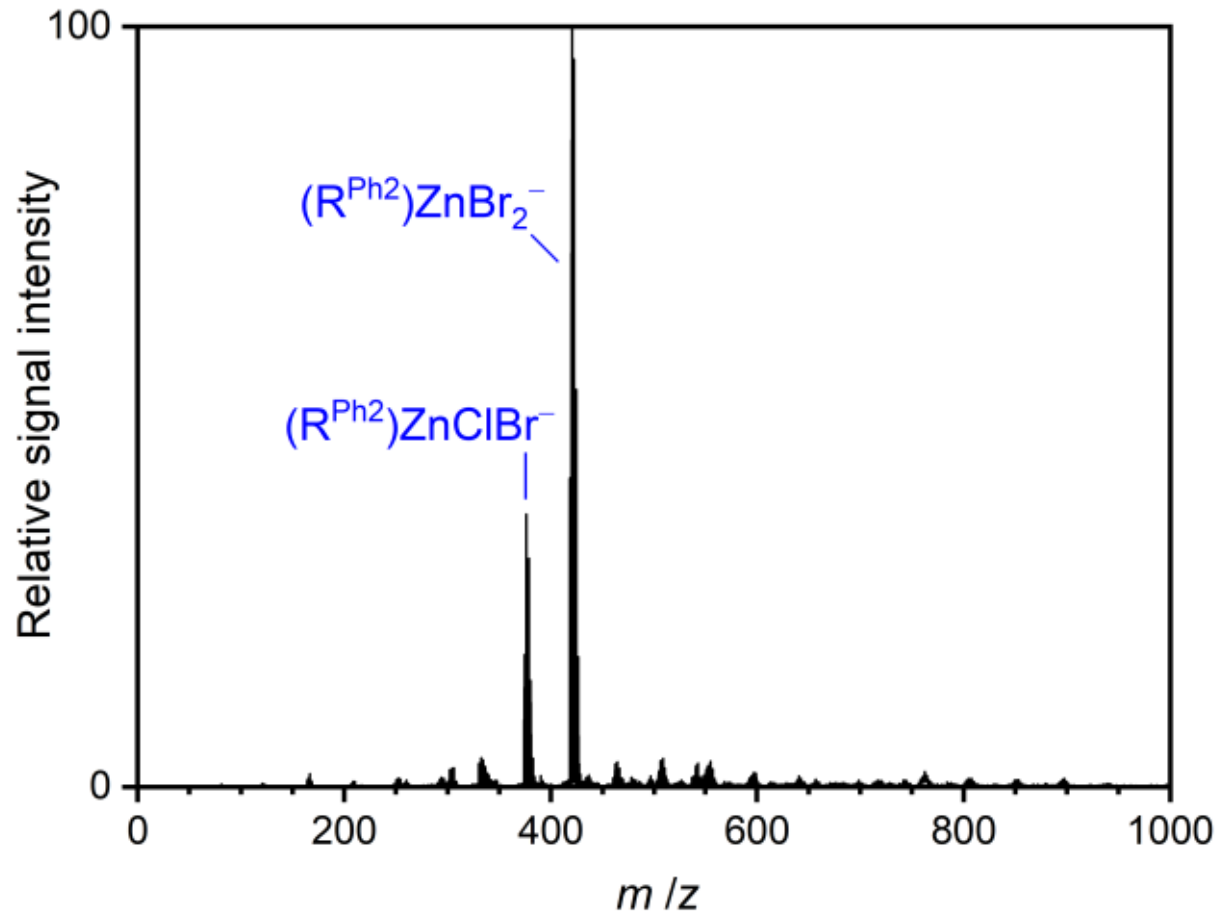

Figure S23. Negative-ion mode ESI-mass spectrum of a solution of $(R^{Ph2})ZnBr·LiCl·LiBr$ in THF (0.038 M).

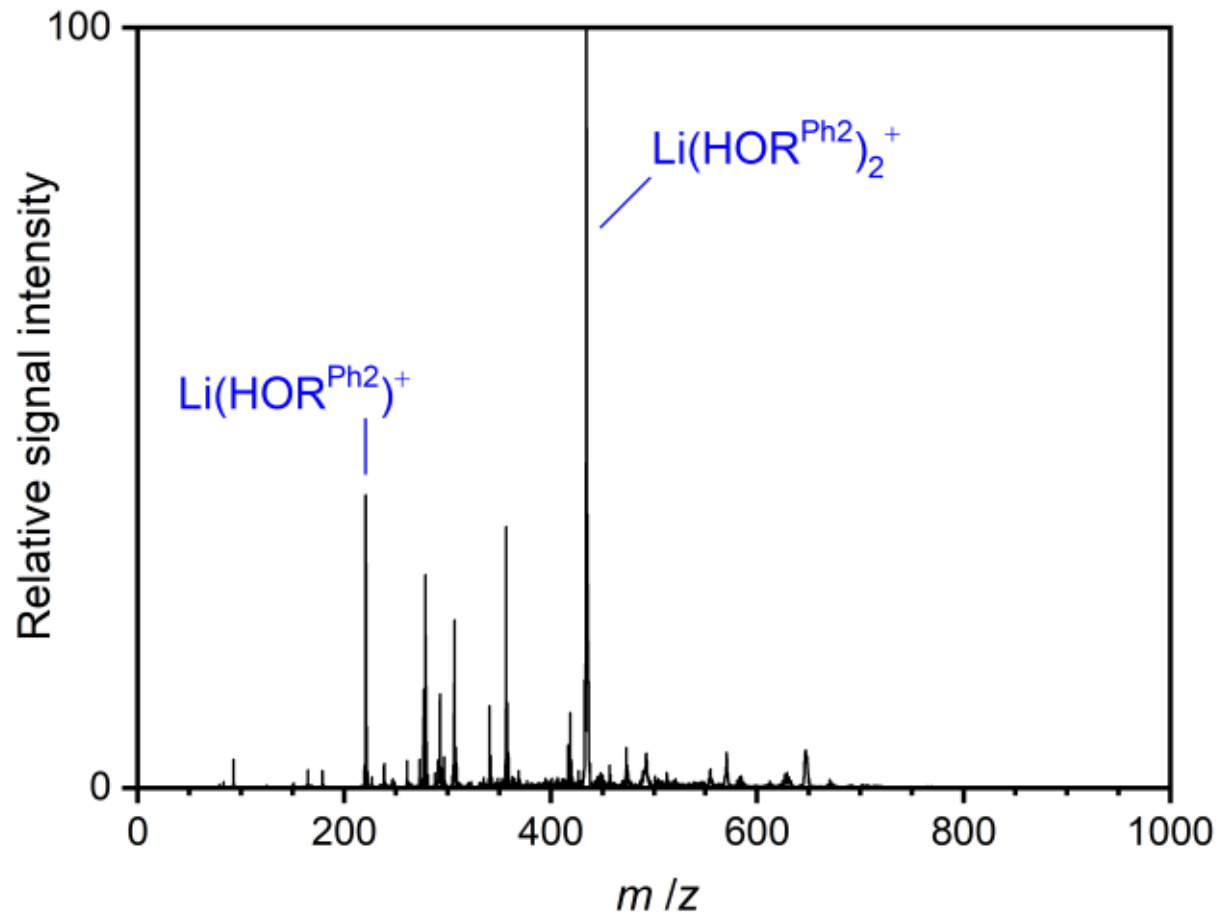

Figure S24. Positive-ion mode ESI-mass spectrum of a solution of $(R^{Ph2})ZnBr·LiCl·LiBr$ in THF (0.038 M).

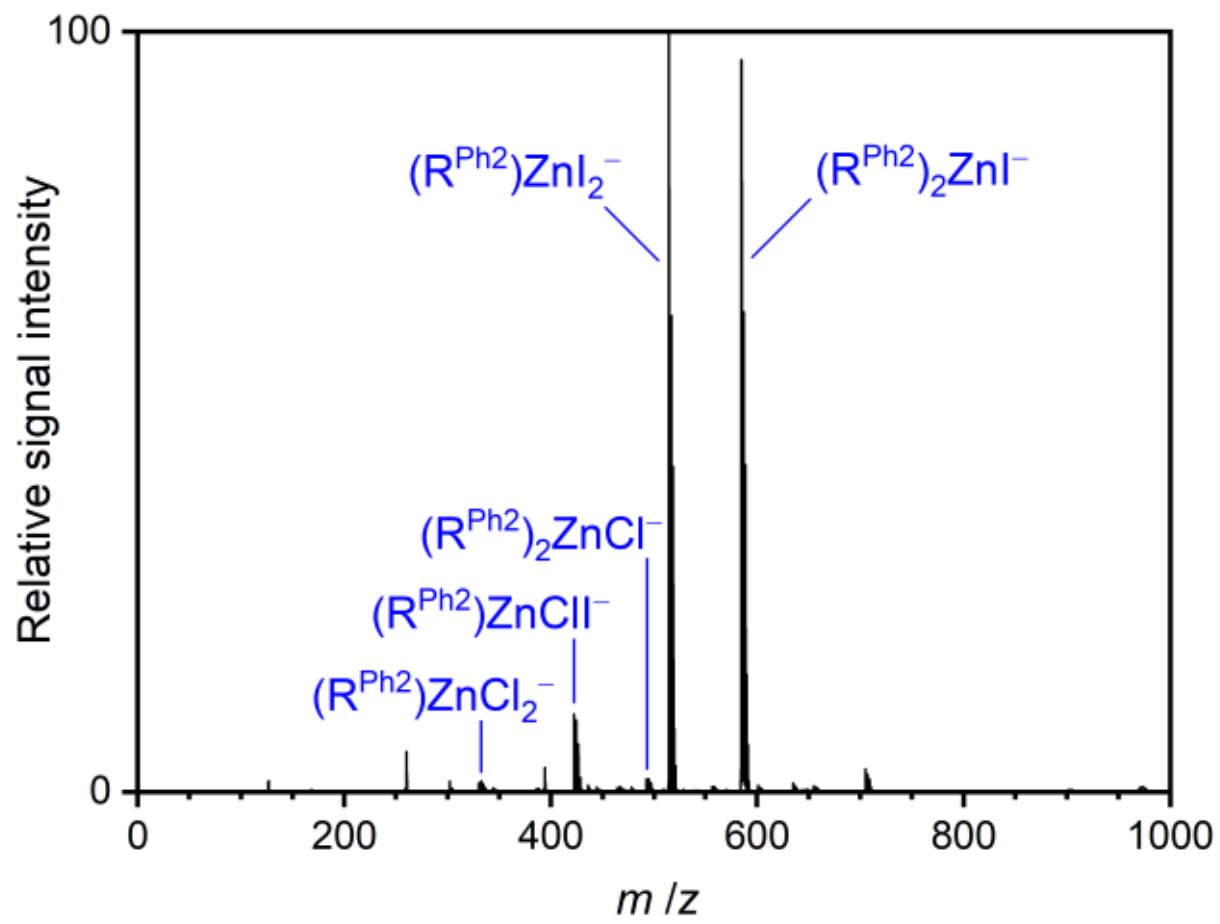

Figure S25. Negative-ion mode ESI-mass spectrum of a solution of $(R^{Ph2})ZnI·LiCl·LiI$ in THF (0.038 M).

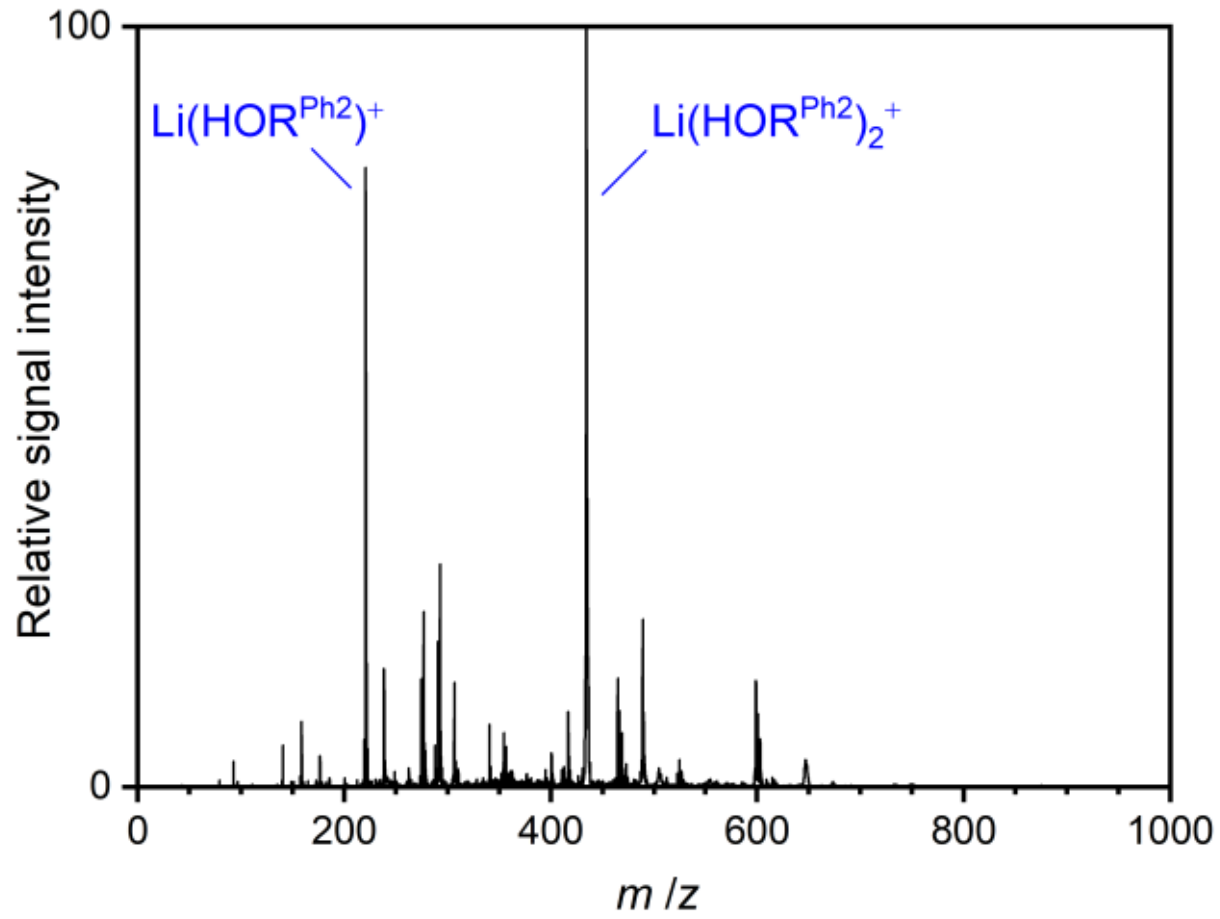

Figure S26. Positive-ion mode ESI-mass spectrum of a solution of $(R^{Ph2})ZnI \cdot LiCl \cdot LiI$ in THF (0.038 M).

2.3 (R^{Ph3})ZnX (X = OPiv, Cl, Br, I, OAc) systems

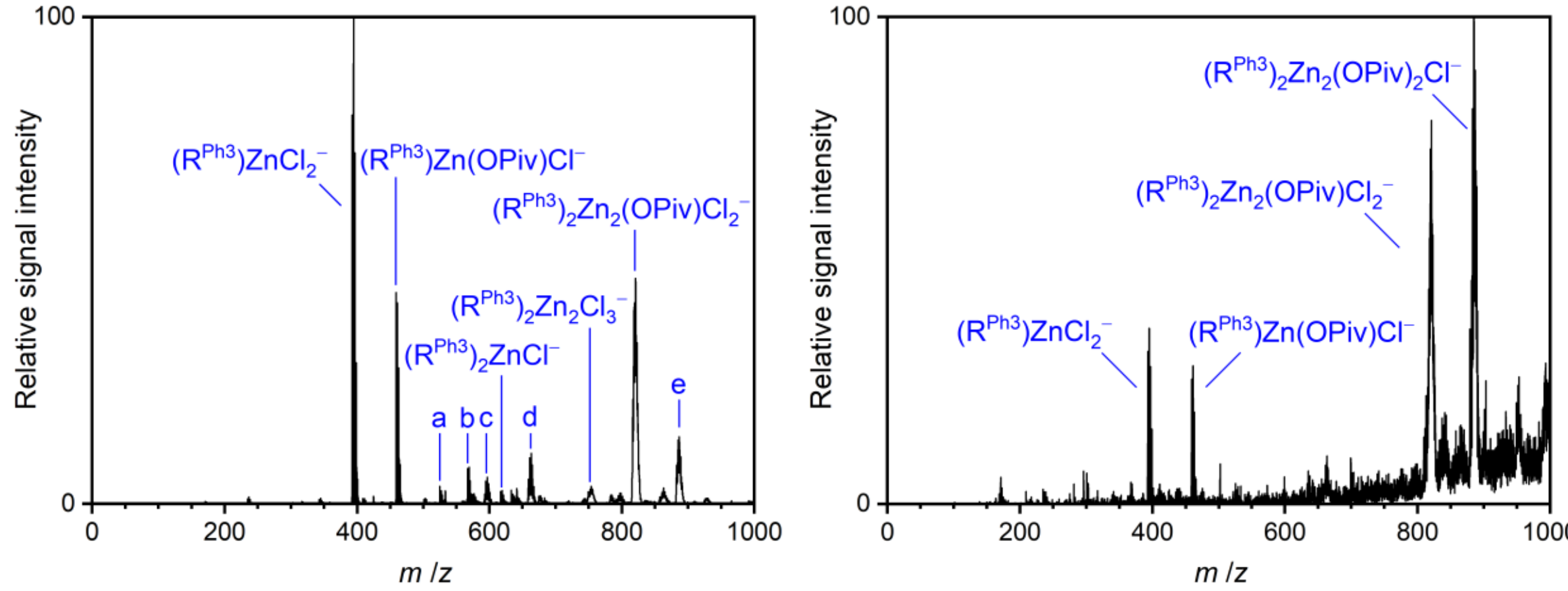

Figure S27. Negative-ion mode ESI-mass spectra of solutions of (R^{Ph3})Zn(OPiv)·Li(OPiv)·LiCl in THF (0.038 M) without (left) and with TMEDA (right), a = $(R^{Ph3})Zn(OPiv)_2^-$, b = $Li(R^{Ph3})Zn(OPiv)_2Cl^-$, c = $(R^{Ph3})Zn_2(OPiv)Cl_3^-$, d = $(R^{Ph3})Zn_2(OPiv)_2Cl_2^-$, e = $(R^{Ph3})_2Zn_2(OPiv)_2Cl^-$.

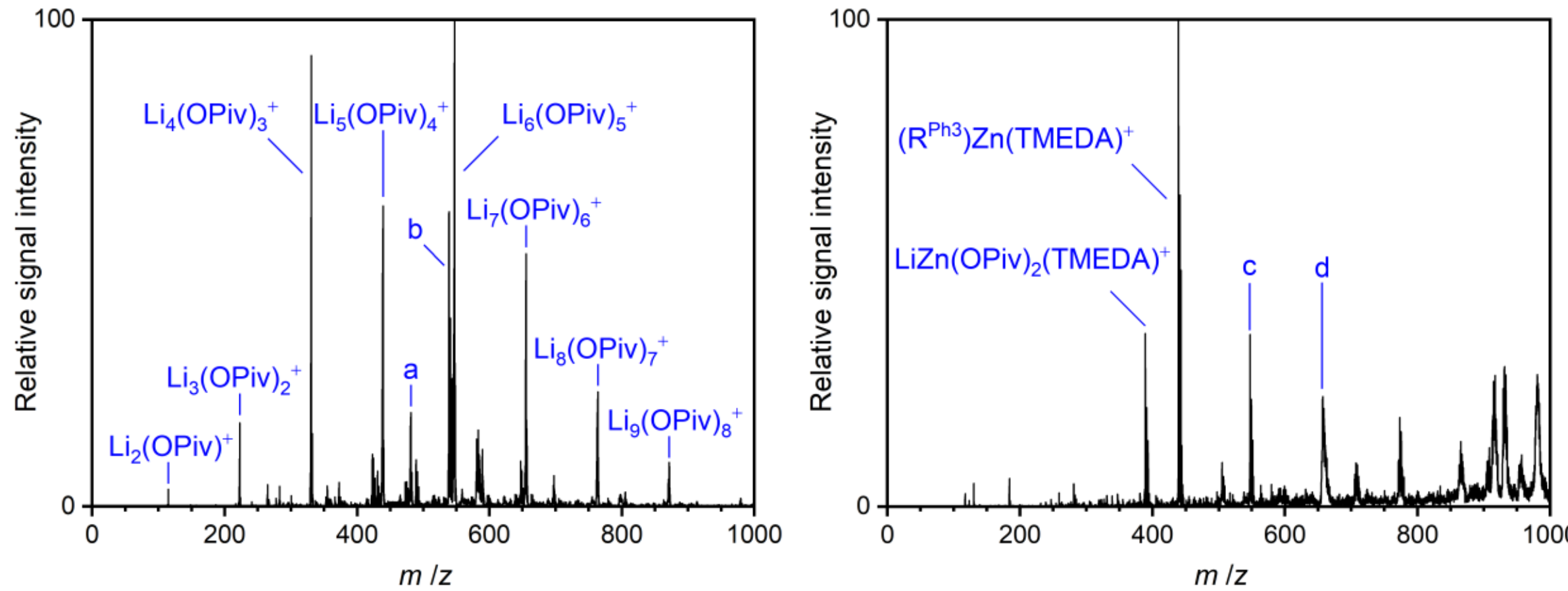

Figure S28. Positive-ion mode ESI-mass spectra of solutions of (R^{Ph3})Zn(OPiv)·Li(OPiv)·LiCl in THF (0.038 M) without (left) and with TMEDA (right), a = $Li_6(OPiv)_4Cl^+$, b = $Li_2(R^{Ph3})Zn(OPiv)_2^+$, c = $Li(R^{Ph3})Zn(OPiv)(TMEDA)^+$, d = $Li_2(R^{Ph3})Zn(OPiv)_2(TMEDA)^+$.

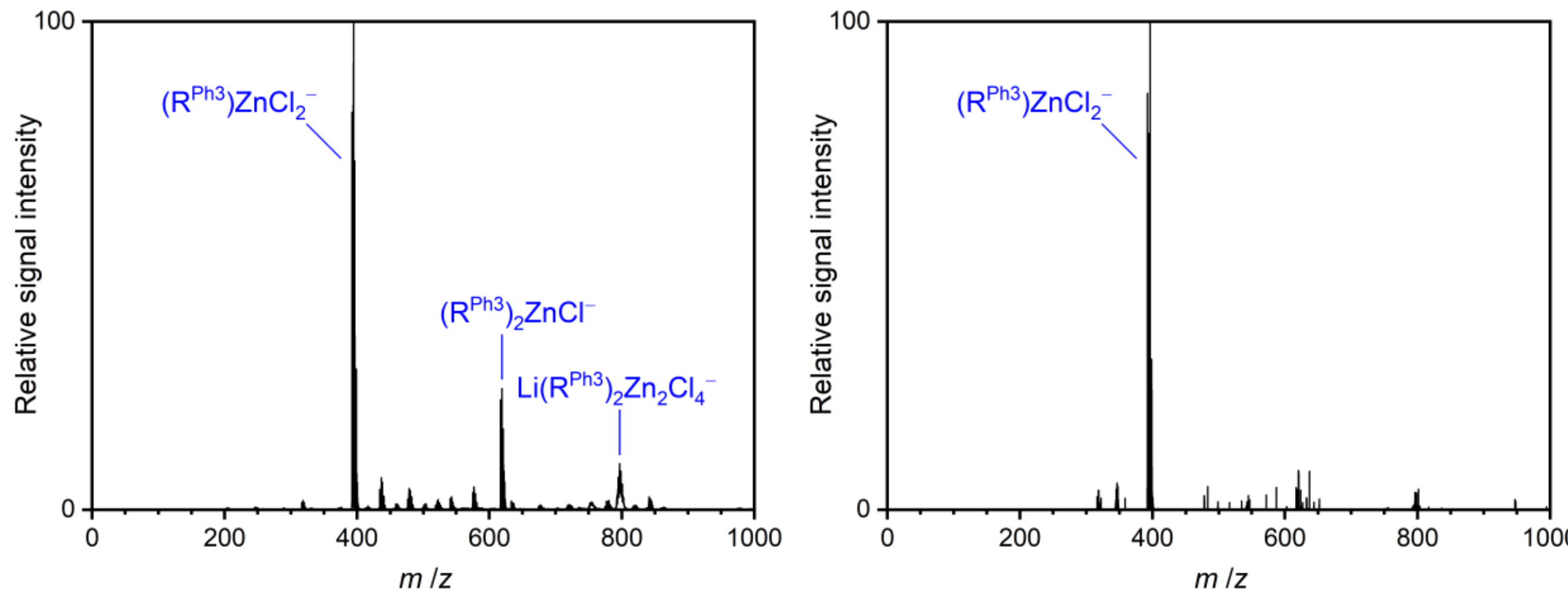

Figure S29. Negative-ion mode ESI-mass spectrum of solutions of (R^{Ph3})ZnCl·2 LiCl in THF (0.038 M) without (left) and with TMEDA (right).

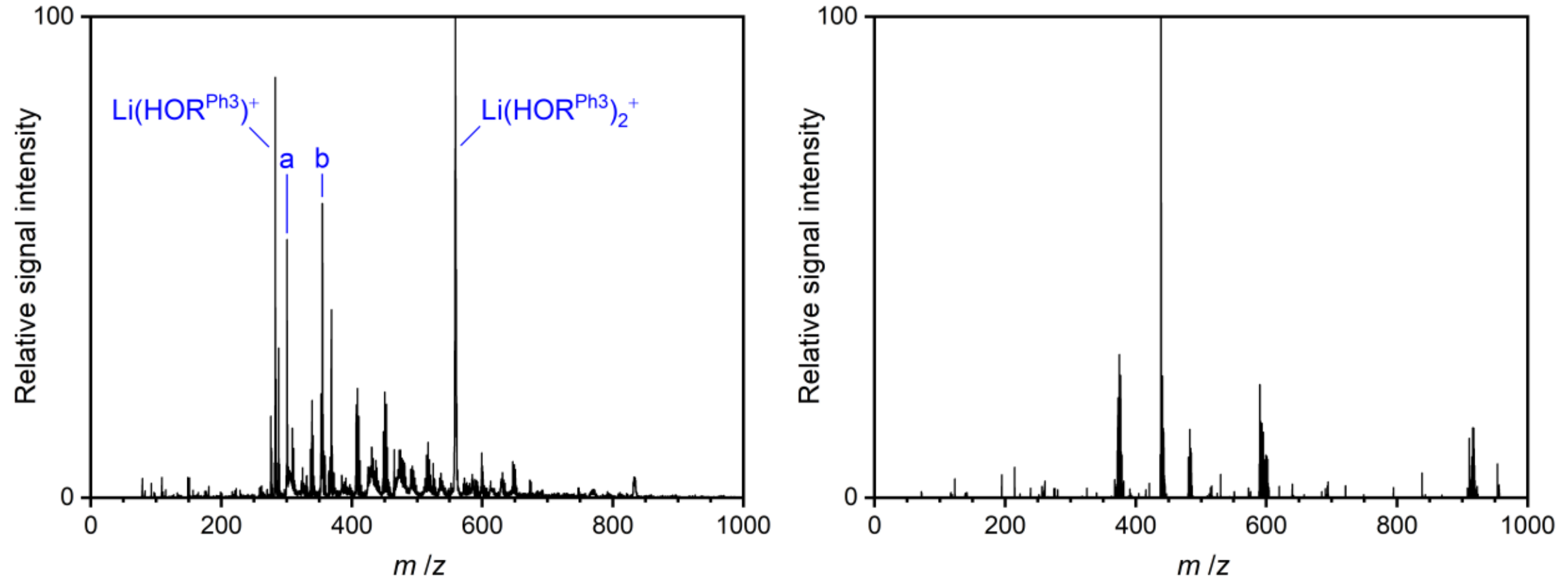

Figure S30. Positive-ion mode ESI-mass spectra of (R^{Ph3})ZnCl·2 LiCl in THF (0.038 M) without (left) and with TMEDA (right), a = Li(HOR^{Ph3})(H_2O)$^+$, b = $Li_4(OH)_3$(HOR^{Ph3})$^+$.

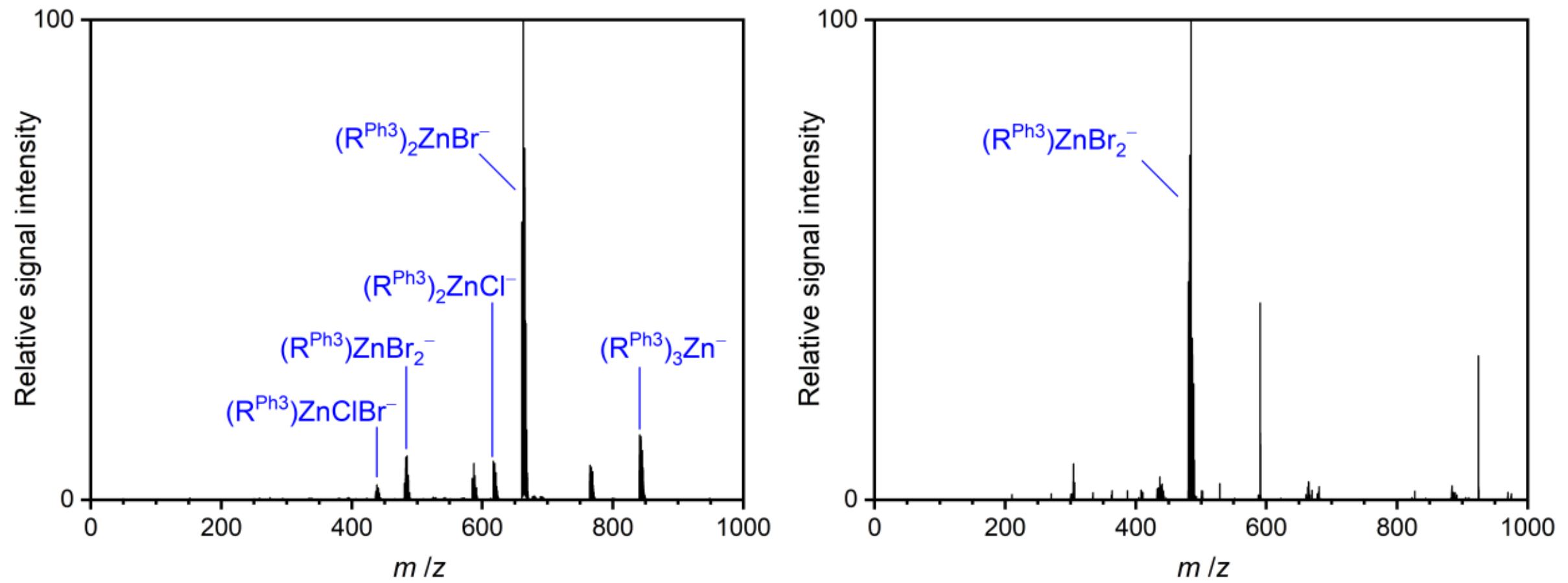

Figure S31. Negative-ion mode ESI-mass spectra of solutions of (R^{Ph3})ZnBr·LiCl·LiBr in THF (0.038 M) without (left) and with TMEDA (right).

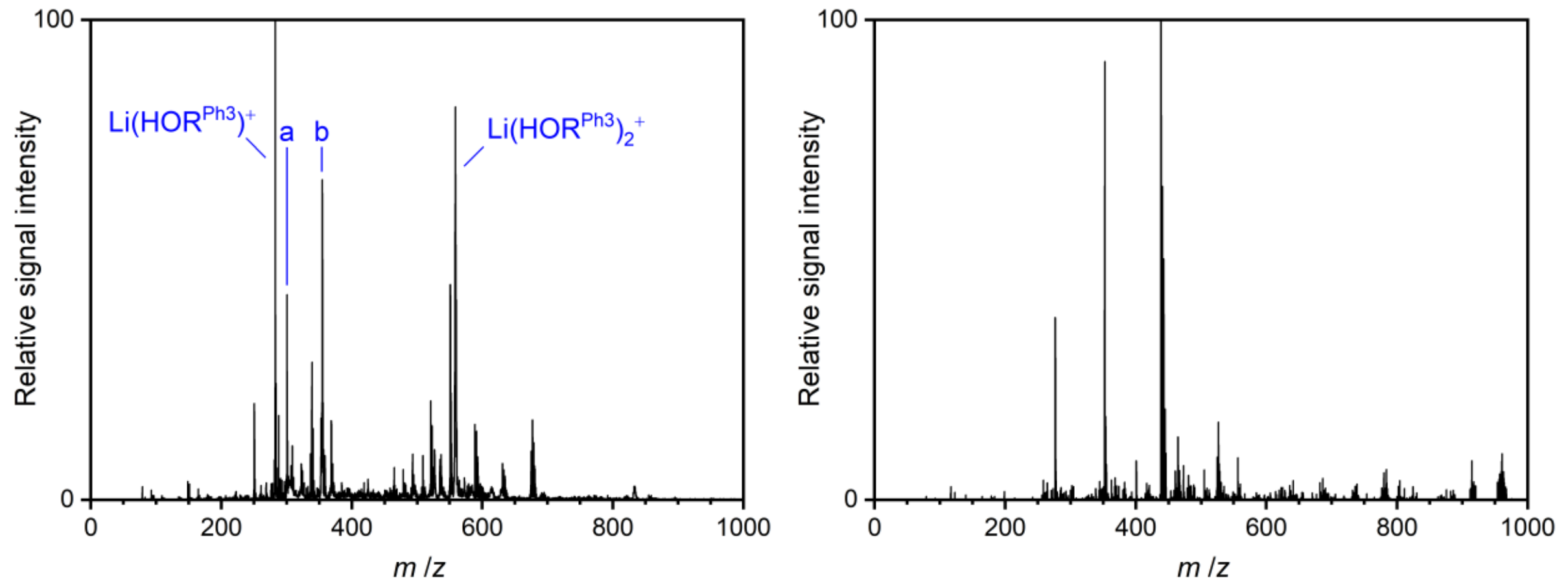

Figure S32. Positive-ion mode ESI-mass spectra of solutions of (R^{Ph3})ZnBr·LiCl·LiBr in THF (0.038 M) without (left) and with TMEDA (right), a = Li(HOR^{Ph3})(H_2O)$^+$, b = Li(HOR^{Ph3})(THF)$^+$.

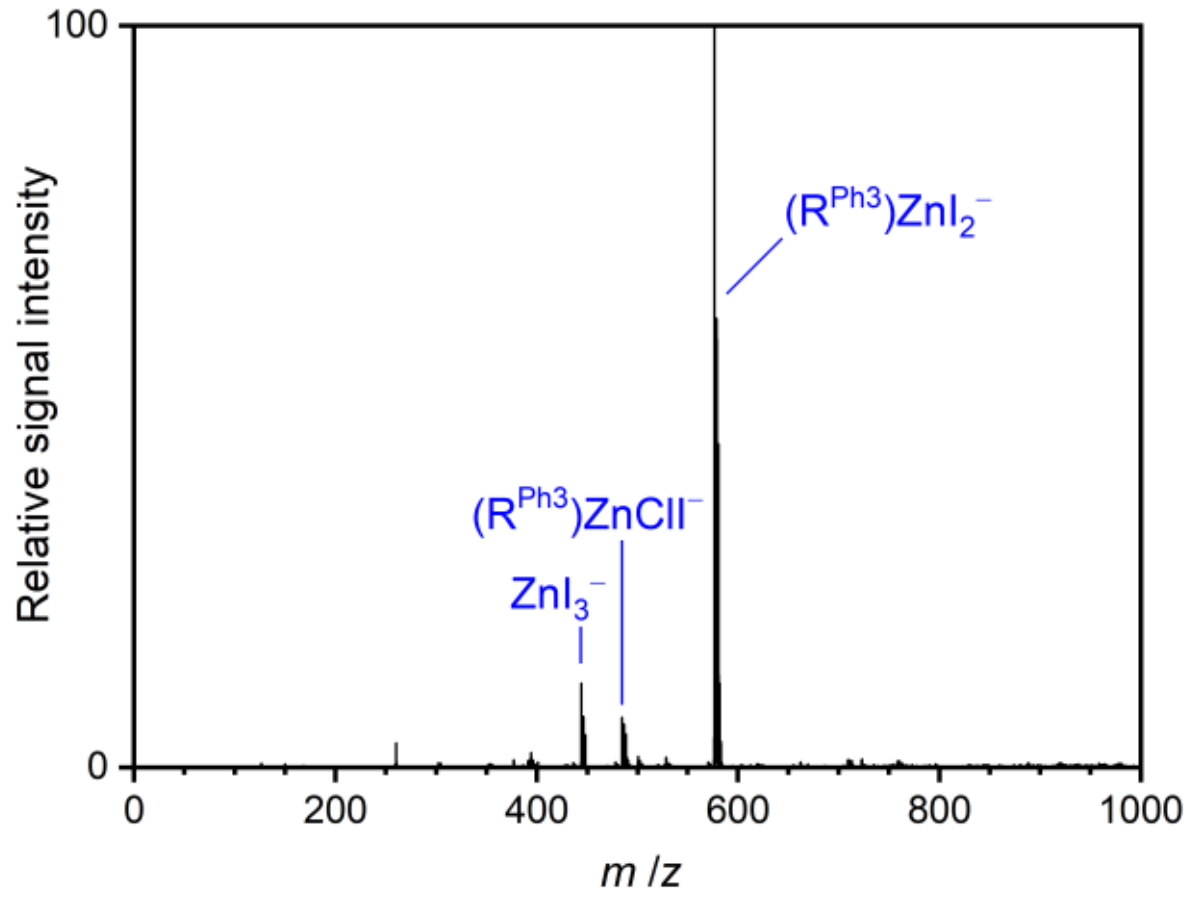

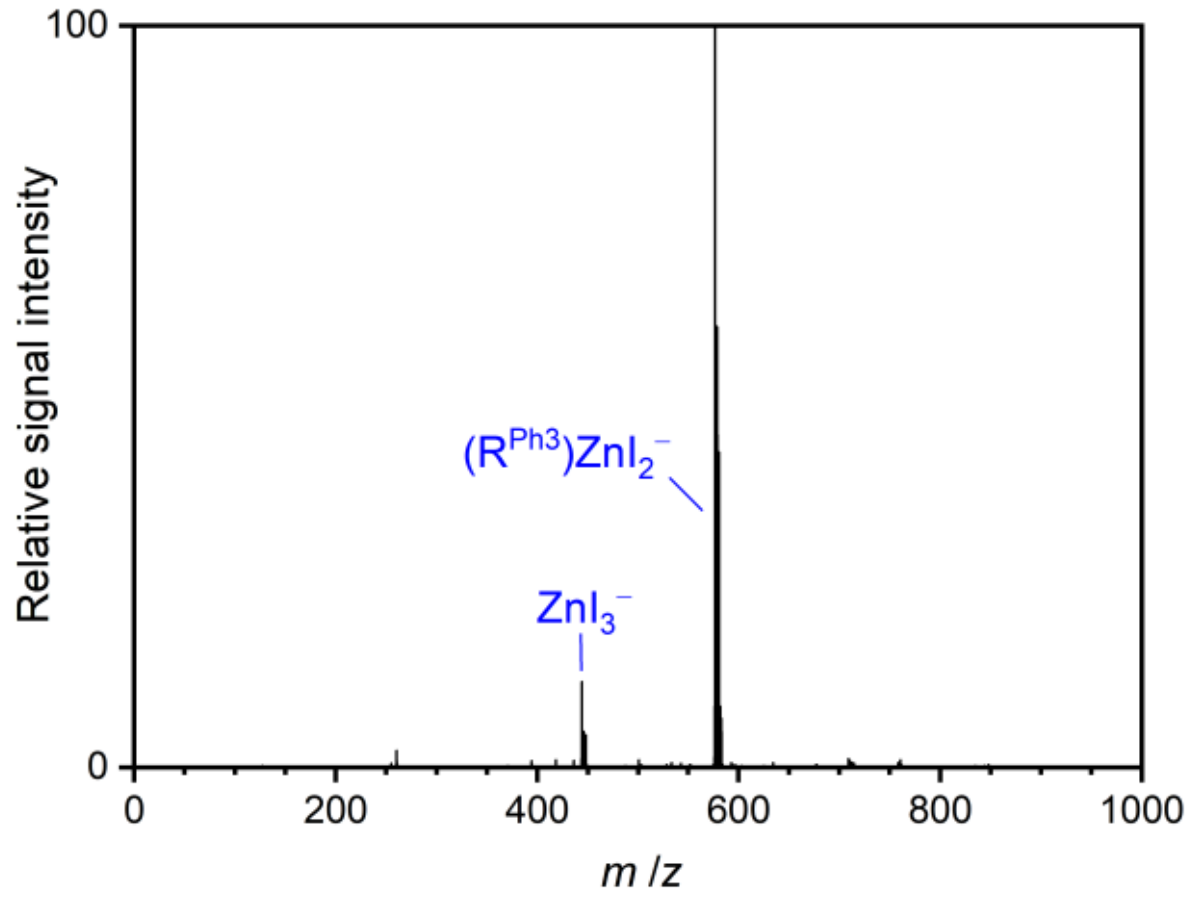

Figure S33. Negative-ion mode ESI-mass spectra of solutions of $(R^{Ph3})ZnI \cdot LiCl \cdot LiI$ in THF (0.038 M) without (left) and with TMEDA (right).

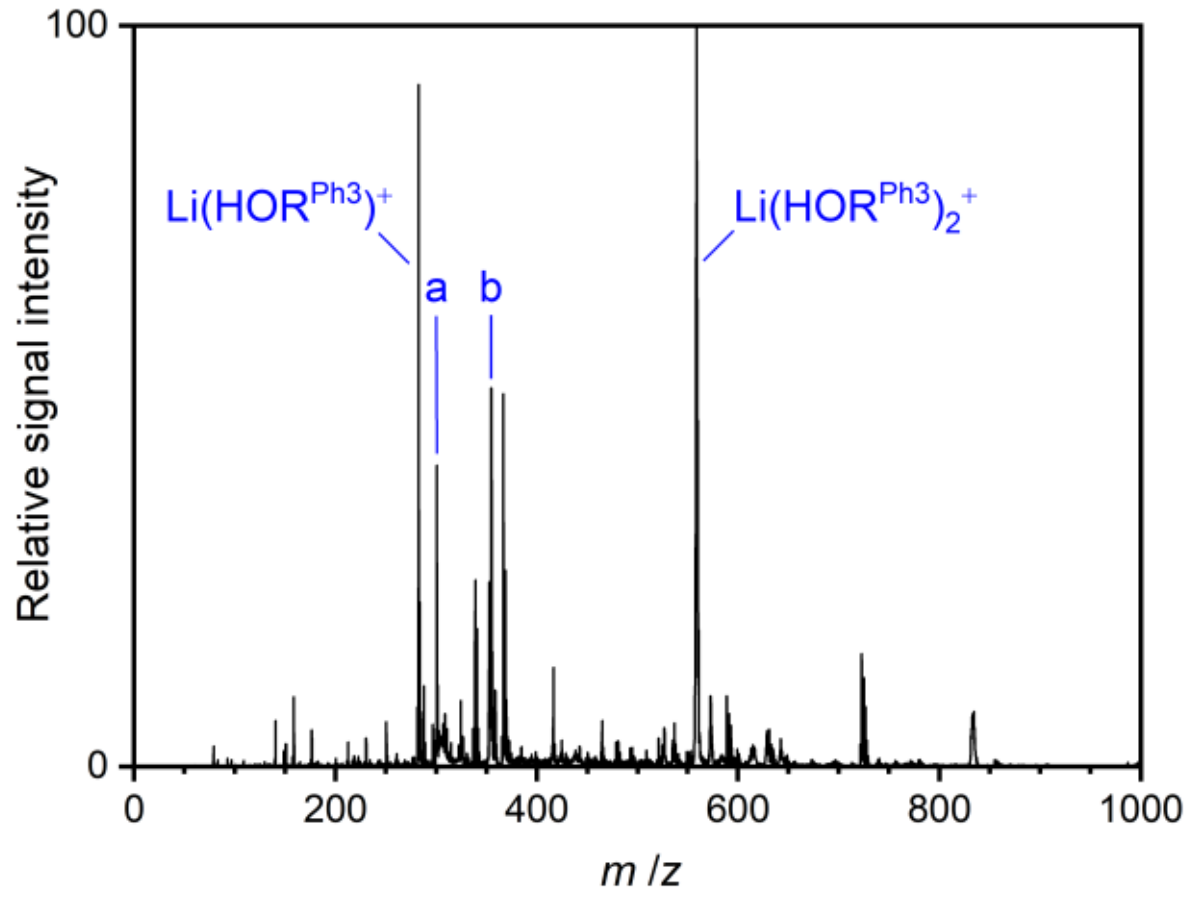

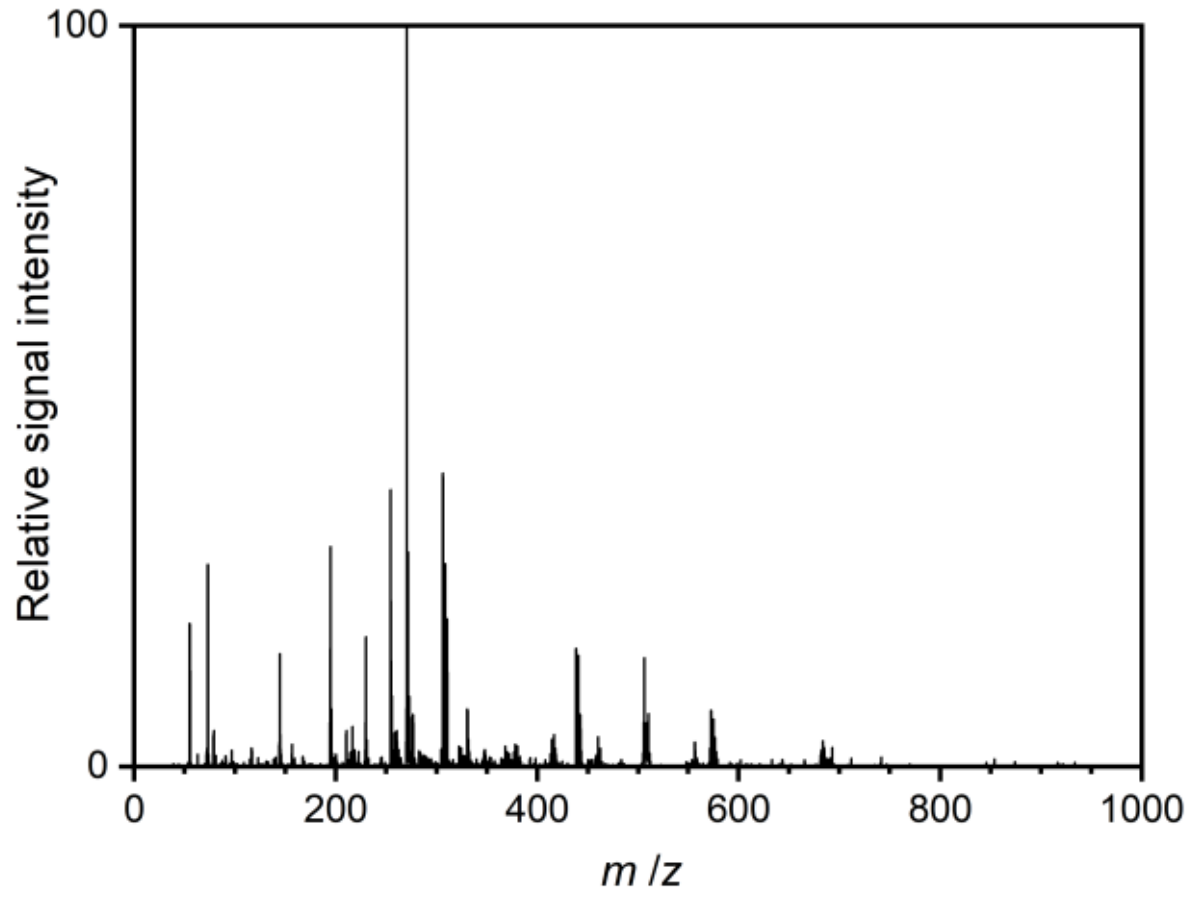

Figure S34. Positive-ion mode ESI-mass spectra of solutions of $(R^{Ph3})ZnI \cdot LiCl \cdot LiI$ in THF (0.038 M) without (left) and with TMEDA (right), a = $Li(HOR^{Ph3})(H_2O)^+$, b = $Li(HOR^{Ph3})(THF)^+$.

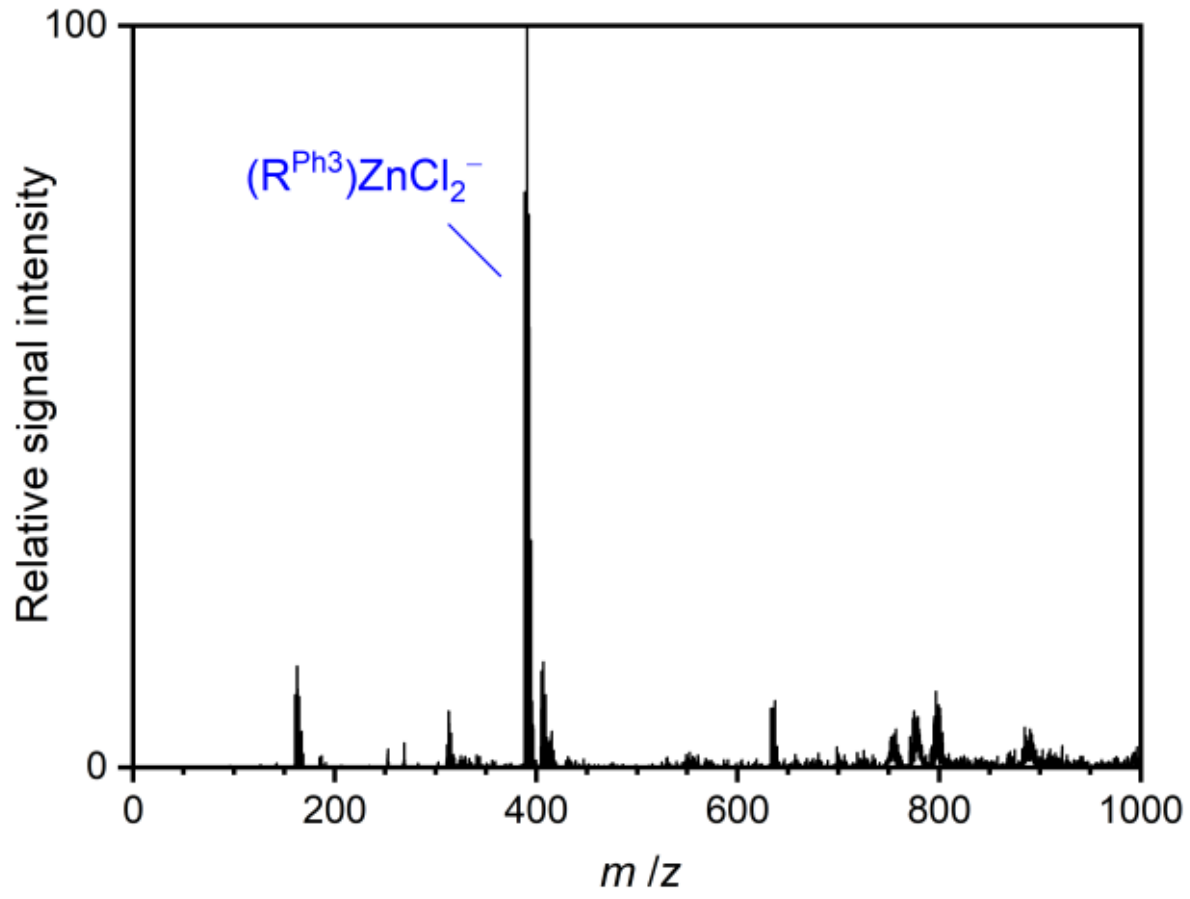

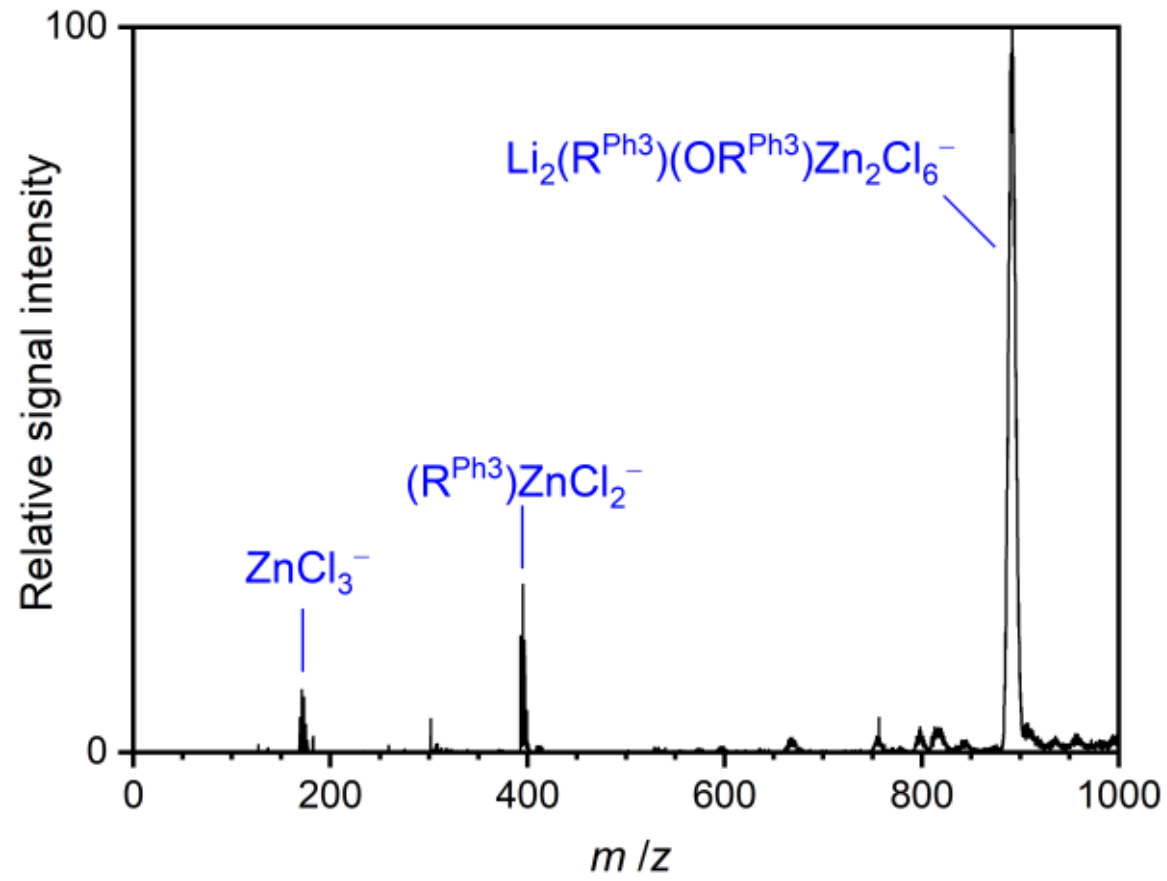

Figure S35. Negative-ion mode ESI-mass spectra of solutions of $(R^{Ph3})ZnOAc \cdot LiCl \cdot LiOAc$ in THF (0.038 M) without (left) and with TMEDA (right).

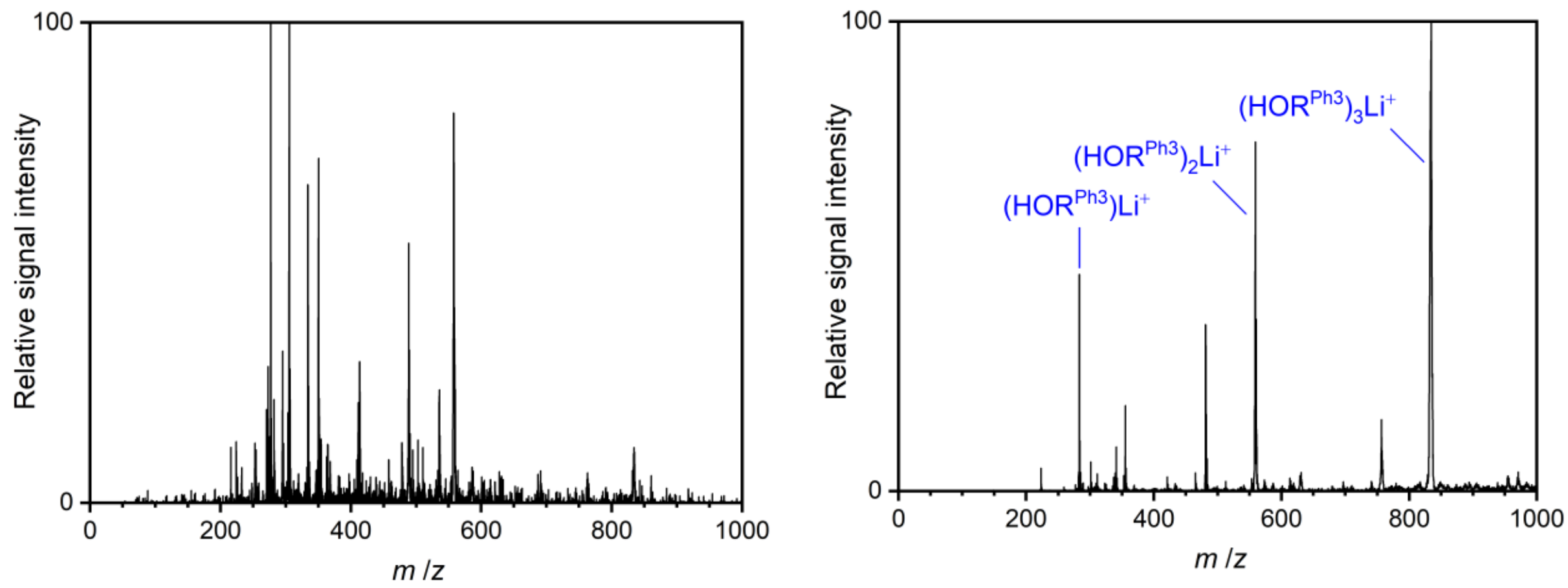

Figure S36. Positive-ion mode ESI-mass spectra of solutions of (R^{Ph3})ZnOAc·LiCl·LiOAc in THF (0.038 M) without (left) and with TMEDA (right).

3. X-ray crystallographic characterization

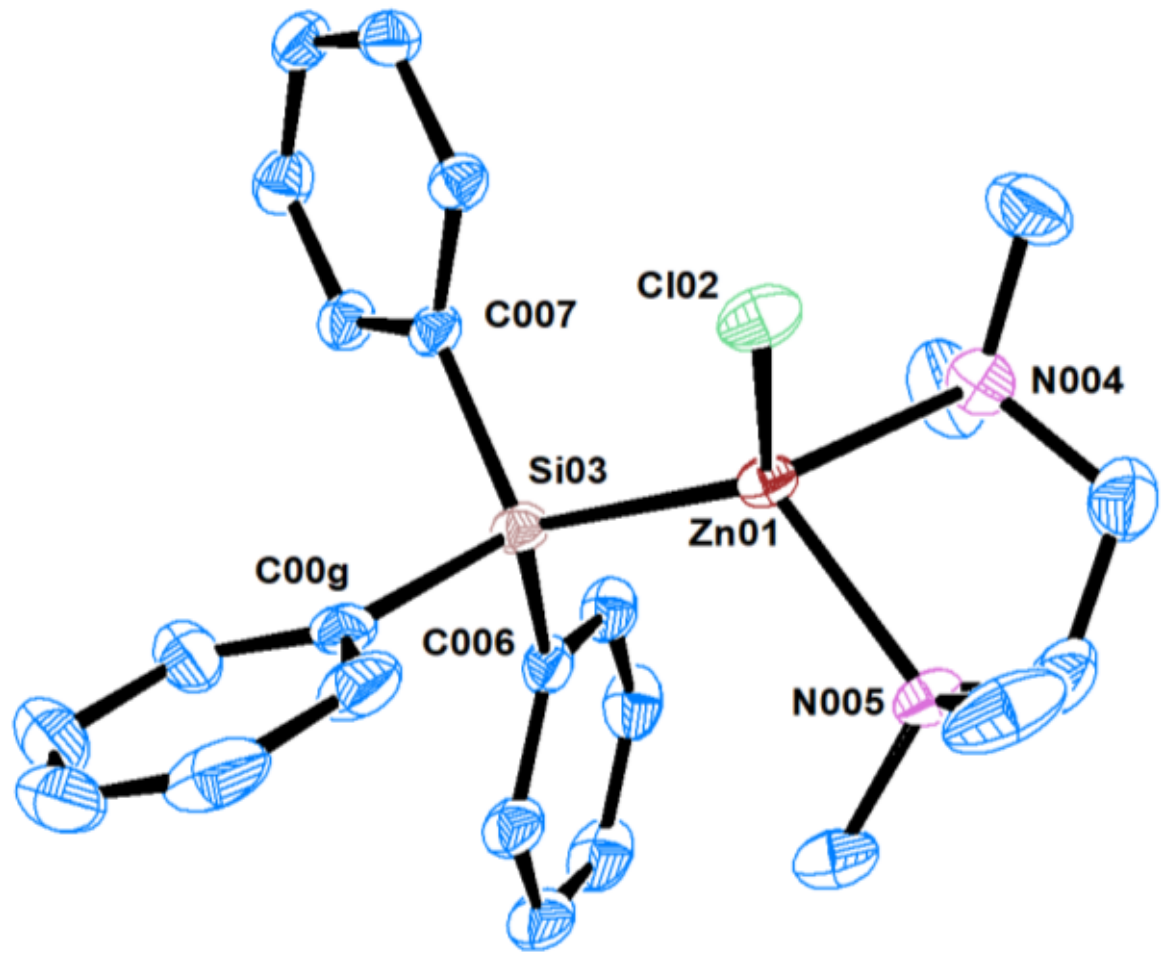

Figure S37. Molecular structure of $Ph_3SiZnCl$·TMEDA (CCDC 2559901). Selected bond lengths [Å] and angles [°]: Zn01–Cl02 2.2688(8), Zn01–Si03 2.3839(8), Zn01–N004 2.145(2), Zn01–N005 2.128(2), Si03–C006 1.896(3), Si03–C007 1.893(3), Si03–C00g 1.895(3), Cl02–Zn01–Si03 120.22(3), N004–Zn01–Cl02 103.15(7), N004–Zn01–Si03 118.94(7), N005–Zn01–Cl02 105.32(7), N005–Zn01–Si03 117.77(7), N005–Zn01–N004 85.36(10), C006–Si03–Zn01 115.93(9), C007–Si03–Zn01 110.99(9), C007–Si03–C006 107.85(12), C007–Si03–C00g 105.60(12), C00g–Si03–Zn01 111.29(11), C00g–Si03–C006 104.48(13). Thermal ellipsoids are shown at the 50% probability level. Hydrogen atoms are omitted for clarity.

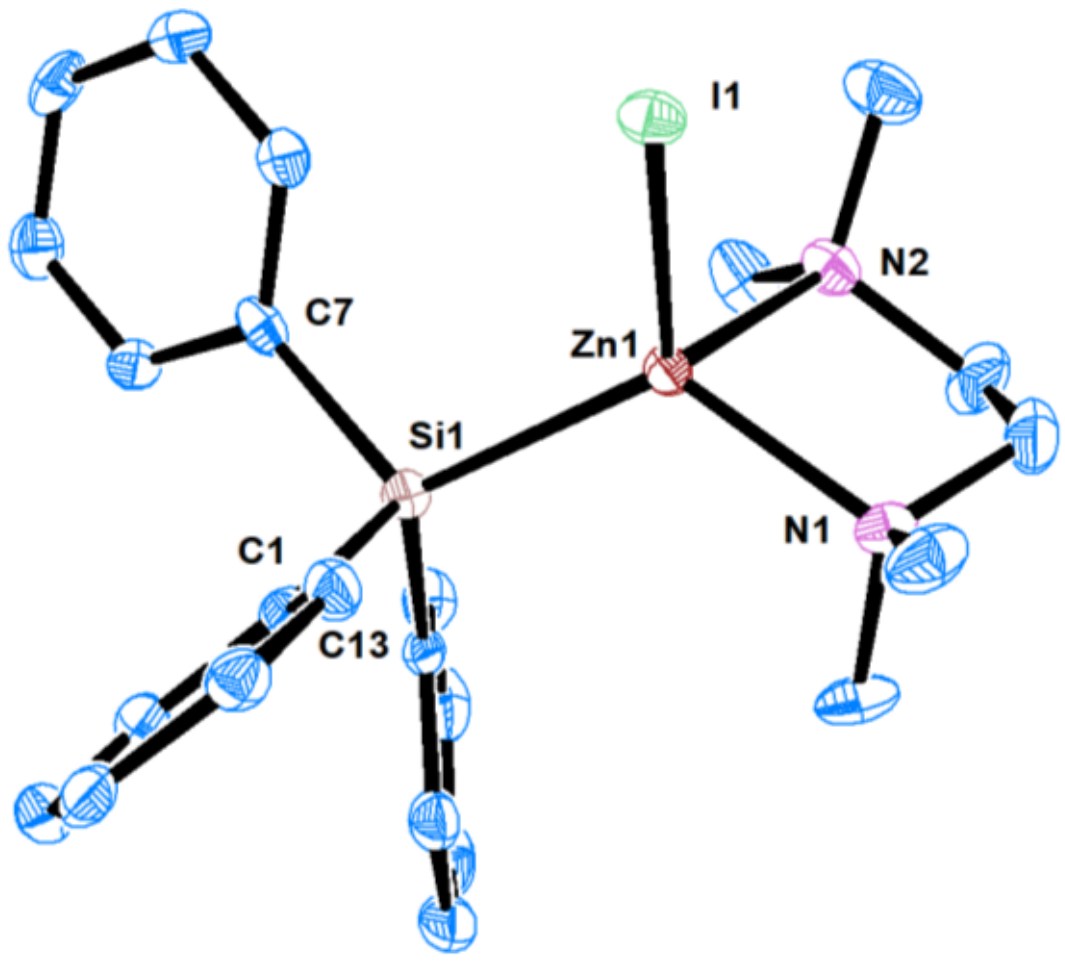

Figure S38. Molecular structure of Ph_3SiZnI·TMEDA (CCDC 2559904). Selected bond lengths [Å] and angles [°]: I1–Zn1 2.6418(6), Zn1–Si1 2.3901(13), Zn1–N2 2.151(4), Zn1–N1 2.117(4), Si1–C13 1.898(4), Si1–C7 1.904(5), Si1–C1 1.902(4), Si1–Zn1–I1 116.82(4), N2–Zn1–I1 105.05(10), N2–Zn1–Si1 119.20(11), N1–Zn1–I1 104.30(10), N1–Zn1–Si1 121.03(10), N1–Zn1–N2 85.32(15), C13–Si1–Zn1 117.35(14), C13–Si1–C7 105.87(19), C13–Si1–C1 105.54(19), C7–Si1–Zn1 110.50(13), C1–Si1–Zn1 109.95(14), C1–Si1–C7 107.04(19). Thermal ellipsoids are shown at the 50% probability level. Hydrogen atoms are omitted for clarity.

4. Time-dependent ESI-mass spectrometric experiments under ambient atmosphere

4.1 (R^{Ph})ZnX·LiX·LiCl (X = OPiv, Cl, Br, I) systems

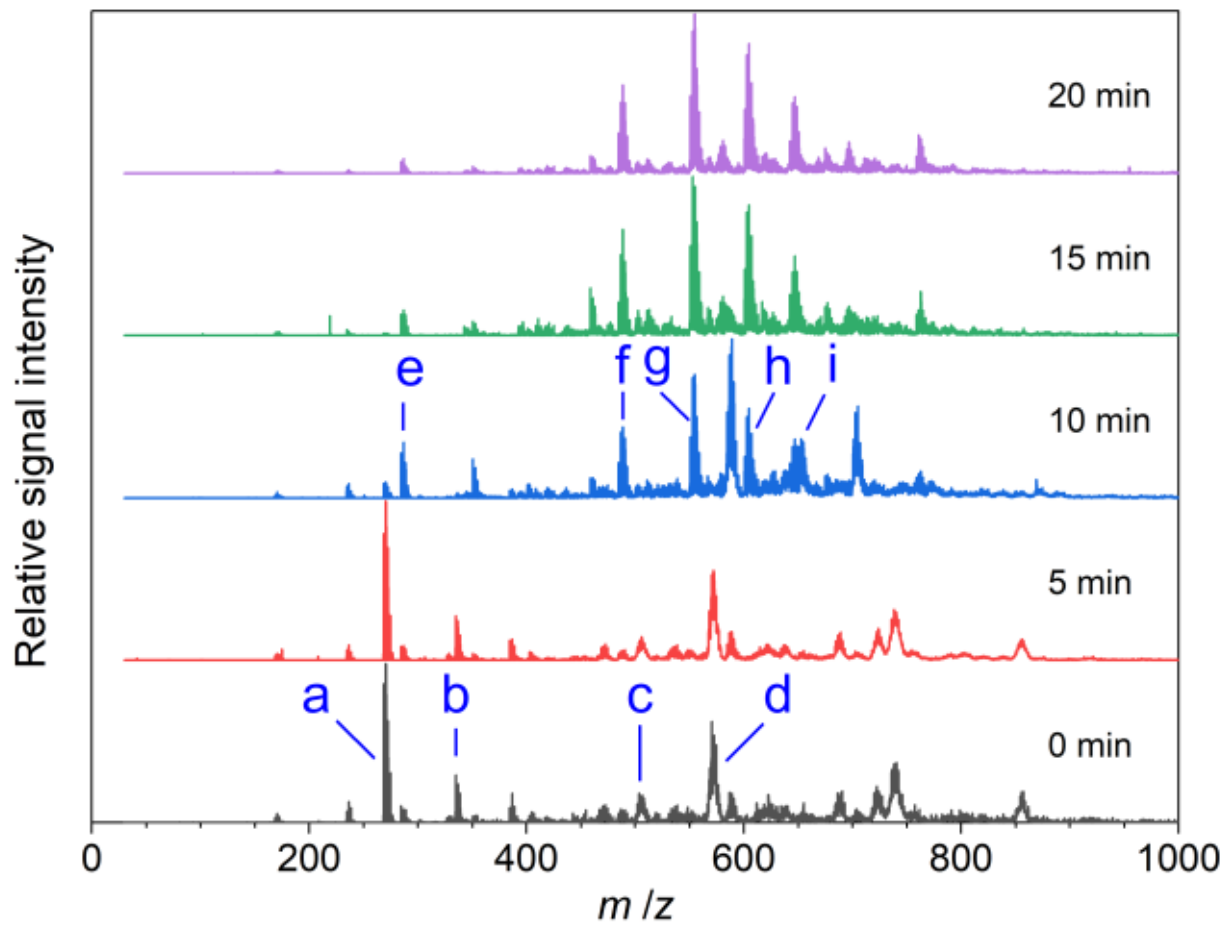

Figure S39. Negative-ion mode ESI-mass spectra of solutions of (R^{Ph})Zn(OPiv)·Li(OPiv)·LiCl in THF (0.038 M) upon exposure to ambient atmosphere for different times, a = (R^{Ph})$ZnCl_2^-$, b = (R^{Ph})Zn(OPiv)Cl^-, c = $(R^{Ph})_2Zn_2Cl_3^-$, d = $(R^{Ph})_2Zn_2$(OPiv)Cl_2^-, e = Zn(OR^{Ph})Cl_2^-, f = Zn_2(OR^{Ph})(OPiv)Cl_3^-, g = Zn_2(OR^{Ph})$(OPiv)_2Cl_2^-$, h = $Zn_2(OR^{Ph})_2$(OPiv)Cl_2^-, i = $LiZn_2(OR^{Ph})_2$(OPiv)Cl_3^-.

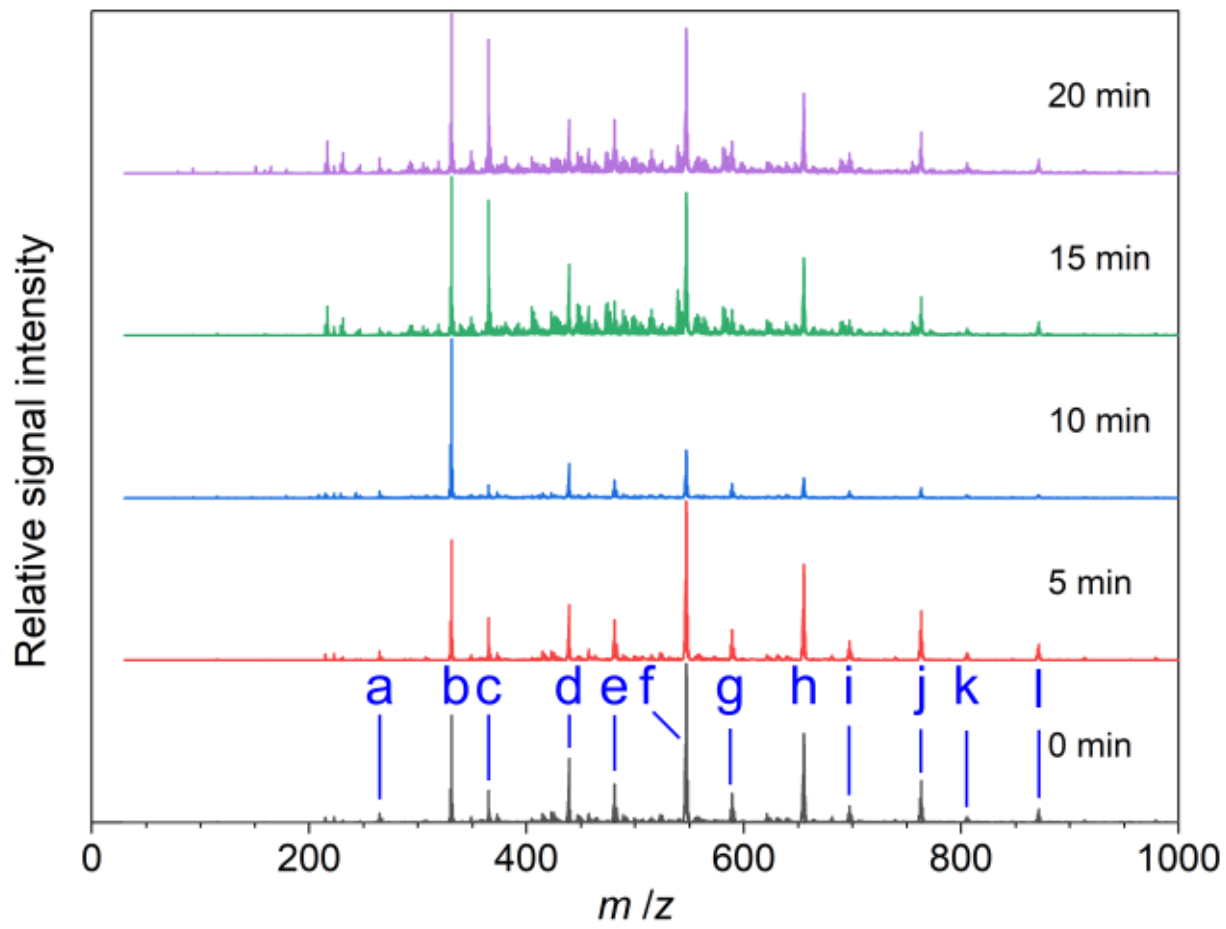

Figure S40. Positive-ion mode ESI-mass spectra of solutions of (R^{Ph})Zn(OPiv)·Li(OPiv)·LiCl in THF (0.038 M) upon exposure to ambient atmosphere for different times, a = $Li_4(OPiv)_2Cl^+$, b = $Li_4(OPiv)_3^+$, c = $Li((R^{Ph})_2O)(THF)^+$, d = $Li_5(OPiv)_4^+$, e = $Li_6(OPiv)_4Cl^+$, f = $Li_6(OPiv)_5^+$, g = $Li_7(OPiv)_5Cl^+$, h = $Li_7(OPiv)_6^+$, i = $Li_8(OPiv)_6Cl^+$, j = $Li_8(OPiv)_7^+$, k = $Li_9(OPiv)_7Cl^+$, l = $Li_9(OPiv)_8^+$.

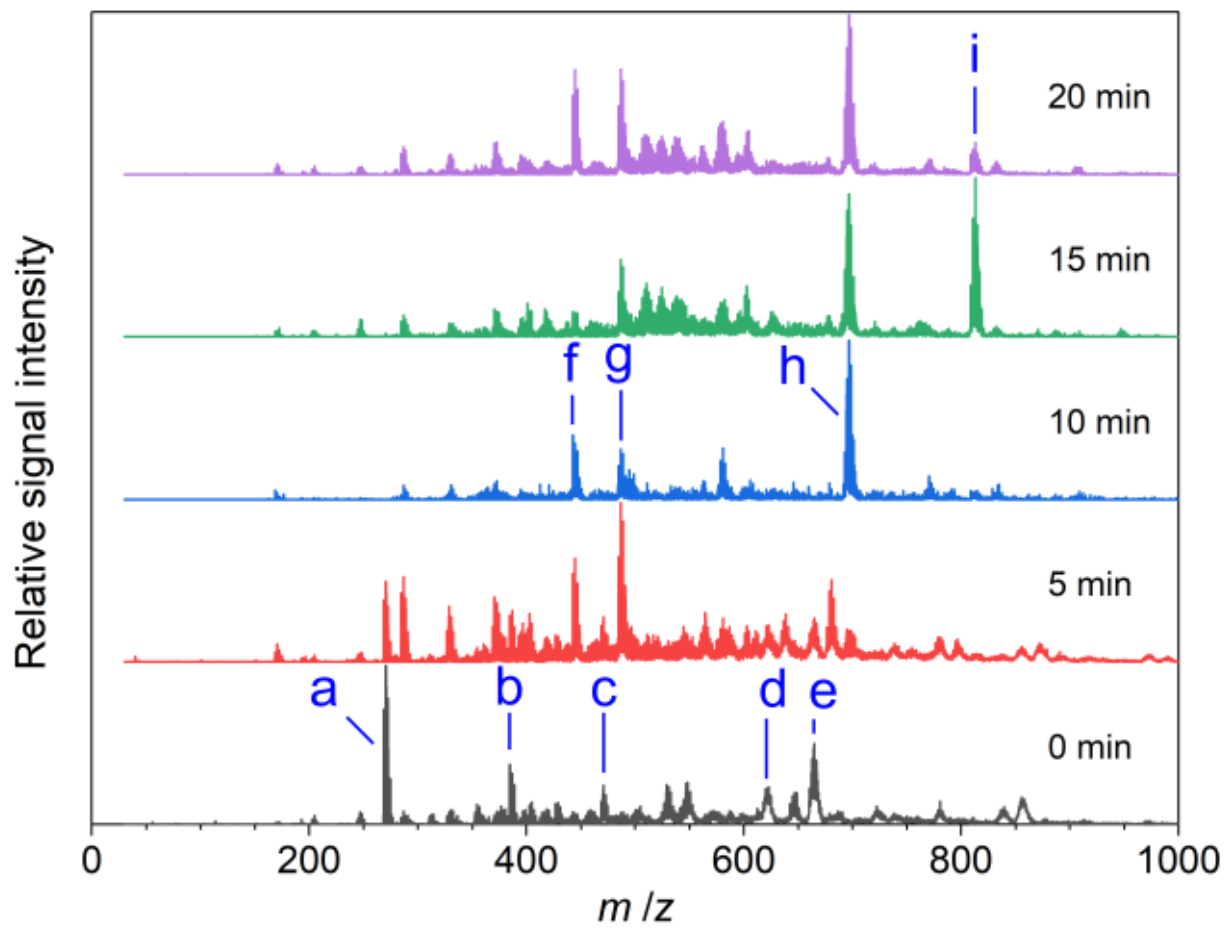

Figure S41. Negative-ion mode ESI-mass spectra of solutions of $(R^{Ph})ZnCl\cdot 2\,LiCl$ in THF (0.038 M) upon exposure to ambient atmosphere for different times, a = $(R^{Ph})ZnCl_2^-$, b = $(R^{Ph})Zn(OR^{Ph})Cl^-$, c = $Li_2(R^{Ph})Zn(OR^{Ph})Cl_3^-$, d = $(R^{Ph})_2Zn_2(OR^{Ph})Cl_2^-$, e = $Li(R^{Ph})_2Zn_2(OR^{Ph})Cl_3^-$, f = $LiZn(OR^{Ph})_2Cl_2^-$, g = $Li_2Zn(OR^{Ph})_2Cl_3^-$, h = $LiZn_2(OR^{Ph})_3Cl_3^-$, i = $LiZn_2(OR^{Ph})_4Cl_2^-$.

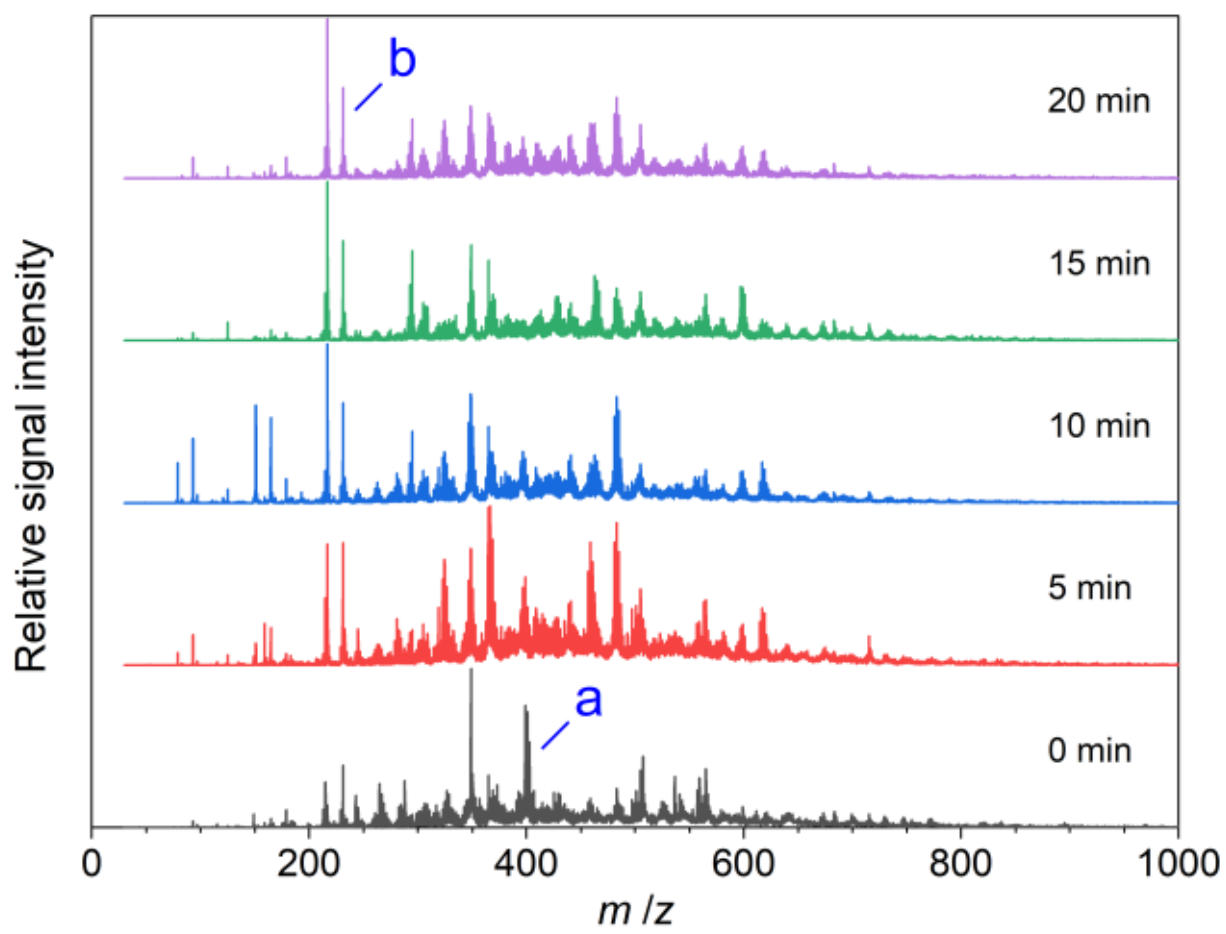

Figure S42. Positive-ion mode ESI-mass spectra of solutions of $(R^{Ph})ZnCl\cdot 2\,LiCl$ in THF (0.038 M) upon exposure to ambient atmosphere for different times, a = $Li_2(R^{Ph})Zn(OR^{Ph})Cl^+$, b = $Li(HOR^{Ph})(THF)^+$.

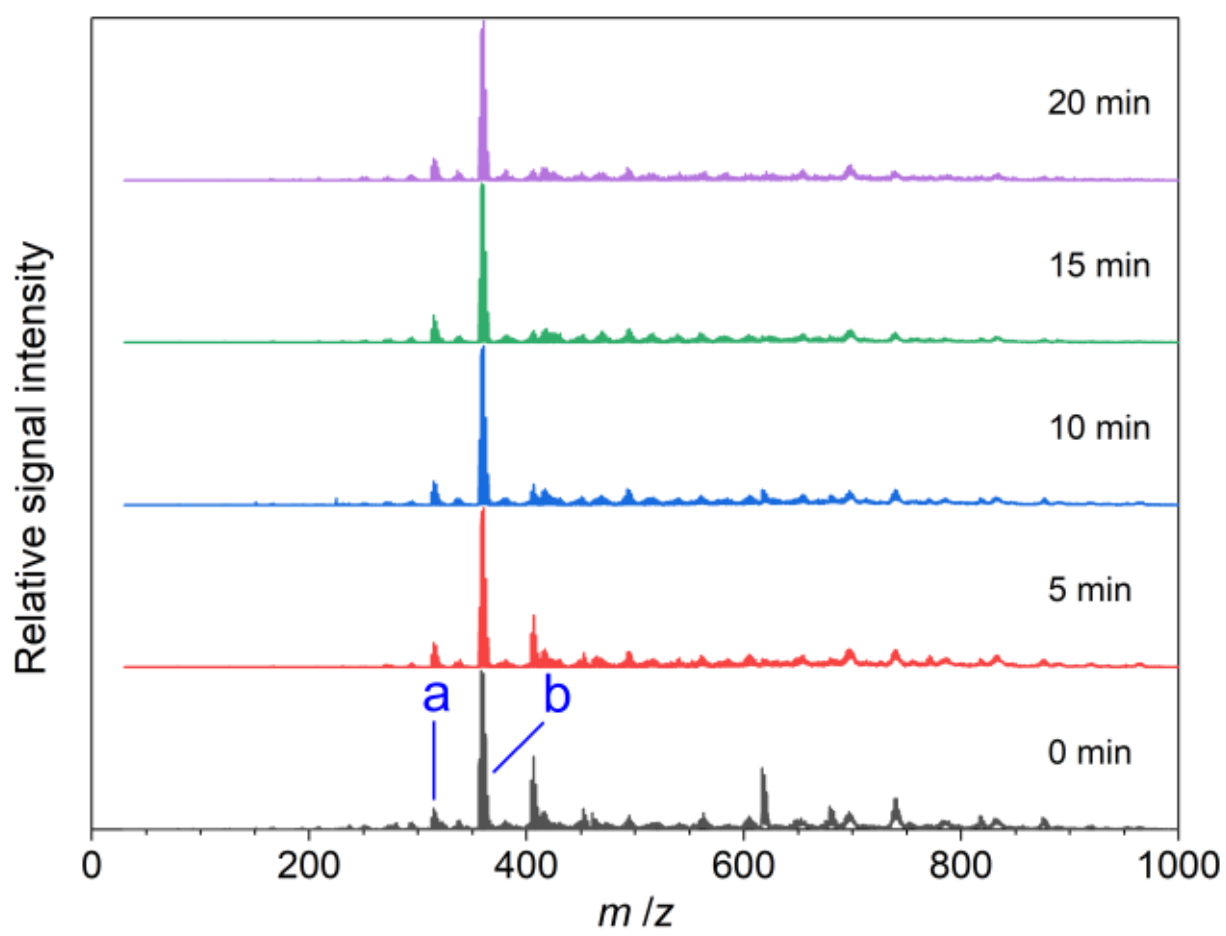

Figure S43. Negative-ion mode ESI-mass spectra of solutions of $(R^{Ph})ZnBr\cdot LiCl\cdot LiBr$ in THF (0.038 M) upon exposure to ambient atmosphere for different times, a = $(R^{Ph})ZnClBr^-$, b = $(R^{Ph})ZnBr_2^-$.

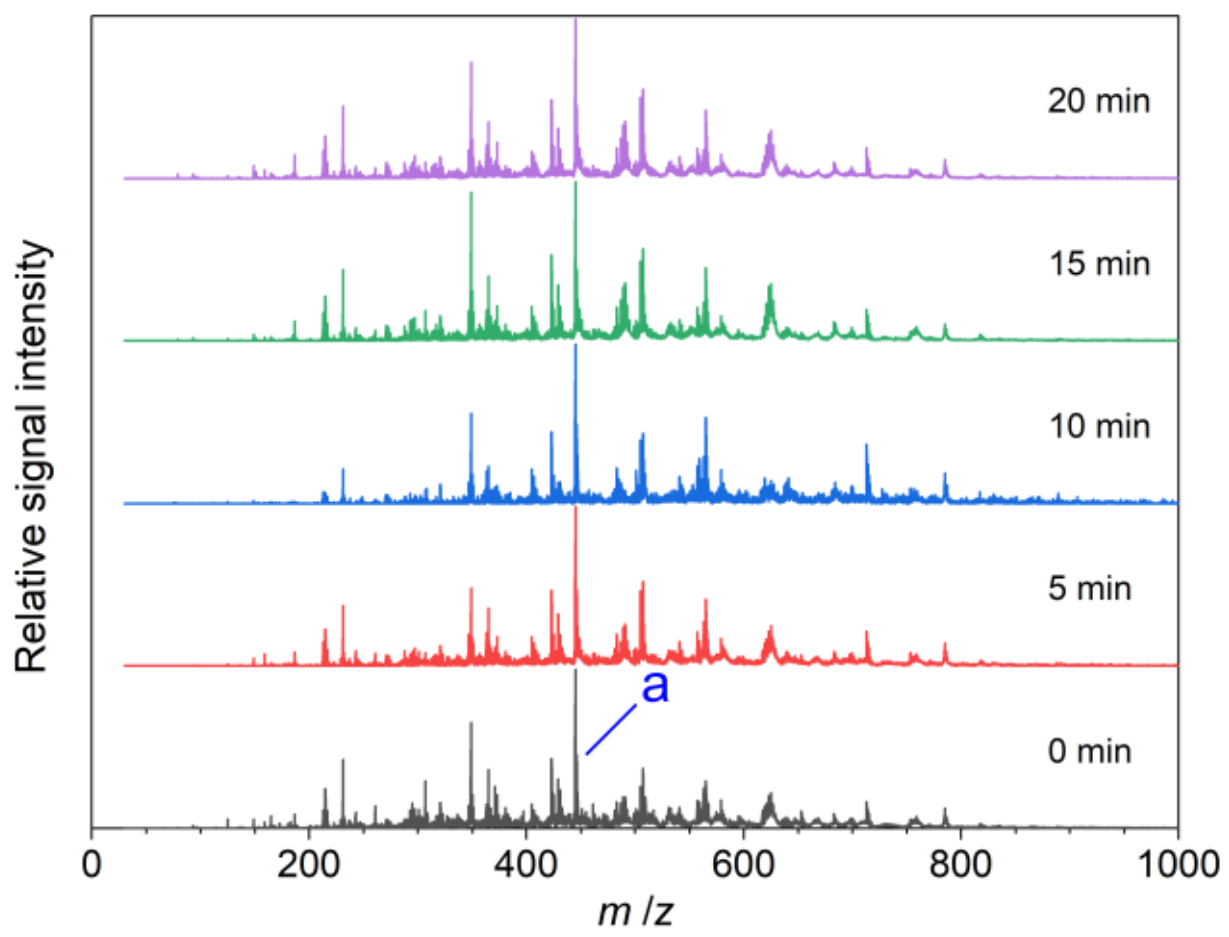

Figure S44. Positive-ion mode ESI-mass spectra of solutions of $(R^{Ph})ZnBr{\cdot}LiCl{\cdot}LiBr$ in THF (0.038 M) upon exposure to ambient atmosphere for different times, a = $Li(HOR^{Ph})(OR^{Ph}{}_2)^+$.

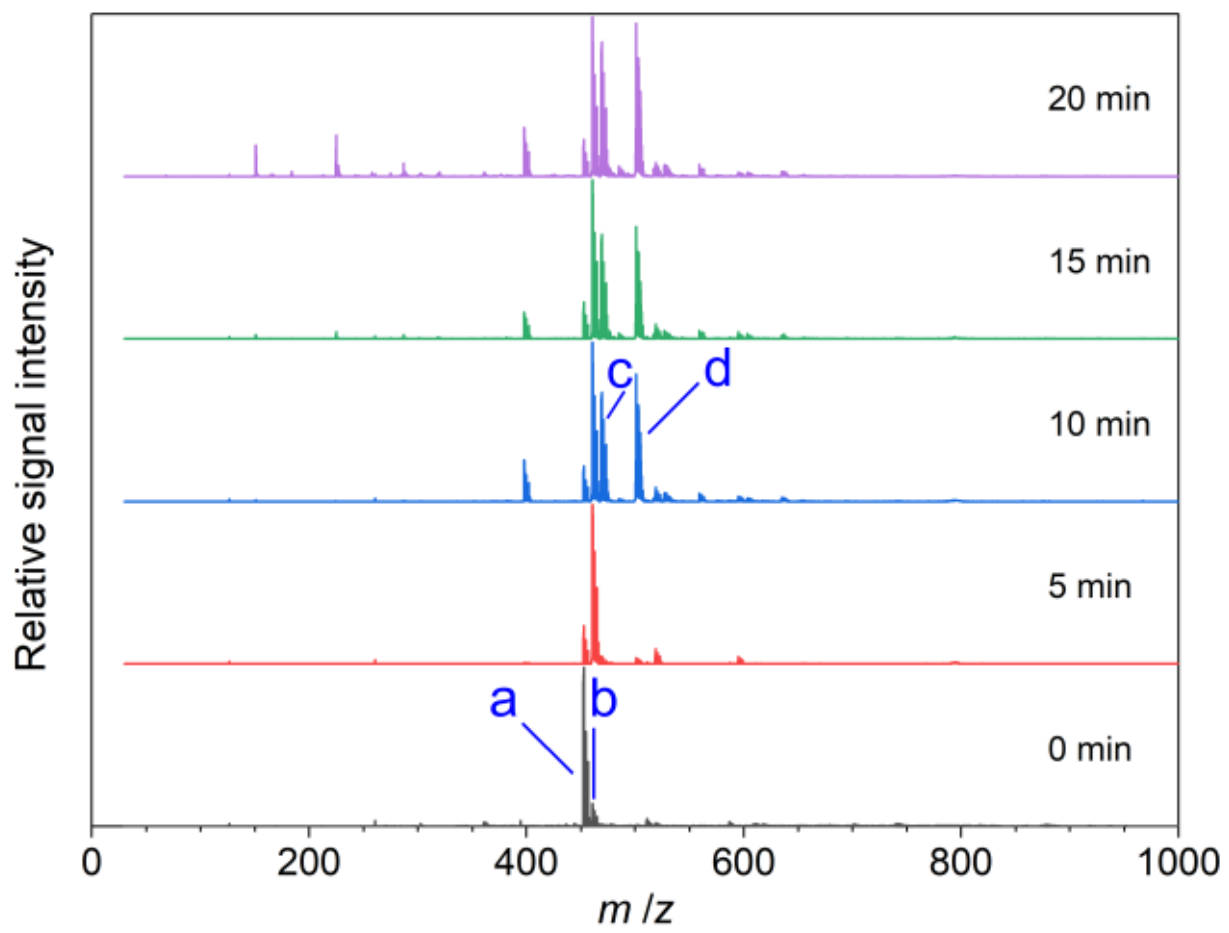

Figure S45. Negative-ion mode ESI-mass spectra of solutions of $(R^{Ph})ZnI{\cdot}LiCl{\cdot}LiI$ in THF (0.038 M) upon exposure to ambient atmosphere for different times, a = $(R^{Ph})ZnI_2^-$, b = $(R^{Ph})_2ZnI^-$, c = $(R^{Ph})_3Zn^-$, d = $Zn(OR^{Ph})_3^-$.

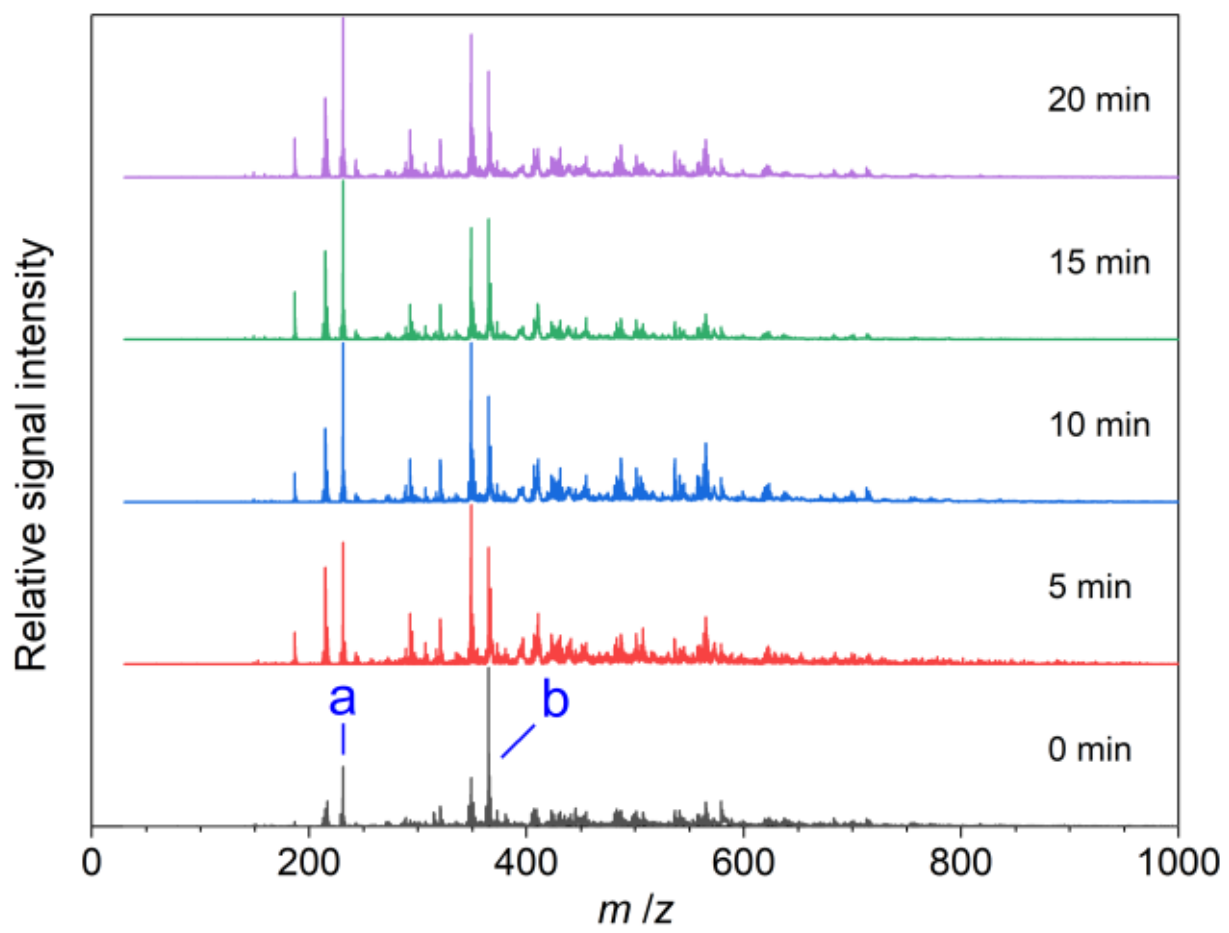

Figure S46. Positive-ion mode ESI-mass spectra of solutions of $(R^{Ph})ZnI{\cdot}LiCl{\cdot}LiI$ in THF (0.038 M) upon exposure to ambient atmosphere for different times, a = $Li(HOR^{Ph})(THF)^+$, b = $Li(R^{Ph})_2O(THF)^+$.

4.2 (R^{Ph2})ZnX·LiX·LiCl (X = OPiv, Cl, Br, I) systems

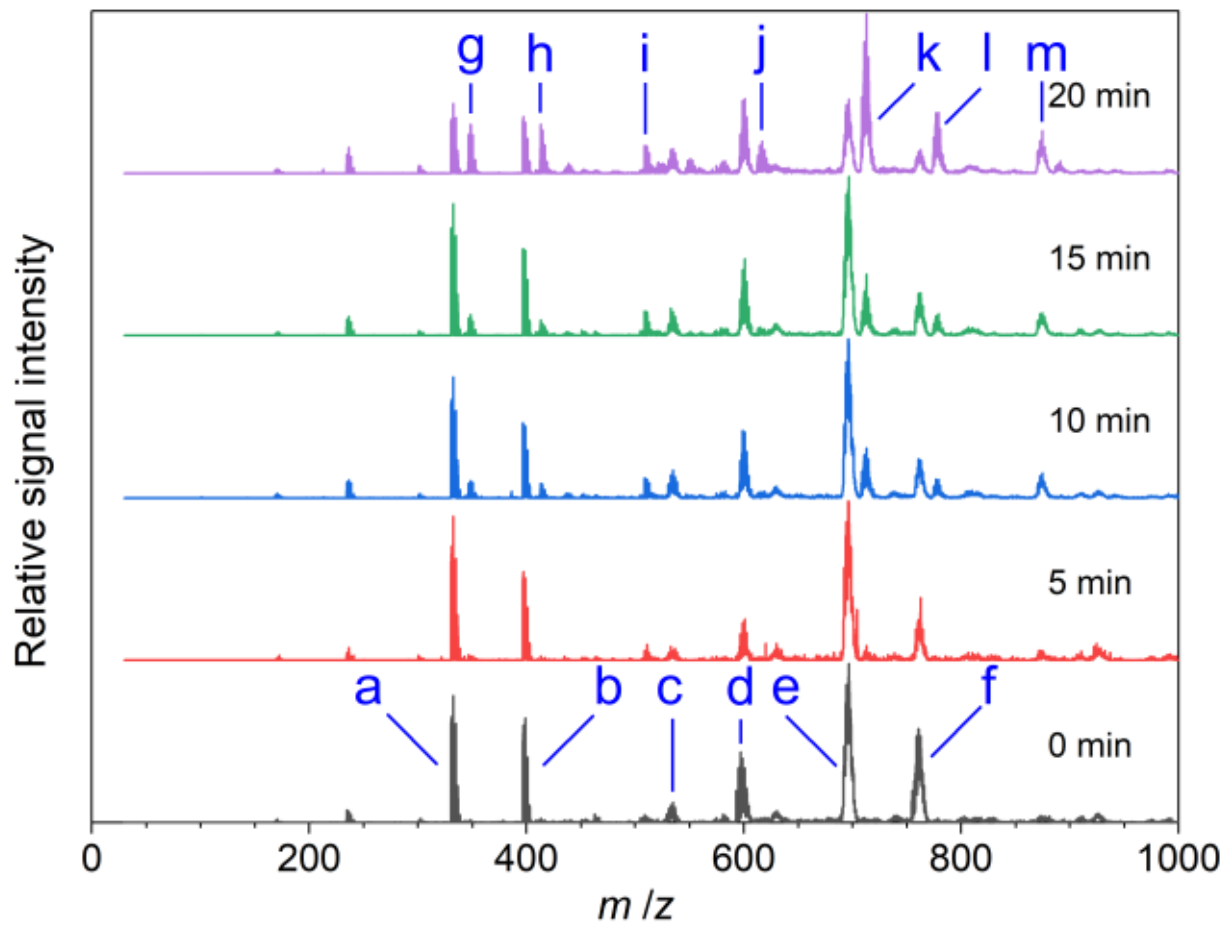

Figure S47. Negative-ion mode ESI-mass spectra of solutions of (R^{Ph2})Zn(OPiv)·Li(OPiv)·LiCl in THF (0.038 M) upon exposure to ambient atmosphere for different times, a = (R^{Ph2})$ZnCl_2^-$, b = (R^{Ph2})Zn(OPiv)Cl^-, c = (R^{Ph2})Zn_2(OPiv)Cl_3^-, d = (R^{Ph2})$Zn_2(OPiv)_2Cl_2^-$, e = $(R^{Ph2})_2Zn_2$(OPiv)Cl_2^-, f = $(R^{Ph2})_2Zn_2(OPiv)_2Cl^-$, g = Zn($OR^{Ph2}$)$Cl_2^-$, h = Zn($OR^{Ph2}$)(OPiv)$Cl^-$, i = ($R^{Ph2}$)Zn($OR^{Ph2}$)$Cl^-$, j = Zn_2(OR^{Ph2})$(OPiv)_2Cl_2^-$, k = (R^{Ph2})Zn_2(OR^{Ph2})(OPiv)Cl_2^-, l = (R^{Ph2})Zn_2(OR^{Ph2})$(OPiv)_2Cl^-$, m = $(R^{Ph2})_2Zn_2$(OR^{Ph2})(OPiv)Cl^-.

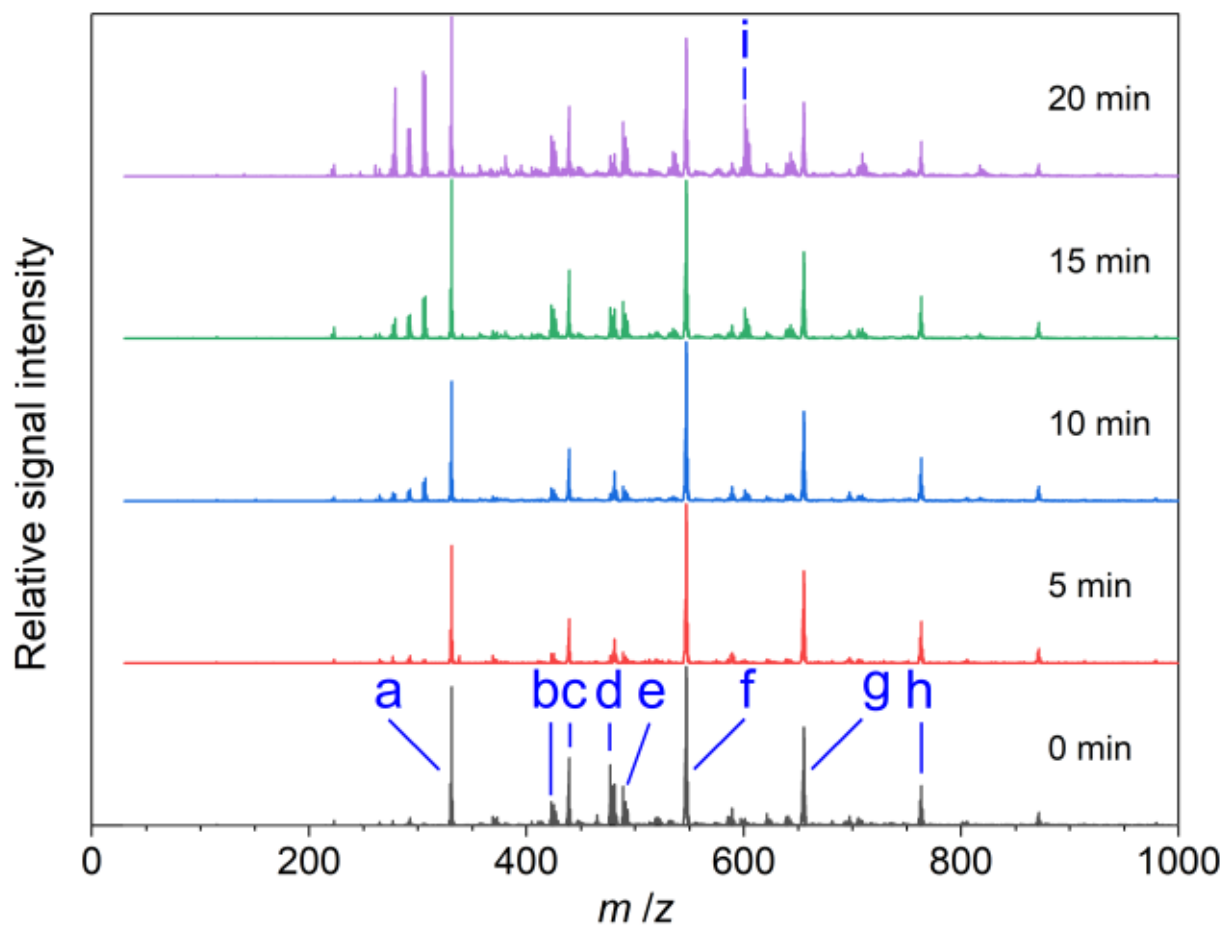

Figure S48. Positive-ion mode ESI-mass spectra of solutions of (R^{Ph2})Zn(OPiv)·Li(OPiv)·LiCl in THF (0.038 M) upon exposure to ambient atmosphere for different times, a = $Li_4(OPiv)_3^+$, b = $Li_3Zn(OPiv)_3Cl^+$, c = $Li_5(OPiv)_4^+$, d = Li_2(R^{Ph2})$Zn(OPiv)_2^+$, e = $Li_3Zn(OPiv)_4^+$, f = $Li_6(OPiv)_5^+$, g = $Li_7(OPiv)_6^+$, h = $Li_8(OPiv)_7^+$, i = Li_3Zn(OR^{Ph2})$(OPiv)_3^+$.

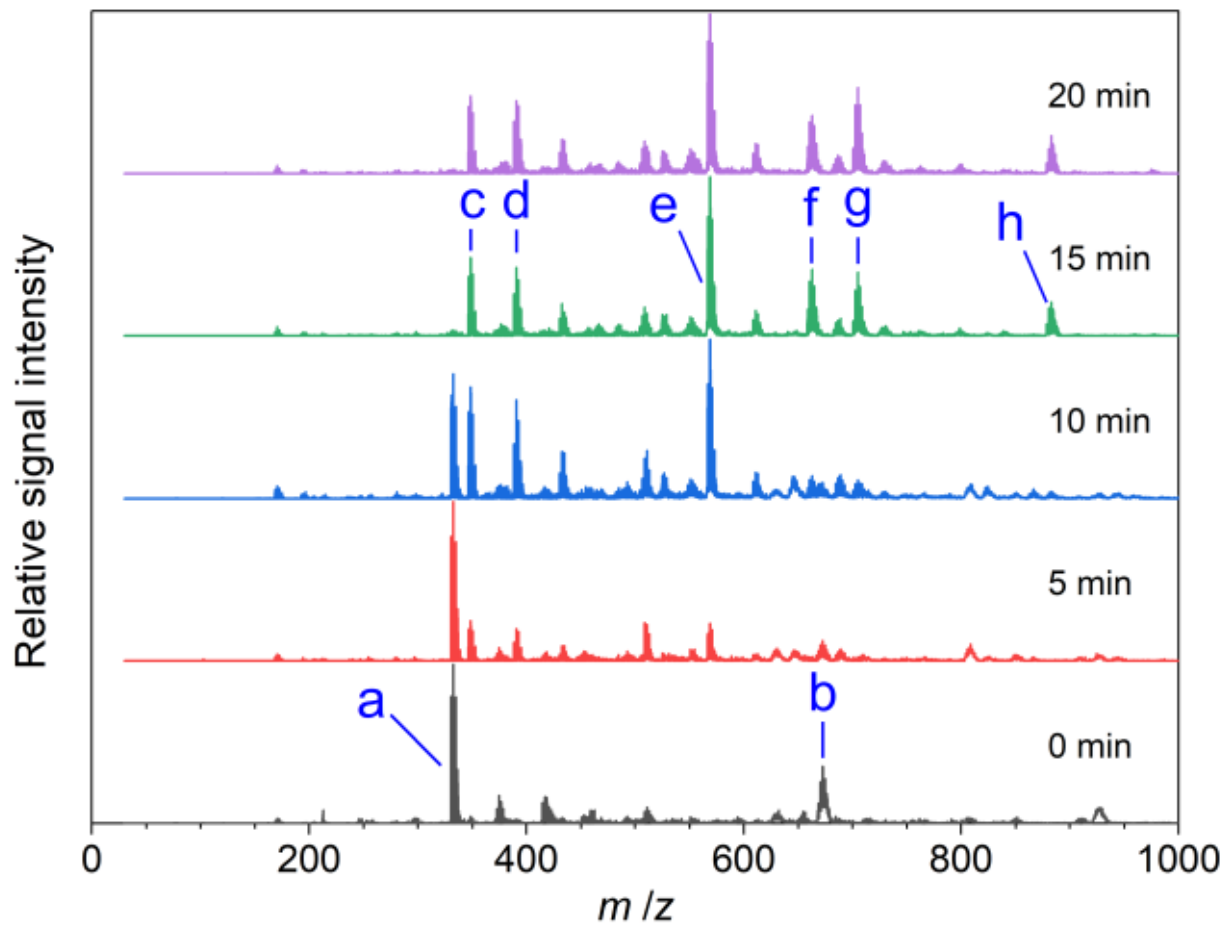

Figure S49. Negative-ion mode ESI-mass spectra of solutions of $(R^{Ph2})ZnCl{\cdot}2\,LiCl$ in THF (0.038 M) upon exposure to ambient atmosphere for different times, a = $(R^{Ph2})ZnCl_2^-$, b = $Li(R^{Ph2})_2Zn_2Cl_4^-$, c = $Zn(OR^{Ph2})Cl_2^-$, d = $LiZn(OR^{Ph2})Cl_3^-$, e = $Li(OR^{Ph2})_2ZnCl_2^-$, f = $Zn_2(OR^{Ph2})_2Cl_3^-$, g = $LiZn_2(OR^{Ph2})_2Cl_4^-$, h = $LiZn_2(OR^{Ph2})_3Cl_3^-$.

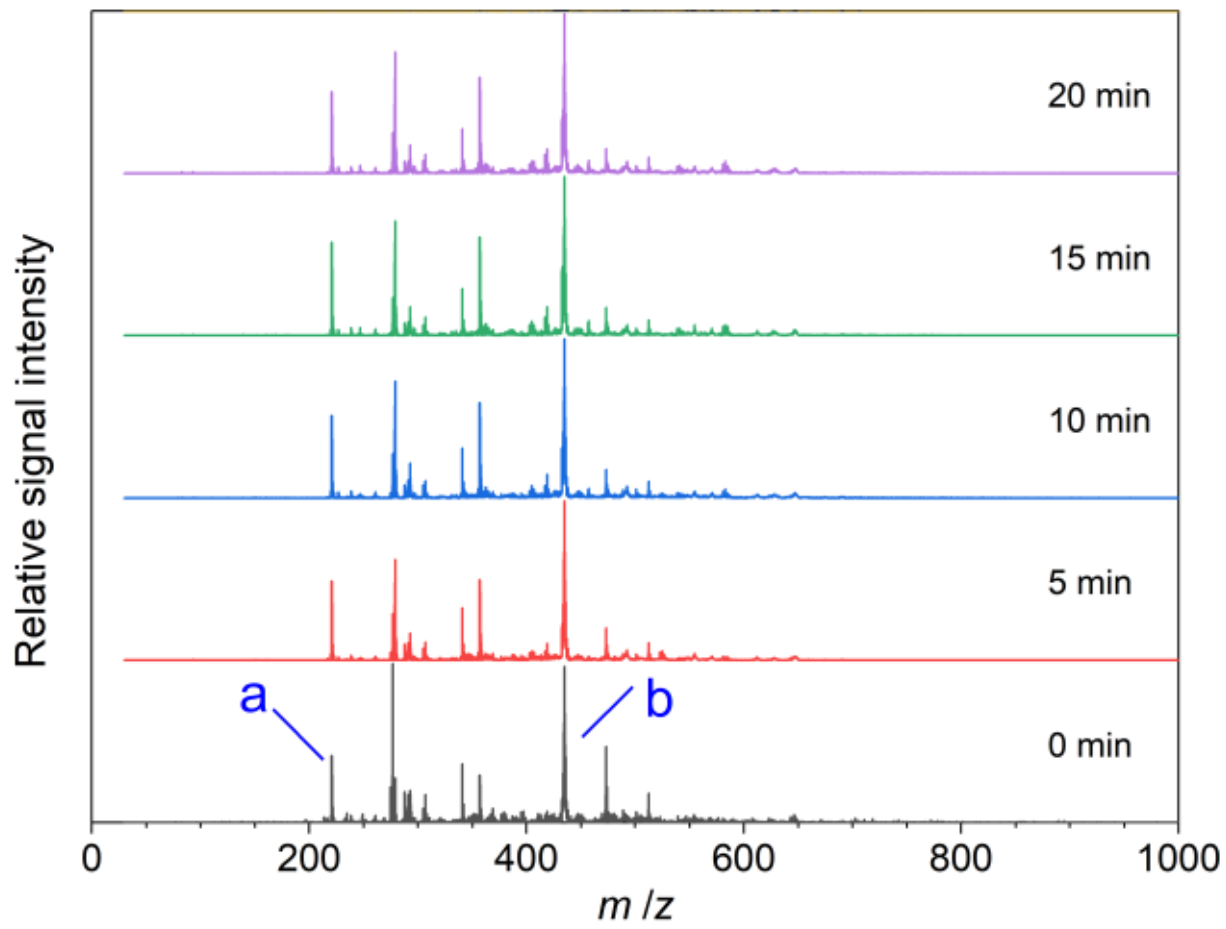

Figure S50. Positive-ion mode ESI-mass spectra of solutions of $(R^{Ph2})ZnCl{\cdot}2\,LiCl$ in THF (0.038 M) upon exposure to ambient atmosphere for different times, a = $Li(HOR^{Ph2})^+$, b = $Li(HOR^{Ph2})_2^+$.

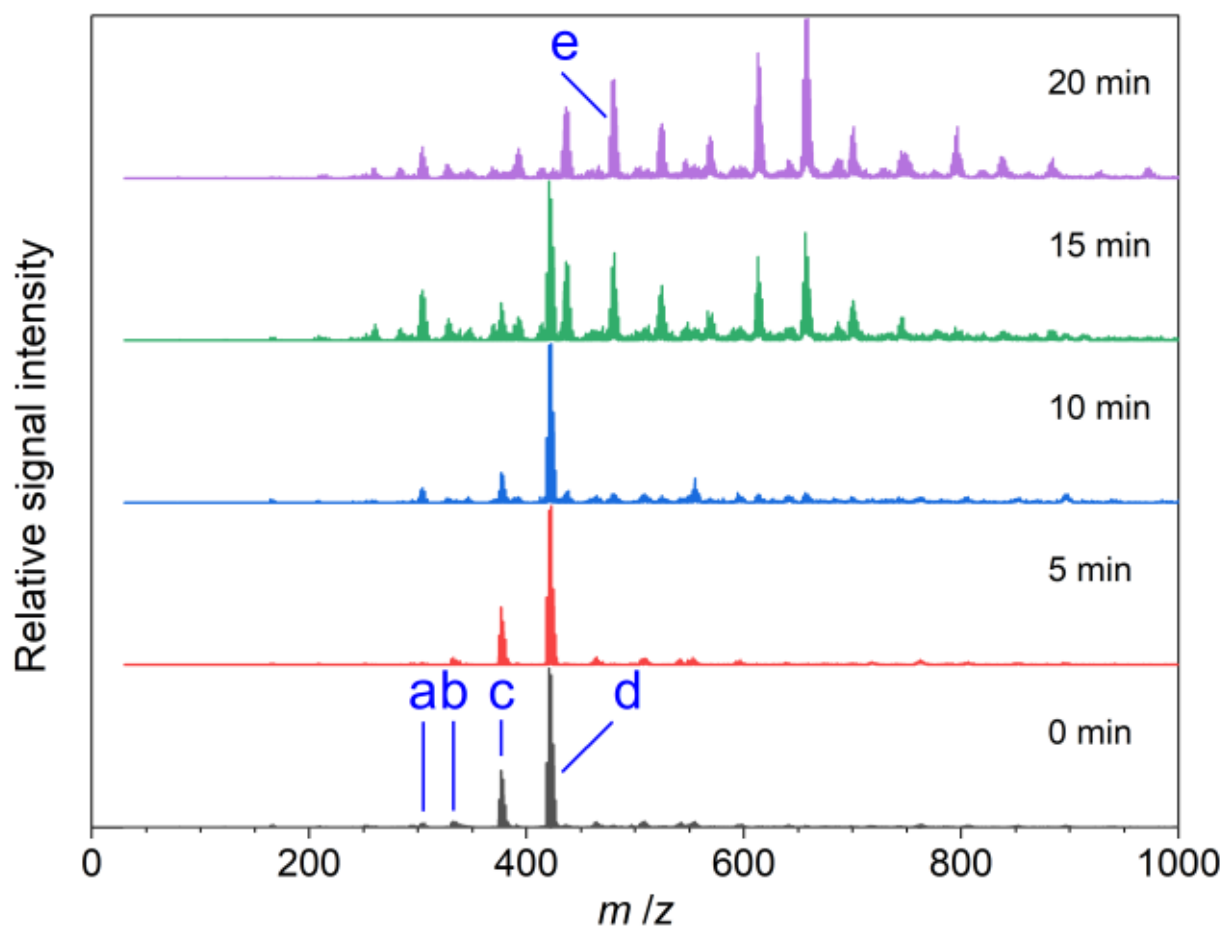

Figure S51. Negative-ion mode ESI-mass spectra of solutions of $(R^{Ph2})ZnBr{\cdot}LiCl{\cdot}LiBr$ in THF (0.038 M) upon exposure to ambient atmosphere for different times, a = $ZnBr_3^-$, b = $(R^{Ph2})ZnCl_2^-$, c = $(R^{Ph2})ZnClBr^-$, d = $(R^{Ph2})ZnBr_2^-$, e = $LiZn(OR^{Ph2})ClBr_2^-$.

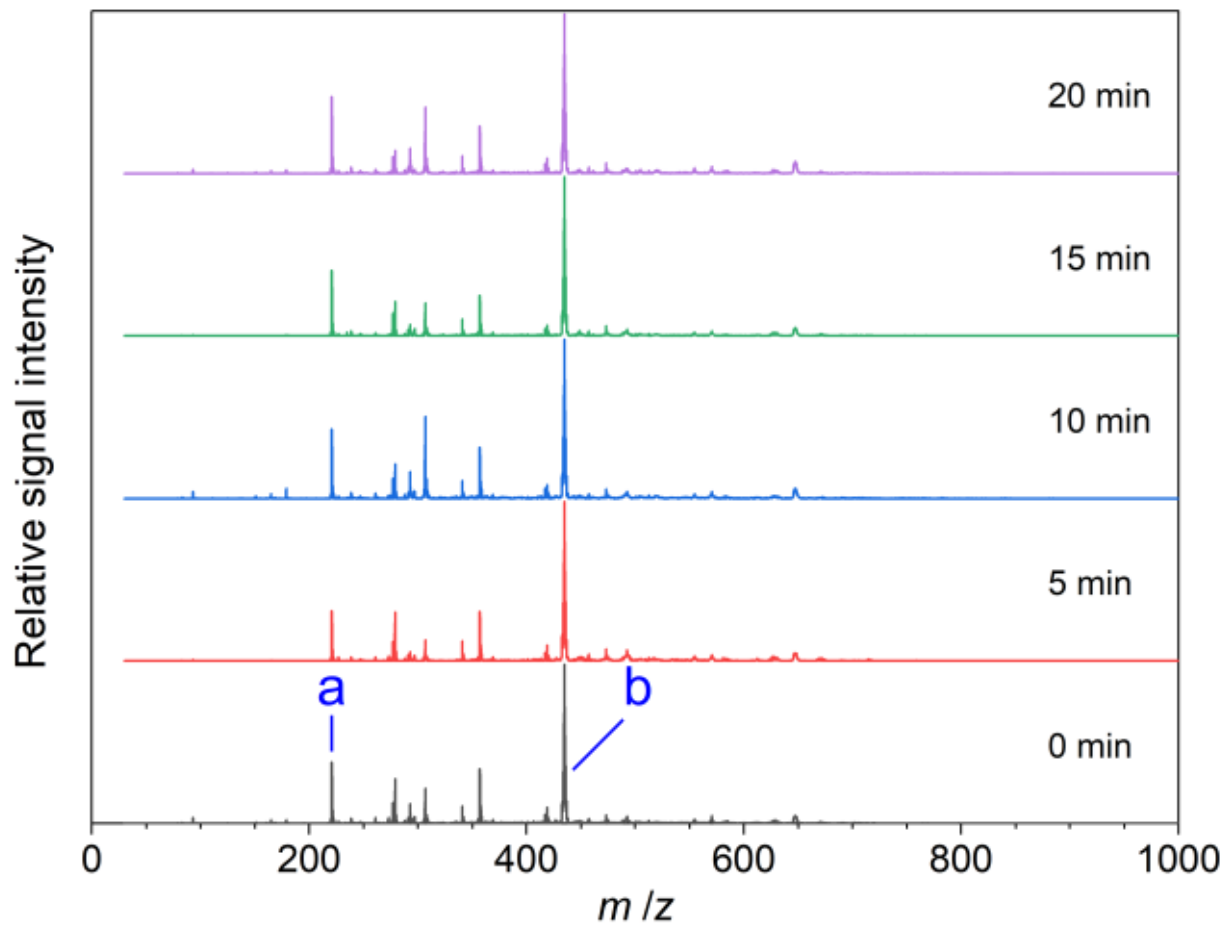

Figure S52. Positive-ion mode ESI-mass spectra of solutions of (R^{Ph2})ZnBr·LiCl·LiBr in THF (0.038 M) upon exposure to ambient atmosphere for different times, a = Li(HOR^{Ph2})$^+$, b = Li(HOR^{Ph2})$_2^+$.

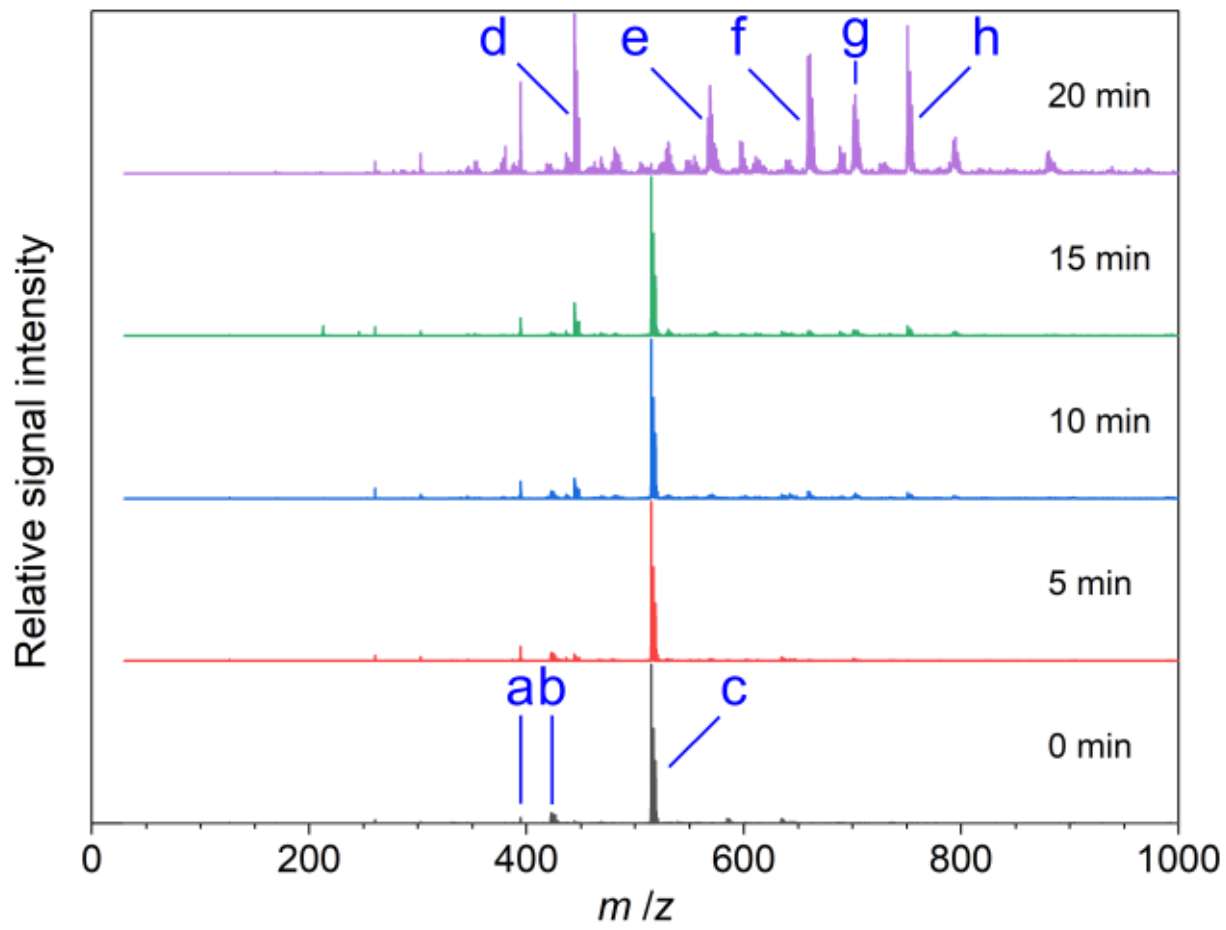

Figure S53. Negative-ion mode ESI-mass spectra of solutions of (R^{Ph2})ZnI·LiCl·LiI in THF (0.038 M) upon exposure to ambient atmosphere for different times, a = $Li_2I_3^-$, b = (R^{Ph2})ZnClI$^-$, c = (R^{Ph2})ZnI_2^-, d = ZnI_3^-, e = LiZn(OR^{Ph2})$_2Cl_2^-$, f = LiZn(OR^{Ph2})$_2$ClI$^-$, g = Li_2Zn(OR^{Ph2})$_2Cl_2I^-$, h = LiZn(OR^{Ph2})$_2I_2^-$.

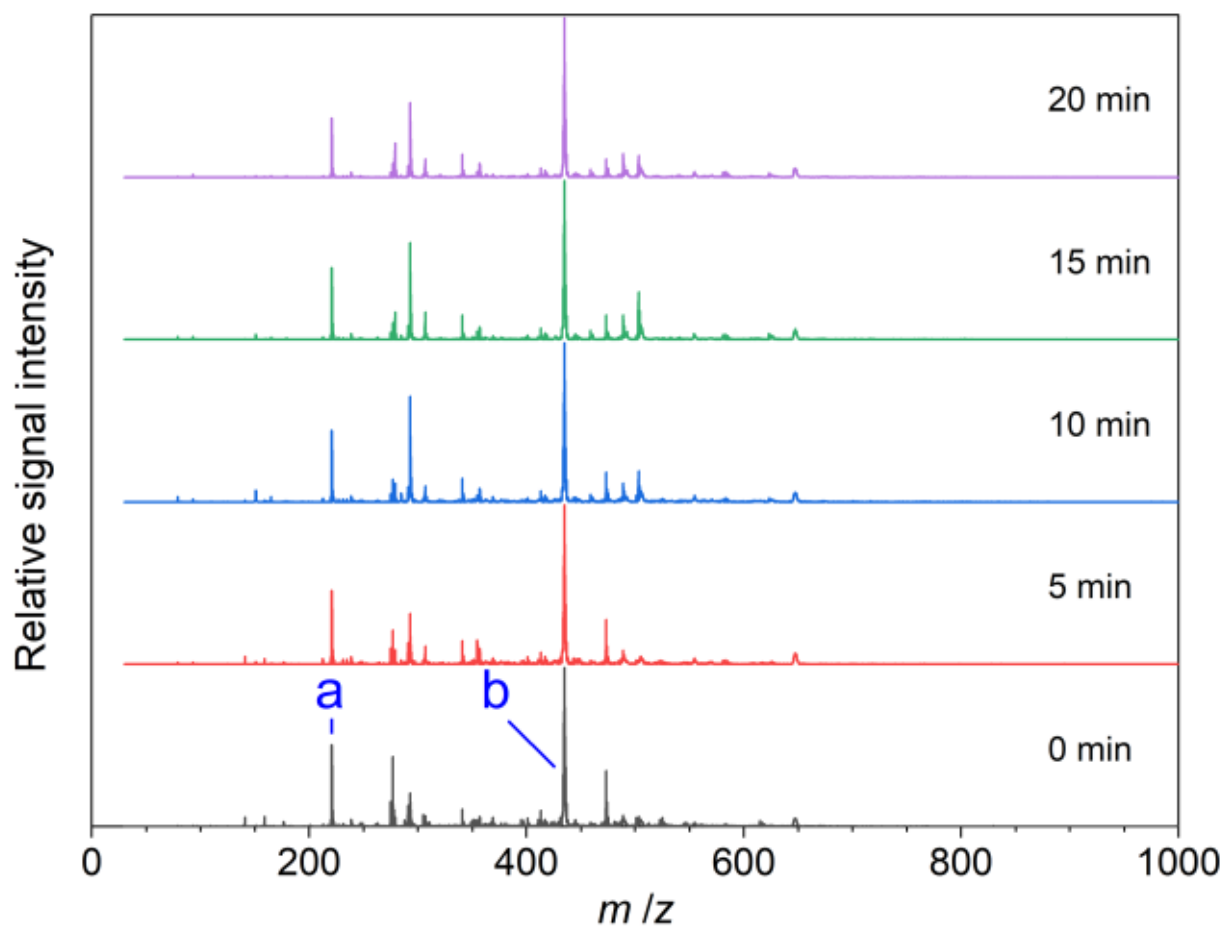

Figure S54. Positive-ion mode ESI-mass spectra of solutions of (R^{Ph2})ZnI·LiCl·LiI in THF (0.038 M) upon exposure to ambient atmosphere for different times, a = Li(HOR^{Ph2})$^+$, b = Li(HOR^{Ph2})$_2^+$.

4.3 (R^{Ph3})ZnX·LiX·LiCl (X = OPiv, Cl, Br, I, OAc) systems

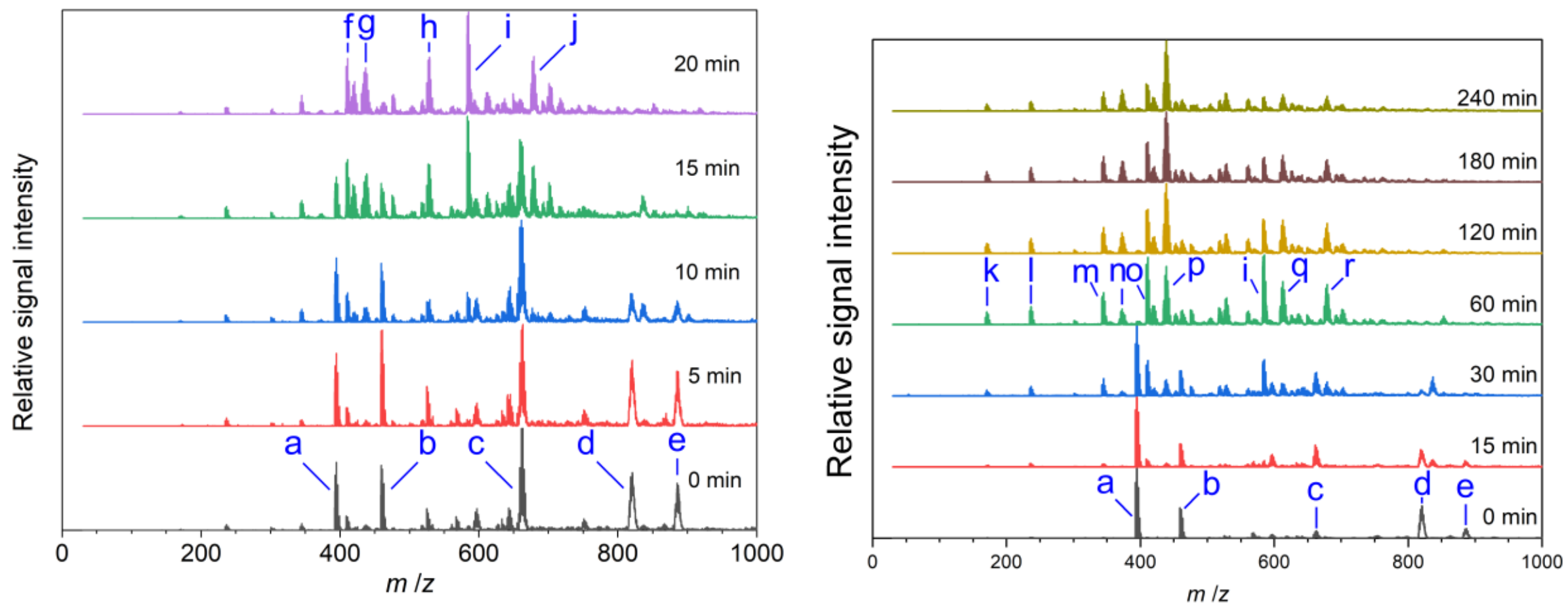

Figure S55. Negative-ion mode ESI-mass spectra of solutions of (R^{Ph3})Zn(OPiv)·Li(OPiv)·LiCl in THF at 0.038 M (left) and 0.16 M (right), upon exposure to ambient atmosphere for different times, a = $(R^{Ph3})ZnCl_2^-$, b = $(R^{Ph3})Zn(OPiv)Cl^-$, c = $(R^{Ph3})Zn_2(OPiv)_2Cl_2^-$, d = $(R^{Ph3})_2Zn_2(OPiv)Cl_2^-$, e = $(R^{Ph3})_2Zn_2(OPiv)_2Cl^-$, f = $Zn(OR^{Ph3})Cl_2^-$, g = $Zn_3(OPiv)(OH)_4Cl_2^-$, h = $LiZn_2(OPiv)_3(OH)Cl_2^-$, i = $LiZn(OR^{Ph3})(OPiv)_2Cl^-$, j = $LiZn_2(OPiv)_5Cl^-$, k = $ZnCl_3^-$, l = $Zn(OPiv)Cl_2^-$, m = $LiZn(OPiv)_2Cl_2^-$, n = $Zn_2(OPiv)Cl_4^-$, o = $LiZn(OPiv)_3Cl^-$, p = $Zn_2(OPiv)_2Cl_3^-$, q = $Zn_2(OR^{Ph3})(OPiv)Cl_3^-$, r = $Zn_2(OR^{Ph3})(OPiv)_2Cl_2^-$.

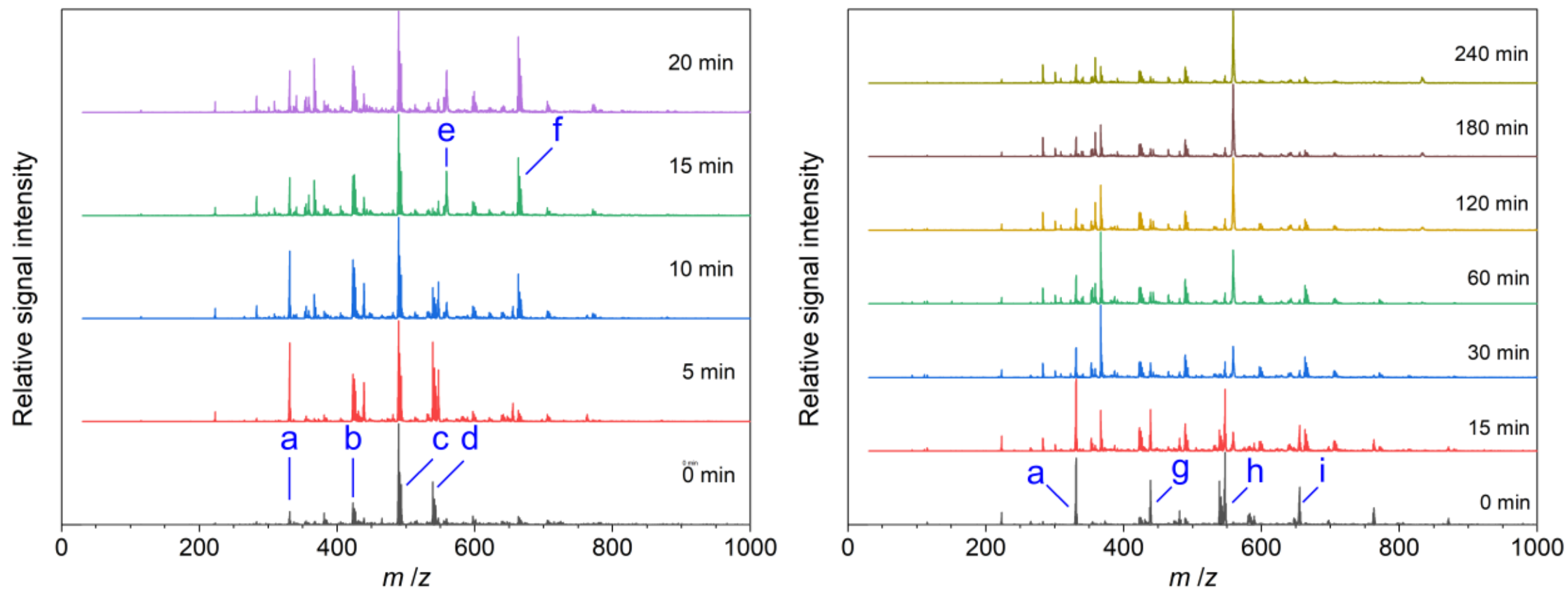

Figure S56. Positive-ion mode ESI-mass spectra of solutions of (R^{Ph3})Zn(OPiv)·Li(OPiv)·LiCl in THF at 0.038 M (left) and 0.16 M (right), upon exposure to ambient atmosphere for different times, a = $Li_4(OPiv)_3^+$, b = $Li_3Zn(OPiv)_3Cl^+$, c = $Li_2Zn(OPiv)_3(THF)(H_2O)_2^+$, d = $Li(R^{Ph3})Zn(OPiv)(THF)(H_2O)_2^+$, e = $Li(HOR^{Ph3})_2^+$, f = $Li_6Zn(OPiv)_5(OH)_2(H_2O)^+$, g = $Li_5(OPiv)_4^+$, h = $Li_6(OPiv)_5^+$, i = $Li_7(OPiv)_6^+$.

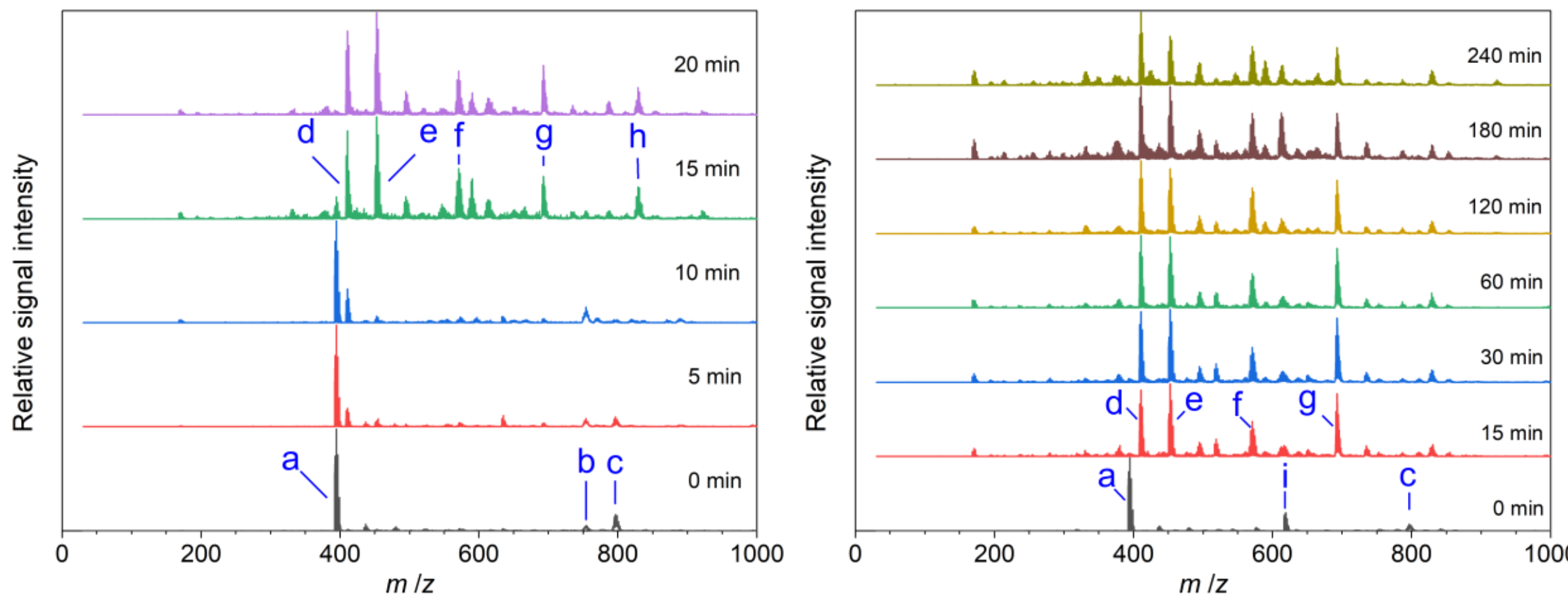

Figure S57. Negative-ion mode ESI-mass spectra of solutions of $(R^{Ph3})ZnCl \cdot 2\,LiCl$ in THF at 0.038 M (left) and 0.16 M (right), upon exposure to ambient atmosphere for different times, a = $(R^{Ph3})ZnCl_2^-$, b = $(R^{Ph3})_2Zn_2Cl_3^-$, c = $Li(R^{Ph3})_2Zn_2Cl_4^-$, d = $Zn(OR^{Ph3})Cl_2^-$, e = $LiZn(OR^{Ph3})Cl_3^-$, f = $Li(OH)(OR^{Ph3})Zn_2Cl_4^-$, g = $LiZn(OR^{Ph3})_2Cl_2^-$, h = $LiZn_2(OR^{Ph3})_2Cl_4^-$, i = $(R^{Ph3})_2ZnCl^-$.

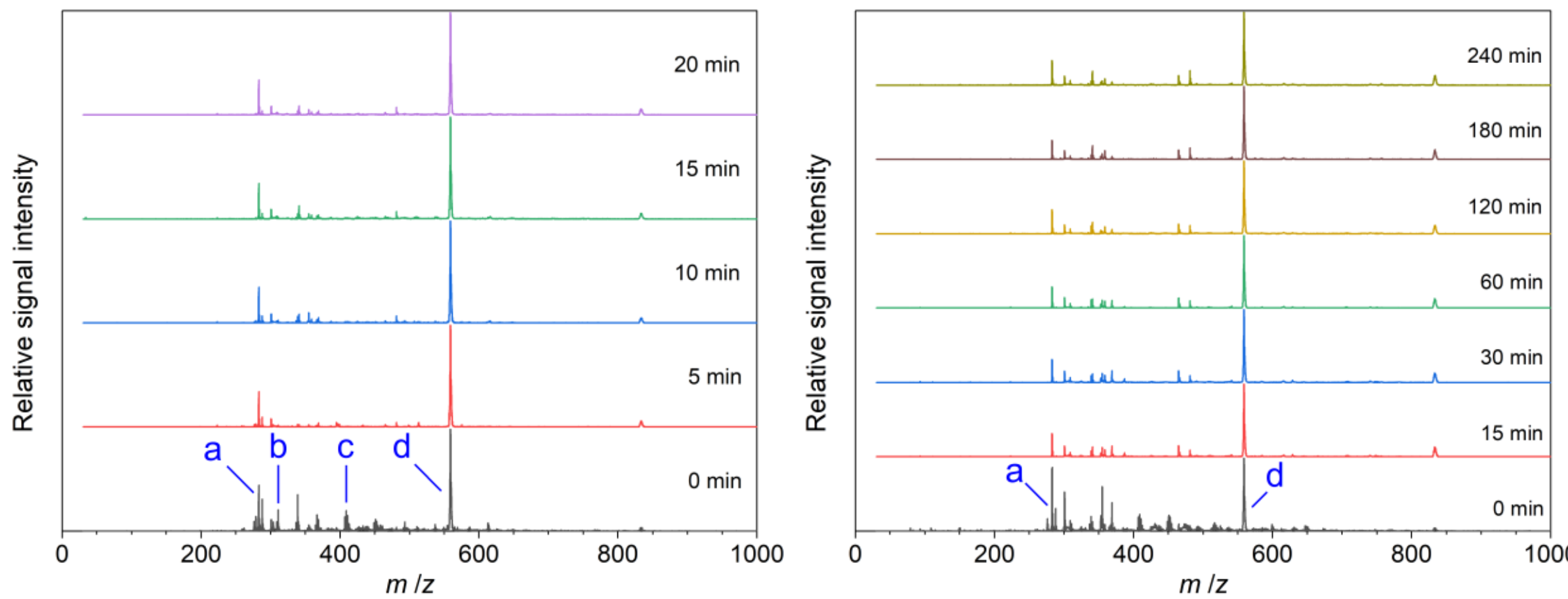

Figure S58. Positive-ion mode ESI-mass spectra of solutions of $(R^{Ph3})ZnCl \cdot 2\,LiCl$ in THF at 0.038 M (left) and 0.16 M (right), upon exposure to ambient atmosphere for different times, a = $Li(HOR^{Ph3})^+$, b = $Li(HOR^{Ph3})(H_2O)^+$, c = $Li_2(R^{Ph3})ZnCl_2^+$, d = $Li(HOR^{Ph3})_2^+$.

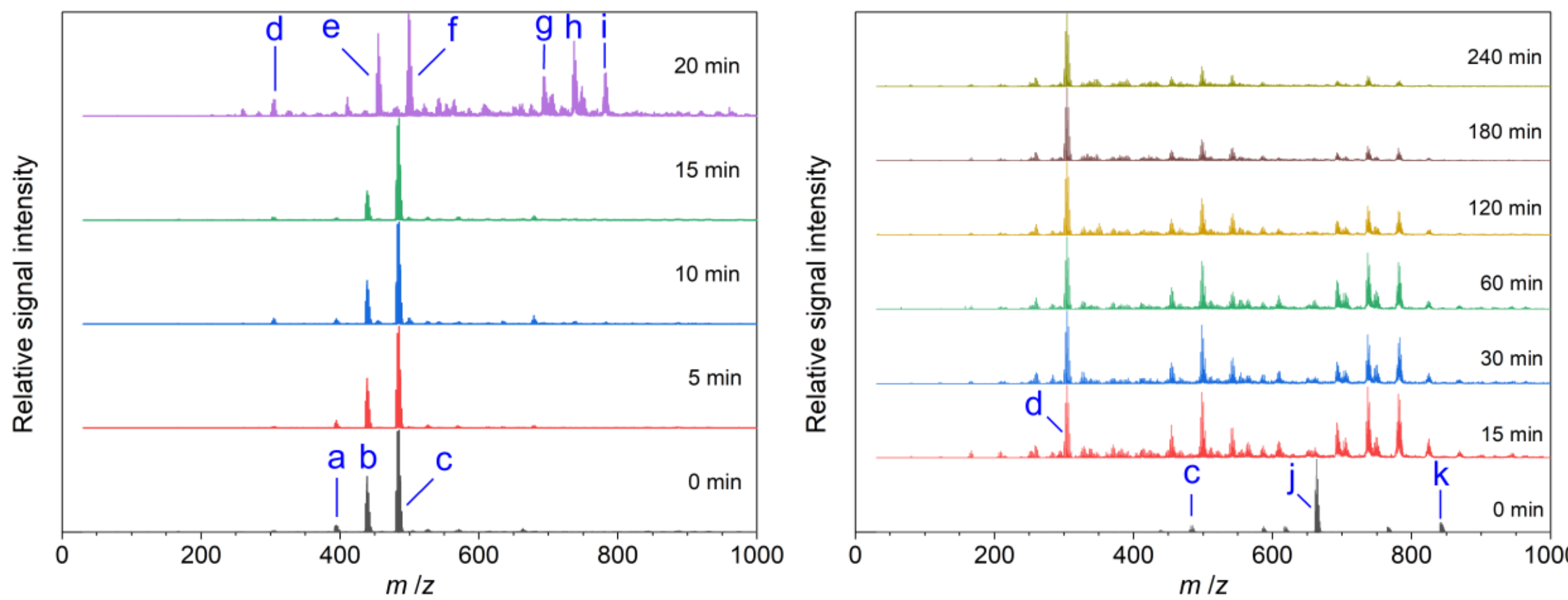

Figure S59. Negative-ion mode ESI-mass spectra of solutions of $(R^{Ph3})ZnBr \cdot LiCl \cdot LiBr$ in THF at 0.038 M (left) and 0.16 M (right), upon exposure to ambient atmosphere for different times, a = $(R^{Ph3})ZnCl_2^-$, b = $(R^{Ph3})ZnClBr^-$, c = $(R^{Ph3})ZnBr_2^-$, d = $ZnBr_3^-$, e = $Zn(OR^{Ph3})ClBr^-$, f = $Zn(OR^{Ph3})Br_2^-$, g = $LiZn(OR^{Ph3})_2Cl_2^-$, h = $LiZn(OR^{Ph3})_2ClBr^-$, i = $LiZn(OR^{Ph3})_2Br_2^-$, j = $(R^{Ph3})_2ZnBr^-$, k = $(R^{Ph3})_3Zn^-$.

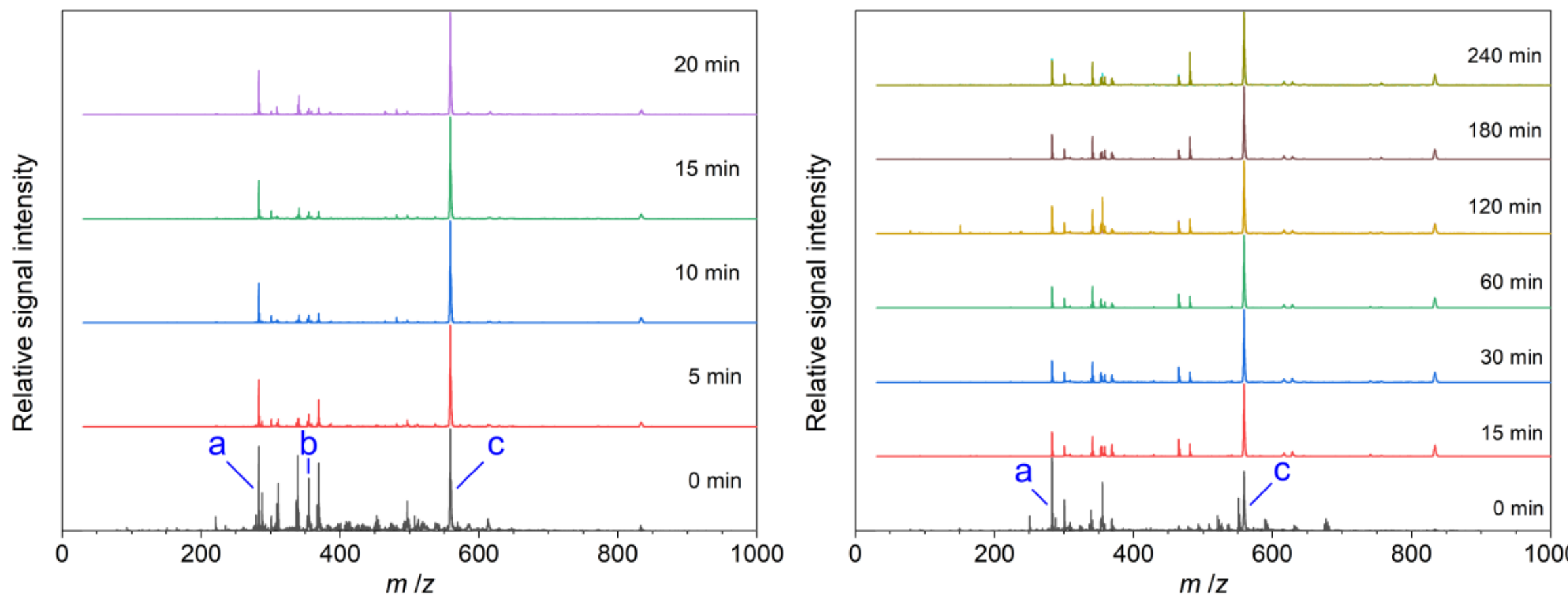

Figure S60. Positive-ion mode ESI-mass spectra of solutions of (R^{Ph3})ZnBr·LiCl·LiBr in THF at 0.038 M (left) and 0.16 M (right), upon exposure to ambient atmosphere for different times, a = Li(HOR^{Ph3})$^+$, b = Li(HOR^{Ph3})(THF)$^+$, c = Li(HOR^{Ph3})$_2^+$.

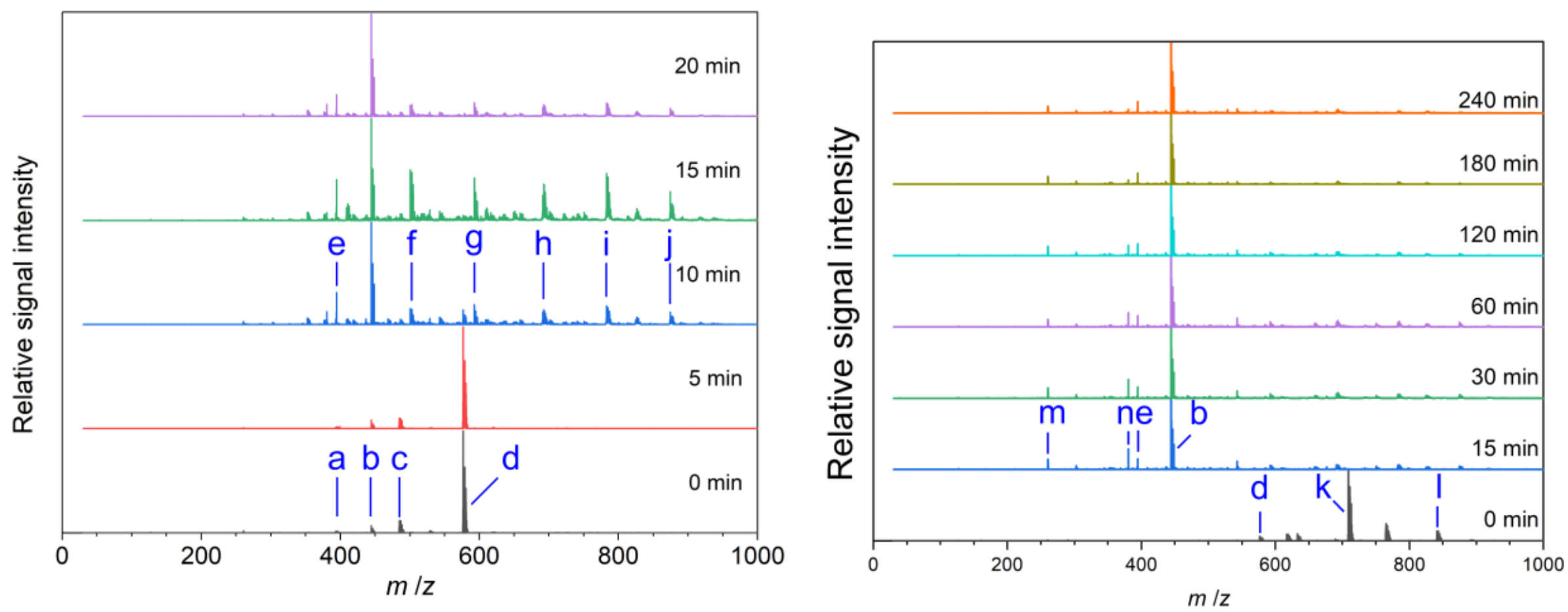

Figure S61. Negative-ion mode ESI-mass spectra of solutions of (R^{Ph3})ZnI·LiCl·LiI in THF at 0.038 M (left) and 0.16 M (right), upon exposure to ambient atmosphere for different times, a = (R^{Ph3})$ZnCl_2^-$, b = ZnI_3^-, c = (R^{Ph3})ZnClI$^-$, d = (R^{Ph3})ZnI_2^-, e = $Li_2I_3^-$, f = Zn(OR^{Ph3})ClI$^-$, g = Zn(OR^{Ph3})I_2^-, h = LiZn(OR^{Ph3})$_2Cl_2^-$, i = LiZn(OR^{Ph3})$_2$ClI$^-$, j = LiZn(OR^{Ph3})$_2I_2^-$, k = (R^{Ph3})$_2$ZnI$^-$, l = (R^{Ph3})$_3$Zn$^-$, m = LiI_2^-, n = I_3^-.

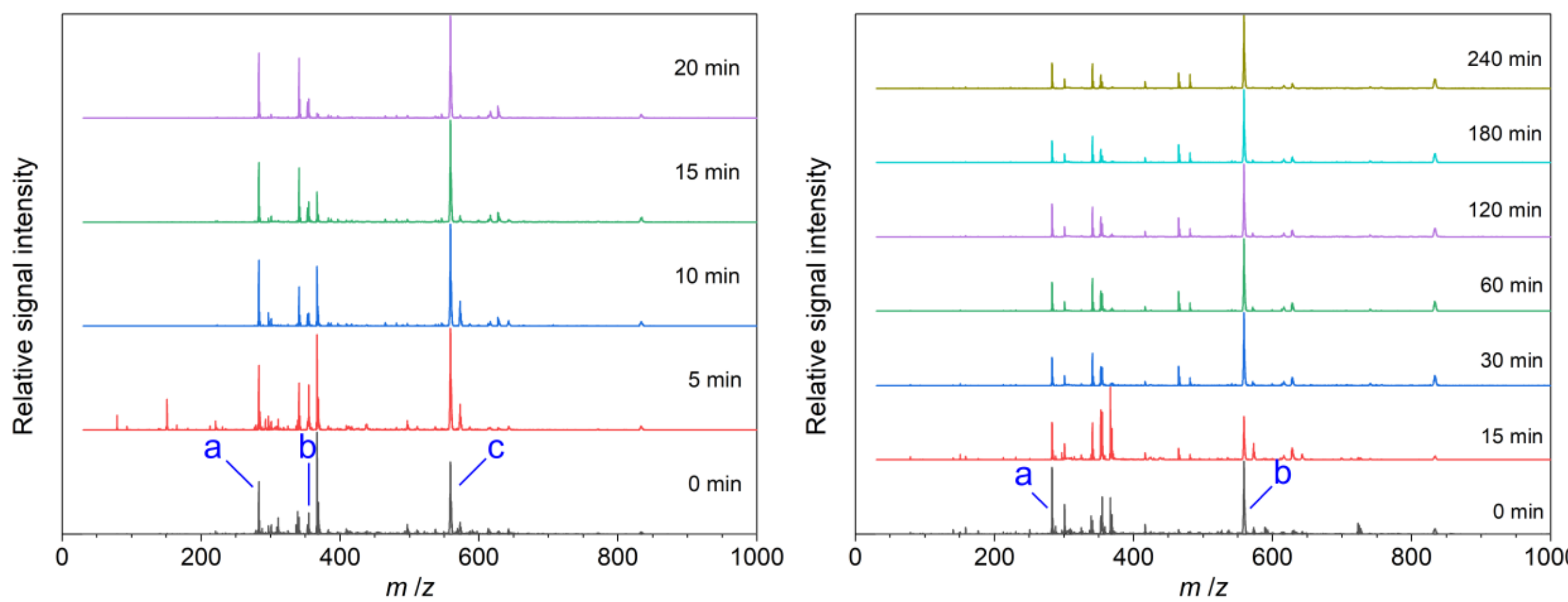

Figure S62. Positive-ion mode ESI-mass spectra of solutions of (R^{Ph3})ZnI·LiCl·LiI in THF at 0.038 M (left) and 0.16 M (right), upon exposure to ambient atmosphere for different times, a = Li(HOR^{Ph3})$^+$, b = Li(HOR^{Ph3})(THF)$^+$, c = Li(HOR^{Ph3})$_2^+$.

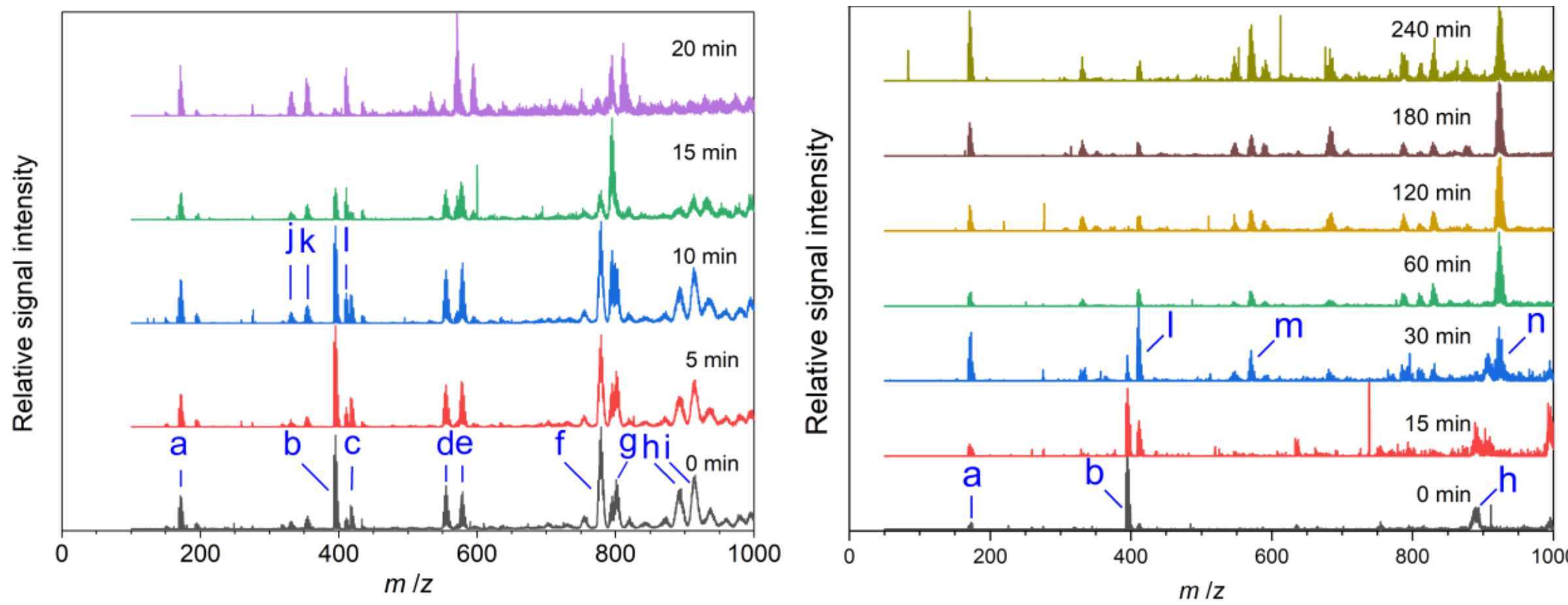

Figure S63. Negative-ion mode ESI-mass spectra of solutions of $(R^{Ph3})ZnOAc\cdot LiCl\cdot LiOAc$ in THF at 0.038 M (left) and 0.16 M (right), upon exposure to ambient atmosphere for different times, a = $ZnCl_3^-$, b = $(R^{Ph3})ZnCl_2^-$, c = $(R^{Ph3})Zn(OAc)Cl^-$, d = $(R^{Ph3})Zn_2(OAc)Cl_3^-$, e = $(R^{Ph3})Zn_2(OAc)_2Cl_2^-$, f = $(R^{Ph3})_2Zn_2(OAc)Cl_2^-$, g = $(R^{Ph3})_2Zn_2(OAc)_2Cl^-$, h = $(R^{Ph3})_2Zn_3Cl_5^-$, i = $(R^{Ph3})_2Zn_3(OAc)Cl_4^-$, j = $Zn_2(OAc)Cl_4^-$, k = $Zn_2(OAc)_2Cl_3^-$, l = $(OR^{Ph3})ZnCl_2^-$, m = $(R^{Ph3})_2ZnCl_2^-$, n = $(R^{Ph3})_2Zn_2Cl_4(OAc)_2Li_2^-$.

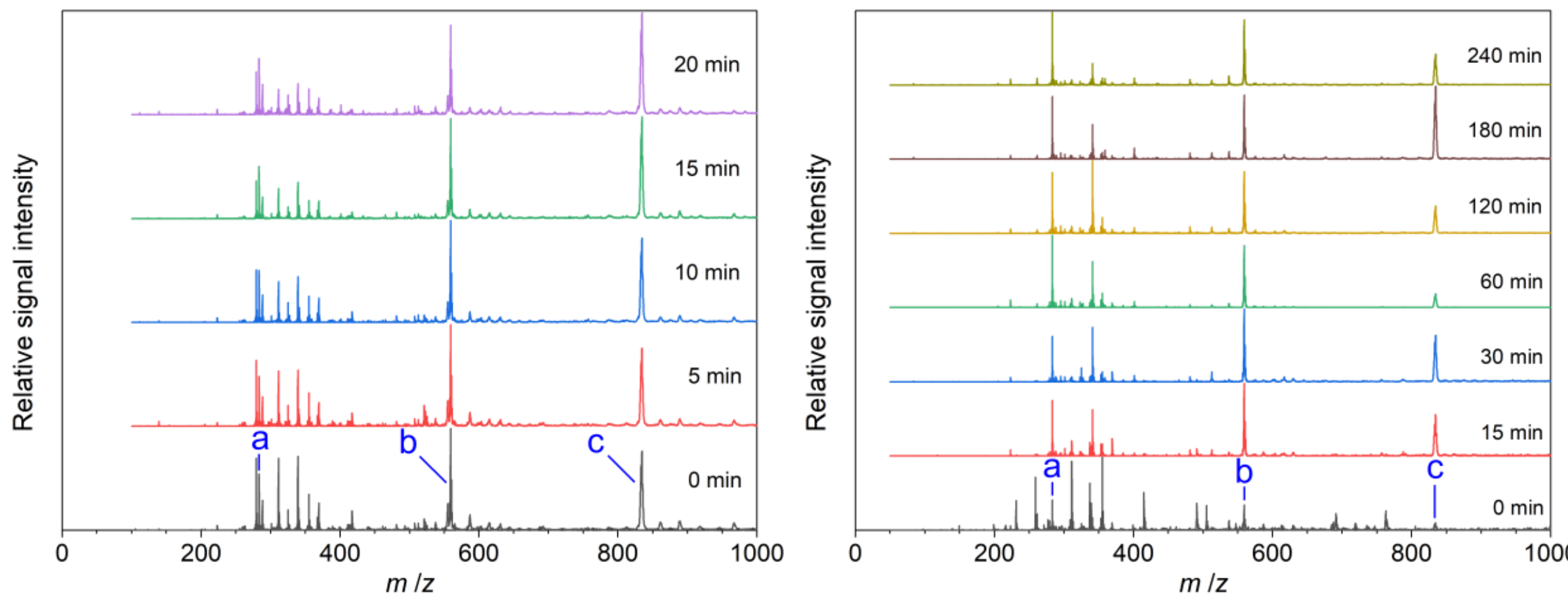

Figure S64. Positive-ion mode ESI-mass spectra of solutions of $(R^{Ph3})ZnOAc\cdot LiCl\cdot LiOAc$ in THF at 0.038 M (left) and 0.16 M (right), upon exposure to ambient atmosphere for different times, a = $Li(HOR^{Ph3})^+$, b = $Li(HOR^{Ph3})_2^+$, c = $Li(HOR^{Ph3})_3^+$.

5. Time-dependent ^{1}H-NMR experiments under ambient atmosphere

5.1 (R^{Ph})ZnX·LiX·LiCl (X = OPiv, Cl, Br, I) systems

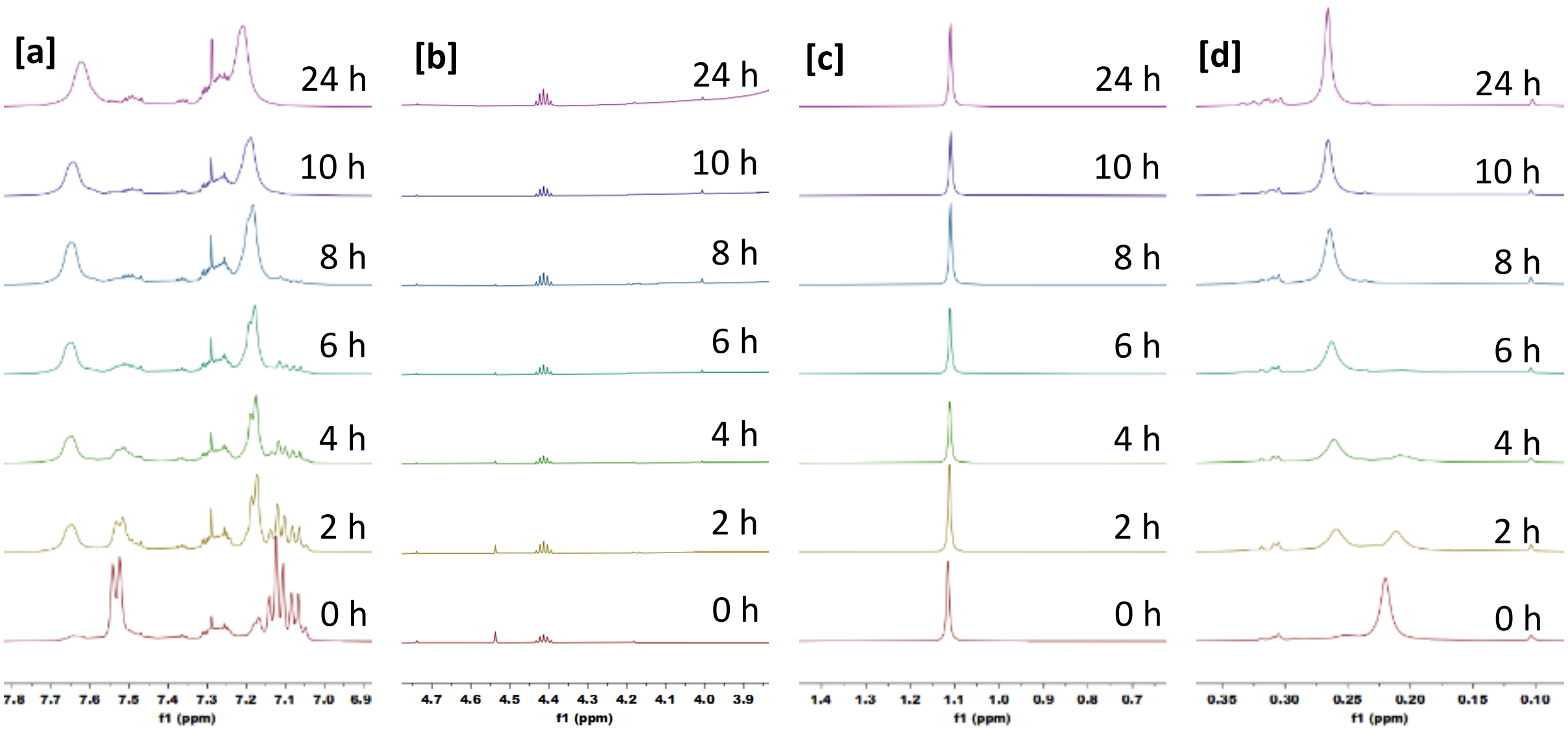

Figure S65. Time-dependent ^{1}H-NMR spectra of (R^{Ph})Zn(OPiv)·Li(OPiv)·LiCl in THF-d_8 (400 MHz). **[a]** Aryl region. **[b]** Si–OH region. **[c]** OPiv region. **[d]** CH_3 region.

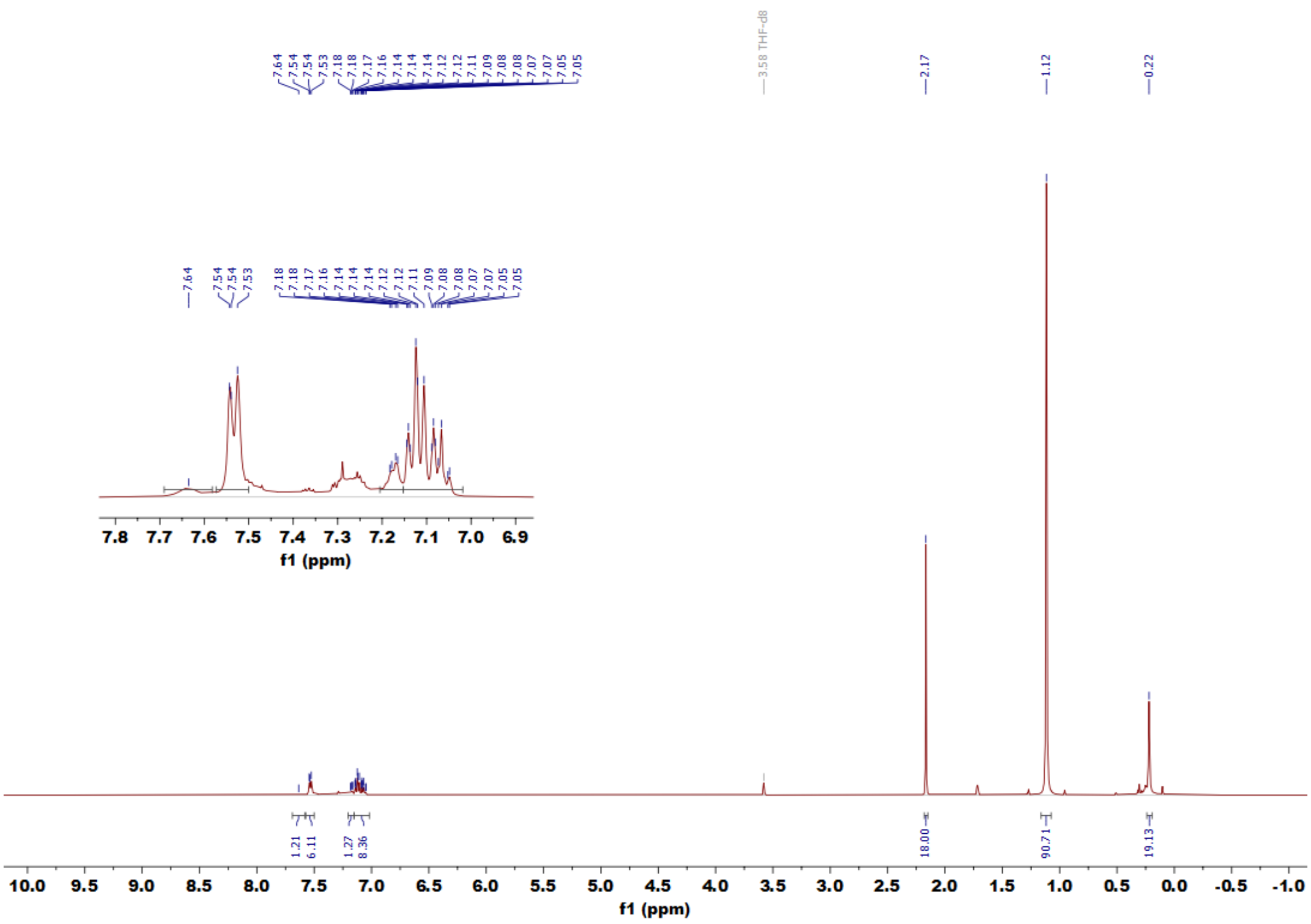

Figure S66. ^{1}H-NMR spectrum of (R^{Ph})Zn(OPiv)·Li(OPiv)·LiCl in THF-d_8 (400 MHz) at 0 h.

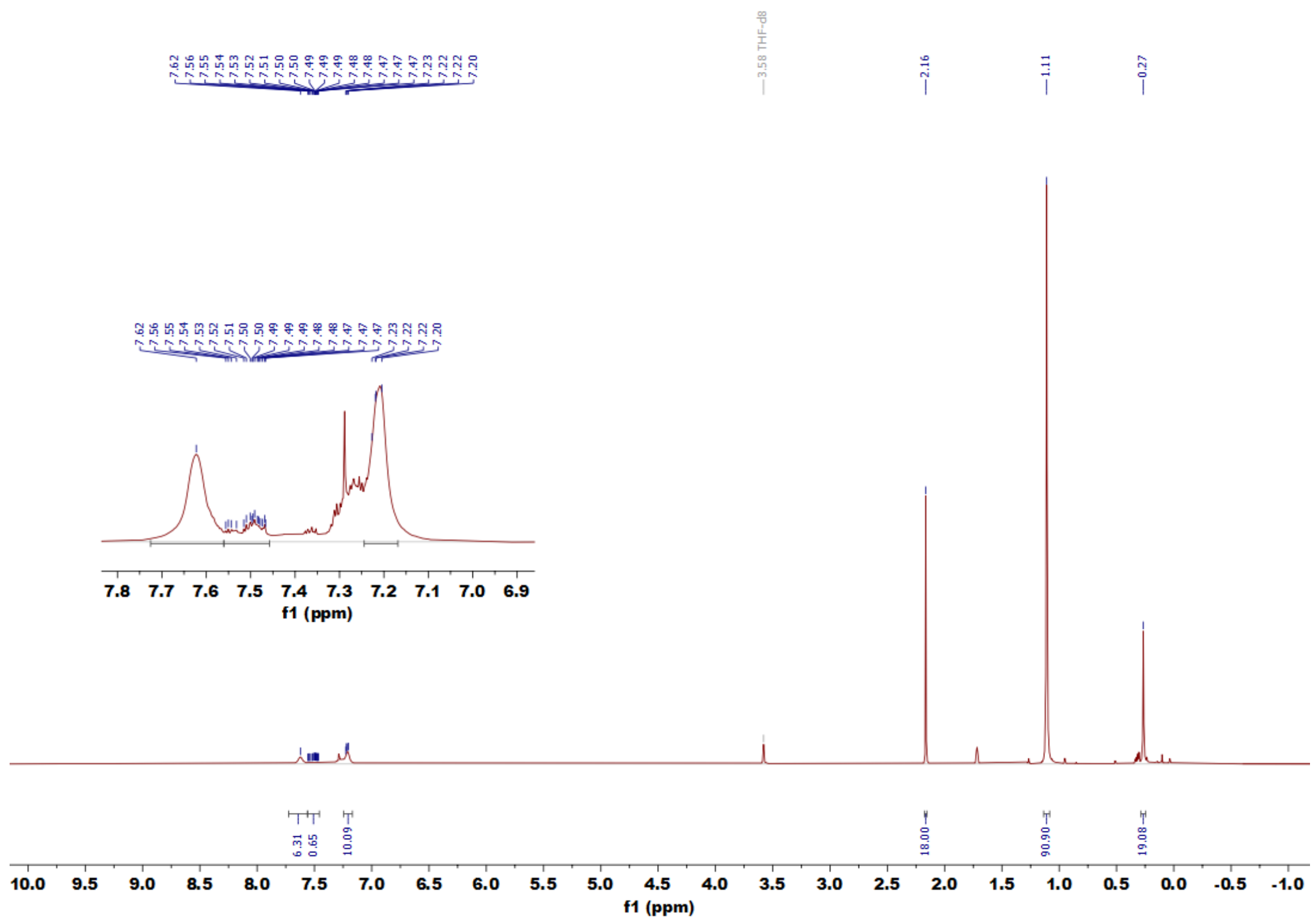

Figure S67. ^{1}H-NMR spectrum of (R^{Ph})Zn(OPiv)·Li(OPiv)·LiCl in THF-d_8 (400 MHz) after exposure for 24 h.

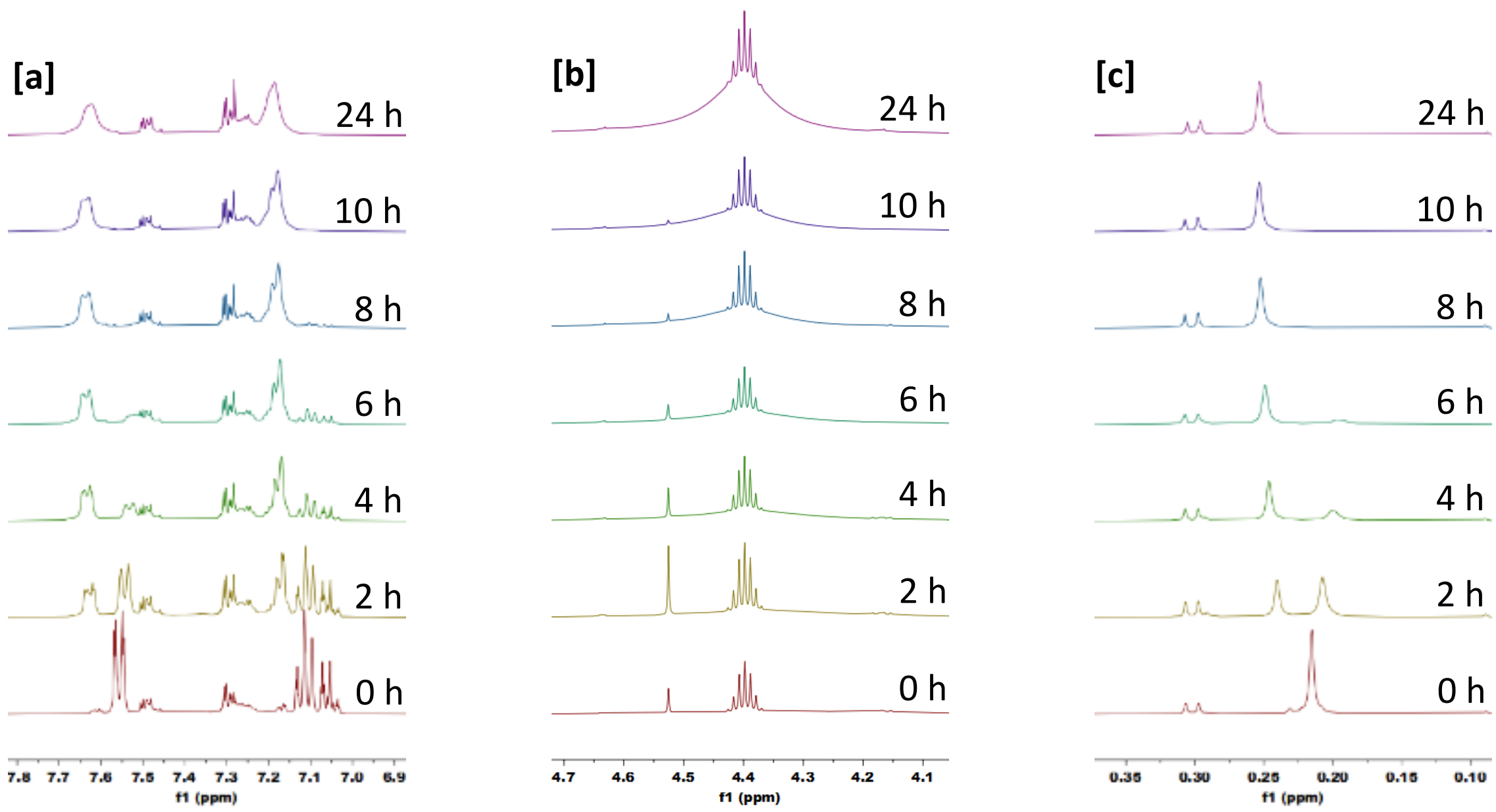

Figure S68. Time-dependent ^{1}H-NMR spectra of (R^{Ph})ZnCl·2 LiCl in THF-d_8 (400 MHz). **[a]** Aryl region. **[b]** Si–OH region. **[c]** CH_3 region.

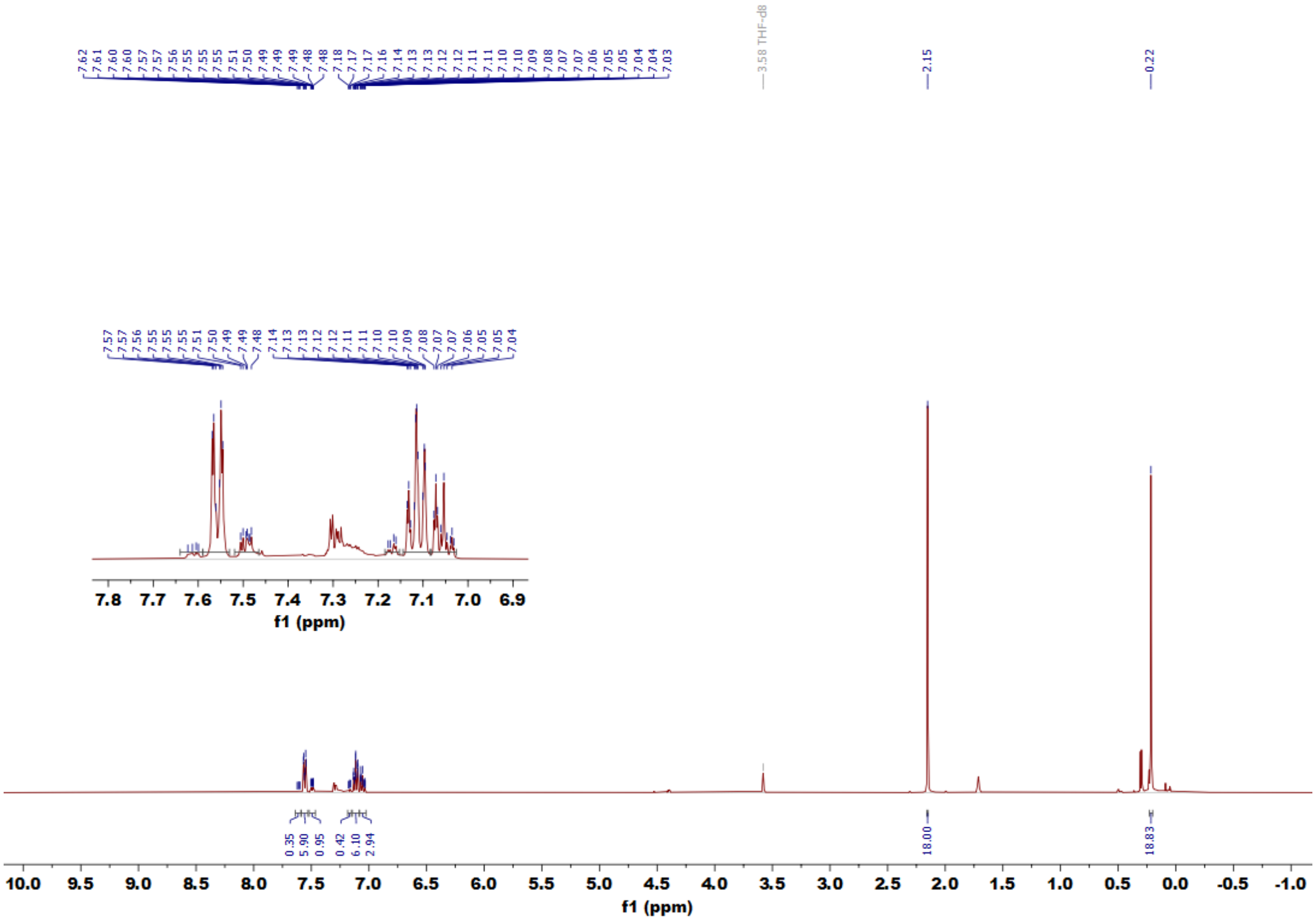

Figure S69. ^{1}H-NMR spectrum of (R^{Ph})ZnCl·2 LiCl in THF-d_8 (400 MHz) at 0 h.

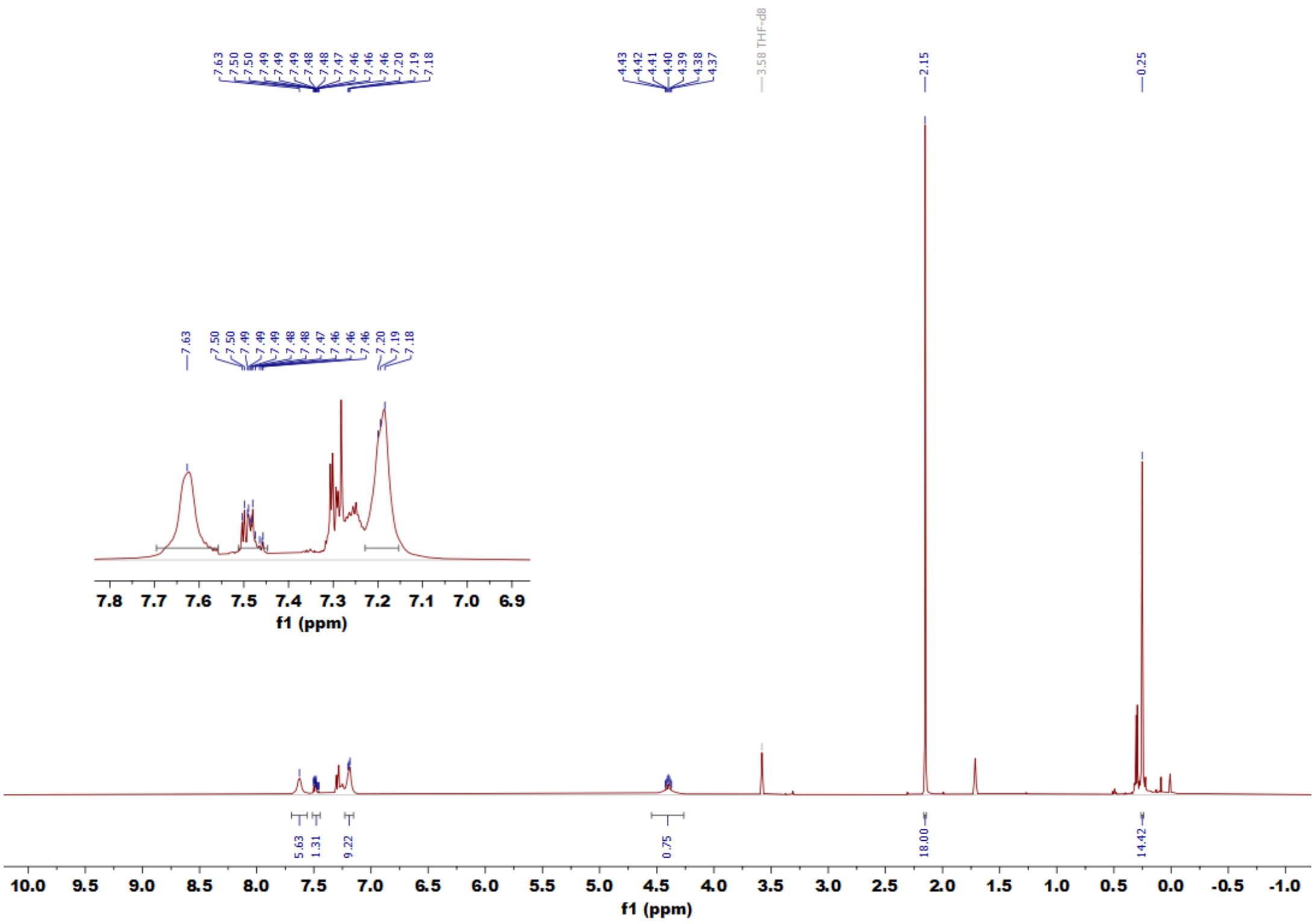

Figure S70. ^{1}H-NMR spectrum of (R^{Ph})ZnCl·2 LiCl in THF-d_8 (400 MHz) after exposure for 24 h.

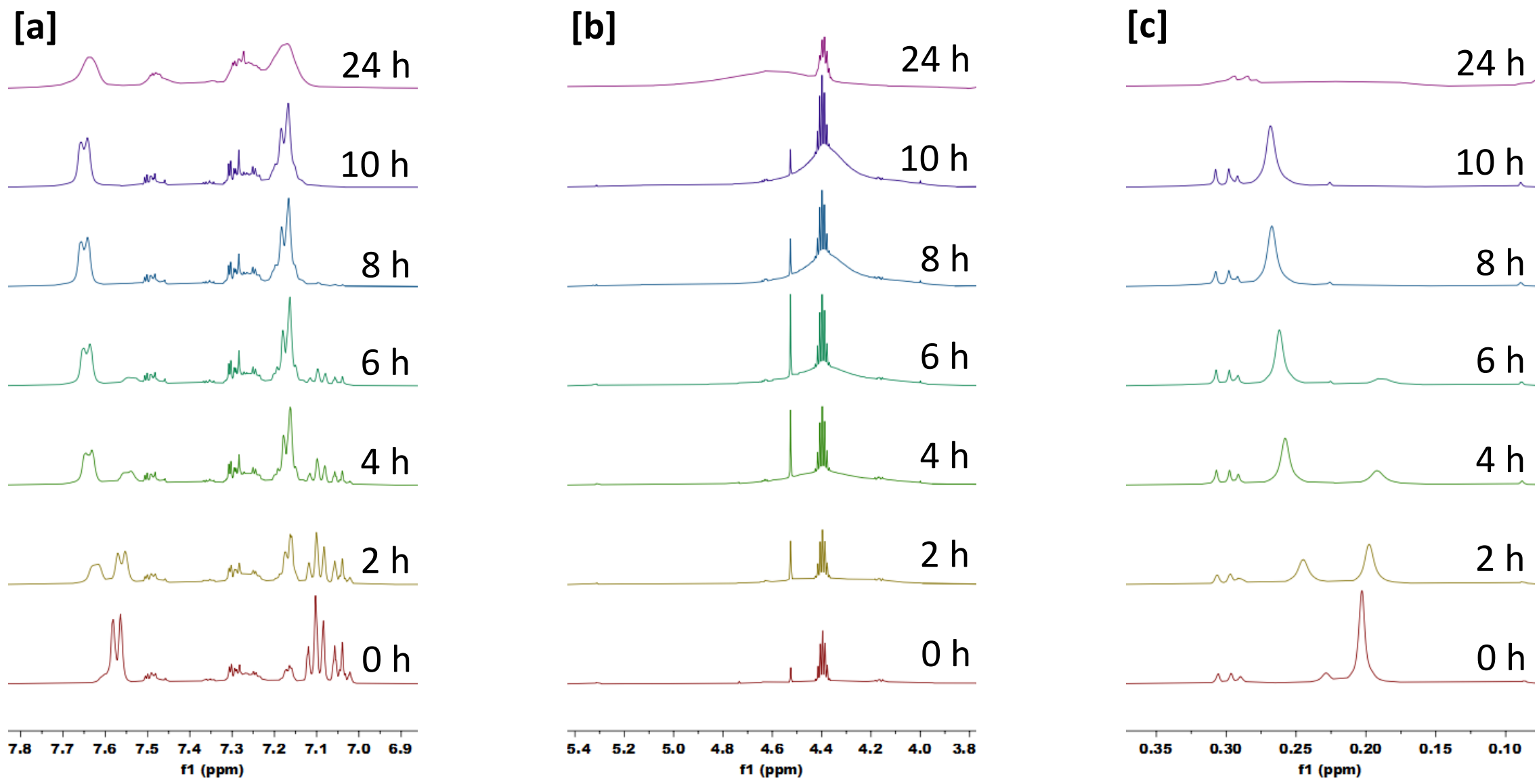

Figure S71. Time-dependent ^{1}H-NMR spectra of (R^{Ph})ZnBr·LiCl·LiBr in THF-d_8 (400 MHz). **[a]** Aryl region. **[b]** Si–OH region. **[c]** CH_3 region.

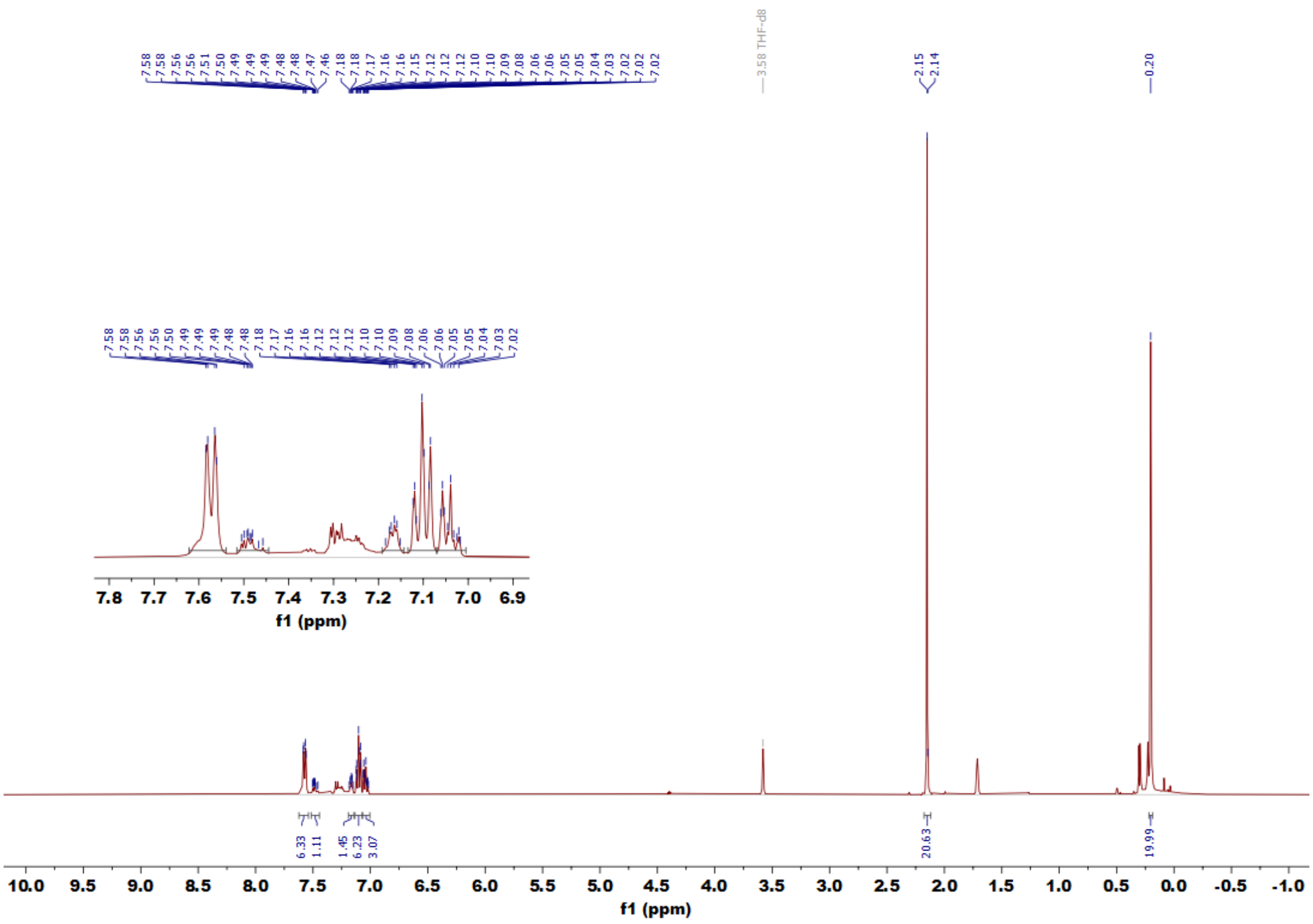

Figure S72. ^{1}H-NMR spectrum of (R^{Ph})ZnBr·LiCl·LiBr in THF-d_8 (400 MHz) at 0 h.

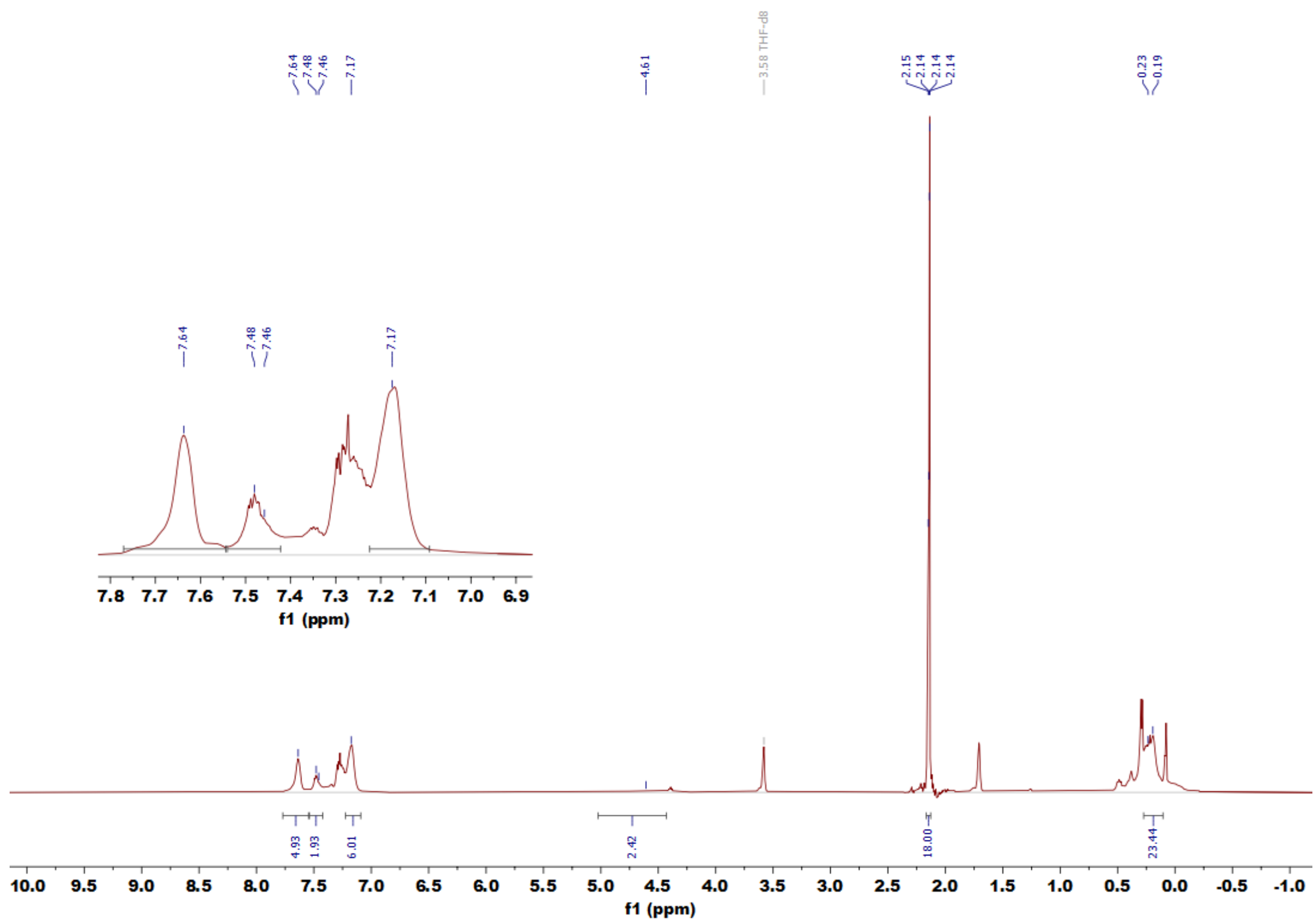

Figure S73. ^{1}H-NMR spectrum of (R^{Ph})ZnBr·LiCl·LiBr in THF-d_8 (400 MHz) after exposure for 24 h.

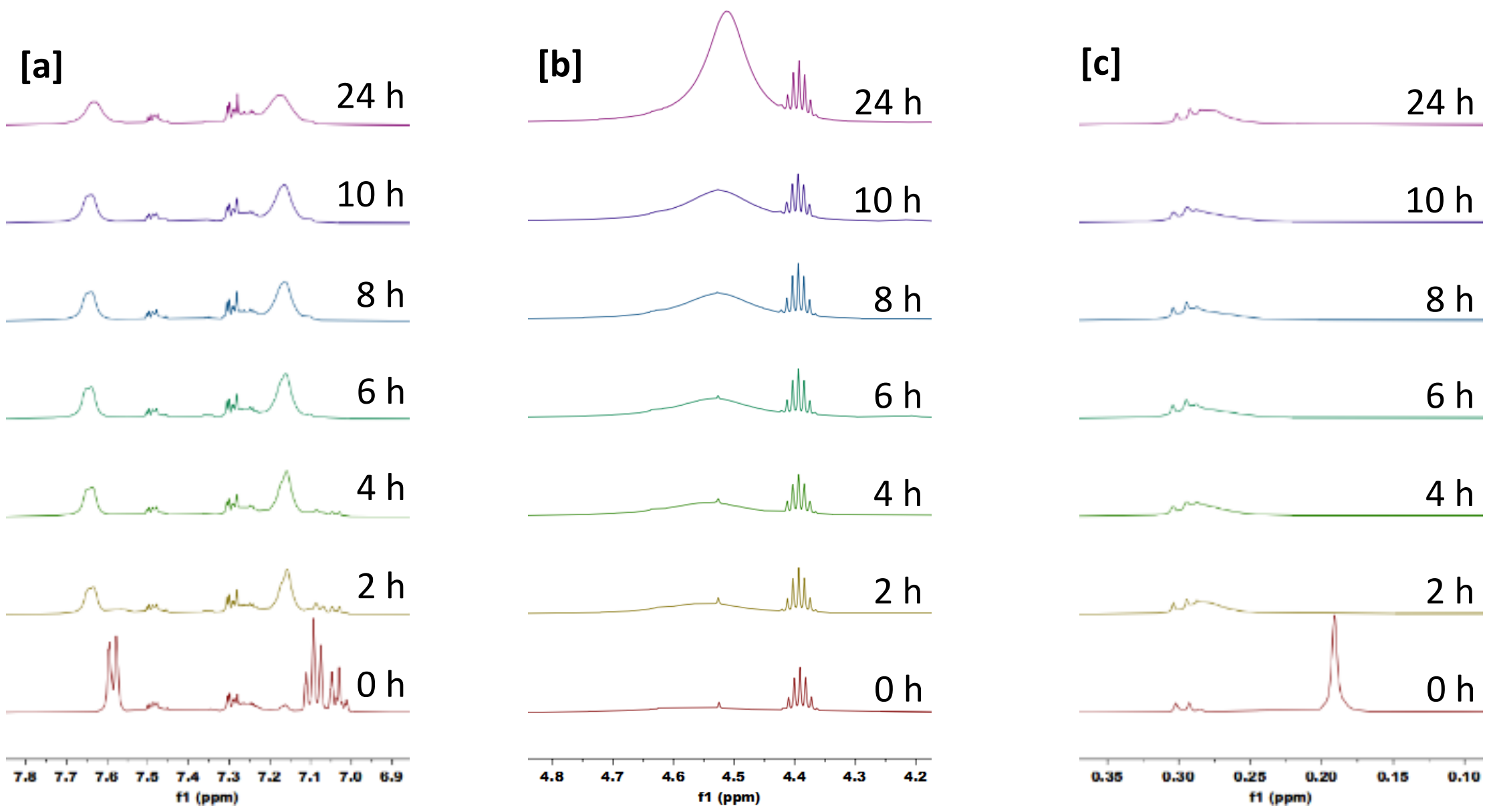

Figure S74. Time-dependent ^{1}H-NMR spectra of (R^{Ph})ZnI·LiCl·LiI in THF-d_8 (400 MHz). **[a]** Aryl region. **[b]** Si–OH region. **[c]** CH_3 region.

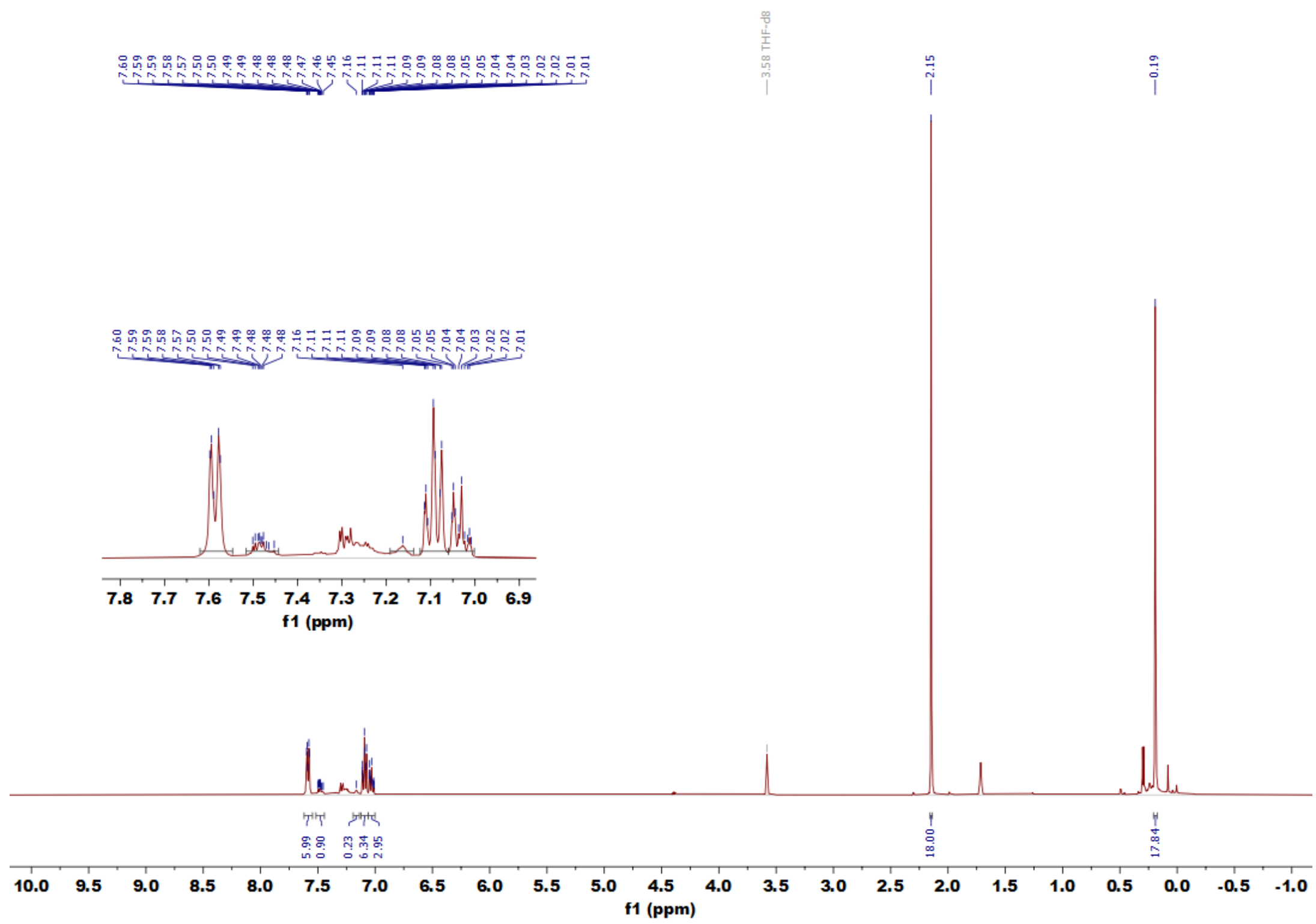

Figure S75. ^{1}H-NMR spectrum of (R^{Ph})ZnI·LiCl·LiI in THF-d_8 (400 MHz) at 0 h.

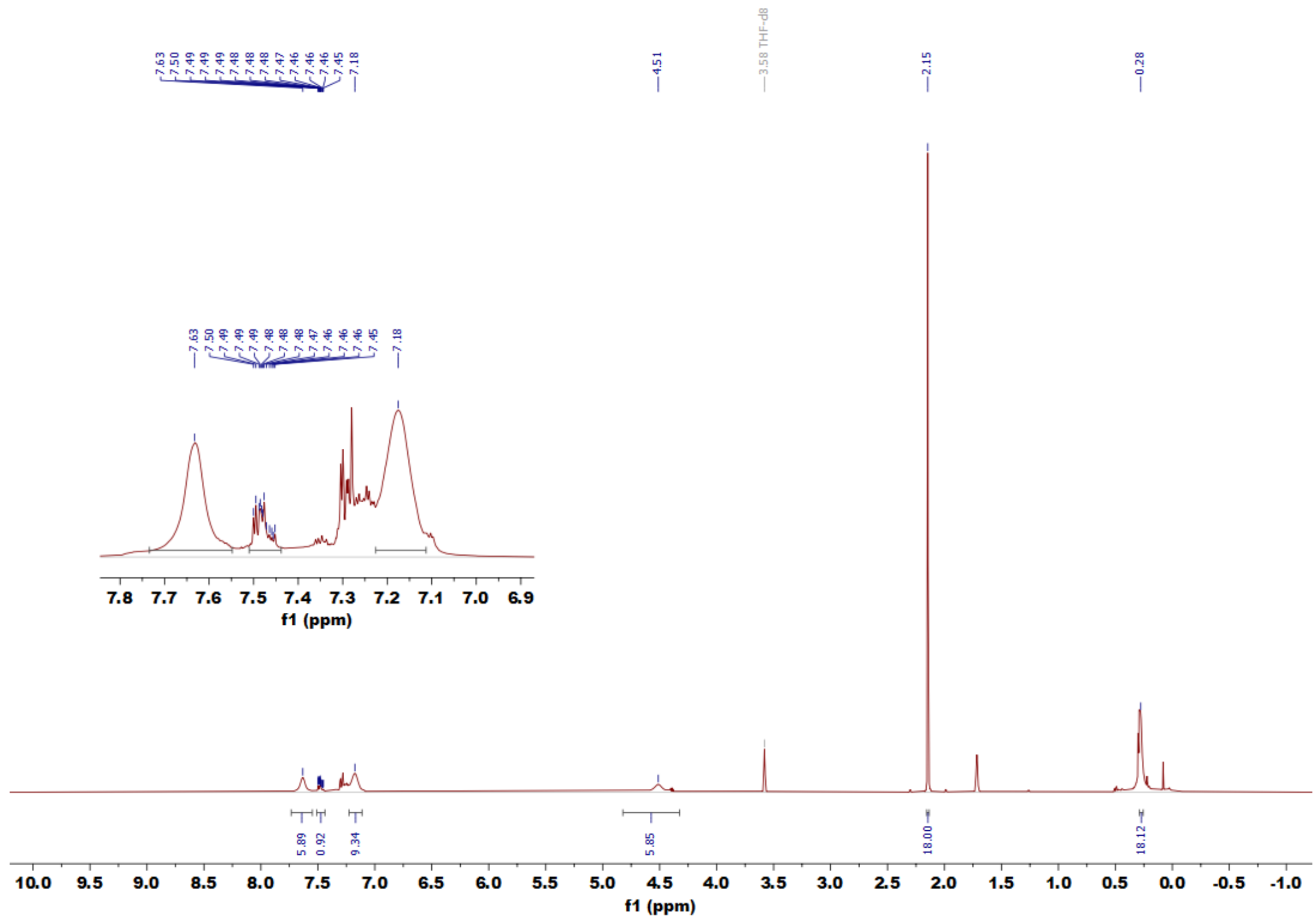

Figure S76. ^{1}H-NMR spectrum of (R^{Ph})ZnI·LiCl·LiI recorded in THF-d_8 (400 MHz) after exposure for 24 h.

5.2 (R^{Ph2})ZnX·LiX·LiCl (X = OPiv, Cl, Br, I) systems

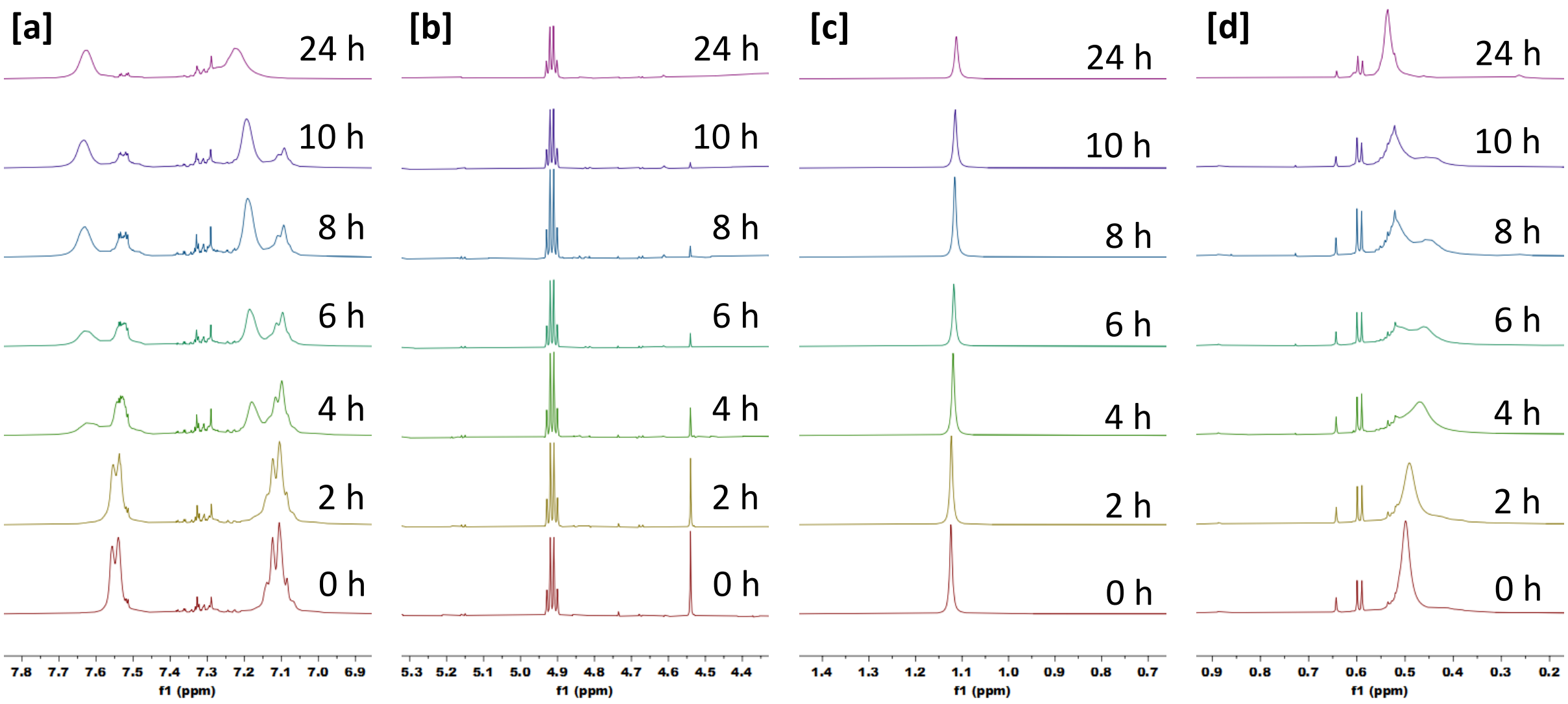

Figure S77. Time-dependent ^{1}H-NMR spectra of (R^{Ph2})Zn(OPiv)·Li(OPiv)·LiCl in THF-d_8 (400 MHz). **[a]** Aryl region. **[b]** Si–OH region. **[c]** OPiv region. **[d]** CH_3 region.

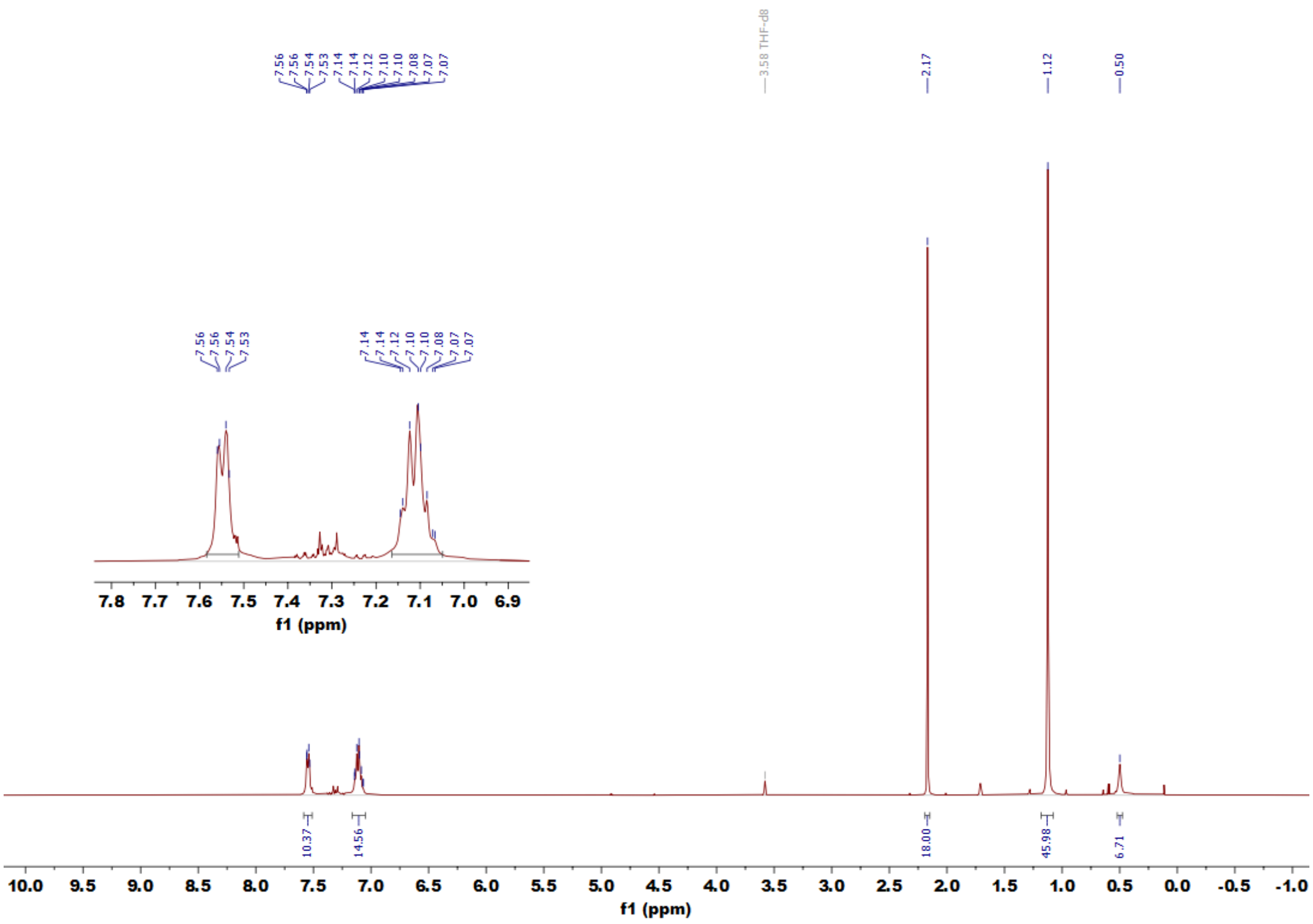

Figure S78. ^{1}H-NMR spectrum of (R^{Ph2})Zn(OPiv)·Li(OPiv)·LiCl recorded in THF-d_8 (400 MHz) at 0 h.

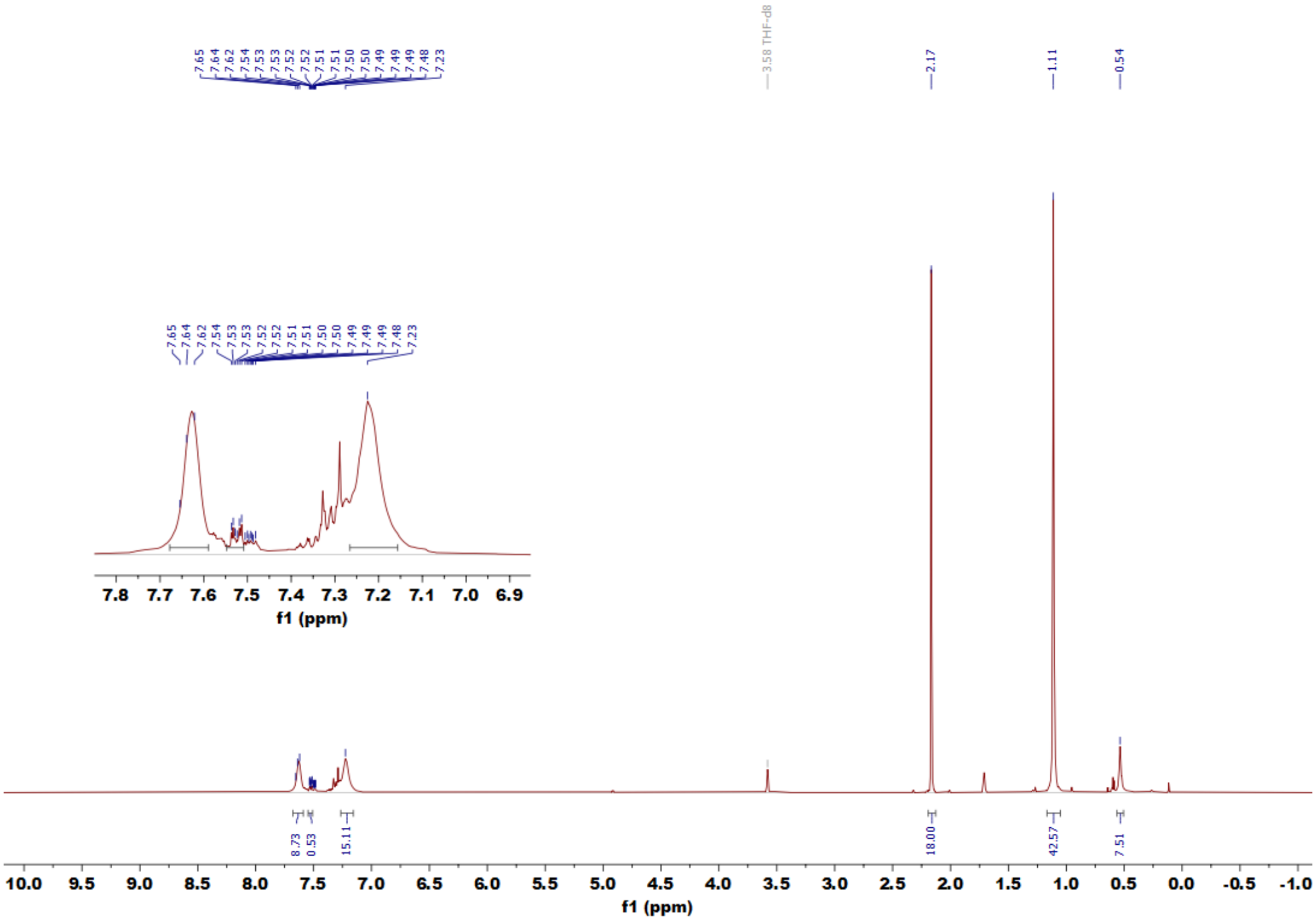

Figure S79. ^{1}H-NMR spectrum of (R^{Ph2})Zn(OPiv)·Li(OPiv)·LiCl in THF-d_8 (400 MHz) after exposure for 24 h.

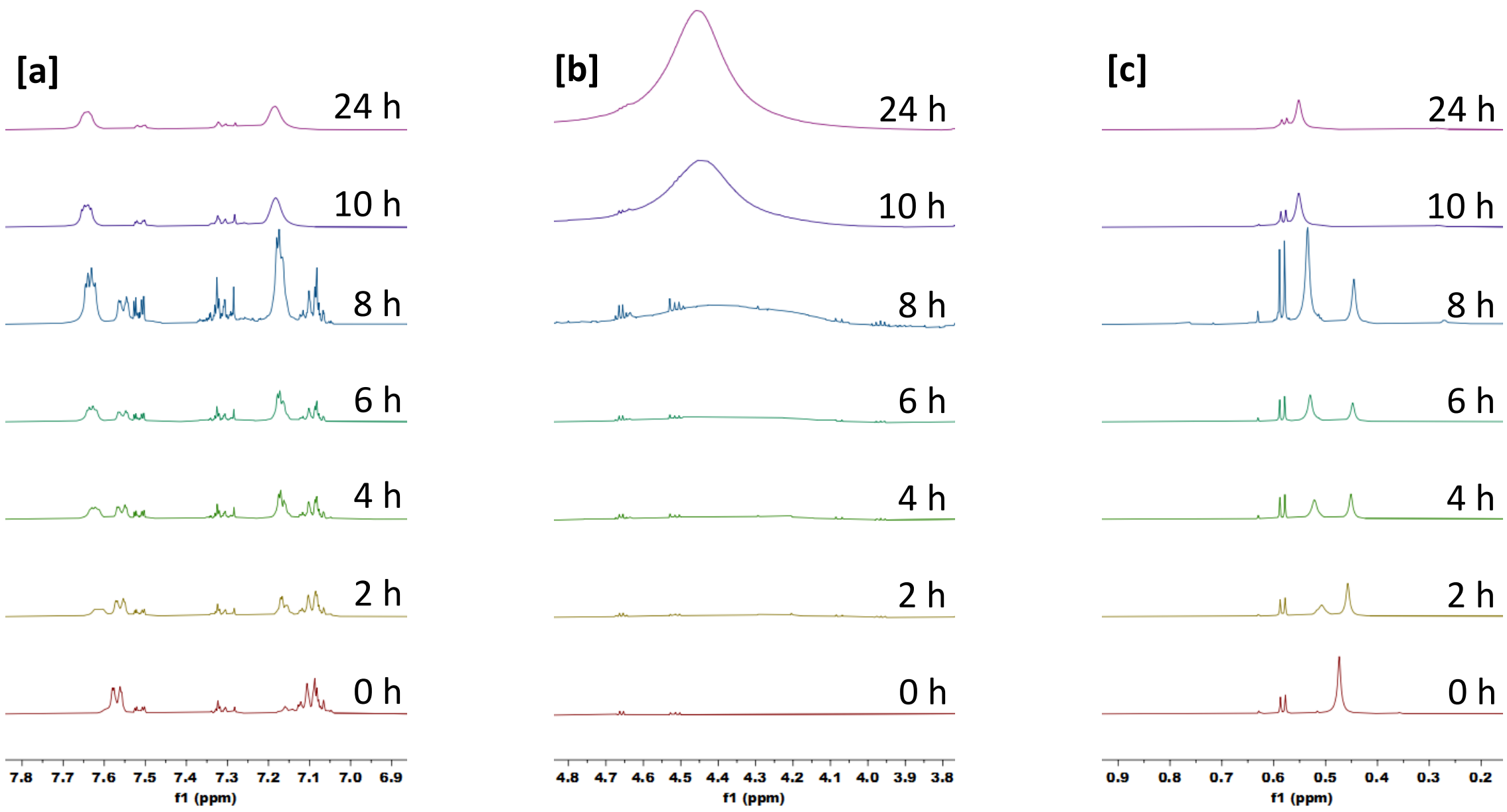

Figure S80. Time-dependent ^{1}H-NMR spectra of (R^{Ph2})ZnCl·2 LiCl in THF-d_8 (400 MHz). **[a]** Aryl region. **[b]** Si–OH region. **[c]** CH_3 region.

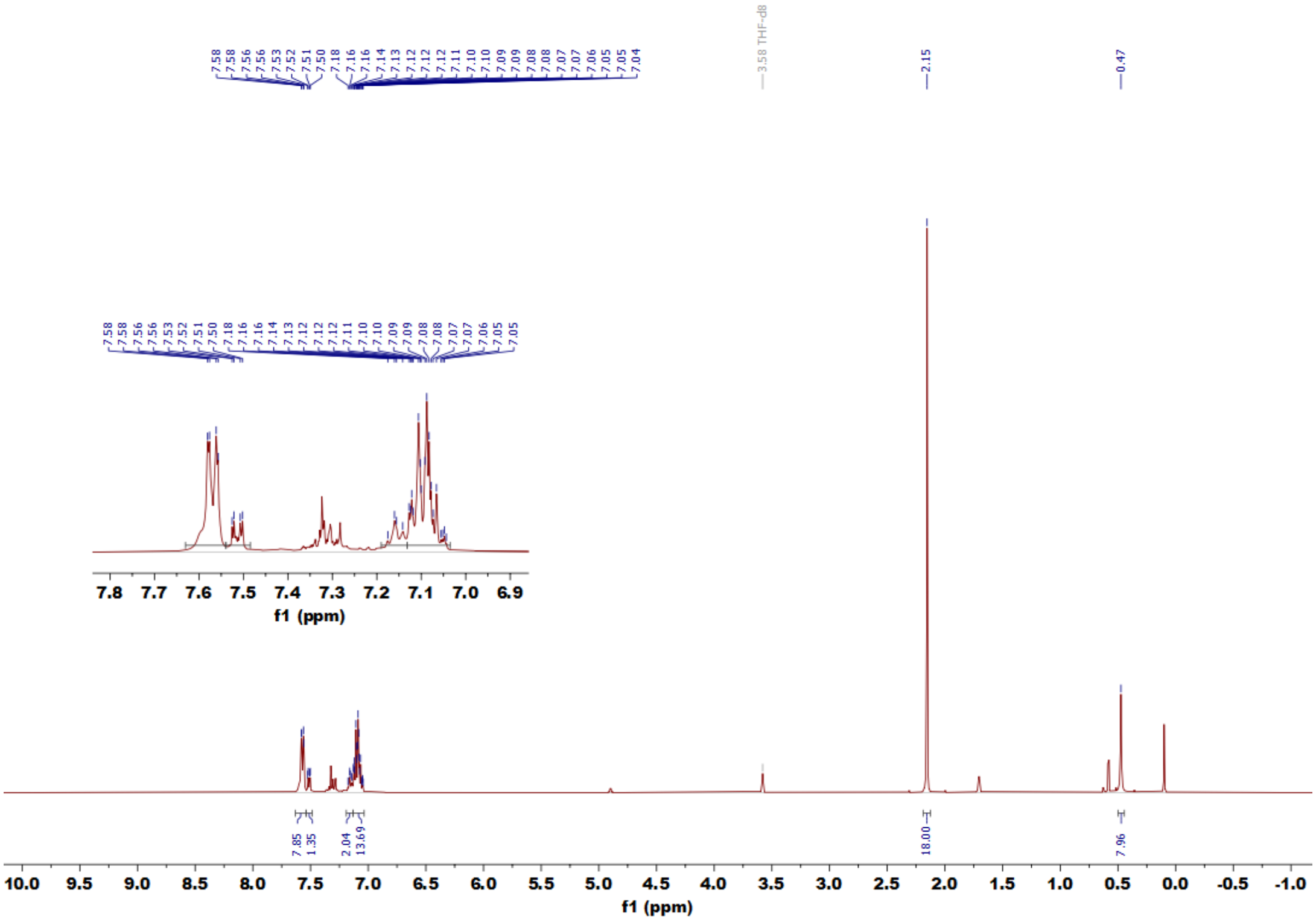

Figure S81. ^{1}H-NMR spectrum of (R^{Ph2})ZnCl·2 LiCl in THF-d_8 (400 MHz) at 0 h.

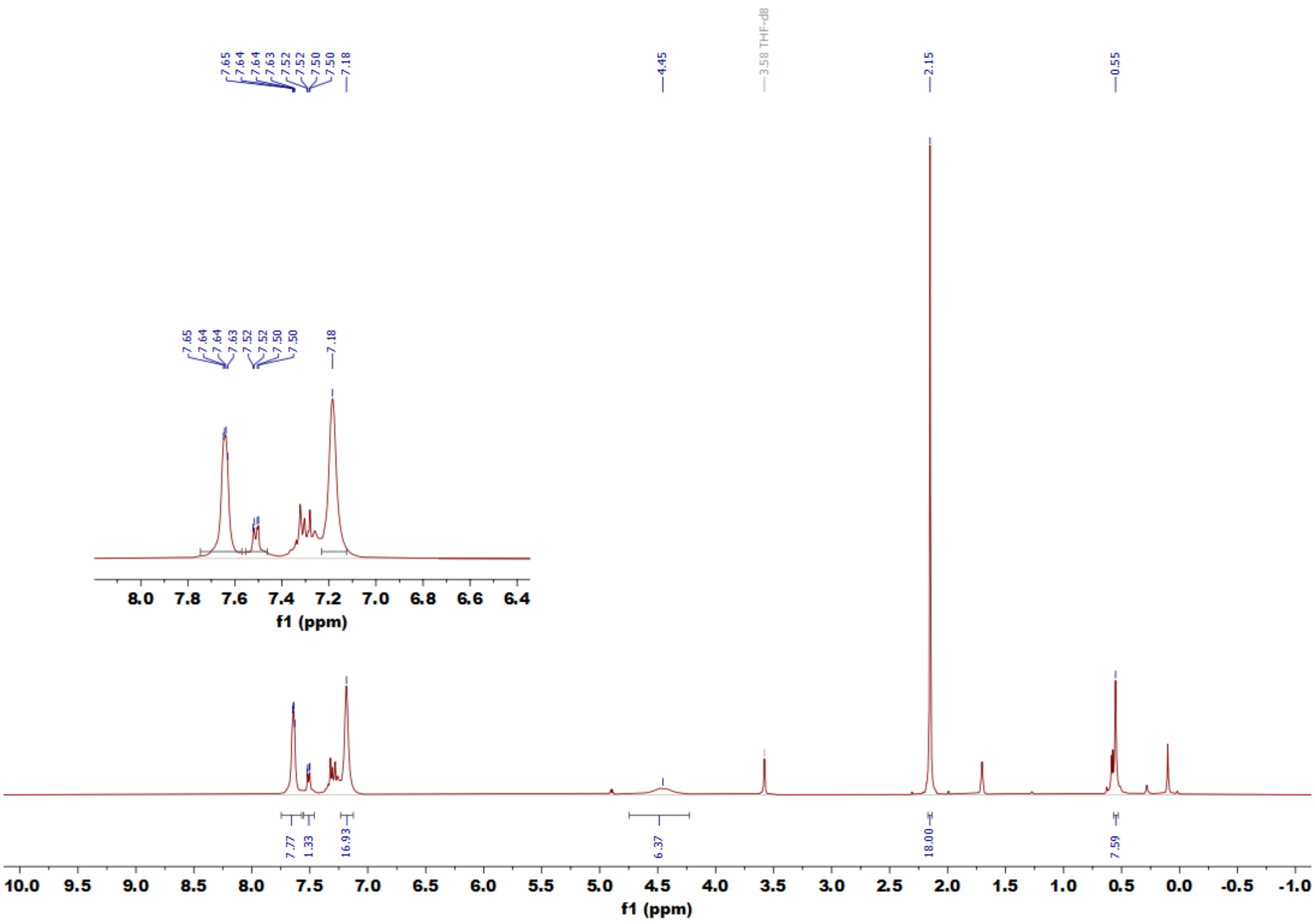

Figure S82. ^{1}H-NMR spectrum of (R^{Ph2})ZnCl·2 LiCl in THF-d_8 (400 MHz) after exposure for 24 h.

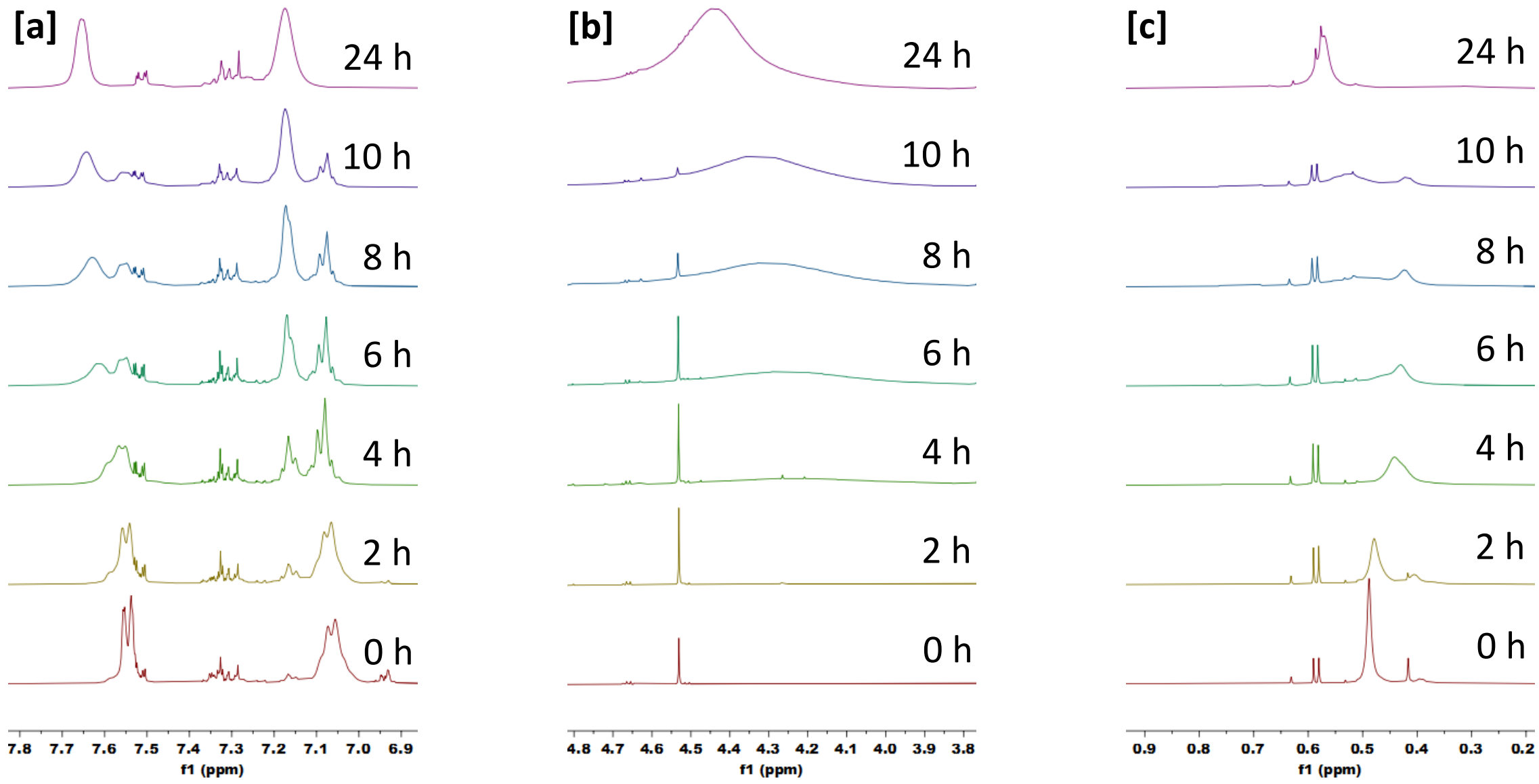

Figure S83. Time-dependent ^{1}H-NMR spectra of $(R^{Ph2})ZnBr \cdot LiCl \cdot LiBr$ in THF-d_8 (400 MHz). **[a]** Aryl region. **[b]** Si–OH region. **[c]** CH_3 region.

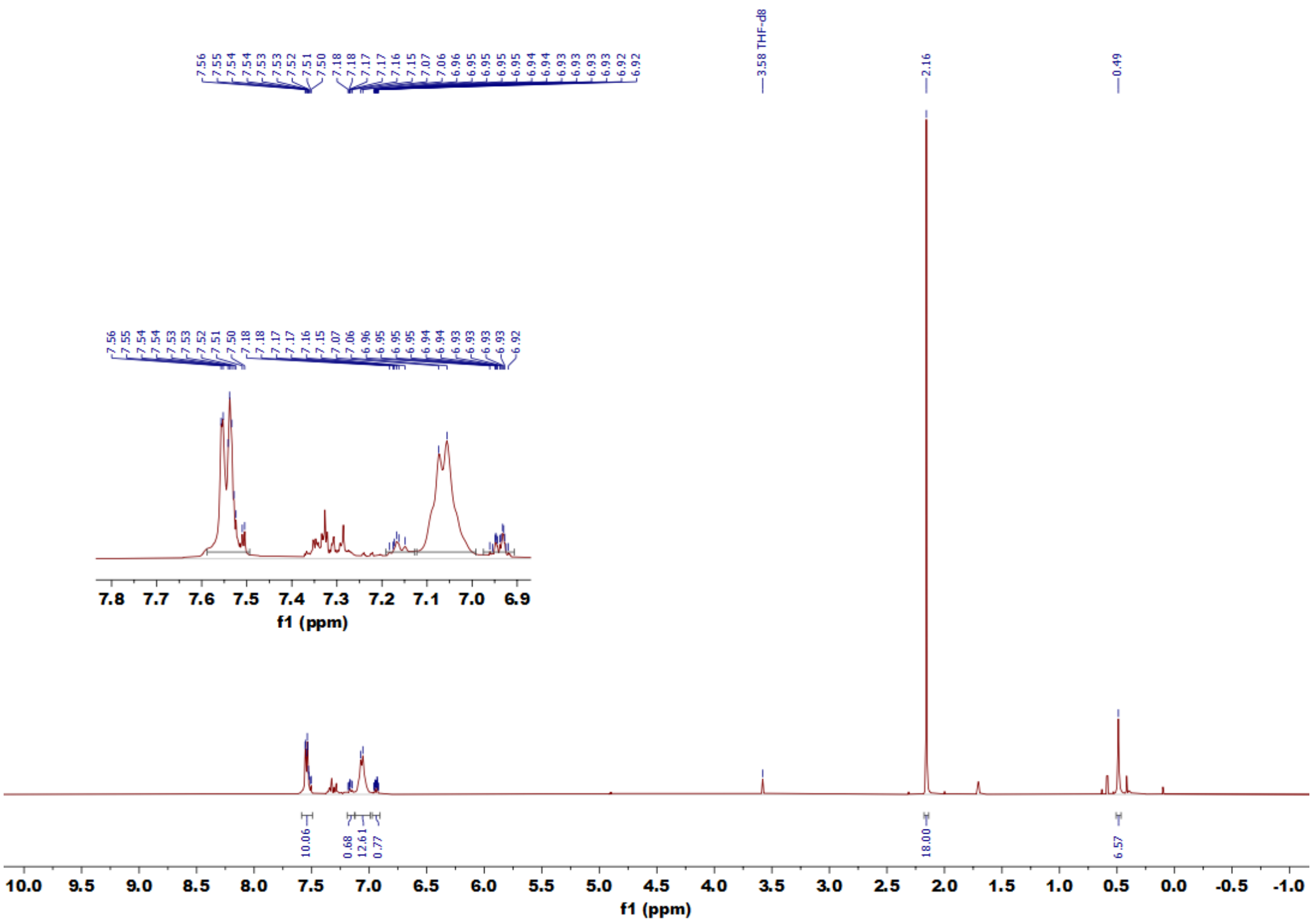

Figure S84. ^{1}H-NMR spectrum of $(R^{Ph2})ZnBr \cdot LiCl \cdot LiBr$ in THF-d_8 (400 MHz) at 0 h.

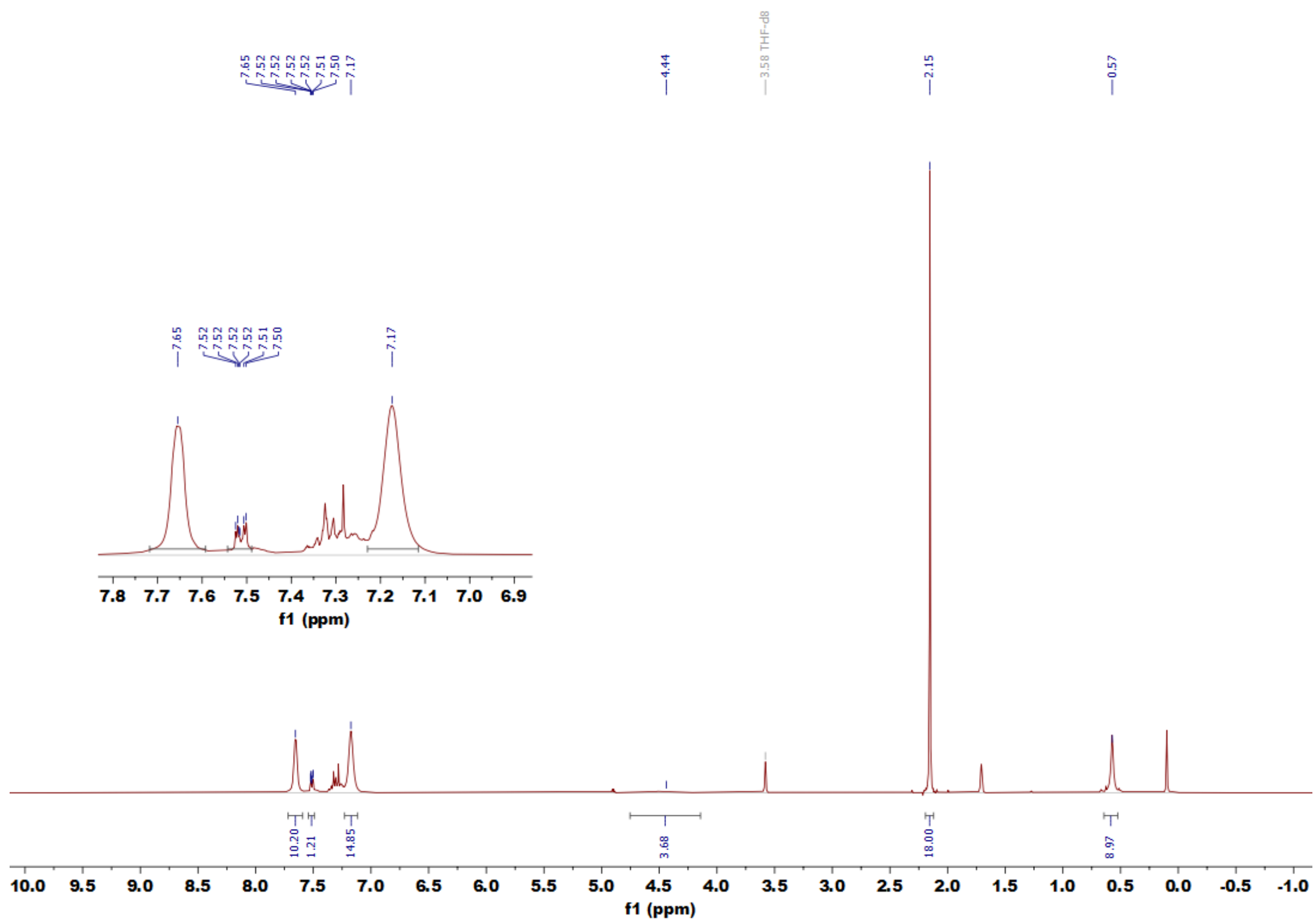

Figure S85. ^{1}H-NMR spectrum of (R^{Ph2})ZnBr·LiCl·LiBr in THF-d_8 (400 MHz) after exposure for 24 h.

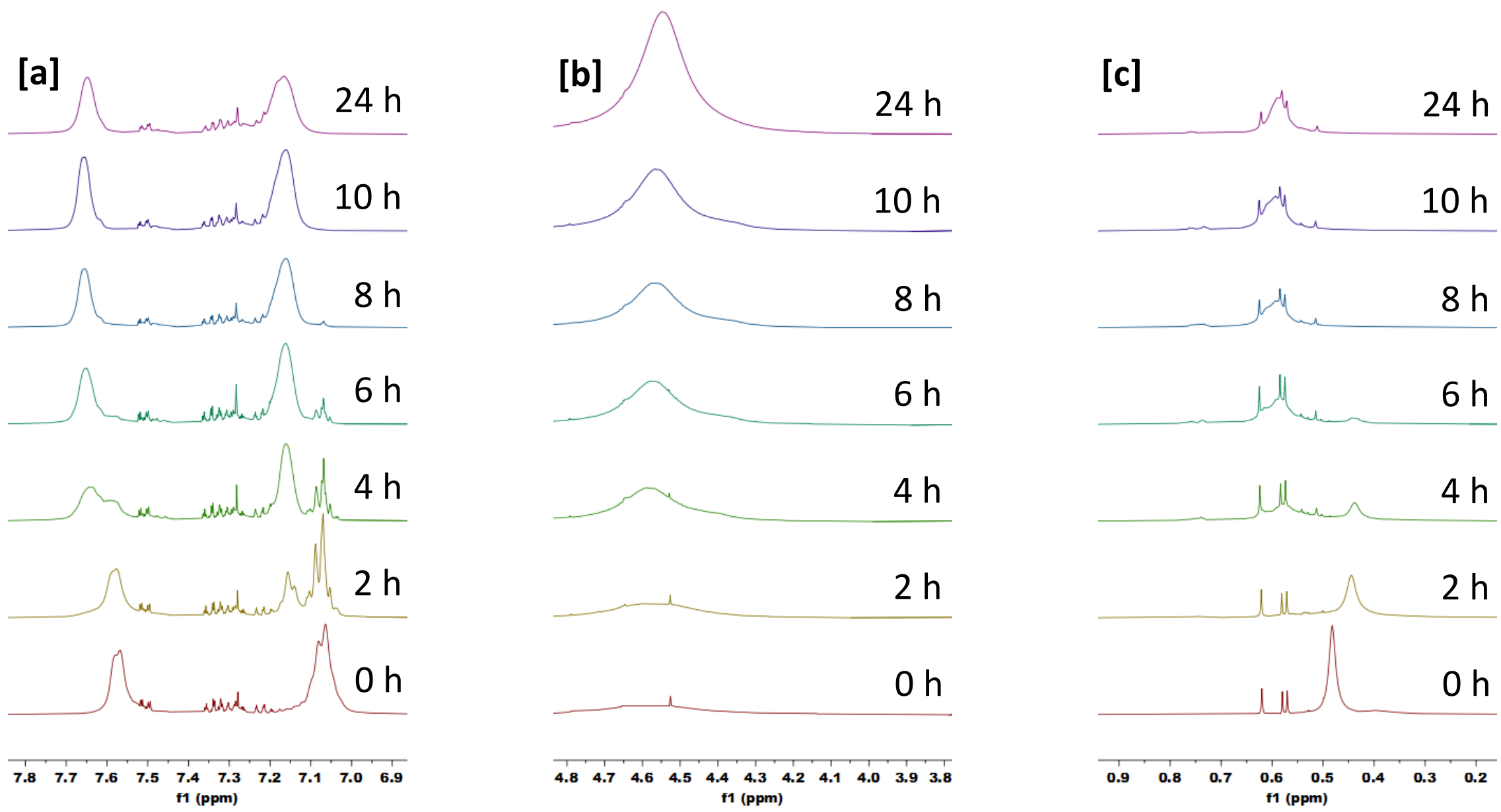

Figure S86. Time-dependent ^{1}H-NMR spectra of (R^{Ph2})ZnI·LiCl·LiI in THF-d_8 (400 MHz). **[a]** Aryl region. **[b]** Si–OH region. **[c]** CH_3 region.

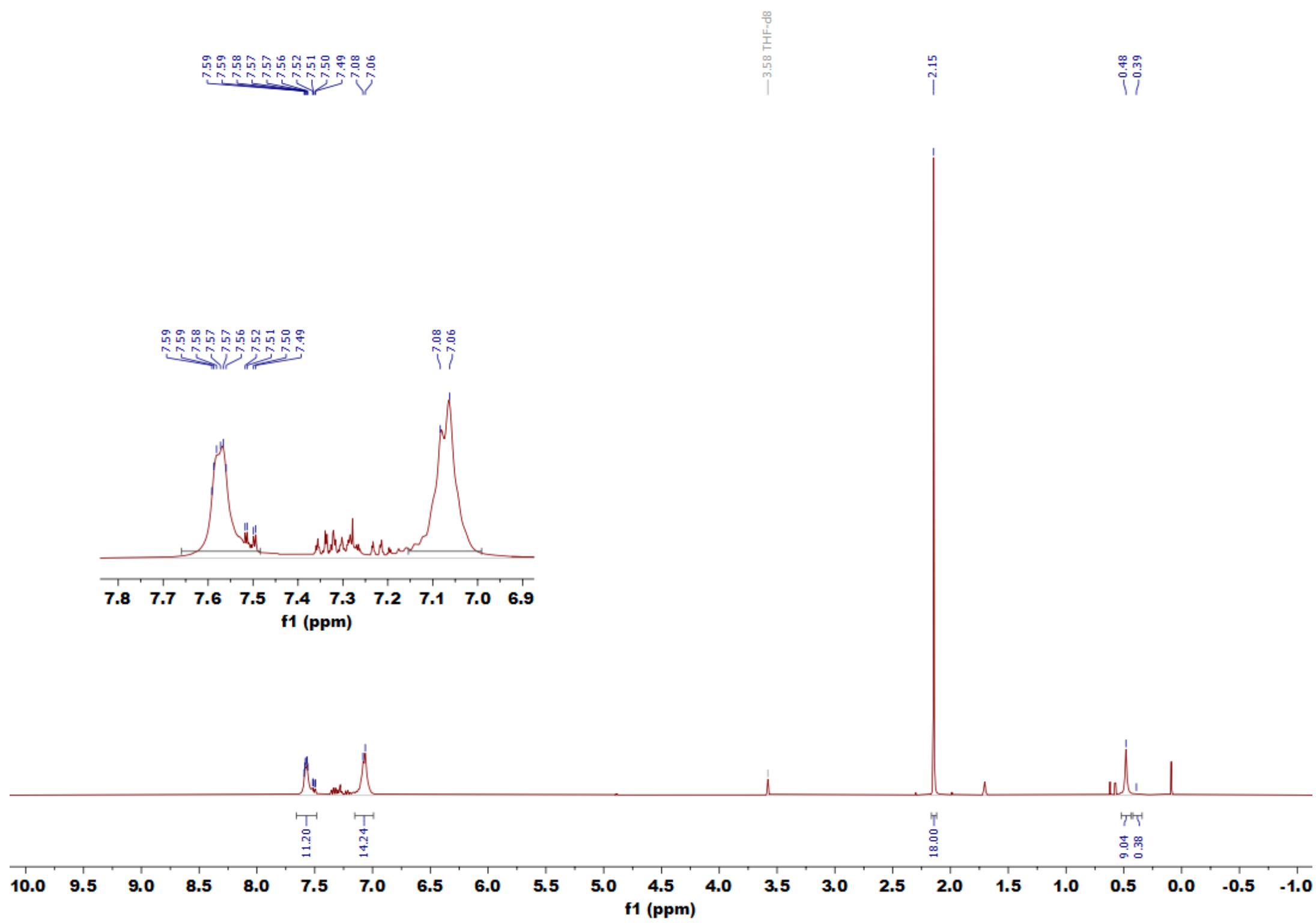

Figure S87. ^{1}H-NMR spectrum of (R^{Ph2})ZnI·LiCl·LiI in THF-d_8 (400 MHz) at 0 h.

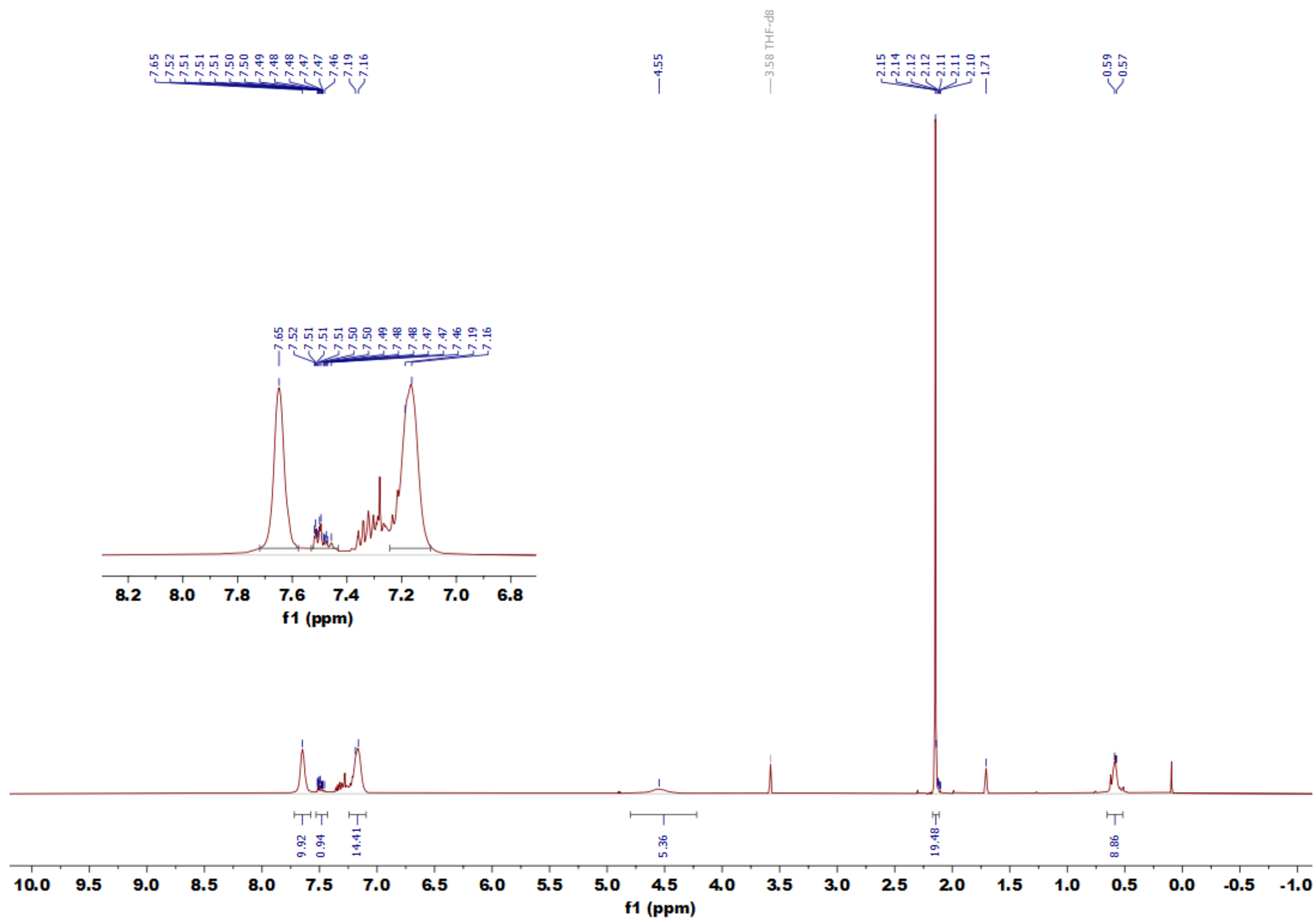

Figure S88. ^{1}H-NMR spectrum of (R^{Ph2})ZnI·LiCl·LiI in THF-d_8 (400 MHz) after exposure for 24 h.

5.3 (R^{Ph3})ZnX·LiX·LiCl (X = OPiv, Cl, Br, I, OAc) systems

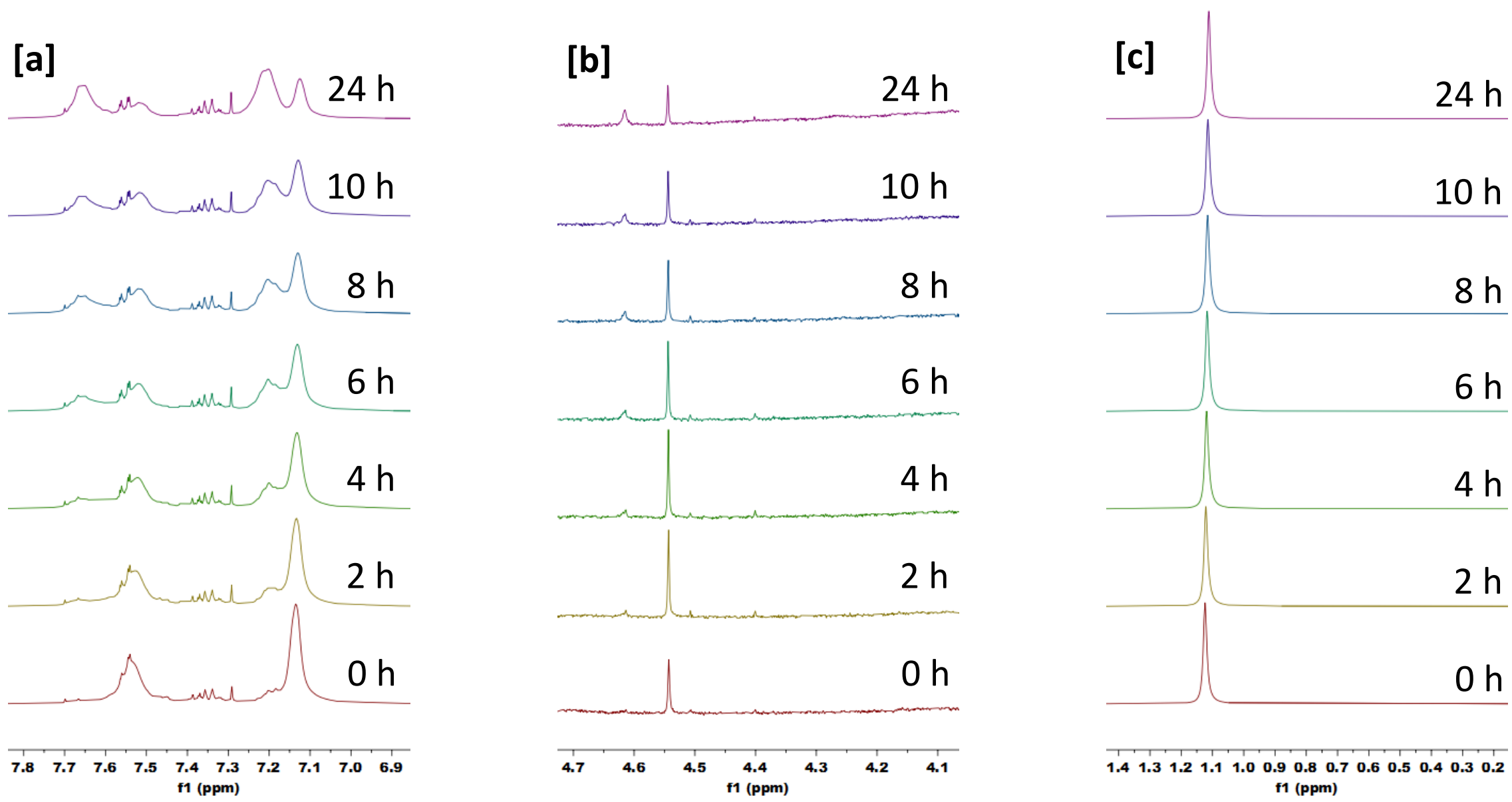

Figure S89. Time-dependent ^{1}H-NMR spectra of (R^{Ph3})Zn(OPiv)·Li(OPiv)·LiCl in THF-d_8 (400 MHz). **[a]** Aryl region. **[b]** Si–OH region. **[c]** OPiv region.

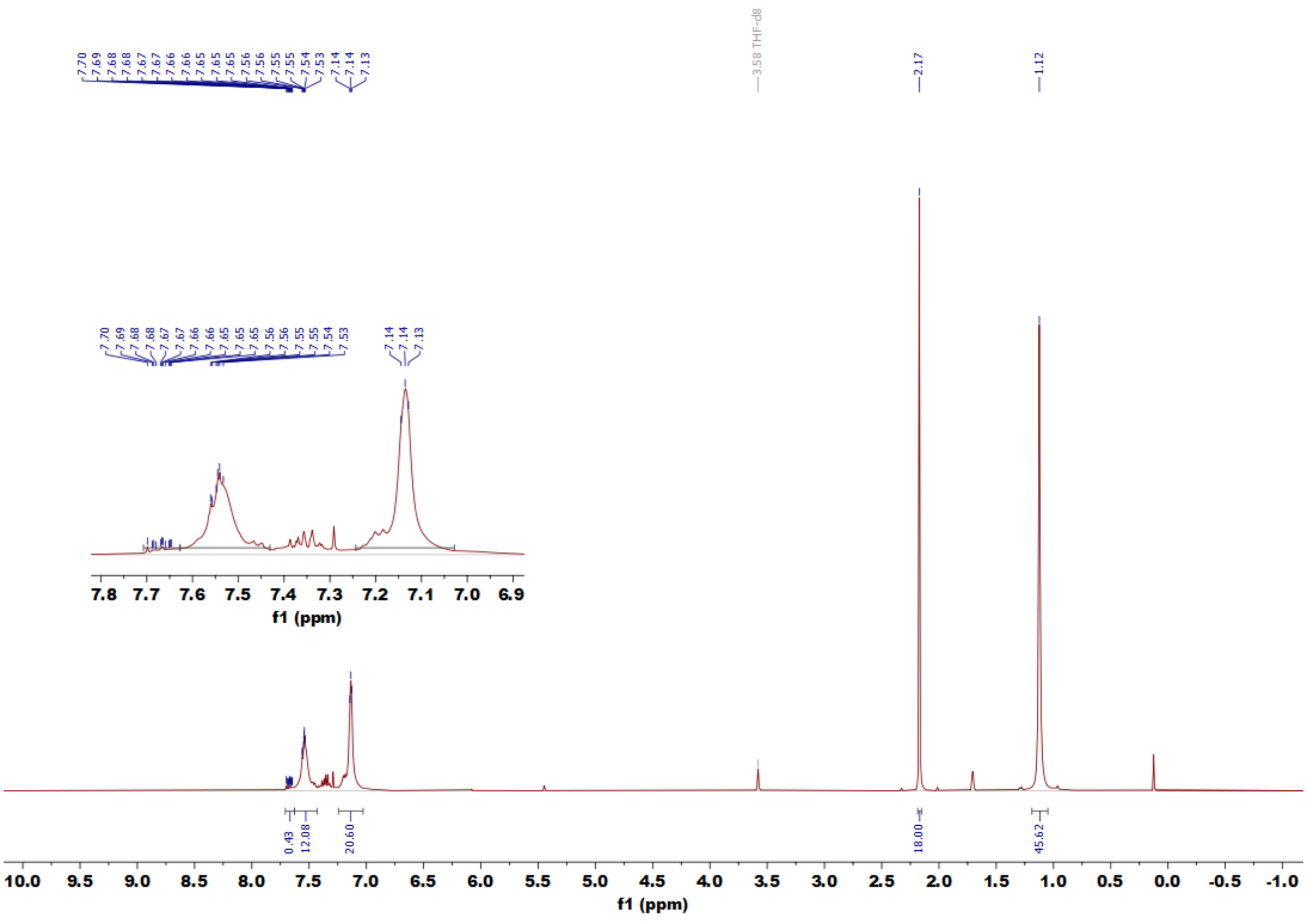

Figure S90. ^{1}H-NMR spectrum of (R^{Ph3})Zn(OPiv)·Li(OPiv)·LiCl in THF-d_8 (400 MHz) at 0 h.

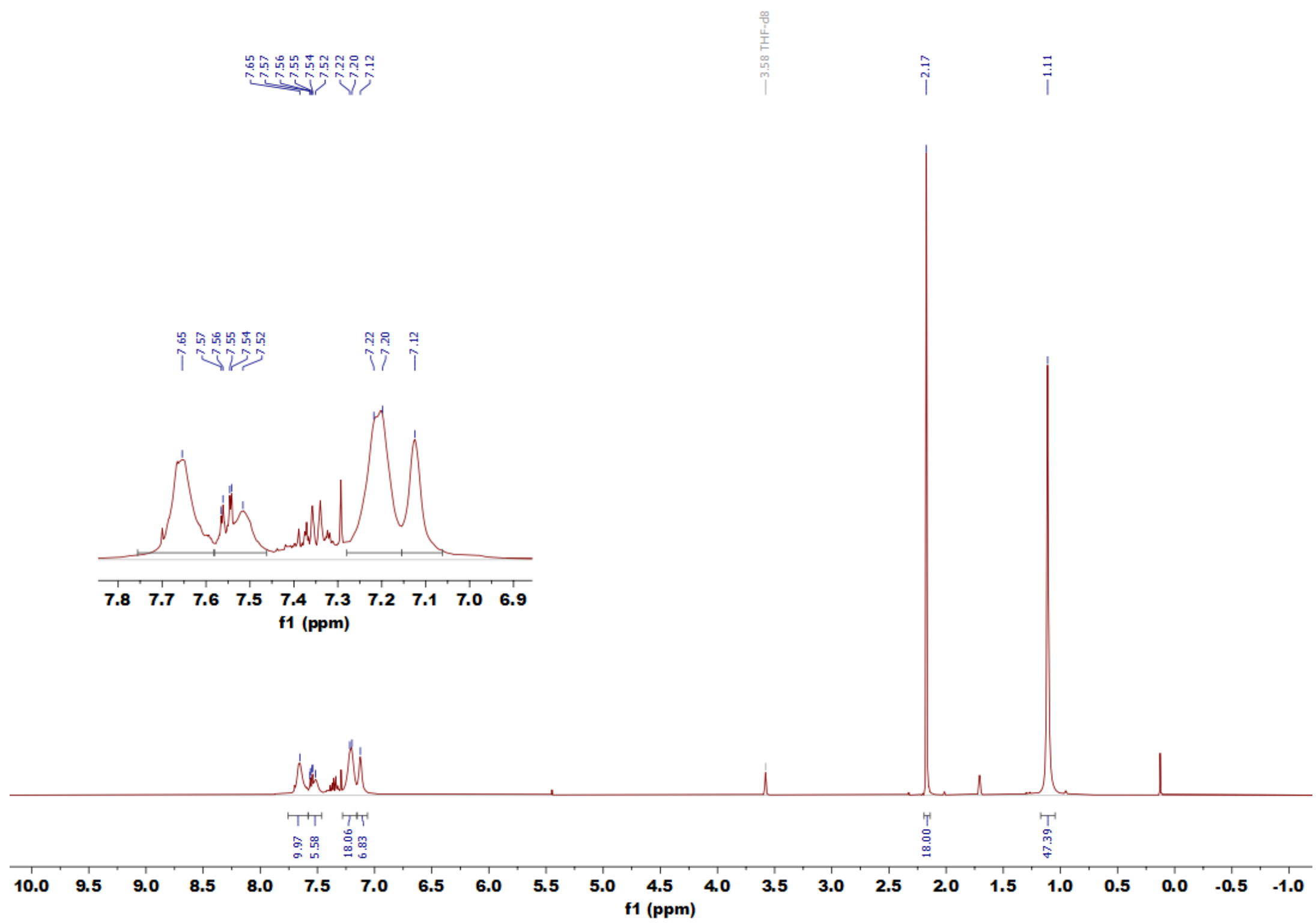

Figure S91. ^{1}H-NMR spectrum of (R^{Ph3})Zn(OPiv)·Li(OPiv)·LiCl in THF-d_8 (400 MHz) after exposure for 24 h.

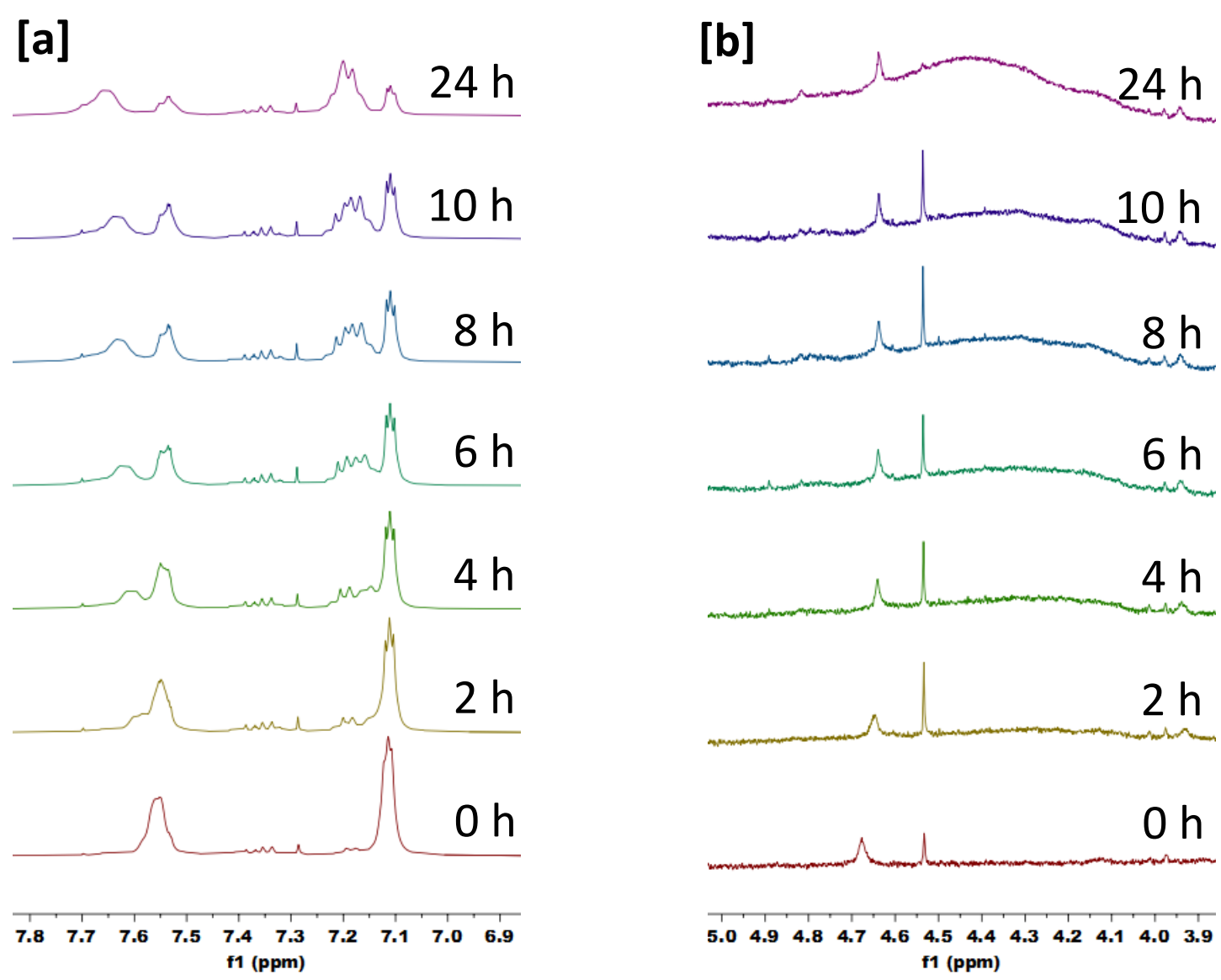

Figure S92. Time-dependent ^{1}H-NMR spectra of (R^{Ph3})ZnCl·2 LiCl in THF-d_8 (400 MHz). **[a]** Aryl region. **[b]** Si–OH region.

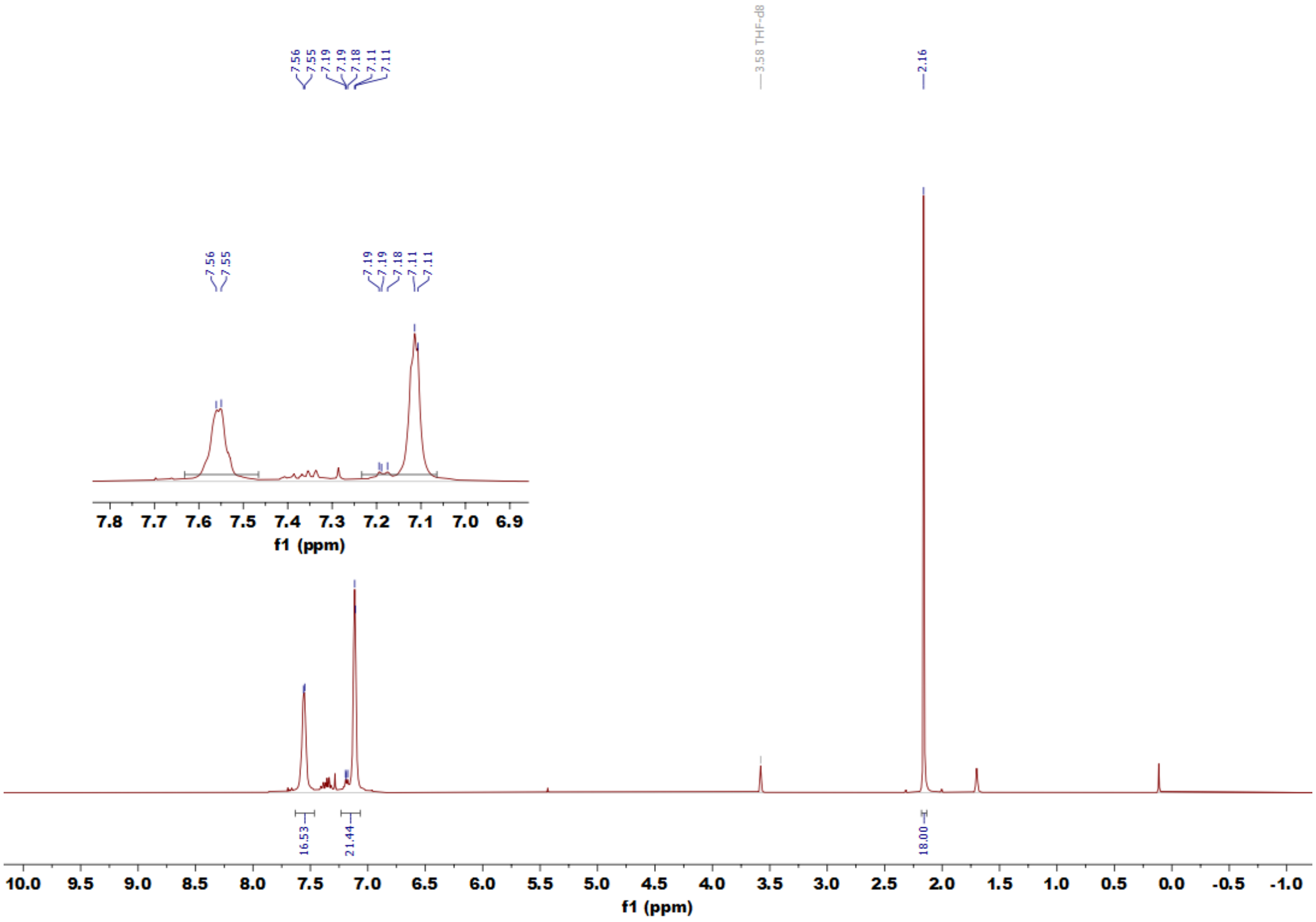

Figure S93. ^{1}H-NMR spectrum of (R^{Ph3})ZnCl·2 LiCl in THF-d_8 (400 MHz) at 0 h.

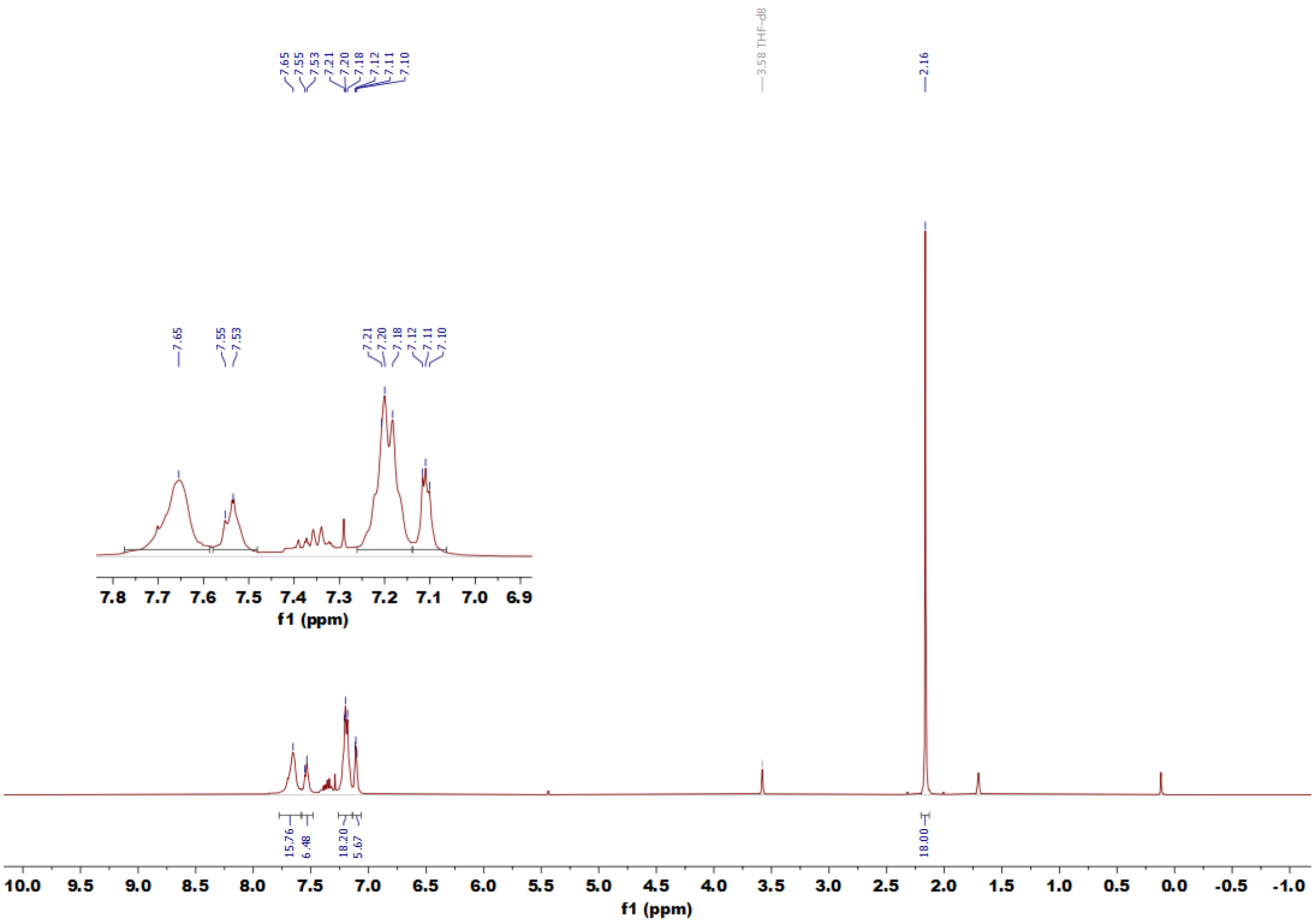

Figure S94. ^{1}H-NMR spectrum of (R^{Ph3})ZnCl·2 LiCl in THF-d_8 (400 MHz) after exposure for 24 h.

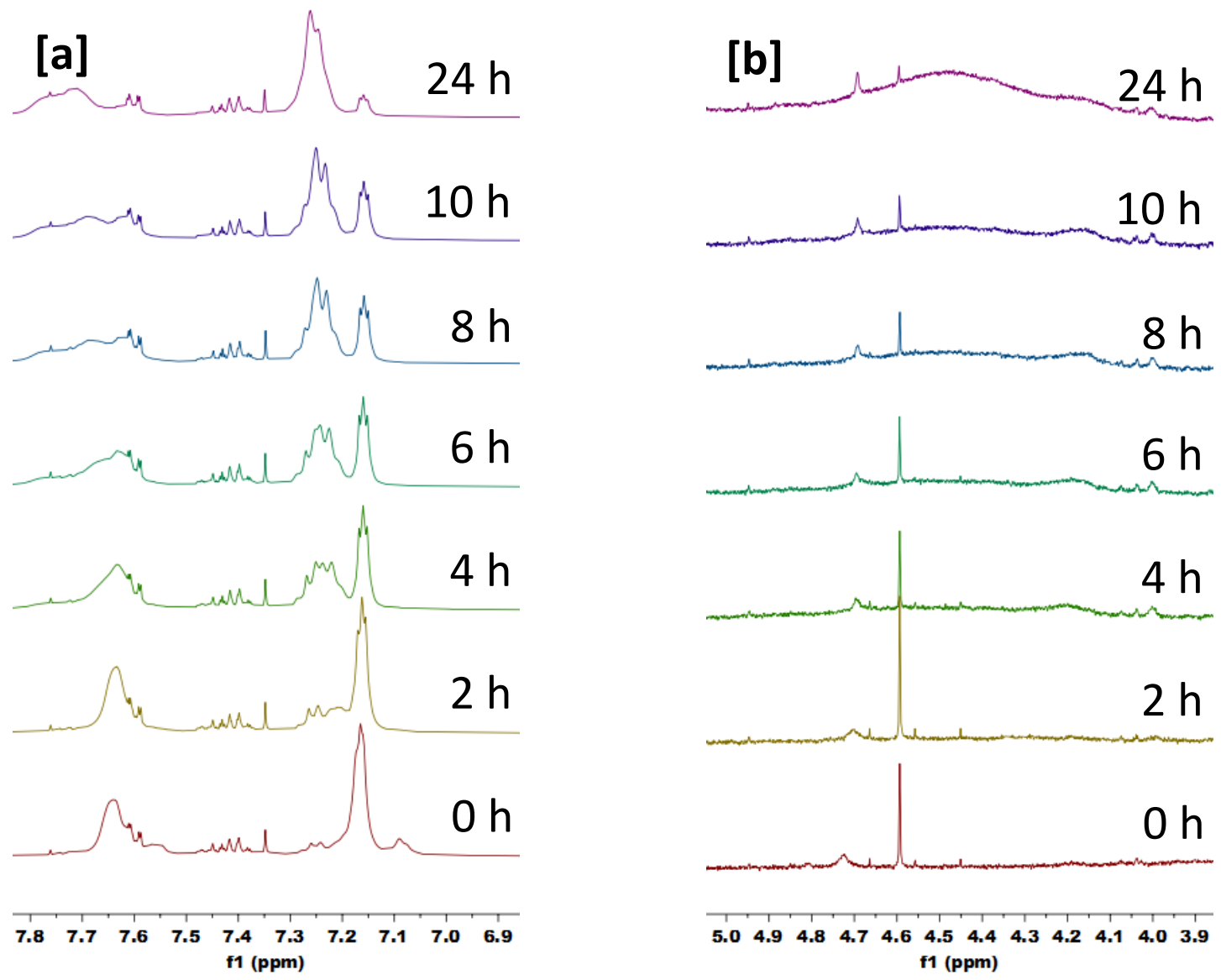

Figure S95. Time-dependent ^{1}H-NMR spectra of (R^{Ph3})ZnBr·LiCl·LiBr in THF-d_8 (400 MHz). **[a]** Aryl region. **[b]** Si–OH region.

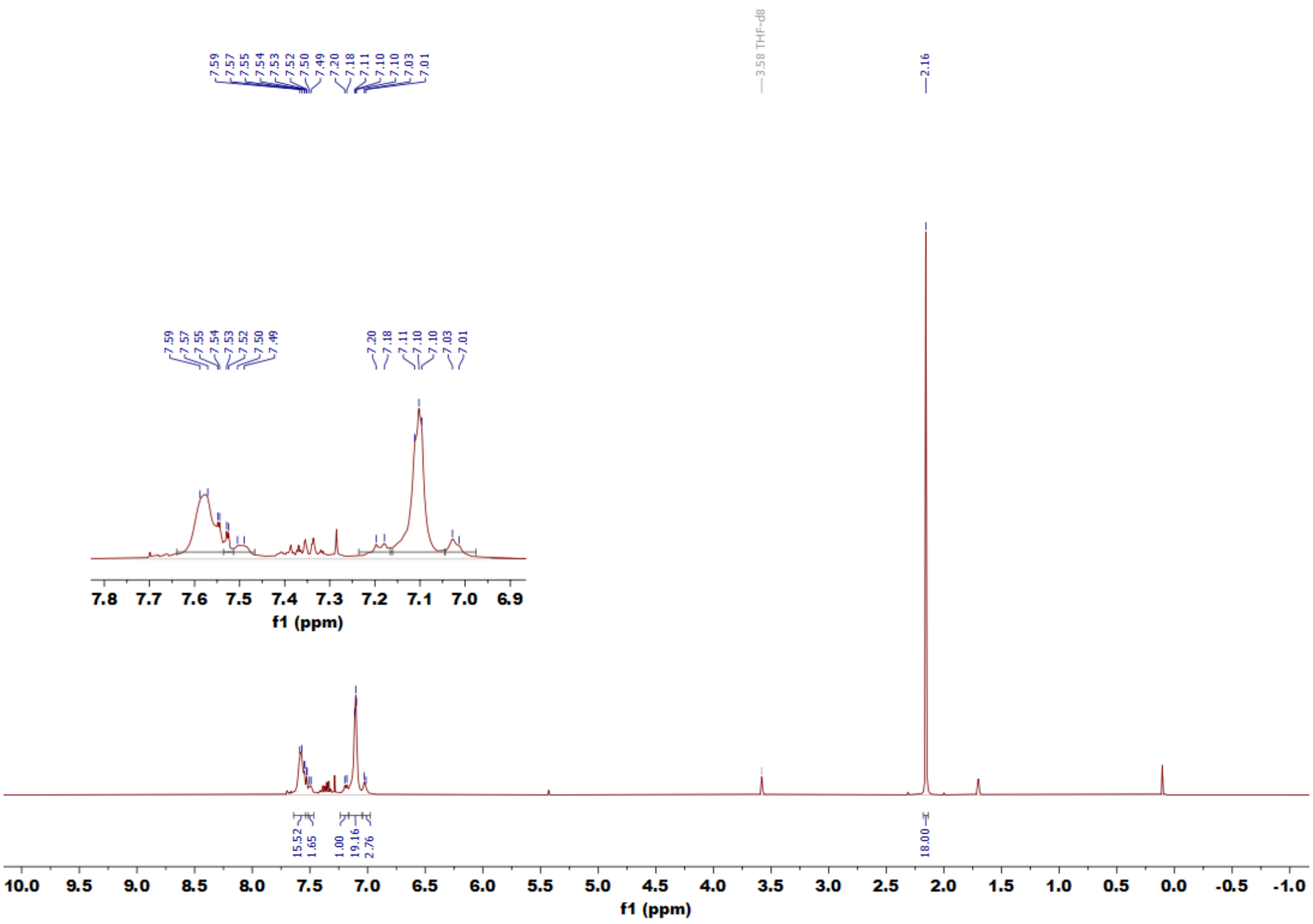

Figure S96. ^{1}H-NMR spectrum of (R^{Ph3})ZnBr·LiCl·LiBr in THF-d_8 (400 MHz) at 0 h.

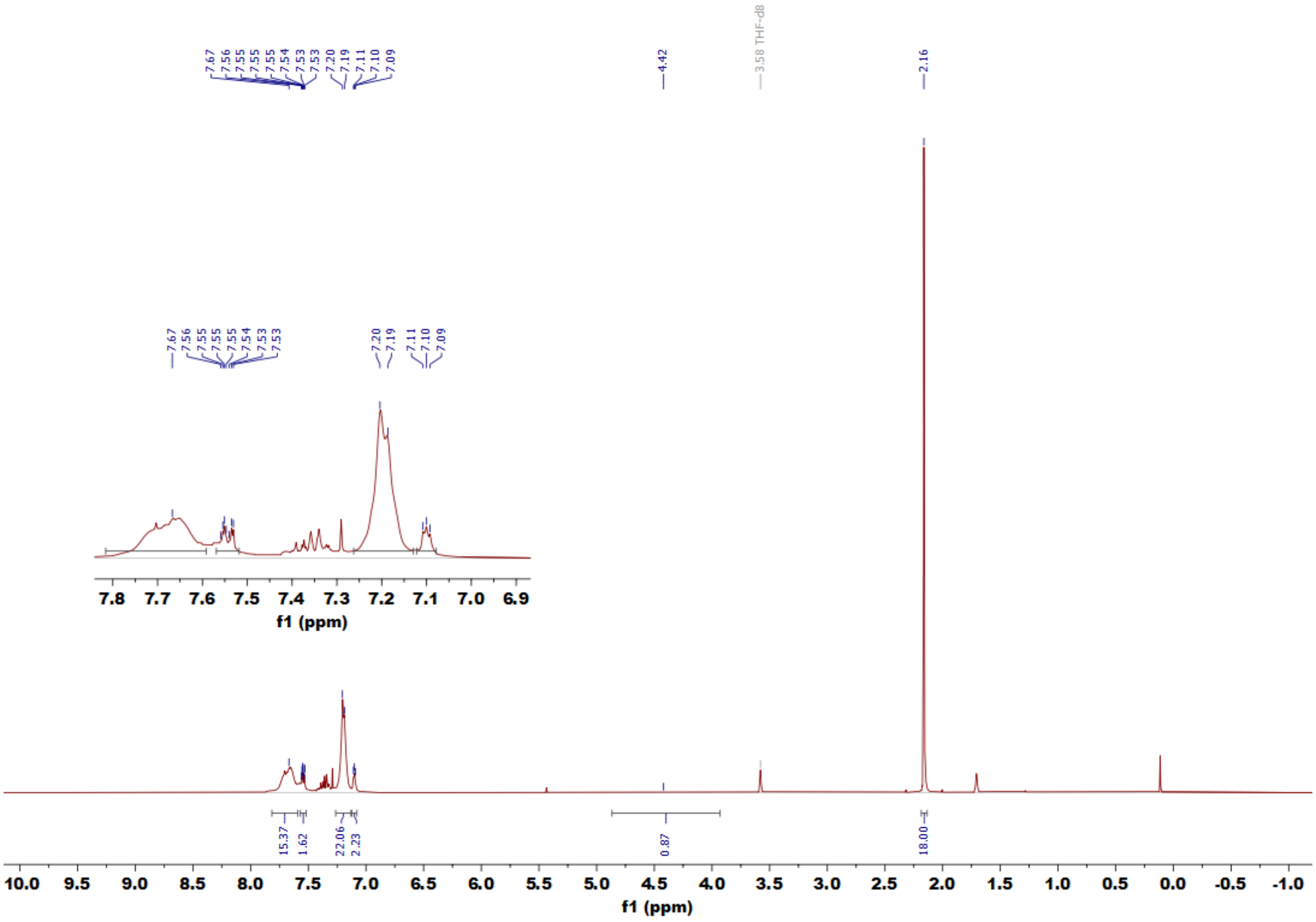

Figure S97. ^{1}H-NMR spectrum of (R^{Ph3})ZnBr·LiCl·LiBr in THF-d_8 (400 MHz) after exposure for 24 h.

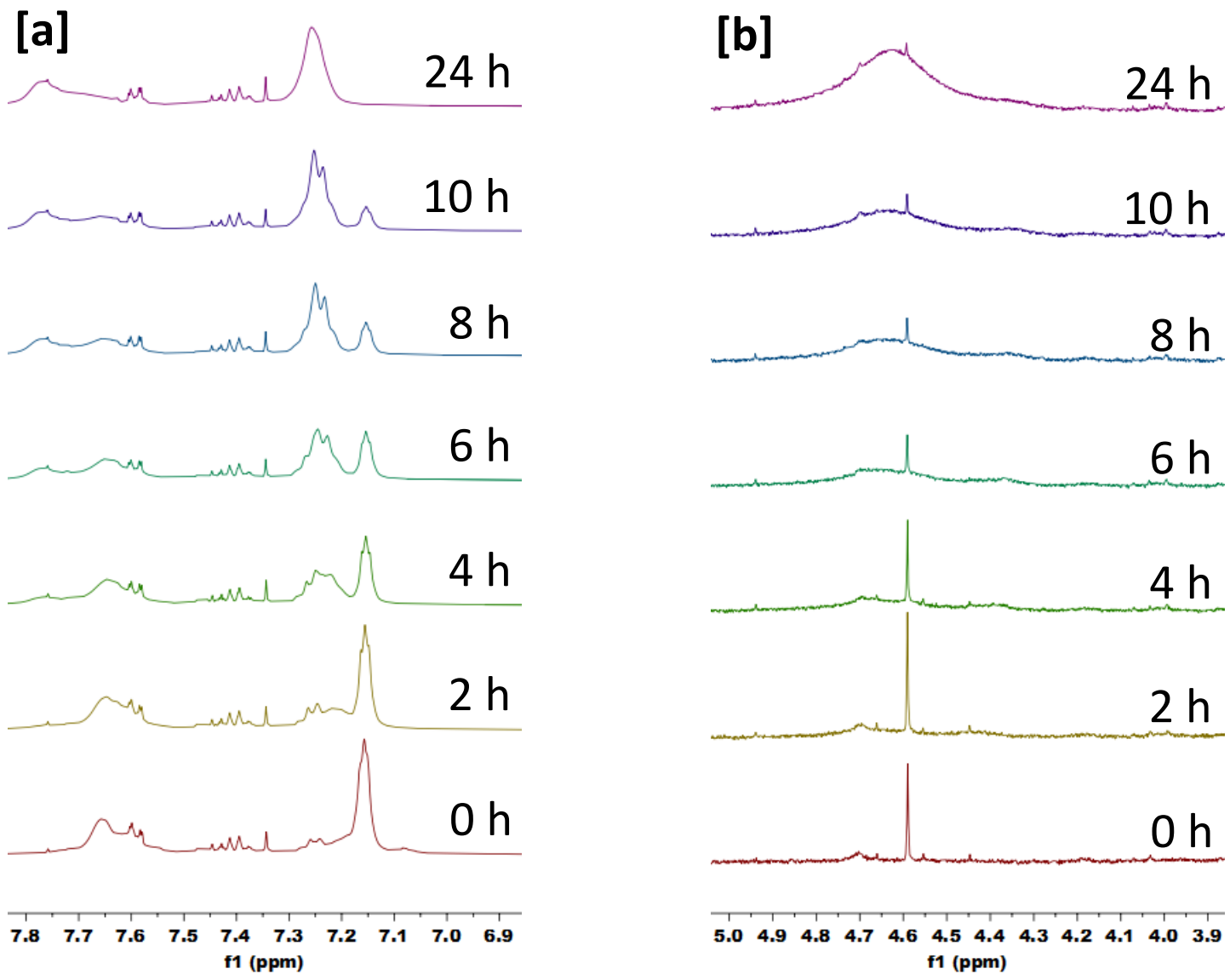

Figure S98. Time-dependent ^{1}H-NMR spectra of (R^{Ph3})ZnI·LiCl·LiI in THF-d_8 (400 MHz). **[a]** Aryl region. **[b]** Si–OH region.

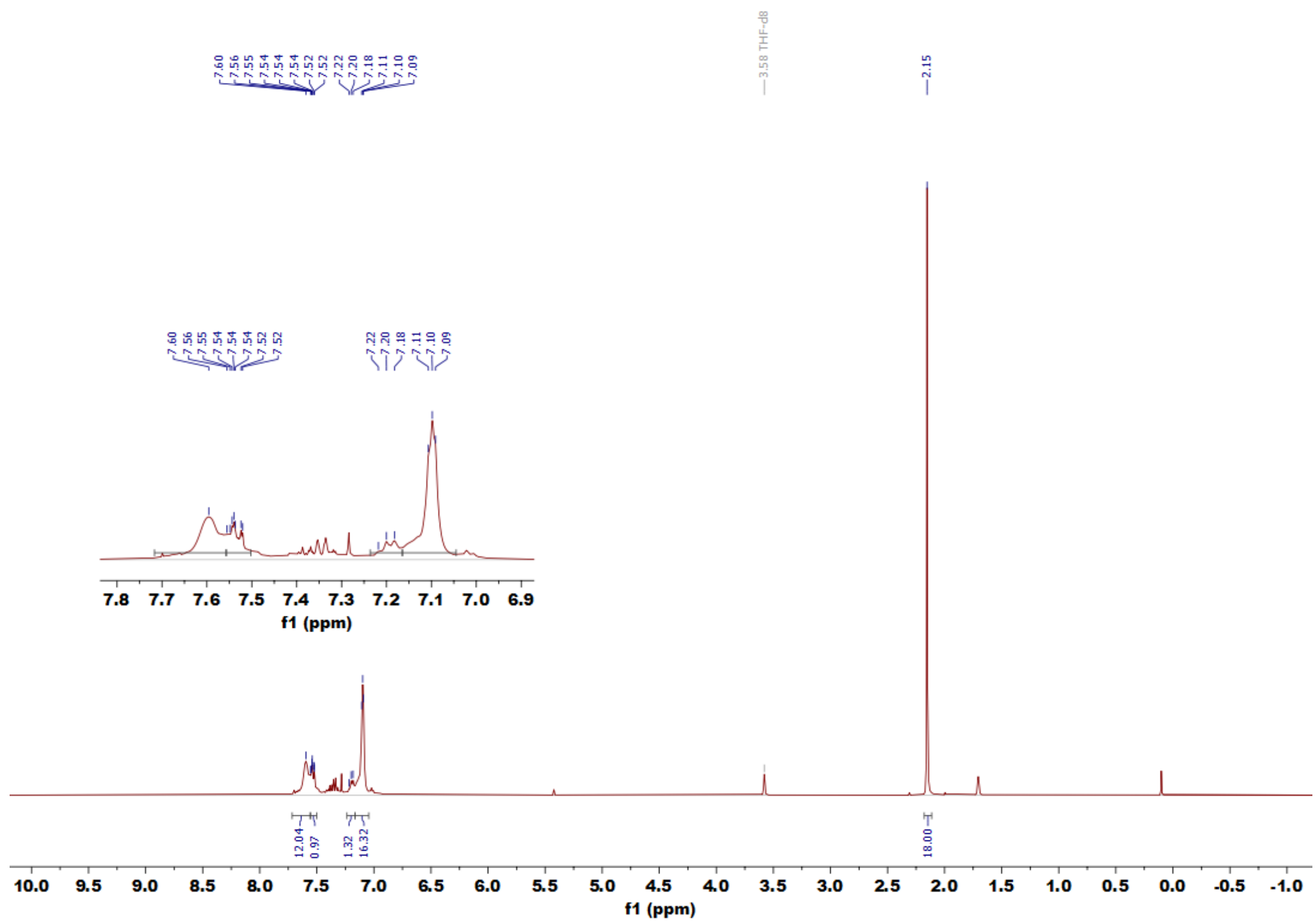

Figure S99. ^{1}H-NMR spectrum of (R^{Ph3})ZnI·LiCl·LiI in THF-d_8 (400 MHz) at 0 h.

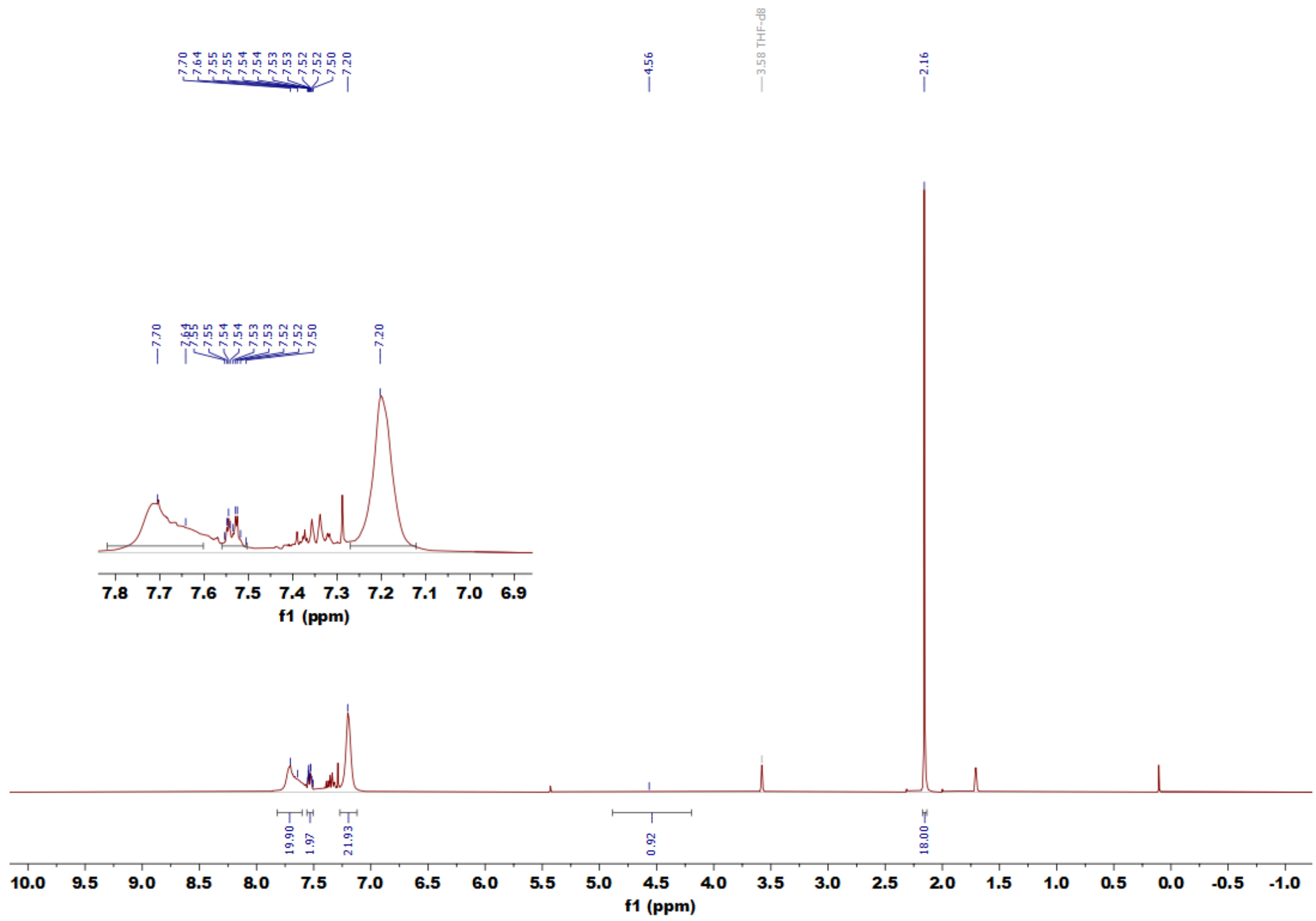

Figure S100. ^{1}H-NMR spectrum of (R^{Ph3})ZnI·LiCl·LiI in THF-d_8 (400 MHz) after exposure for 24 h.

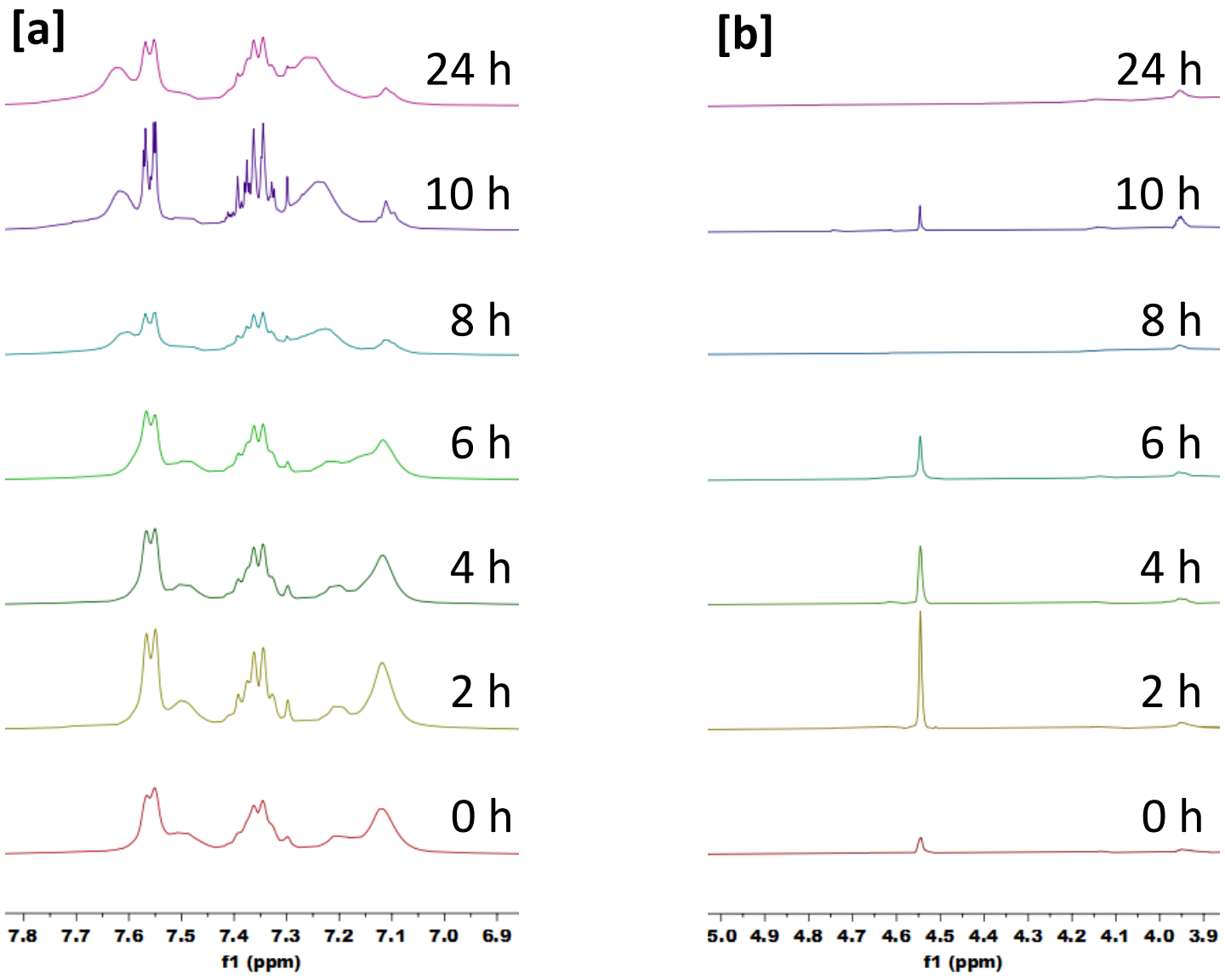

Figure S101. Time-dependent ^{1}H-NMR spectra of (R^{Ph3})Zn(OAc)·Li(OAc)·LiCl in THF-d_8 (400 MHz). **[a]** Aryl region. **[b]** Si–OH region.

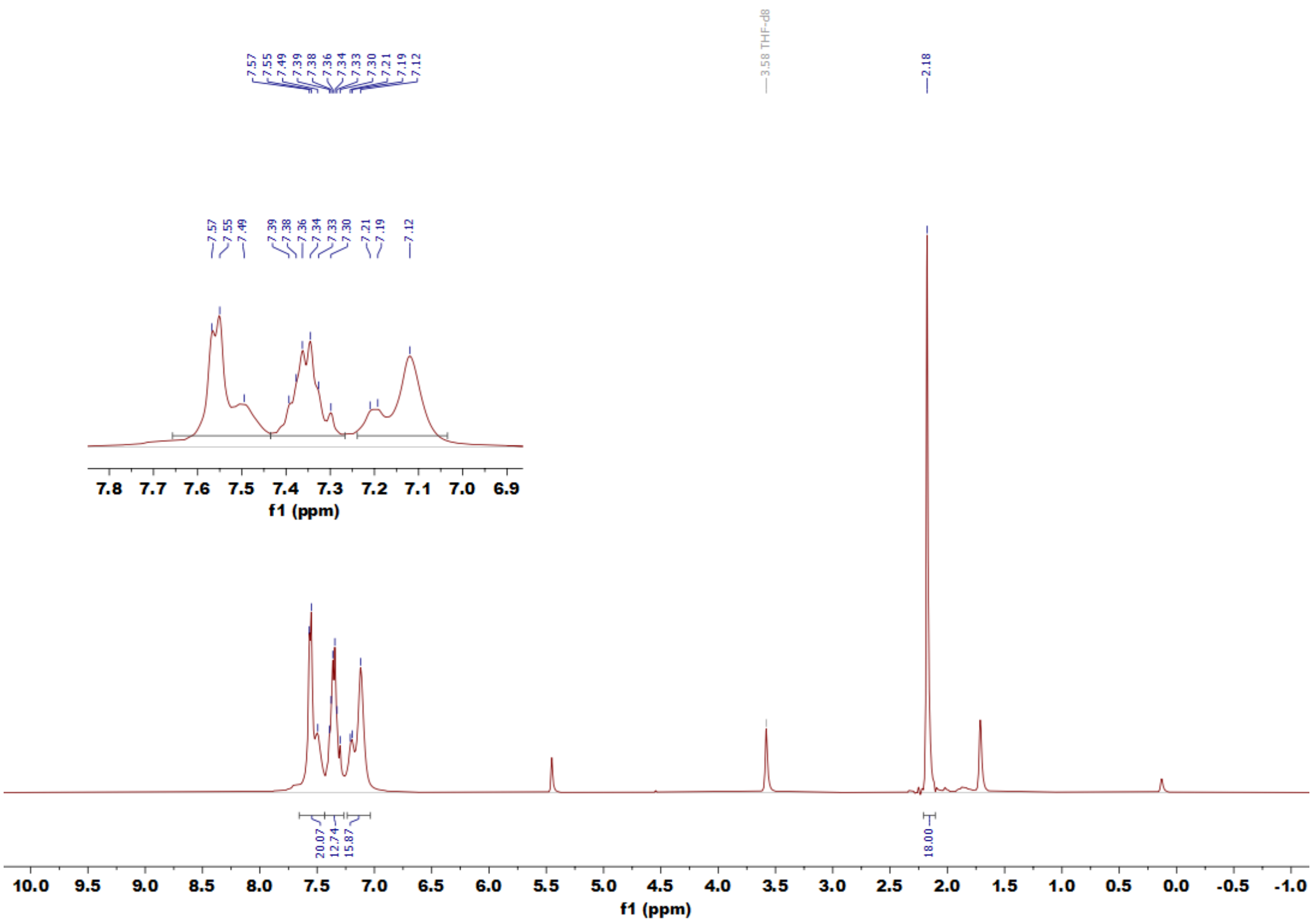

Figure S102. ^{1}H-NMR spectrum of (R^{Ph3})Zn(OAc)·Li(OAc)·LiCl recorded in THF-d_8 (400 MHz) at 0 h.

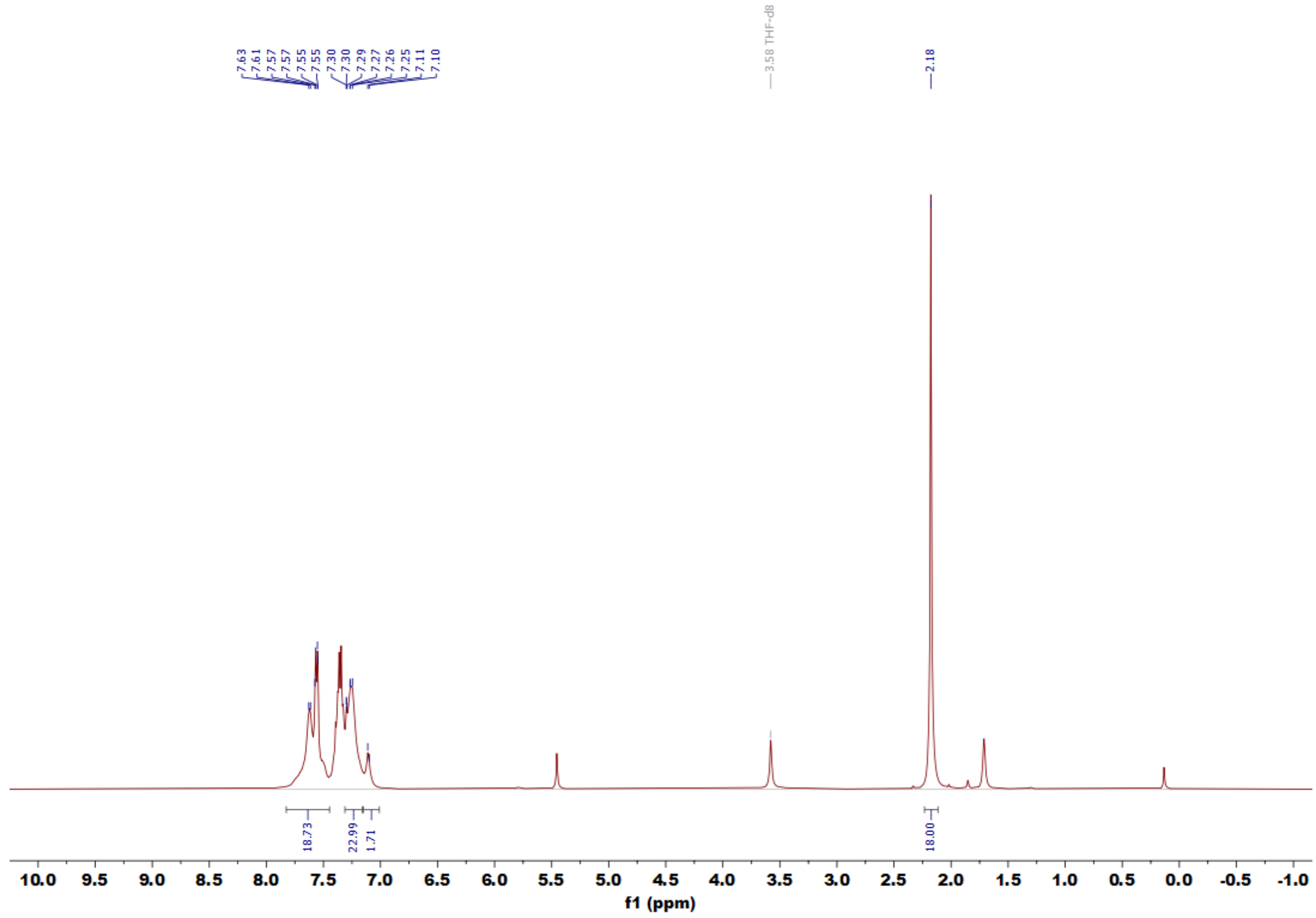

Figure S103. ^{1}H-NMR spectrum of (R^{Ph3})Zn(OAc)·Li(OAc)·LiCl in THF-d_8 (400 MHz) after exposure for 24 h.

6. NMR Studies Supporting the Assignment of Silanol Chemical Shifts

To support the assignment of silanol-related resonances in THF-d_8, NMR spectra of the reaction solution were compared with those of an authentic Ph_3SiOH sample (Figures S104–S107). Commercial Ph_3SiOH exhibits a sharp Si–OH resonance at $\delta_H \approx 6.2$ ppm, which disappears after the addition of D_2O due to exchange processes. After the exposure to ambient atmosphere, the $(R^{Ph3})ZnCl\cdot LiCl$ reaction mixture shows a broader signal at $\delta_H \approx 4.3–4.6$ ppm, which is also affected by the addition of D_2O. When added to the reaction solution, Ph_3SiOH loses its sharp resonance at $\delta_H \approx 6.2$ ppm, indicating rapid exchange and a strong matrix effect. Taken together, the comparison with an authentic Ph_3SiOH sample, the D_2O exchange experiments, and the ESI-mass evidence are consistent with the assignment of the $\delta_H \approx 4.3–4.6$ ppm region to silanol-related exchangeable protons rather than free Ph_3SiOH.

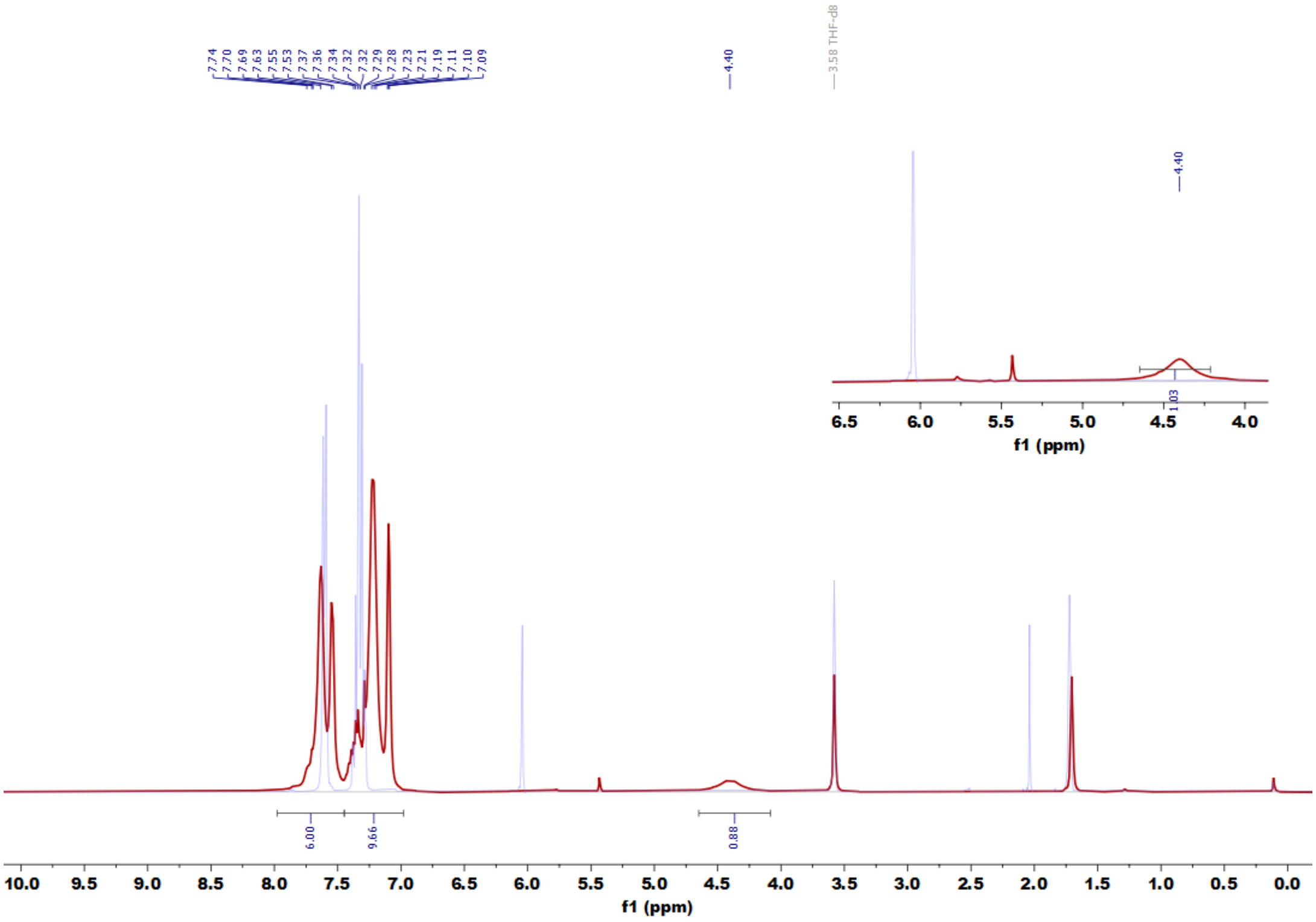

Figure S104. ^{1}H-NMR spectra (400 MHz) of commercial Ph_3SiOH in THF-d_8 (blue) and the reaction solution $(R^{Ph3})ZnCl\cdot LiCl$ in THF-d_8 after exposure for 10 h (red). Commercial Ph_3SiOH exhibits a sharp Si–OH resonance at $\delta_H \approx 6.2$ ppm, whereas the reaction solution shows a broader signal at $\delta_H \approx 4.3–4.6$ ppm, indicating a distinct chemical environment for the exchangeable proton.

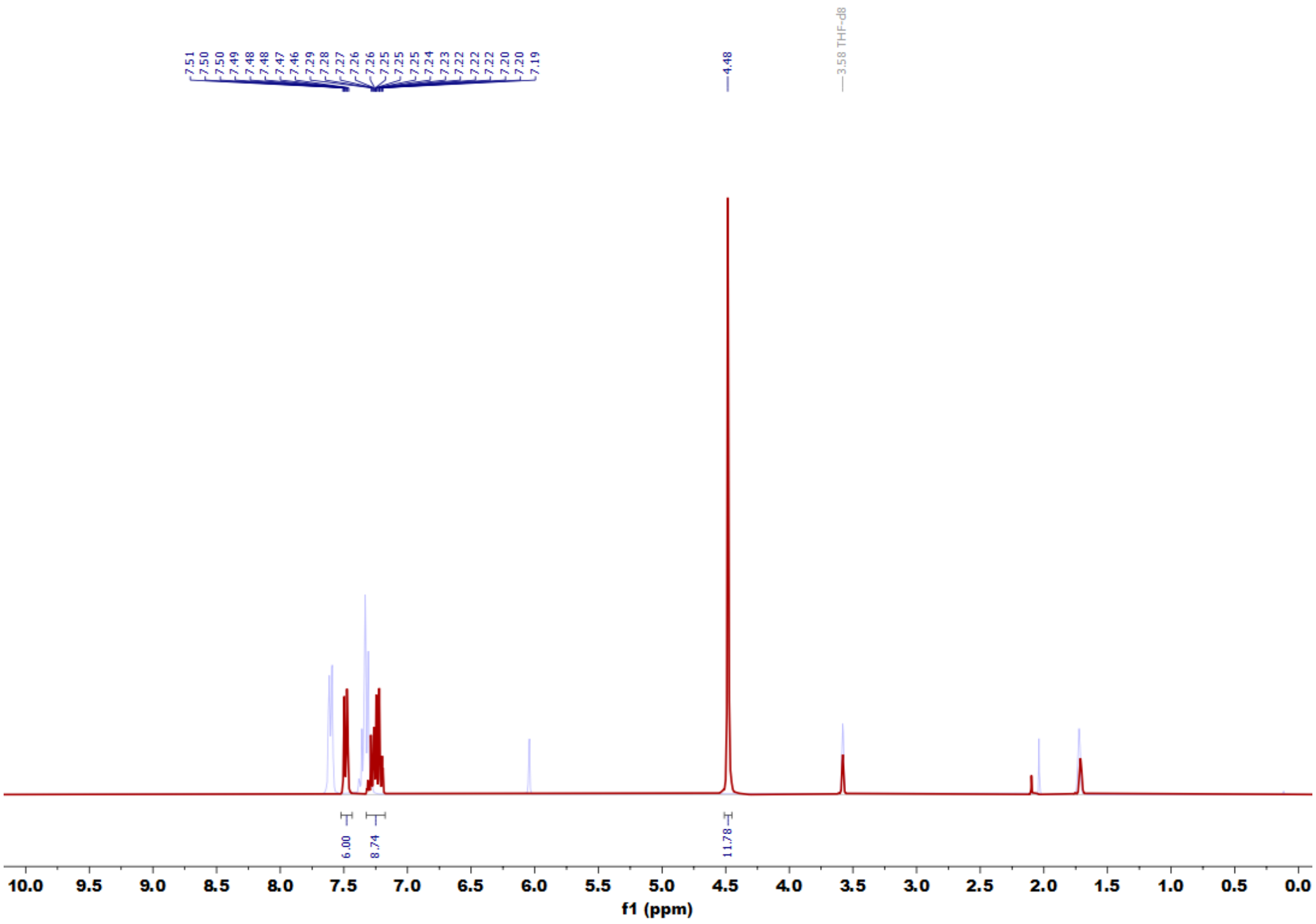

Figure S105. ^{1}H-NMR spectra of commercial Ph_3SiOH in THF-d_8 (400 MHz) before (blue) and after the addition of D_2O (red). The change in the Si–OH resonance upon addition of D_2O confirms its exchangeable-proton character.

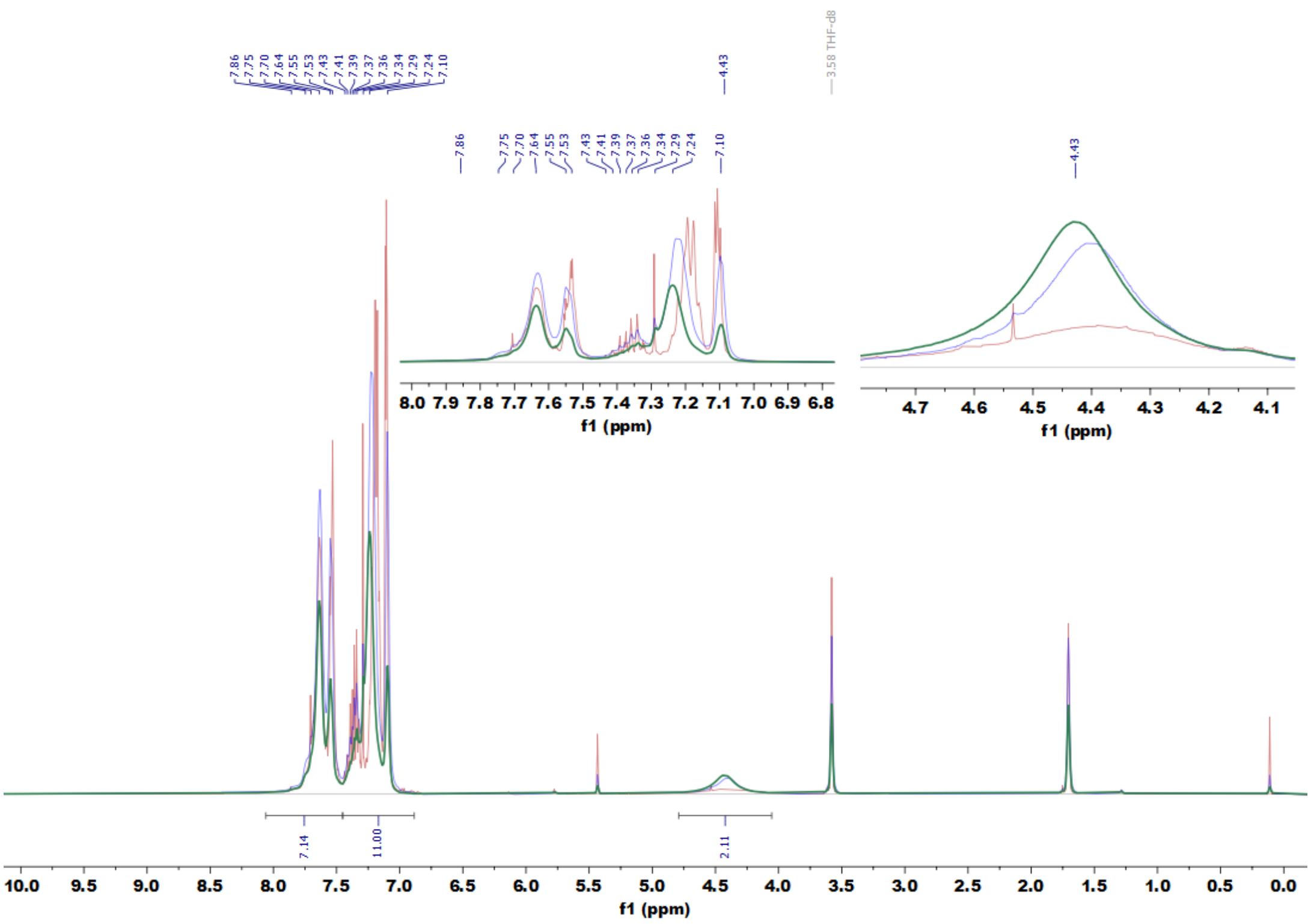

Figure S106. ^{1}H-NMR spectra of the reaction solution of $(R^{Ph3})ZnCl{\cdot}LiCl$ in THF-d_8 (400 MHz) after exposure for 10 h without D_2O (red), after exposure for 10 h with D_2O (blue), and after exposure for 24 h with D_2O (green). Changes in the $\delta_H \approx 4.3$–4.6 ppm region indicate the presence of exchangeable protons in the reaction solution.

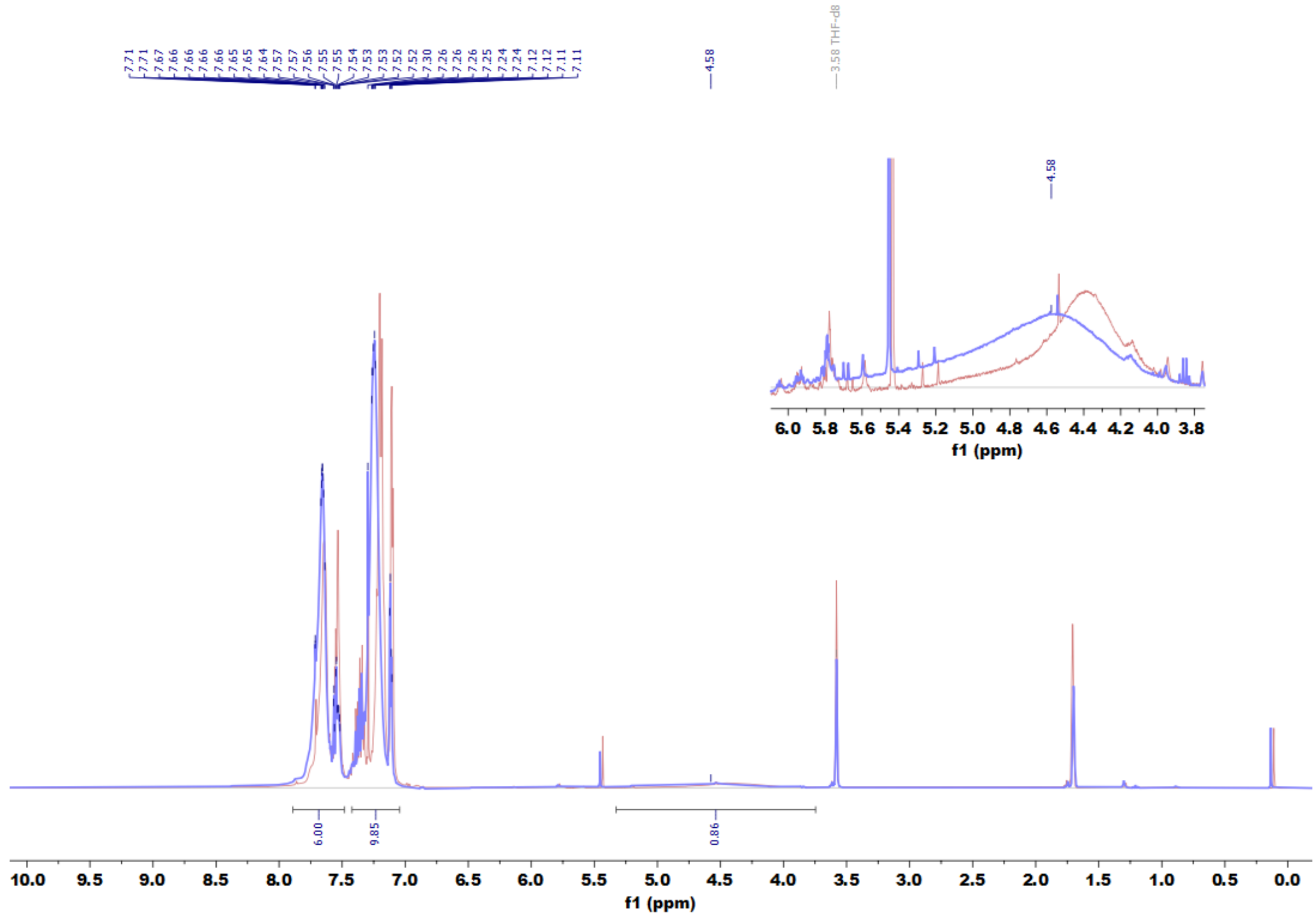

Figure S107. ^{1}H-NMR spectra (400 MHz) of the reaction solution of $(R^{Ph3})ZnCl\cdot LiCl$ in THF-d_8 after exposure for 10 h (red) and after addition of purified Ph_3SiOH (blue). Addition of Ph_3SiOH leads to disappearance of the sharp free Ph_3SiOH resonance without a clear enhancement of the broad signal at $\delta_H \approx 4.3–4.6$ ppm, indicating rapid proton exchange and a strong matrix effect.

7. References

(40) W. G. Kofron, L. M. A. Baclawski, "Convenient Method for Estimation of Alkyllithium Concentrations" *J. Org. Chem*. 41 (1976): 1879–1880.

(41) K. L. Vikse, M. P. Woods, "Pressurized Sample Infusion for the Continuous Analysis of Air- And Moisture-Sensitive Reactions Using Electrospray Ionization Mass Spectrometry" *Organometallics* 29 (2010): 6615–6618.
